# Small-Scale Mineral and Microbial Heterogeneities near a Fumarole at the Furnas Hydrothermal Zone on the Azores

**DOI:** 10.3390/life16071086

**Published:** 2026-06-28

**Authors:** Dirk Schulze-Makuch, Alexander Bartholomäus, Felix Leo Arens, Kai Mangelsdorf, Dirk Wagner

**Affiliations:** 1 Astrobiology Group, Institute of Physics and Astronomy, Technische Universität Berlin, Hardenbergstr. 36A, 10623 Berlin, Germany; felix.leo.arens@googlemail.com; 2 Section Geomicrobiology, GFZ Helmholtz Centre for Geosciences, Telegrafenberg, 14473 Potsdam, Germany; abartho@gfz.de (A.B.); dirk.wagner@gfz.de (D.W.); 3 Department of Plankton and Microbial Ecology, Leibniz Institute of Freshwater Ecology and Inland Fisheries, 16775 Stechlin, Germany; 4 School of the Environment, Washington State University, Pullman, WA 99164, USA; 5 Section Organic and Earth Surface Geochemistry, GFZ Helmholtz Centre for Geosciences, Telegrafenberg, 14473 Potsdam, Germany; k.mangelsdorf@gfz.de; 6 Institute for Geosciences, University of Potsdam, Karl-Liebknecht-Str. 24-25, 14476 Potsdam, Germany

**Keywords:** heterogeneities, microbial diversity, mineralogy, hydrothermal, scaling, fumarole, PLFA, DNA sequencing, extremophiles, microhabitat

## Abstract

The Azores are characterized by intense volcanic activity, creating unique environments such as fumarole sites, where geothermal gases and high temperatures drive distinct chemical and biological processes. To investigate small-scale heterogeneity within such a site, six visually distinct samples were collected within a 30 cm radius at an active fumarole on São Miguel Island. The samples were analyzed for elemental and mineralogical composition, bacterial lipid biomarkers (PLFAs), and microbial community structure using a novel DNA separation technique to specifically target the living microbiome. Despite mineralogical similarities across all samples—predominantly composed of alunite, alkali-feldspar, and quartz—significant microbial heterogeneity was observed. Both PLFA and bacterial iDNA analyses revealed distinct microbial communities associated with specific conditions indicated by the specific colors: red and brown samples were dominated by Proteobacteria and Actinobacteriota, yellow and green by Thermoplasmatota and Actinobacteriota, and white and gray by Crenarchaeota. Interestingly, the gray samples exhibited a broader microbial composition, sharing some taxa with all other samples. These striking color variations are likely driven by differences in both specific mineral composition and microbial pigmentation, reflecting localized biogeochemical processes. Our findings demonstrate that extreme microbial heterogeneity can occur over remarkably small spatial scales within fumarolic systems, underscoring the complex interplay between chemical and biological factors in these dynamic volcanic habitats.

## 1. Introduction

Hydrothermal systems associated with active volcanism represent dynamic environments characterized by steep gradients in temperature, pH, redox conditions, and fluid composition. These gradients can drive intense water–rock interaction and lead to the formation of chemically and mineralogically diverse substrates, particularly in acid-sulfate systems. The Azores archipelago provides an ideal natural laboratory to investigate such hydrothermal processes, as it combines active volcanism with well-developed hydrothermal systems and pronounced geochemical variability.

The Azores Islands are located at the triple junction of the Eurasian, North American, and Nubian plates [1]. The islands are the emerged peaks of various volcanic ridges of the Azores Plateau, which is an area of anomalous oceanic crust thickening [2]. The source of the Azores volcanism has been attributed to the presence of a mantle plume (e.g., [3,4,5,6]), although this is controversial [7,8,9]. The Azores archipelago consists of nine volcanic islands [10], including São Miguel Island, which is comprised of three major trachytic central volcanoes: Furnas (including the older Povoação caldera), Fogo, and Sete Cidades [11].

**Figure 1:**
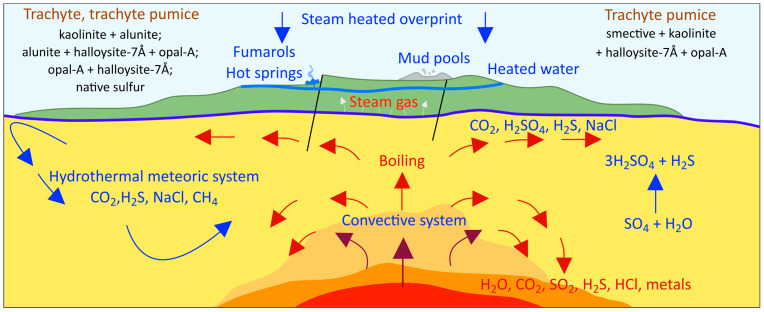
The acid-sulfate alteration model of the active hydrothermal system of the Furnas volcano. During the fluid circulation in the convective meteoric system, aluminum (Al) becomes more soluble than silicon (Si) and low- or high-supersaturated solutions of Si and Al will favor the crystallization of kaolinite or halloysite-7 Å [11,14]. Red arrows indicate warmer waters, blue arrows colder waters. Adapted from [11].

The Furnas volcano hosts an active hydrothermal system, with equilibrium temperatures estimated to range between 200 °C and 275 °C [12]. Thermal water rising to the surface is predominantly meteoric in origin and heated by magmatic vapors [11,13]. The acidic conditions observed in the fumarole and thermal springs are a consequence of H_2_S and S oxidation with production of sulfuric acid at temperatures below 200 °C in a convective meteoric system [12,13]. This acidic fluid circulation in a volcanic or post-volcanic environment interacts with the minerals from the host rocks, forming new minerals as a result of that interaction [11,14,15]. In the Caldeira das Furnas, trachytic rocks are altered by sulfuric acid solutions into assemblages dominated by alunite, clay minerals, and sinter rocks corresponding to the acid-sulfate alteration [11], similar to alteration systems described on Mars [16]. Bobos and Gomes [11] identified three distinct mineral assemblages: kaolinite + alunite; alunite + opal-A +/− halloysite-7Å +/− native sulfur; and opal-A +/− alunite (Figure 1).

Such alteration processes produce chemically and mineralogically heterogeneous substrates that can vary at very small spatial scales due to localized fluid flow, gas emission, and reaction pathways [11,15]. These small-scale heterogeneities provide a structurally and chemically diverse template for subsequent microbial colonization and activity.

In addition to their geochemical complexity, hydrothermal systems host diverse microbial communities adapted to extreme conditions, including high temperatures, low pH, and elevated concentrations of dissolved ions. These communities are typically dominated by thermophilic and acidophilic microorganisms, including members of the Archaea such as Crenarchaeota and Thermoplasmatota, as well as bacteria such as Proteobacteria and Actinobacteriota [17,18,19]. Many of these organisms are chemolithoautotrophs that derive energy from the oxidation or reduction of sulfur and iron compounds, processes that are central to hydrothermal biogeochemistry [20,21,22]. Microbial metabolisms can actively influence mineral formation and transformation through sulfur oxidation, iron redox cycling, and biofilm formation, thereby contributing to localized geochemical heterogeneity [21,23].

At the same time, mineral surfaces and geochemical conditions strongly control microbial colonization and activity. Distinct mineral phases and microenvironmental conditions create specific ecological niches, resulting in spatially structured microbial communities even over very small distances [24,25,26]. This tight coupling between mineralogy and microbiology is often reflected in visible color variations, which may arise from mineral precipitates such as iron oxides or sulfur phases but also from microbial pigments and biofilms [27,28]. Despite increasing recognition of the coupling between geochemical and microbial processes in hydrothermal environments, the extent to which microbial communities vary at centimeter to sub-decimeter scales remains poorly constrained. Previous studies have demonstrated that microbial communities can be highly heterogeneous in hydrothermal systems, but most investigations have focused on larger spatial scales or bulk samples, potentially masking fine-scale variability [18,19]. In particular, it remains unclear to what degree visually distinct microhabitats, such as differently colored mineral patches, correspond to distinct microbial assemblages and metabolic potentials.

To address this question, we investigated a fumarolic site in the Furnas hydrothermal system, where strongly contrasting color patches occur within a distance of less than 30 cm. Six samples representing these visually distinct microenvironments were analyzed for their elemental and mineralogical composition as well as their microbial community structure. To resolve both total and potentially active microbial populations, we combined phospholipid fatty acid (PLFA) analysis with high-resolution 16S rRNA gene sequencing and a DNA separation approach distinguishing intracellular (iDNA) from extracellular DNA (eDNA).

The aim of this study is to assess how small-scale mineralogical and geochemical heterogeneities are linked to microbial community composition and to evaluate whether visually distinct mineral patches represent functionally distinct microbial habitats. By focusing on centimeter-scale variability, this work provides insight into the fine-scale structuring of microbial life in hydrothermal systems and highlights the importance of microscale heterogeneity for understanding biogeochemical processes in extreme environments.

## 2. Materials and Methods

### 2.1. Study Site Description

**Figure 2:**
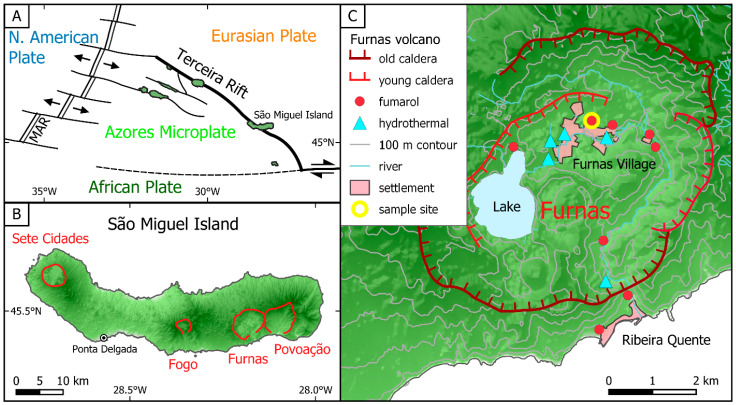
(**A**) Simplified tectonic map of the Azores archipelago with the Terceira Rift east of the Mid-Atlantic Ridge (MAR) leading to the volcanic activity in this region. San Miguel Island is situated on the rift system, which led to (**B**) former volcanic eruptions forming four calderas. (**C**) One of the calderas is the Furnas caldera, which still exhibits fumaroles and hydrothermal springs [29,30], with the sampling location marked.

**Figure 3:**
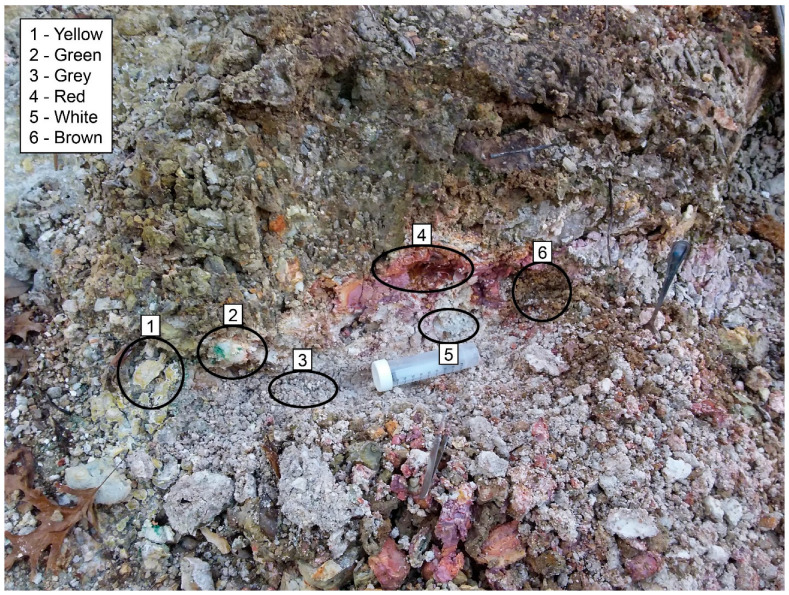
Samples taken at an active fumarole area at the Furnas village caldera (scale: 50 mL centrifuge tube). All samples were taken within 30 cm from each other.

Samples were taken at the Furnas village caldera (Figure 2). Different colorations were noted on the outer wall of the caldera, and six samples with different colors (yellow, green, gray, red, white, and brown) were taken within each other’s vicinity at a maximum distance of 30 cm from Sample 1 to 6 (Figure 3).

### 2.2. Chemical Analyses

Samples were taken with gloves under sterilized conditions and transported to the lab at the Technical University Berlin, where they were stored for further analysis. For ion analysis, sample aliquots were dried at 60 °C, sieved dry to <2 mm grain size, and leached in duplicates with a 1:10 ratio (sample:water (*w*/*w*)) for ion analysis. Anionic species (Cl^−^, NO_3_^−^, SO_4_^2−^) were measured by ion chromatography (IC; DIONEX DX-120 ion chromatograph, Thermo Fisher Scientific Inc., Waltham, MA, USA). Cations (Na^+^, K^+^, Ca^2+^, Fe^2+^, Mg^2+^, NH_4_^+^, and Al^3+^) were determined by inductively coupled plasma optical emission spectrometry (ICP-OES) (iCAP 6000 ICP Spectrometer, Thermo Fisher Scientific, Waltham, MA, USA). Samples were measured in duplicate, and blanks were measured alongside the samples for quality control.

The bulk mineralogy was analyzed via X-ray Diffraction (XRD). First, 5 g sample aliquots were dried at 60 °C and ground to powder. XRD analysis was performed by using a D2 Phaser (Bruker, Billerica, MA; USA) powder diffractometer. The X-ray source is a Cu Kα radiation (Kα_1_: λ_1_ = 1.540598 Å; Kα_2_: λ_2_ = 1.54439 Å) with a performance of 30 kV and 10 mA. A step interval of 0.013° 2Θ with a step-counting time of 20 s was used at a scanning range from 5° to 90° 2Θ. Evaluation was conducted semi-quantitatively using the “Powder Diffraction File Minerals 2019” (International Centre of Diffraction Data) together with the software High Score 5.2 from PANalytical (Almelo, The Netherlands).

### 2.3. PLFA Analysis

Freeze-dried and ground sample material was extracted with a solvent mixture of methanol (MeOH)/dichloromethane (DCM)/ammonium acetate buffer (pH 7.6) at a ratio of 2:1:0.8 (*v*/*v*) using a flow blending system for 5 min [31]. For sediment separation, the solution was centrifuged for 10 min at 2500 rpm. Subsequently, the sample was re-extracted twice with solvent mixture, and all extracts were combined in a separation funnel. For compound quantification, an internal standard was added (PC_54_, deuterated phosphatidyl choline). To separate the organic phase from the aqueous phase, the solvent mixture was changed to a ratio of 1:1:0.9 (*v*/*v*). After the organic phase was separated, the aqueous phase was re-extracted twice with DCM. The combined organic phases were evaporated using a TurboVap 500 (Zymark, Hopkinton, MA, USA).

Due to its complexity, the extract was fractionated using two chromatographic columns in sequence. The upper column was filled with 1 g silica gel (63–200 µm) and 0.5 g sodium sulfate as the top layer, and a lower column was filled with 1 g Florisil (150–250 µm). A low polar lipid fraction was obtained using 20 mL chloroform, a free fatty acid fraction using 50 mL methyl formate with 0.025% glacial acetic acid and a glycolipid fraction using 20 mL acetone. The final phospholipid fraction was eluted only from the upper column using 25 mL methanol. To improve the recovery of phospholipids, the silica gel column material was re-extracted twice by a polar solvent mixture of methanol and water (1:0.6). For phase separation, the above-mentioned Bligh and Dyer [31] procedure was applied. The combined PL fractions were evaporated to dryness using a TurboVap system and, finally, a gentle stream of nitrogen.

To obtain the phospholipid fatty acids (PLFAs), an ester cleavage procedure was applied. Subsequently, the methylated PLFAs were measured using a Trace Gas Chromatograph (GC) 1310 (Thermo Fisher Scientific, Waltham, MA, USA) coupled to a TSQ 9000 mass spectrometer (MS. Thermo Fisher Scientific, Waltham, MA, USA). The GC was equipped with a cold injection system operating in the splitless mode and an SGE BPX 5 fused-silica capillary column (50 m length, 0.22 mm ID, 0.25 µm film thickness) using the following temperature conditions: initial temperature 50 °C (1 min isothermal) and heating rate 3 °C/min to 310 °C, held isothermally for 30 min. Helium was used as carrier gas with a constant flow of 1 mL/min. The injector temperature was programmed from 50° to 300 °C at a rate of 10 °C/s. The MS operated in the electron impact mode at 70 eV. Full-scan mass spectra were recorded from *m*/*z* 50 to 650 at a scan rate of 1.5 scans/s.

### 2.4. DNA Analysis

#### 2.4.1. Extraction of Extracellular (eDNA) and Intracellular (iDNA) DNA

DNA was extracted according to the method of Medina Caro et al. [32], which was slightly modified as described below. All samples were processed in triplicate and included one blank control, except the red sample from sample location #4 (Figure 3) without a replicate due to limited sample material. In a 15 mL sterile conical tube, 5 g of rock material and 0.4 g of polyvinylpolypyrrolidone (PVPP, Sigma-Aldrich (St. Louis, MO, USA), order No. 77627 (6)) were carefully suspended in 5–6 mL of cold (4 °C) sodium phosphate (NaP) buffer (Na_2_HPO_4_ and NaH_2_PO_4_, 0.12 M, pH 8) to form a slightly viscous slurry [33]. The tube was chilled on ice for 1 min and then shaken twice for 5 min at 150 rpm in a horizontal position on an orbital shaker with cooling on ice in between for 3 min. After a centrifugation step (500 *g*, 4 °C, 10 min, swing-out rotor) the supernatant was transferred to a sterile 15 mL conical tube and kept on ice. The remaining pellet was resuspended in 3.0–3.5 mL NaP buffer, and the separation procedure was repeated three more times with all supernatants pooled in one tube (6–8 mL).

#### 2.4.2. eDNA and iDNA Separation

After centrifugation (4643 *g*, 4 °C, 1 h, swing-out rotor), the supernatant representing the eDNA fraction was transferred to a new tube, and the pellet containing the intact cells with the iDNA were kept on ice. The supernatant was passed through a 0.2 μm syringe filter (VWR international, Radnor, PA, USA; cellulose acetate) pre-rinsed with 500 μL NaP buffer. After passing the sample, the filter was rinsed with another 500 μL NaP buffer and then added to the filtered eDNA solution. The filtrate was collected in a sterile 50 mL conical tube and kept on ice until further processing. The iDNA containing pellet was carefully resuspended in 1 mL of NaP buffer, transferred to a sterile 2 mL reaction vial (Eppendorf, Hamburg, Germany; low binding), and centrifuged for 20 min at 12,000 *g* to remove any remaining eDNA.

#### 2.4.3. iDNA Extraction

The supernatant was discarded, and the pellet suspended in 750 μL NaP buffer by incubation in a thermal shaker for two periods of 5 min at 70 °C and 250 rpm with cooling on ice in between for 2 min. The suspension was transferred to a PowerBead tube (Mo Bio laboratories, Inc., (Carlsbad, CA USA)) (buffer removed), mixed with 60 μL solution C1 (PowerSoil Kit, Mo Bio), and vortexed horizontally for 10 min according to the kit instructions. After centrifugation (10,000 *g*, 1 min), the supernatant was transferred to a new low-binding 2 mL reaction vial and mixed with 250 μL of solution C2 (vortex 5 s; incubation 5 min, 4 °C; centrifugation at 10,000 *g*, 1 min). The supernatant (ca.1 mL) was kept on ice in a 50 mL sterile conical tube.

#### 2.4.4. Further eDNA and iDNA Handling

iDNA and eDNA solutions were each mixed with a threefold amount of guanidine hydrochloride (GuaHCl) (6M GuaHCl in TE buffer, pH 6.7, 10 mM Tris HCl, 1 mM EDTA) and 15 μL (iDNA) or 18 μL (eDNA) of silica suspension (prepared according to [34] by inverting the tubes several times. The tubes were secured horizontally on an orbital shaker and shaken for 45 min and 175 rpm to bind the DNA to the silica particles. Finally, the silica was allowed to settle for 10 min on ice before the centrifugation step (4643 *g*, 10 min, RT, swing-out rotor). The supernatants were carefully removed by suction except for a residual of 1.5 mL to resuspend the silica pellets. The suspensions were transferred to new sterile 2 mL low-binding reaction vials and centrifuged (9000 *g*, RT, 3 min). The supernatants were discarded, and the pellets were each washed with 600 μL of washing buffer (55% EtOH, 70 mM NaCl, 10 mM Tris, 2.6 mM EDTA) (vortex 2 s, centrifuge at 9000 *g*, 1 min). The resulting supernatants were discarded, the pellets were again centrifuged (9000 *g*, RT, 3 min), and the remaining buffer was removed. The pellets were air-dried in a clean bench for 15 min. Finally, the DNA was eluted by resuspending the pellets in 100 μL (eDNA) or 80 μL (iDNA) of Tris buffer (1 mM Tris, pH 8.0, preheated to 50 °C) by pipetting up and down, then vortexed for 2 s, followed by an incubation period of 10 min in a thermal shaker (50 °C, 300 rpm). The silica suspensions were centrifuged (9000 *g*, RT, 2 min), and the supernatants were transferred to new 1.5 mL low-binding reaction vials. To remove any residual silica particles, the supernatants were centrifuged again (9000 *g*, RT, 5 min) and were transferred to new vials. DNA concentrations were mostly below the detection limit of fluorometric quantification methods.

#### 2.4.5. 16S rRNA Gene Amplicon Pool Preparation for Illumina Sequencing

PCR amplification targeted the hypervariable region V4 of the 16S rRNA (forward primer, 515F: 5′-GTGCCAGCMGCCGCGGTAA-3′; reverse primer, 806R: 5′-GGACTACHVGGGTWTCTAAT-3′, each of them specified with 6-bp tags). PCR amplification was performed in at least triplicate in 25 μL reactions (2.5 μL 10× PCR buffer, 0.5 μL ultrapure dNTP-mix (5 mM), 0.25 μL of each primer (10 mM), 1.5 μL MgCl2 (25 mM), 2–5 μL template, and 0.25 μL HotStar Taq polymerase (Qiagen, Hilden, Germany)) under the following conditions: initial denaturation at 95 °C for 15 min, followed by 10 cycles of 95 °C for 30 s, 65 °C −1 °C/cycle for 30 s, 72 °C for 45 s, 25 to 40 cycles (depending on DNA concentration of the different samples) of 95 °C for 30 s, 56 °C for 30 s, 72 °C for 45 s, and a final extension step of 10 min at 72 °C. The reactions were pooled, purified with Agencourt AMPure XP magnetic beads (Beckman Coulter Life Science, Krefeld Germany), and quantified with the Qubit Fluorometer (Invitrogen™, Thermo Fisher Scientific, USA). Purified PCR amplicons from all samples were pooled in equimolar ratios to a final concentration of approximately 120 ng/µL. Library preparation and sequencing of the amplicon pool with the Illumina MiSeq technology was carried out by Eurofins Genomics (Ebersberg, Germany). The raw data are available at the ENA EuropeanNucleotideArchive under the accession number PRJEB111675.

#### 2.4.6. Processing of 16S rRNA MiSeq Data and ASV Generation

Paired-end sequencing raw reads were demultiplexed and quality trimmed using Cutadapt v3.7 [35], discarding low-quality bases (-q 20) and short reads (-m 150). R v4.0.5 (R Core Team, 2022) and DADA2 v1.16 [36] were used to generate an ASV table with pooling approach and assign taxonomy, including forward and reverse read merging. Non-default parameters for the different functions were the following; filterAndTrim: truncLen 240/200, minLen 200, maxN 0, maxEE 2,2, truncQ 2, and rm.phix TRUE; dada: pool TRUE; and removeBimeraDenovo: method consensus. For taxonomic assignment, SILVA database v138.1 [37] was used. Replicates were averaged by (arithmetic) mean. Chloroplast and mitochondria ASVs were removed. Singletons were removed. ASVs that occur with more the 0.1% abundance in the blank control were removed. Rarefaction to 50,000 reads per sample was carried out using vegan v2.5-6 (https://cran.r-project.org/package=vegan, accessed on 18 October 2024) corrected for sequencing bias.

#### 2.4.7. Statistical Analysis, Visualization, and BLAST

For statistical analysis and visualization, the R package ggplot2 v3.2.1 [38] and VennDiagram v1.7.3 (https://CRAN.R-project.org/package=VennDiagram, accessed on 18 October 2024) were used; for ordination, phyloseq v1.28.0 [39] with the PCoA method and Bray–Curtis distance was used. Blastn from BLAST+ v.2.16.0 [40] and the complete NCBI nt database (downloaded on 18 October 2024) were used locally. To obtain the taxonomy from the BLAST results, the R package taxonomy v.10.6 (https://CRAN.R-project.org/package=taxonomizr, accessed on 18 October 2024) and NCBI taxonomy database (downloaded on 18 October 2024) were used.

## 3. Results

### 3.1. Geochemistry

**Figure 4:**
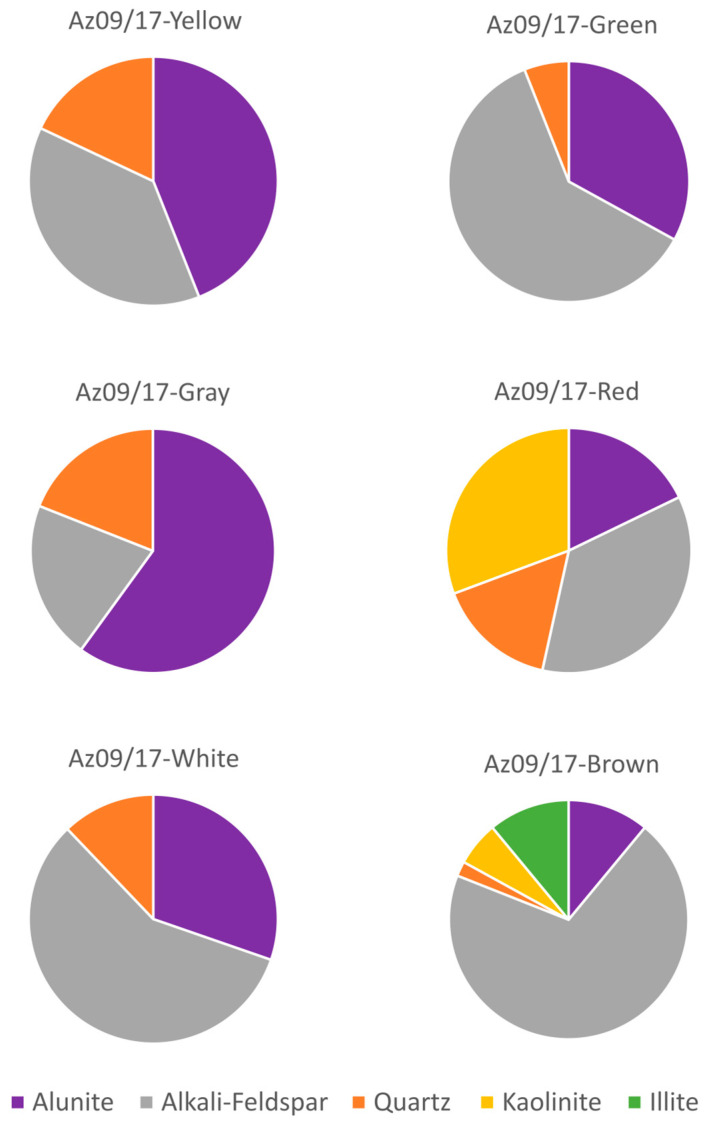
Mineral assemblages identified and semi-quantified by XRD in collected samples. Although the color varies, the mineral variation identified by XRD is small. All samples contain, besides quartz and alkali-feldspar (including albite, anorthoclase, orthoclase, and sanidine), alunite (KAl_3_(SO_4_)_2_(OH)_6_). Red and brown samples additionally contain detectable amounts of phyllosilicates (clay minerals, i.e., kaolinite and illite). Furthermore, an amorphic phase can be identified within the diffractogram as a broad, diffuse hump around 20° 2θ, which could be caused by volcanic glass or opal-A.

The six samples taken in the Furnas caldera were analyzed for their ionic composition by ICP-OES and IC and mineral composition by XRD. Ion analysis results revealed that sulfate is the dominating anion. The major cation was Al^3+^, with minor amounts of the other cations. Only the yellow sample has substantial amounts of Na^+^, K^+^, Ca^2+^, Fe^2+^, and Mg^2+^ in addition to Al^3+^ (Table 1). The mineral analysis showed that alunite, alkali-feldspar, and quartz made up the bulk of the mineral composition in all collected samples (Figure 4). The red sample also included kaolinite and the brown sample kaolinite and illite. The XRD results also indicated the presence of an amorphous phase, especially prominent in the white, yellow, and green samples. This amorphous phase contributed to a higher background signal, potentially masking minor mineral phases that may remain below the detection limit.

### 3.2. Phospholipid Fatty Acids (PLFAs)

**Figure 5:**
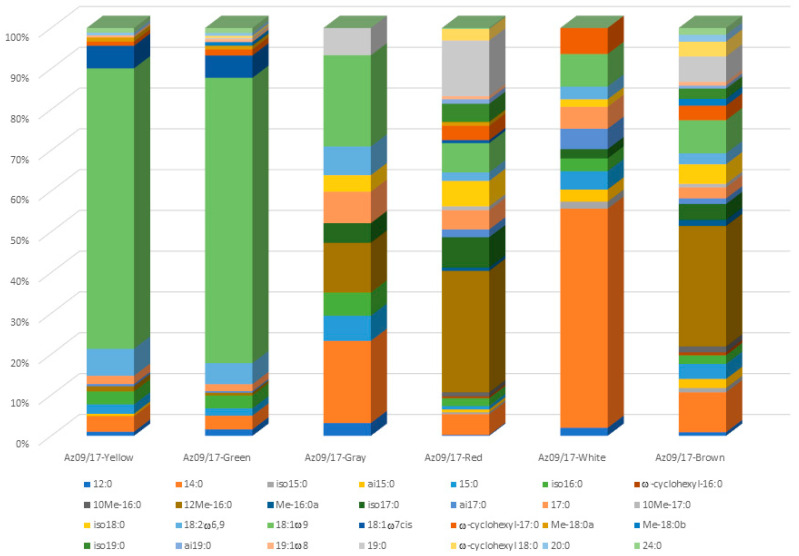
The fingerprint phospholipid fatty acid (PLFA) composition of the investigated Azores samples without the strongly dominating 16:0 (up to 70%) and 18:0 fatty acids (up to 37%). C:X = number of carbon atoms:number of double bonds; ω = double-bond position counted from the tail end; iso and ai = iso and anteiso FA; 10- and 12-Me = mid-chain branch at positions 10 and 12 counted from the functional group; w-cyclohexyl fatty acids = tail-end cyclohexyl group.

PLFA analysis only reflects the bacterial community, while Archaea do not contain any PLFAs. The PLFA distributions of the six samples were all very strongly dominated by the 16:0 (up to 70%) and partly by 18:0 (up to 37%) FAs (Supplement S1, Table S1). The strong dominance of these two very common FAs makes it difficult to recognize similarities in the overall PLFA compositions. Therefore, in Figure 5, we removed these two FAs from the PLFA distributions and compared only the remaining smaller (fingerprint) PLFAs. Generally, FAs range from C_12_ to C_18_ in the white sample, from C_12_ to C_19_ in the gray sample, and from C_12_ to C_24_ in the green, yellow, red, and brown samples. In addition to the *n*-FAs, *iso* and *anteiso* FAs with 15, 16 (only iso), 17, and 19 carbon atoms, mid-chain branched (10- and 12Me) FAs with 16 and 17 (only 10Me) carbon atoms, a series of unsaturated FAs with 18 (18:1w7cis, 18:1w9cis, 18:2w6,9) and 19 (19:1w8cis), and some very unusual w-cyclohexyl-FAs with 16, 17, and 18 carbon atoms were found in small amounts in the sample series (Figure 5, Supplemental Table S1).

Comparing the PLFA composition of the investigated samples similarities can be observed between the yellow and green as well as the red and brown samples. The white sample shows another quite unique pattern, and the gray sample shares some similarities with all samples. The green and yellow samples show the highest level of similarity, and both are strongly dominated by 18:1w9cis and to a smaller degree by 18:1w7cis and 18:2w6,9cis. The red and brown samples also show a very similar PLFA distribution with 12Me-16:0 as the dominant FA followed by 19:0, 18:1w9cis, and 14:0. The white and gray samples both show high amounts of 14:0; however, the gray samples show also some characteristics of the red and brown samples (abundant 19:0 and 12Me-16:0) and the green and yellow samples (18:1w9cis). These results suggest similarities in the associated microbial communities at least of the green and yellow as well as red and brown samples. The sampling locations of these sample pairs are also quite close to each other (Figure 3), while the sample positions of the gray and white samples are between these two groups.

### 3.3. Microbial Community Structure Based on 16S rRNA

To get a complete overview of the prokaryotic microbial communities, we performed a specialized DNA extraction method that separates intracellular DNA (iDNA) of intact cells and extracellular DNA (eDNA). This approach allows us to distinguish between relic or dead and active microbial communities and to be sure that we are handling active microbial communities for all samples. The DNA was subjected to 16S rRNA amplification and sequencing followed by bioinformatic processing, including the generation of amplicon sequence variants (ASVs) and further analyses. In total, 8.03 million raw reads and 6.64 million processed reads were obtained, with an average of 201,107 reads per sample.

**Figure 6:**
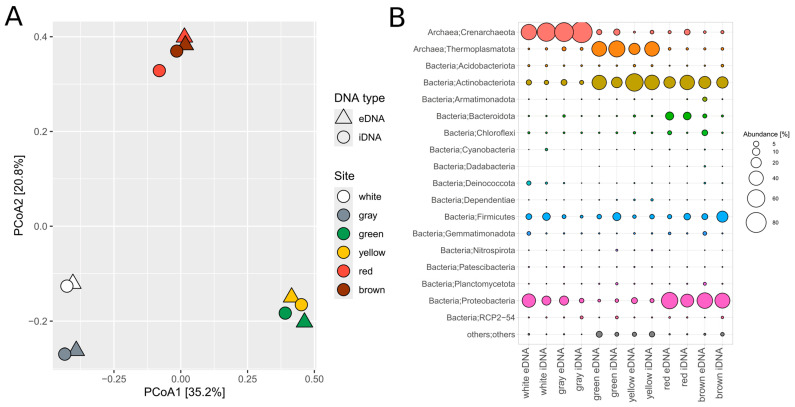
Microbial community differences based on 16S rRNA. (**A**) Beta-diversity analysis using the PCoA method and the Bray–Curtis dissimilarity. The microbial communities show 3 distinct clusters of similar communities. iDNA and eDNA are similar. (**B**) Bubble plot for ASVs summarized by phylum showing iDNA and eDNA. The cluster of white/gray is dominated by Crenarchaeota, while the green/yellow cluster shows the highest abundance of Thermoplasmatota and Actinobacteriota. The cluster of red and brown samples show high levels of Proteobacteria and Actinobacteriota.

The results show three distinct clusters of samples with similar microbial communities (Figure 6A). The clusters are red/brown, yellow/green, and white/gray (see also Figure 3). The different DNA extraction methods show only minor differences of the microbial community composition. The same trends are reflected in the relative abundance of detected taxa shown in Figure 6B. The most abundant phyla are Crenarchaeota, Thermoplasmatota, Actinobacteriota, Firmicutes, and Proteobacteria. The Archaea are highly variable between the clusters, with Crenarchaeota dominant in white/gray samples and Thermoplasmatota dominant in yellow/green sample, while red/brown show only minor fractions of both. Actinobacteriota are dominant in yellow/green and red/brown samples. Interestingly, the yellow/green cluster shows ASVs that could not be taxonomically assigned.

The microbial community composition is highly dominated by single ASVs potentially representing single species. For example, the white and gray samples contain a single ASV of the genus *Stygiolobus*, with an abundance ranging from 28% to 79% of total relative abundance (Supplement S2, Table S2). The yellow and green samples are dominated by a few ASVs from the families *Thermoplasmataceae* and *Mycobacteriaceae*, forming up to 40% of the abundance of these samples, including single top ASVs with 14% to 47% relative abundance. Also, red and brown samples are dominated by single ASVs with different taxonomic assignments, e.g., from the phylum RCP2-54, order Group 1.1c and families *Bacillaceae*, *Moraxellaceae*, *Micrococcaceae*, *Sulfolobaceae*, and *Thermicanaceae*.

To get more insights into the most abundant potential species, we blasted the top 20 ASVs. The results are shown in Supplement S3, Table S3. Few of the ASVs show 100% identity to known species, e.g., ASV4—*Mycobacterium botniense*, ASV11—*Pseudomonas gessardii*, or ASV12—*Ralstonia pickettii*. Many of the top 20 ASVs have best hits to unknown species, some with even 100% identity. Other ASVs have best hits in the entire NCBI database with only 97% identity.

The different DNA extraction techniques reveal very similar results, with eDNA and iDNA mainly showing very similar abundances, (see top20 phyla bubble plot, Figure 6B, Supplemental Table S2). There are only single ASVs that show a larger variation between eDNA and iDNA, e.g., ASV11 for white and gray samples or ASV23 for the brown samples representing specific microhabitats.

## 4. Discussion

This study investigated small-scale mineralogical and microbial heterogeneities within the Furnas fumarolic hydrothermal system on San Miguel, Azores. By combining geochemical, mineralogical, and microbiological analyses across visually distinct patches, we assessed how centimeter-scale environmental variability shapes microbial community composition. Our results reveal pronounced spatial heterogeneity, highlighting that even closely adjacent locations can host distinct microbial assemblages linked to local mineralogical and geochemical conditions.

At the field site, we collected geochemical data and also analyzed collected samples for PLFAs and DNA. The mineral results align well with the local geological context of a hydrothermal system influenced by volcanic gases and fluids, which alter the caldera host rock, i.e., a trachyte bedrock, composed mainly of alkali feldspar [41]. Water–rock interactions at temperatures > 200 °C, and H_2_S and S oxidation can lead to the formation of the detected sulfates and clays [42]. The amorphic hump visible in the XRD diffractograms is likely attributed to opal-A, which is a known hydrothermal alteration product in the Furnas fumaroles forming sinter [11]. This amorphous phase may also mask minor mineral fractions, potentially explaining why certain suspected secondary phases were not definitively identified in the diffractograms. The quartz, however, is unlikely to be a direct product of the fumarole alteration due to its acidic conditions. It may have crystallized in deeper hydrothermal fluids and transported later to the surface, or older amorphous sinter deposits have recrystallized into quartz [43]. In the water-soluble phase, aluminum and sulfate were identified as the major cation and anion, respectively, consistent with the surrounding water bodies (Furnas Lake and adjacent groundwater [13]). The observed color variations could be partially due to the complex mineral composition of acid-sulfate rocks, which are found in fumaroles [11,44,45,46]. Driving factors for the mineral composition can be numerous, including temperature, pH, gas, fluid and rock composition, fluid-rock ratios, and microbial processes. The green color could be caused by the salt melanterite (FeSO_4_ ∙ 7 H_2_O), which has been reported in the hydrothermal system of the Furnas volcano [11] and precipitates under high sulfate concentrations and extremely low pH [47]. Although we did not detect melanterite by XRD, the ion composition could indicate its presence as it is water soluble, but only in trace amounts. The reddish coloration may also be attributed to clay minerals such as illite and kaolinite, in which Fe^3+^ can substitute for Al^3+^ in the crystal structure, resulting in a reddish-brownish appearance. Additionally, the common acid-sulfate mineral jarosite also presents yellowish to red color, which precipitates at low pH (<3) after Fe^2+^ oxidation. However, jarosite was not detected in our samples. Overall, the color differences within the investigated small-scale area reflect a highly heterogenous environment. Fractures supplying hydrothermal waters create localized gradients in pH, temperature, and acidity, significantly influencing mineral deposition patterns within the fumarole system and its adjacent environment.

The PLFA fingerprint patterns, after neglecting the very dominant C_16:0_ and C_18:0_ fatty acids, reveal strong similarities between the microhabitats represented by the yellow and green as well as the red and brown colors (Figure 5) but with significant differences between these pairs. This suggests a similar microbial community within but different communities between these microhabitat pairs, which is confirmed by the DNA analysis result showing two distinct clusters for these pairs (Figure 6A). The PLFA pattern of the white microhabitat is significantly different from the other two sample pairs, which also is in line with the beta-diversity results (Figure 6A), showing a distinct offset of the DNA composition of the white microhabitat from those of the other two pairs. The PLFA inventory of the gray-colored microhabitat shows characteristics of all other microhabitats having abundant amounts of the 14:0 (white microhabitat), 18:1ω9 (green/yellow microhabitats), and 12Me-16:0 (red/brown microhabitat) fatty acids (FAs). This could indicate that the gray microhabitat represents a mixture of microorganisms from the other locations. However, the DNA results indicate that the microbial community in the gray microhabitat has a higher similarity to that of the white one, although the gray microbial community does not plot as close to the white microhabitat as the green and yellow, and red and brown microhabitats do to each other. One reason for the closer proximity of the white and gray microhabitats could be that the microbial composition of these samples is dominated by *Chrenarchaeota*, which are not resembled by the PLFA method, as the cell membranes of archaea do not contain any phospholipids.

More uncommon PLFAs are the ω-cyclohexyl-FAs with 16, 17, and 18 carbon atoms, whereas the C_16_ congener was only found in the red and brown microhabitats, C_17_ in the white, green, yellow, red, and brown, and C_18_ in the green, red, and brown microhabitats. The gray sample does not contain any of these fatty acids. Oshima and Ariga [48] reported these biomarkers in acido-thermophilic bacteria from Japanese hot springs, which could match the investigated habitat. Methylated C_17_ and C_19_ and at least C_19_ w-cyclohexyl FAs have also been found in *Rubrobacter* species [49,50]. Another biomarker for *Rubrobacter* is the 12Me-16:0 FA, which is also found in the green and yellow but particularly in the red and brown as well as in the white microhabitats. The DNA data show that *Rubrobacter* is present (~6%) at least in the red microhabitats (see Supplement Tables). 18:1w9 partly accompanied by 18:1w7 is present in all samples but most abundantly in the green and yellow ones. 18:1w9 is among others found in cyanobacteria and could be an indicator of these species in the investigated samples [51]. However, the DNA data only indicate small amounts of cyanobacteria.

The used 16S rRNA method uses universal primers that are designed to detect a wide range of bacterial and archaeal taxa [52]. While both PFLA and 16S rRNA analyses revealed consistent clustering patterns among the different colored spots, the 16S rRNA data further showed that most communities were dominated by archaeal ASVs, indicating that archaea represent a major component of the active microbial communities in this hydrothermal system.

The similarity between eDNA and iDNA fractions within individual microhabitats shows an active microbial turnover and indicates that the detected communities largely represent living and potentially metabolically active populations rather than relic DNA [53,54]. Despite the extremely small spatial scale of sampling (≤30 cm; Figure 3), microbial communities segregate into clearly distinct compositional clusters, demonstrating a pronounced spatial structuring at the centimeter scale. The dominance of extremophilic taxa across all microhabitats reflects the harsh environmental conditions characterized by high ion concentrations, low pH, and elevated temperatures [55]. However, the strong differentiation between communities suggests that subtle local gradients, rather than bulk environmental conditions, are the primary drivers of microbial community assembly.

ASV1 and ASV5 were identified as members of the genus *Stygiolobus*. Interestingly, the genus *Stygiolobus* was defined in 1991 by describing *Stygiolobus azoricus* found on the Azores [56]. ASV5 is potentially identified as *Stygiolobus caldivivus* [57]. Both organisms are hyperthermophile and can grow chemolithoautotrophic with S^0^ and *S. caldivivus* also with ferric citrate and FeCl_3_. Both ASVs predominantly occur in white and gray microhabitats, where they reach very high relative abundances (up to 70%). This strong dominance indicates that these microhabitats represent highly selective ecological niches favoring sulfur-based chemolithoautotrophy. The occurrence of these taxa in environments with low detectable Fe further suggests that sulfur metabolism, rather than iron cycling, may be the dominant energy pathway in these niches.

An intriguing observation is the correspondence between microbial community composition and sample coloration. ASV4, which we identified as *Mycobacterium botniense* by BLAST results, is very abundant in the yellow microhabitat (10–47%) and was shown to produce yellow pigments [58]. In addition, this organism is known to grow up to a temperature of 50 °C and has a high salt tolerance. Also, other archaea are known to produce pigments [59]. Given that the bulk ionic composition is relatively similar across different microhabitats, these findings raise the possibility that microbial pigmentation contributes significantly to the observed color differences. For building material, it was shown that microorganisms, mainly cyanobacteria, algae, and fungi, are causing discoloration [60]. However, for natural rock material microorganisms have not been considered as a main cause of coloring previously.

Our results suggest that microbial communities in hydrothermal systems are not merely passive responders to bulk geochemical conditions but are structured by highly localized environmental gradients that operate at the centimeter scale. The strong segregation of distinct, functionally specialized taxa indicates that these microhabitats represent discrete ecological niches, likely defined by subtle variations in fluid flow, redox conditions, and energy availability. Consequently, visually distinct mineral patches may serve as proxies for underlying geochemical microenvironments and their associated microbial metabolisms. This highlights the importance of microscale heterogeneity as a fundamental organizing principle of microbial life in hydrothermal systems and suggests that such visually expressed heterogeneity may provide a useful guide for targeted sampling in the search for biosignatures in hydrothermal environments.

## 5. Conclusions

Despite largely similar bulk mineralogy across all collected samples within a range of 30 cm, microbial communities segregate into three distinct clusters that correspond closely to visually defined microhabitats at the centimeter scale. This decoupling of mineralogical similarity and biological diversity suggests that subtle, localized geochemical gradients, rather than bulk composition, act as primary drivers of microbial community assembly. The strong dominance of specific archaeal and bacterial taxa within each cluster further indicates that these microenvironments represent highly selective ecological niches. Together, our results demonstrate that hydrothermal systems are structured as mosaics of functionally distinct microbial (micro)habitats, even at the sub-decimeter level.

These findings also have important implications for the search for life on Mars and other planetary bodies, as they suggest that potential biosignatures and habitable niches may occur at very small spatial scales that are not resolved by bulk sampling approaches. Visually distinct mineral features, such as color variations, may therefore provide valuable targets for identifying localized microbial activity and guiding life-detection strategies in hydrothermal environments beyond Earth.

## Figures and Tables

**Table 1:** Ionic composition of samples analyzed ^1^.

Sample Name	Na^+^	K^+^	Ca^2+^	Fe^2+^	Mg^2+^	NH_4_^+^	Al^3+^
AZ09/17-Yellow	208.2	78.7	28.6	288.4	21.7	2.4	2713
AZ09/17-Green	36.7	1.7	0.3	23.5	2.8	1.0	489.3
AZ09/17-Gray	14.8	ND	0.1	0.2	1.7	1.4	179.2
AZ09/17-Red	15.4	0.6	0.2	ND	3.2	0.8	239.0
AZ09/17-White	20.4	ND	0.3	0.3	3.2	1.3	436.2
AZ09/17-Brown	3.9	0.2	0.2	ND	1.6	0.5	87.0
All values in mg/kg							
**Sample Name**	**SO_4_^2−^**		**F^−^**		**Cl^−^**	**NO_3_^−^**	
AZ09/17-Yellow	6369		ND		ND	ND	
AZ09/17-Green	1549		ND		0.4	2.0	
AZ09/17-Gray	150.8		ND		0.4	0.7	
AZ09/17-Red	718.8		0.9		1.5	0.2	
AZ09/17-White	1259		0.4		1.1	0.4	
AZ09/17-Brown	225.8		1.1		0.5	0.1	
All values in mg/kg							

^1^ Note: Samples were also analyzed for lithium, nitrite, bromide, and phosphate, but the concentrations of these ions were in all samples below the detection limit (ND; which was at 0.1 mg/kg).

## Data Availability

The raw sequencing data from this study are available at the ENA European Nucleotide Archive under the accession number PRJEB111675.

## Supplementary Materials

The following supporting information can be downloaded at: https://www.mdpi.com/article/10.3390/life16071086/s1, Supplement S1-Table S1: Phospholipid fatty acids (PLFA) distribution of the investigated Azores samples; Supplement S2-Table S2: Average (mean) ASV read counts from rarefied (n = 50,000) samples; Supplement S3-Table S3: Taxonomy based on NCBI database; Supplementary Figure S1: Slide2.

## Abbreviations

The following abbreviations are used in this manuscript:

16S rRNA	16S Ribosomal Ribonucleic Acid
ASV	Amplicon Sequence Variant
BLAST	Basic Local Alignment Search Tool
eDNA	Extracellular Deoxyribonucleic Acid (DNA)
IC	Ion Chromatography
ICP-OES	Inductively Coupled Plasma Optical Emission Spectrometry
iDNA	Intracellular Deoxyribonucleic Acid (DNA)
FA	Fatty Acid
NCBI	National Center for Biotechnology Information
PCR	Polymerase Chain Reaction
PLFA	Phospholipid Fatty Acid
XRD	X-ray Diffractometry
