# Supplementary material for: Small-Scale Mineral and Microbial Heterogeneities near a Fumarole at the Furnas Hydrothermal Zone on the Azores
Source: Life (Basel). 2026 Jun 28;16(7):1086. doi: 10.3390/life16071086 (PMC13412118; doi:10.3390/life16071086)
Supplement: Supplementary file 1 [file life-16-01086-s001.zip › Supplement 1 - Table S1_revKM_DSM.pdf]

PLFA	Az09/ 17-White	Az09/ 17-Grey	Az09/ 17-Green	Az09/ 17-Yellow	Az09/ 17-Red	Az09/ 17-Brown
12:0	0.08	0.65	0.37	0.25	0.04	0.34
14:0	2.11	4.12	0.78	0.95	0.98	3.68
*iso*15:0	0.07				0.10	0.40
*ai*15:0	0.12			0.14	0.13	0.83
15:0	0.18	1.27	0.42	0.58	0.16	1.40
*iso*16:0	0.12	1.18	0.74	0.80	0.38	0.80
16:0	58.33	60.47	70.30	67.79	46.10	48.73
ω-cy-hexyl-16:0					0.08	0.27
10Me-16:0					0.20	0.53
12Me-16:0		2.49	0.15	0.31	5.72	11.15
Me-16:0a					0.16	0.55
*iso*17:0	0.09	0.99			1.43	1.48
*ai*17:0	0.20		0.09	0.14	0.37	0.53
17:0	0.21	1.58	0.39	0.51	0.90	1.01
10Me-17:0					0.18	0.33
*iso*18:0	0.07	0.83			1.20	1.81
18:2w6,9	0.12	1.45	1.20	1.65	0.41	1.02
18:1w9	0.31	4.59	16.23	17.16	1.38	3.07
18:1w7cis			1.27	1.39	0.14	
ω-cy-hexyl-17:0	0.25		0.34	0.25	0.67	1.35
18:0	37.74	19.01	6.49	7.25	34.64	13.52
Me-18:0a			0.21	0.25	0.18	
Me-18:0b			0.21			0.63
*iso*19:0					0.86	0.95
*ai*19:0					0.21	0.28
19:1w8			0.16	0.13	0.14	0.34
19:0		1.37	0.08		2.63	2.36
ω-cy-hexyl18:0			0.12		0.54	1.37
20:0			0.16	0.17		0.64
24:0			0.28	0.29	0.05	0.63

Table S1: Phospholipid fatty acids (PLFA) distribution of the investigated Azores samples. C:X = number of carbon atoms:number of double bonds, ω = double bond position counted from the tail end, *iso* and *ai* = *iso* and *anteiso* FA, 10- and 12-Me = mid chain branch at position 10 and 12 counted from the functional group, ω-cyclohexyl fatty acids = tail end cyclohexyl ring, a and b = unknown methyl-group position;
