# Supplementary material for: Small-Scale Mineral and Microbial Heterogeneities near a Fumarole at the Furnas Hydrothermal Zone on the Azores
Source: Life (Basel). 2026 Jun 28;16(7):1086. doi: 10.3390/life16071086 (PMC13412118; doi:10.3390/life16071086)
Supplement: Supplementary file 1 [file life-16-01086-s001.zip › Supplement 2 - Table S2.txtconv2pdf.pdf]

Average (mean) ASV read counts from rarefied (n=50000) samples.

	white eDNA	white iDNA	grey eDNA	grey iDNA	green eDNA	green iDNA	yellow eDNA	yellow iDNA	red eDNA	red iDNA	brown eDNA	brown iDNA	Kingdom	Phylum	Class	Order	Family	Genus
ASV_1	17623	27343	17535.67	25650.33	203.33	362.33	107	683.33	11	1656	269.33	391	Archaea	Crenarchaeo	Thermoprote	Sulfolobales	Sulfolobacea	Stygiolobus
ASV_3	644	2258	13663	14756.67	26.67	66	59	123.33	0	322	103	129.33	Archaea	Crenarchaeo	Thermoprote	Sulfolobales	Sulfolobacea	NA
ASV_4	97	79.67	105.33	39.67	11343	2857	21017	10867	205	119	208.67	650.67	Bacteria	Actinobacter	Actinobacter	Corynebacte	Mycobacteria	Mycobacterium
ASV_5	3591.67	3069	3401.67	3199	311.33	590	25	127.33	104	181	46.67	72.33	Archaea	Crenarchaeo	Thermoprote	Sulfolobales	Sulfolobacea	Stygiolobus
ASV_7	36.67	59.33	59	605.33	13.67	313.33	11.5	10.33	127	72	10.33	30.67	Bacteria	RCP2-54	NA	NA	NA	NA
ASV_8	110.33	69.33	9.33	41	5.67	47.33	0.5	11.67	400	25	5.67	23.67	Archaea	Crenarchaeo	Nitrososphae	Group 1.1c	NA	NA
ASV_9	28	85.67	13.67	11.67	4438	8237	2374	4462	114	43	60	108.67	Archaea	Thermoplasn	Thermoplasn	Thermoplasn	Thermoplasn	A-plasma
ASV_10	36.33	74	11.67	8.67	2118	2323.67	5528	9714	56	67	54	99.33	Archaea	Thermoplasn	Thermoplasn	Thermoplasn	Thermoplasn	A-plasma
ASV_11	6080	235.67	3916.67	498.67	36	16.67	598.5	63	37	87	55.67	53.67	Bacteria	Proteobacter	Gammaprote	Pseudomona	Pseudomona	Pseudomonas
ASV_12	3023.67	3827.67	234.67	80	46.67	40.33	276	166.67	1868	7118	892.33	4217.33	Bacteria	Proteobacter	Gammaprote	Burkholderia	Burkholderia	Ralstonia
ASV_13	29.33	58.33	1213	549.67	2434	3814	142.5	372.33	58	71	53.67	92	Archaea	Thermoplasn	Thermoplasn	Thermoplasn	Thermoplasn	A-plasma
ASV_15	31	55	6	5.33	2436.67	3679.67	1790	1922.67	57	24	35.33	64	Archaea	Thermoplasn	Thermoplasn	Thermoplasn	Thermogymn	NA
ASV_16	42.33	30.67	17	11.67	1225.33	675	5224.5	6901	24	58	62	310.67	Bacteria	Actinobacter	Actinobacter	Corynebacte	Mycobacteria	Mycobacterium
ASV_17	982.33	4522.67	435.33	96	98.33	31	147	48.67	21	80	442.67	655.67	Bacteria	Firmicutes	Bacilli	Staphylococc	Staphylococc	Staphylococcus
ASV_18	9.67	13.67	37	9.33	6610.67	3014.67	52.5	495	118	29	58.33	33.33	Archaea	Thermoplasn	Thermoplasn	BSLdp215	NA	NA
ASV_19	26.67	25	1407.67	127	5734.67	1561.67	1128	446	64	82	100.33	112	Bacteria	Actinobacter	Actinobacter	Corynebacte	Mycobacteria	Mycobacterium
ASV_20	14.33	42	25	4.67	1684	2747	1031.5	1943.33	40	21	24	48.67	Archaea	Thermoplasn	Thermoplasn	Thermoplasn	Thermoplasn	A-plasma
ASV_21	12	43.33	20.33	46.33	100.67	103.33	3	6.67	32	7	6.67	31	Archaea	Crenarchaeo	Nitrososphae	Group 1.1c	NA	NA
ASV_22	140.67	19.67	12.67	3.67	2640	1053	987	1924	72	29	57.33	77.33	Bacteria	NA	NA	NA	NA	NA
ASV_23	68.33	3	35.67	1.33	89.67	36	442.5	82.67	23	166	12833.33	189.33	Bacteria	Proteobacter	Gammaprote	Enterobacter	Enterobacter	Klebsiella
ASV_24	393.67	54.67	94	30.33	1306.67	1822	9	59	55	12	23.33	37	Archaea	Crenarchaeo	Thermoprote	Sulfolobales	Sulfolobacea	Acidianus
ASV_25	1119.67	117.33	80	10	4.67	4.33	76.5	43.33	4	7	217.33	3378.67	Bacteria	Proteobacter	Gammaprote	Burkholderia	Comamonad	NA
ASV_26	16.33	343.33	11.67	9.67	472.33	2651	233	441.33	69	16	21	31	Bacteria	Actinobacter	Acidimicrobii	Acidimicrobia	Acidimicrobia	Ferrimicrobium
ASV_27	17	16.67	54.67	26.67	1.67	11	0	7.33	13	20	0.67	0.67	Archaea	Crenarchaeo	Thermoprote	Sulfolobales	Sulfolobacea	Stygiolobus
ASV_28	12	10.67	47.33	7.67	28.67	18.33	83.5	86.67	521	145	887.67	4372.33	Bacteria	Proteobacter	Gammaprote	Pseudomona	Moraxellacea	Enhydrobacter
ASV_29	22.67	12.33	44.67	3	1.67	4.67	97	22	1	2	21.67	3076.67	Bacteria	Actinobacter	Actinobacter	Micrococcale	Micrococcace	Micrococcus
ASV_33	1291.67	5.33	1147.33	41.67	17.67	2.33	163.5	81	4	29	13	11.67	Bacteria	Proteobacter	Gammaprote	Pseudomona	Pseudomona	Pseudomonas
ASV_34	9.33	8.67	4.33	0.33	10.33	2	15.5	15.33	509	8	122.33	3349.67	Bacteria	Firmicutes	Bacilli	Lactobacillale	Streptococca	Streptococcus
ASV_35	1333	229	90.67	1	5.67	5	39	20.33	2	3	145	42.67	Bacteria	Deinococcota	Deinococci	Thermales	Thermaceae	Meiothermus
ASV_36	18.33	6	22.33	0.33	0	4.67	78.5	15.33	0	0	18.33	2252.67	Bacteria	Proteobacter	Gammaprote	Burkholderia	Comamonad	NA
ASV_39	8.67	9	2	6.33	121.33	630.67	136	464.67	21	12	6.33	10	Bacteria	Actinobacter	Acidimicrobii	NA	NA	NA
ASV_40	711.67	747.33	1	10.67	12	0	2	0	0	0	0	0.33	Bacteria	Proteobacter	Gammaprote	Enterobacter	Idiomarinace	Idiomarina
ASV_41	10.67	6.33	12.67	0	0.67	0.67	59.5	10.33	0	0	3.67	1614.67	Bacteria	Firmicutes	Bacilli	Staphylococc	Staphylococc	Nosocomiicoccus
ASV_42	5	4.67	16.67	34	136.67	977.67	16.5	11.67	37	0	9	10	Bacteria	Firmicutes	Sulfobacillia	Sulfobacillale	Sulfobacillace	Sulfobacillus
ASV_43	14	7.33	13.33	1.67	0	1.33	54.5	18.33	0	0	14	1516	Bacteria	Firmicutes	Bacilli	Caldalkalibac	Caldalkalibac	Caldalkalibacillus
ASV_44	12.67	26.67	80.33	3.67	10.67	7.33	63	12	1373	34	423	165	Bacteria	Bacteroidota	Bacteroidia	Flavobacteria	Weeksellacea	Cloacibacterium
ASV_45	5.33	7	38.67	12.67	121.67	936.33	17.5	2.67	42	3	4	7	Bacteria	Firmicutes	Sulfobacillia	Sulfobacillale	Sulfobacillace	Sulfobacillus
ASV_46	530	174	546	55.33	8	9.67	120.5	10.33	7	22	14.67	21.33	Bacteria	Proteobacter	Alphaproteol	Rhizobiales	Beijerinckiace	Methylobacterium-Methylorubrum
ASV_47	1	7.67	2.33	2.33	587.67	914.67	13	10.67	16	2	6	14.67	Archaea	Thermoplasn	Thermoplasn	Thermoplasn	Thermoplasn	NA
ASV_48	9	111.67	15.67	24	11.67	15.67	49	78.67	31	31	400	782	Bacteria	Proteobacter	Gammaprote	Pseudomona	Halomonada	Halomonas
ASV_49	7.67	9.33	0	4	7.67	4.67	0	0	0	0	0.33	1.33	Bacteria	Chloroflexi	Ktedonobact	Ktedonobact	Ktedonobact	NA
ASV_50	4.67	0.33	0.33	0	7.67	0.67	0.5	15.33	2	40	1982.67	35.67	Bacteria	Actinobacter	Actinobacter	0319-7L14	NA	NA
ASV_51	3.33	146	6.67	0	8.33	11.67	14.5	8	6639	3	4.33	377	Bacteria	Proteobacter	Gammaprote	Pseudomona	Halomonada	Halomonas
ASV_52	1.67	1.67	1	1	275	326.67	288	673	8	4	5.33	8.67	Archaea	Thermoplasn	Thermoplasn	Thermoplasn	Thermoplasn	Cuniculiplasma
ASV_53	1.67	3	2.33	1.67	467.67	152.67	406	494.67	16	5	10.67	7.67	Bacteria	NA	NA	NA	NA	NA
ASV_54	1	4.67	2.67	1	255	325.33	279	622	5	4	5.33	8	Archaea	Thermoplasn	Thermoplasn	Thermoplasn	Ferroplasma	Acidiplasma
ASV_55	0.33	5.67	0	0	1	0	0.5	0	3	0	2	13	Bacteria	Proteobacter	Alphaproteol	Rhizobiales	NA	NA
ASV_56	5.67	0.33	1	1	6	0	2	20	2	34	1811	39.67	Bacteria	Actinobacter	Actinobacter	Euzebyales	Euzebyaceae	NA
ASV_57	1092	4.67	5	0	0.33	3	26.5	5	0	0	12.67	11.33	Bacteria	Firmicutes	Bacilli	Staphylococc	Staphylococc	Staphylococcus
ASV_59	1.33	0	0	0	7	0	1	13	0	29	1517.33	21	Bacteria	Armatimonac	NA	NA	NA	NA
ASV_61	1.67	3.67	0.33	0	448	313.67	29.5	60.67	10	3	6	4.67	Archaea	Thermoplasn	Thermoplasn	Thermoplasn	Thermoplasn	Thermoplasma
ASV_63	2	3.67	0.33	23	1.33	27	1.5	0.33	2	8	0.67	52.67	Bacteria	Proteobacter	Alphaproteol	Acetobactera	Acetobactera	Acidicaldus
ASV_64	4.33	3.67	109	127.33	64.33	287.67	35	30.67	4	5	5.33	5	Bacteria	Actinobacter	Acidimicrobii	Acidimicrobia	Acidimicrobia	Aciditerrimonas
ASV_65	3	7	0	7	0	6.33	0	0	3	0	0	2.67	Bacteria	RCP2-54	NA	NA	NA	NA
ASV_66	4	17.33	8.67	0.33	9.67	0.33	0.5	87	9	68	1025.33	16.67	Bacteria	Firmicutes	Bacilli	Staphylococc	Staphylococc	Staphylococcus
ASV_67	0	5	0	0	0.67	0	0	1.33	2	0	2.33	17	Bacteria	Actinobacter	Thermoleoph	Gaiellales	NA	NA
ASV_68	4.33	7	33.33	20	279	421.67	1.5	17.33	17	7	7	12.33	Archaea	Crenarchaeo	Thermoprote	Sulfolobales	Sulfolobacea	Stygiolobus
ASV_69	0	0.33	0	0	15.33	14.33	2	0	8316	0	3.33	0	Bacteria	Proteobacter	Gammaprote	Pseudomona	Pseudomona	Pseudomonas
ASV_70	4.33	0.33	4	0	8.33	5	48	1.67	0	25	24.67	1455.67	Bacteria	Proteobacter	Gammaprote	Burkholderia	Comamonad	NA
ASV_71	9.33	0.33	7.67	0	14.33	9.67	102	1.33	2	0	2132	27.67	Bacteria	Chloroflexi	Chloroflexia	Kallotenuales	AKIW781	NA
ASV_72	2.33	0	0	0	4.33	0	0.5	10	0	24	1178	17.33	Bacteria	Proteobacter	Alphaproteol	Rhizobiales	Beijerinckiace	Microvirga
ASV_73	2.33	4	1	18.67	0.67	7.33	0	0	5	0	0.33	6.33	Bacteria	Chloroflexi	Ktedonobact	Ktedonobact	Ktedonobact	NA
ASV_74	4.33	0	0	0	3.33	0	0	9.33	0	27	1193.33	17.33	Bacteria	Actinobacter	NA	NA	NA	NA
ASV_75	2	2.67	0.67	1.33	111.33	786.67	10.5	5	29	0	3.67	4	Bacteria	Firmicutes	Sulfobacillia	Sulfobacillale	Sulfobacillace	Sulfobacillus
ASV_76	1.33	4.33	1.33	19	0.33	44	2	1	1	0	2.33	2.33	Bacteria	RCP2-54	NA	NA	NA	NA
ASV_77	6.33	0.33	2	0	7.67	3.33	30	5.33	0	11	1445.67	19	Bacteria	Proteobacter	Gammaprote	Enterobacter	Enterobacter	Buttiauxella
ASV_79	2.33	2.33	11	7	2.67	1.67	0	1.33	11	2	0.33	1.67	Archaea	Crenarchaeo	Nitrososphae	SCGC AB-179	NA	NA
ASV_80	422.33	47	1.33	98	2.33	1	2	11.67	0	0	1	1.33	Bacteria	Proteobacter	Gammaprote	Burkholderia	Comamonad	Acidovorax
ASV_81	5	4.33	6.67	0	0	1.33	23.5	4	0	105	3.67	723	Bacteria	Firmicutes	Bacilli	Bacillales	Bacillaceae	NA
ASV_82	78.67	34.33	79.33	80.33	99	59.33	159	27.33	21	57	90.67	15.67	Bacteria	Actinobacter	Thermoleoph	Solirubrobact	67-14	NA
ASV_83	4	238	1.33	4.67	0	0	0	17.67	0	29	0	1.67	Bacteria	Proteobacter	Gammaprote	Pseudomona	Moraxellacea	Acinetobacter
ASV_84	0.67	4	0	0	1.67	0.67	0	0	2	0	1.67	8	Bacteria	Actinobacter	Actinobacter	NA	NA	NA
ASV_85	1	2.33	0.33	1.33	0	3	0	1.33	8	0	0	1.67	Bacteria	Chloroflexi	Ktedonobact	Ktedonobact	Ktedonobact	Thermogemmatispora
ASV_86	235.33	2.33	16.67	1.67	15	1	8.5	24.33	14	334	381.33	5.33	Bacteria	Actinobacter	Actinobacter	Pseudonocar	Pseudonocar	Amycolatopsis
ASV_87	1.67	2.67	3	372	0	0.67	1.5	4.67	0	3	0.33	1.67	Bacteria	Proteobacter	Gammaprote	Pseudomona	Moraxellacea	Acinetobacter
ASV_88	5	0.67	2.67	0	10	0.33	1	42.33	14	46	842.67	8	Bacteria	Actinobacter	Actinobacter	Nitriliruptora	Nitriliruptora	NA
ASV_89	514	2	0	2	2.67	0	0	0	0	0	0	0	Bacteria	Gemmatimor	Gemmatimor	Gemmatimor	Gemmatimor	NA
ASV_90	5.33	0	14.33	0	4.67	1	34.5	3.67	0	23	19	1126	Bacteria	Firmicutes	Negativicutes	Veillonellales	Veillonellacea	Veillonella
ASV_91	59	34.67	131	217	0	2.67	0	3.67	0	6	1.67	0.33	Archaea	Crenarchaeo	Thermoprote	Sulfolobales	Sulfolobacea	NA
ASV_92	0.33	3.67	0	0	0.67	0	0	0.67	5	0	2	6.33	Bacteria	Proteobacter	Alphaproteol	Rhizobiales	Xanthobacte	NA
ASV_93	4	2.33	1	2.67	85.67	127.33	33.5	244.33	4	0	3.33	4.33	Bacteria	Firmicutes	Bacilli	Alicyclobacill	Alicyclobacill	Alicyclobacillus
ASV_94	4.33	2.67	6.67	0.33	1	1.33	24	4	0	6	5	768.67	Bacteria	Actinobacter	Actinobacter	Corynebacte	Corynebacte	Lawsonella
ASV_95	537	0.67	2.33	0	2.67	2.33	45	4	0	0	35.33	44.33	Bacteria	Proteobacter	Alphaproteol	Sphingomona	Sphingomona	Sphingomonas
ASV_96	3.67	3.33	6.67	2.33	0	1.33	1	0	0	0	0	0	Bacteria	Firmicutes	Sulfobacillia	Sulfobacillale	Sulfobacillace	NA
ASV_98	2.33	108.33	1.33	81.67	0	12.33	1.5	58.67	0	5	2.67	4.33	Bacteria	Firmicutes	Bacilli	Alicyclobacill	Alicyclobacill	Tumebacillus
ASV_99	0	0.33	38.67	0	4	0.67	0	5.67	0	7689	14.67	3.67	Bacteria	Actinobacter	Thermoleoph	Solirubrobact	67-14	NA
ASV_100	1	2	1	1.67	75.67	389	15.5	30	19	0	2	3.33	Bacteria	Firmicutes	Sulfobacillia	Sulfobacillale	Sulfobacillace	NA
ASV_101	2	3.67	0	0.33	68.67	124.33	128.5	123	2	0	0	5	Archaea	Thermoplasn	Thermoplasn	Thermoplasn	Thermoplasn	A-plasma
ASV_102	226.33	251	4.33	1.67	0.33	4.67	1	3.33	0	0	4.67	7	Bacteria	Proteobacter	Alphaproteol	Rhodobacter	Rhodobacter	Paracoccus
ASV_103	123	187.67	10.67	3.67	2	0.67	8	10.33	78	320	34	179	Bacteria	Proteobacter	Gammaprote	Burkholderia	Burkholderia	Ralstonia
ASV_104	1.67	0.33	0.33	0.33	41.67	30.33	163	195.67	5	2	1.67	1.33	Archaea	Thermoplasn	Thermoplasn	Thermoplasn	Ferroplasma	Ferroplasma
ASV_105	35.33	26.33	36	6.67	1.67	5	32	4.67	110	61	48	255.33	Bacteria	Actinobacter	Actinobacter	Propionibact	Propionibact	Cutibacterium
ASV_106	3.33	3	1	0.33	30	328.67	57	302	8	3	3.67	3	Bacteria	Actinobacter	Acidimicrobii	Acidimicrobia	Acidimicrobia	Aciditerrimonas
ASV_107	2.33	2.67	0	18.67	3	15.33	0	0	3	0	0	1	Bacteria	Firmicutes	Sulfobacillia	Sulfobacillale	Sulfobacillace	Sulfobacillus
ASV_108	486	22.33	1.33	1.33	0	0	1.5	1	0	0	3.33	4.33	Bacteria	Firmicutes	Bacilli	Staphylococc	Staphylococc	Staphylococcus
ASV_109	0.67	2.67	0	0	2.67	0	0	0	4	0	1.33	8.67	Bacteria	Proteobacter	Gammaprote	NA	NA	NA
ASV_110	3.67	1.67	2.67	0	0	0.67	23.5	3.67	0	0	1.67	533	Bacteria	Actinobacter	MB-A2-108	NA	NA	NA
ASV_111	1	1	0	0	0	0	0	2	2	0	1.33	677.33	Bacteria	Actinobacter	Actinobacter	Micrococcale	Dermacoccac	Dermacoccus
ASV_112	1	3	0.33	1.33	118.33	167	8.5	23.67	1	0	2.33	4.67	Bacteria	Actinobacter	Actinobacter	Frankiales	Acidotherma	Acidothermus
ASV_113	0	1	0	0	0.67	0	0	0	2	0	0.67	5.67	Bacteria	Actinobacter	MB-A2-108	NA	NA	NA
ASV_114	0.67	2.67	0	0	0	0	0.5	0.67	0	0	3	658.67	Bacteria	Actinobacter	Actinobacter	Corynebacte	Mycobacteria	Mycobacterium
ASV_115	2.33	2	0.33	1	0	3	0	0.67	19	0	0	0	Bacteria	Firmicutes	NA	NA	NA	NA
ASV_116	0	3.67	0	0	0.67	0	0	0	4	0	0.33	10	Bacteria	Actinobacter	NA	NA	NA	NA
ASV_117	1	2.67	2.33	1	52.67	73.33	37.5	161.67	4	3	1.67	1.67	Bacteria	Firmicutes	Bacilli	Alicyclobacill	Alicyclobacill	Acidibacillus
ASV_118	0	3.33	0	0	1.33	0	0	0.67	0	0	1.67	3.67	Bacteria	Firmicutes	Bacilli	Lactobacillale	Enterococcac	Enterococcus
ASV_119	3	0	1.33	0	3	1.33	25	2	0	9	15	886.33	Bacteria	NA	NA	NA	NA	NA
ASV_120	1.67	21	2.67	0.33	0.33	0	0	8.67	4	0	4.67	612.33	Bacteria	Proteobacter	Gammaprote	Burkholderia	Burkholderia	Burkholderia-Caballeronia-Paraburkholderia
ASV_121	1.67	3	0	0	0	1.33	5	1.33	0	0	2	621.33	Bacteria	Proteobacter	Gammaprote	Pseudomona	Moraxellacea	Acinetobacter
ASV_122	0	0.33	0	0.67	7	0	0	36	11	45	626.33	10.67	Bacteria	Actinobacter	Thermoleoph	Solirubrobact	67-14	NA
ASV_123	0.33	1.67	1.33	0.33	89	220.33	20	81.33	4	3	2.67	3	Bacteria	Firmicutes	Sulfobacillia	Sulfobacillale	Sulfobacillace	Sulfobacillus
ASV_124	1	2.33	0	40.33	11	15	2	2	12	0	0.67	1	Bacteria	Firmicutes	Sulfobacillia	Sulfobacillale	Sulfobacillace	Sulfobacillus
ASV_125	1	0.33	0.33	0	6.33	0	1.5	27.33	5	44	616.67	10.67	Bacteria	Gemmatimor	Longimicrobi	Longimicrobi	Longimicrobi	NA
ASV_126	0	1.33	0	0	1	0	0	0	2	0	1.67	2.67	Bacteria	Planctomyce	Planctomyce	Gemmatales	Gemmatacea	NA
ASV_127	1.67	2	1.33	1	136	299.33	3.5	4.33	11	0	1	3	Bacteria	Firmicutes	Sulfobacillia	Sulfobacillale	Sulfobacillace	Sulfobacillus
ASV_128	3.33	2.67	0	0.67	2	6	0	0.33	0	0	0	1.67	Bacteria	Chloroflexi	Ktedonobact	Ktedonobact	Ktedonobact	NA
ASV_129	68	83.67	26	52	17	22	88	39.67	16	8	18.67	34.33	Bacteria	Acidobacteri	Vicinamibact	Vicinamibact	NA	NA
ASV_130	0	0	0	0	6.33	0	0	21.33	6	23	592	1.67	Bacteria	Planctomyce	Phycisphaera	Tepidisphaer	WD2101 soil	NA
ASV_131	0	2.33	0	0	1	0	0	0	0	0	1.33	6	Bacteria	Actinobacter	Actinobacter	NA	NA	NA
ASV_132	0	1	0.33	0	0	0	0	0	0	0	1.33	5.33	Bacteria	Firmicutes	Bacilli	Staphylococc	Staphylococc	Staphylococcus
ASV_133	3	2.67	1.67	2.67	11.33	33.33	99.5	244	3	4	2	4.33	Bacteria	Dependentia	Babeliae	Babeliales	Babeliaceae	NA
ASV_134	2.33	47.67	25	0	0	5.33	4.5	2.33	13	0	4	459.67	Bacteria	Proteobacter	Gammaprote	Burkholderia	Comamonad	Ottowia
ASV_135	0	2	0.33	0	2.33	0	0	0	1	0	1	4.67	Bacteria	Acidobacteri	Holophagae	Subgroup 7	NA	NA
ASV_136	54.67	94.67	16.33	29	4	6	69.5	41.67	4	3	15.33	34	Bacteria	Acidobacteri	Vicinamibact	Vicinamibact	NA	NA
ASV_137	0.33	0.67	2	0	53.67	339.67	21.5	1	8	0	2.33	4	Bacteria	Planctomyce	Phycisphaera	Tepidisphaer	CPla-3 termit	NA
ASV_138	0	3.67	0	0	1	0	0	0	17	0	1	6.33	Bacteria	Actinobacter	Actinobacter	0319-7L14	NA	NA
ASV_139	2	0.67	1	1	50.33	154.67	15	60.67	5	2	0.67	3	Bacteria	Firmicutes	Sulfobacillia	Sulfobacillale	Sulfobacillace	Sulfobacillus
ASV_140	0.33	2.67	0.33	0	19	113.33	1.5	0	2	4	0.33	1.33	Bacteria	Proteobacter	Alphaproteol	Acetobactera	Acetobactera	Acidicaldus
ASV_141	2	0	2.67	0	5.33	1	16	3.33	0	15	11	708.33	Bacteria	Firmicutes	Bacilli	Bacillales	Bacillaceae	NA
ASV_142	1.33	1.33	41.67	135.33	0	10	0	0	4	2	0.33	1	Bacteria	Actinobacter	Thermoleoph	Solirubrobact	Solirubrobact	NA
ASV_144	2.33	2	5	1	0.33	4	0	1	12	2	0	0.33	Bacteria	Firmicutes	Sulfobacillia	Sulfobacillale	Sulfobacillace	NA
ASV_145	313	0.33	0.67	0.33	1.33	0	0	0	0	0	0	0	Bacteria	Gemmatimor	BD2-11 terre	NA	NA	NA
ASV_146	41	23	41.67	61.67	27.33	14.67	68.5	12.33	11	24	27.67	4.67	Bacteria	Proteobacter	Alphaproteol	Sphingomona	Sphingomona	Sphingomonas
ASV_147	0	0	0	0.33	4	4.33	1	1.33	3838	0	2.33	0.33	Bacteria	Actinobacter	Rubrobacteri	Rubrobactera	Rubrobacteri	Rubrobacter
ASV_148	3	0	0	0	2.33	0	0	4.33	0	13	570.33	10	Bacteria	Bacteroidota	Bacteroidia	Sphingobacte	Sphingobacte	Mucilaginibacter
ASV_150	0.33	2.33	0	1	0	2.67	0	0	3	0	0	0.67	Bacteria	Firmicutes	Sulfobacillia	Sulfobacillale	Sulfobacillace	Sulfobacillus
ASV_151	2	2.67	1.33	0.33	23.67	229	25	118.67	6	0	2.33	2.33	Bacteria	Nitrospirota	Leptospirillia	Leptospirillal	Leptospirillac	Leptospirillum
ASV_152	2.33	0	3	0	3.67	0.67	17.5	3	0	12	13	653.33	Bacteria	Actinobacter	Actinobacter	Corynebacte	Corynebacte	Corynebacterium
ASV_153	0	0.33	0.33	0.33	6	2.67	0.5	11.67	1405	26	299.67	1.33	Bacteria	Proteobacter	Alphaproteol	Rhizobiales	Beijerinckiace	Microvirga
ASV_154	3	1	0.33	7	5	105	4.5	0	7	0	0	1	Bacteria	Firmicutes	YNPFFP2	NA	NA	NA
ASV_155	1.67	1.33	0	0	0	0	0	1.67	0	0	2.33	484.33	Bacteria	Proteobacter	Gammaprote	Burkholderia	Burkholderia	Ralstonia
ASV_156	355	1.33	0	1	0	0	0	1	0	0	1.33	1.33	Bacteria	Proteobacter	Gammaprote	Enterobacter	Erwiniaceae	Erwinia
ASV_157	2	1.67	0	2.67	0	1.33	0	0	0	0	0	1.33	Bacteria	Firmicutes	YNPFFP2	NA	NA	NA
ASV_158	0.33	0	0	0	9.67	9	1	0	3603	0	1.33	0.33	Bacteria	Proteobacter	Gammaprote	Pseudomona	Pseudomona	Pseudomonas
ASV_159	51	41.33	21.33	41.67	10	14.33	127	12.33	4	6	16.67	29.33	Bacteria	Proteobacter	Alphaproteol	Sphingomona	Sphingomona	Sphingomonas
ASV_160	0	0	0	0	4	0	0	21	2	26	460	5.67	Bacteria	Firmicutes	Bacilli	Staphylococc	Staphylococc	Staphylococcus

ASV_161	0	1.67	0	0	0.67	0	0	0	1	0	0.67	3	Bacteria	Actinobacter	MB-A2-108	NA	NA	NA
ASV_162	229.33	13.33	32.33	22.33	0	0.67	0.5	2.67	0	2	1.67	0.33	Archaea	Crenarchaeo	Thermoprote	Sulfolobales	Sulfolobacea	Stygiolobus
ASV_163	3	3.33	0	0	0	2.33	0	0	2	0	0	0	Bacteria	Chloroflexi	Ktedonobact	Ktedonobact	Ktedonobact	Thermogemmatispora
ASV_164	0	0.67	0	0	0	0	0	0	0	0	1	6	Bacteria	Actinobacter	Actinobacter	0319-7L14	NA	NA
ASV_166	0	0	33	0	1	0	0	1.33	0	3983	5.33	2.33	Bacteria	Bacteroidota	Bacteroidia	Chitinophaga	Chitinophaga	NA
ASV_167	1.33	1	0.67	0.67	12.33	226.67	8.5	117.33	8	2	0.67	1.67	Bacteria	Proteobacter	Gammaprote	Acidithiobacil	Acidithiobacil	Acidithiobacillus
ASV_168	1	0.33	0.33	0	0	1.33	0	0	3	0	0	0	Bacteria	Firmicutes	Sulfobacillia	Sulfobacillale	Sulfobacillace	NA
ASV_169	2	0	2	0	2	0.67	18.5	4.67	0	16	15	543.67	Bacteria	Actinobacter	Thermoleopł	Solirubrobact	67-14	NA
ASV_170	45.33	27.33	21	12.67	17	5.33	41.5	40.67	5	0	11.33	27	Bacteria	Gemmatimor	Gemmatimor	Gemmatimor	Gemmatimor	NA
ASV_171	3.33	0.67	4	0	0	1.33	17.5	1	0	0	0.67	327.33	Bacteria	Acidobacteri	Blastocatellia	Pyrinomonad	Pyrinomonad	RB41
ASV_172	0	1.67	0	0	1	0	0	0	3	0	1	5	Bacteria	Actinobacter	Actinobacter	Euzebyales	Euzebyaceae	NA
ASV_173	0	0.67	0	0	28.33	44.33	120.5	189.33	0	2	0.67	1.67	Archaea	Thermoplasn	Thermoplasn	Thermoplasn	Thermoplasn	NA
ASV_174	219	0.33	0.67	0	0.33	0	1.5	0	0	0	0	0.67	Bacteria	Proteobacter	Alphaproteol	Rhizobiales	Beijerinckiace	Methylobacterium-Methylorubrum
ASV_175	36	100.67	47.33	63.67	0	1	0	1.33	0	7	0.33	0.67	Archaea	Crenarchaeo	Thermoprote	Sulfolobales	Sulfolobacea	Stygiolobus
ASV_176	290.33	0	1.67	0	0	1.33	7	2	0	0	2.67	1.67	Bacteria	Proteobacter	Gammaprote	Pseudomona	Cellvibrionac	Cellvibrio
ASV_177	1.67	1.67	54.67	41.33	7.67	29.33	0	0.33	2	0	0	0.33	Bacteria	Firmicutes	Sulfobacillia	Sulfobacillale	Sulfobacillace	Sulfobacillus
ASV_178	70	33.33	16	22	15	2.67	27.5	2.67	0	3	2.67	27	Bacteria	Actinobacter	Actinobacter	Pseudonocar	Pseudonocar	Crossiella
ASV_179	34.67	31	19.67	51	24.67	8	44.5	15.33	16	8	28.67	5.67	Bacteria	Gemmatimor	Gemmatimor	Gemmatimor	Gemmatimor	NA
ASV_180	0.67	1.67	0	0.67	0	1	0	0	0	0	0	0	Bacteria	Chloroflexi	Ktedonobact	Ktedonobact	Ktedonobact	Thermogemmatispora
ASV_181	1	2.33	16.67	104.33	0	5	1	0	0	0	0.67	2.33	Bacteria	Firmicutes	Sulfobacillia	Sulfobacillale	Sulfobacillace	Sulfobacillus
ASV_182	30.67	7.67	25.67	31.67	7.33	34.67	101.5	21.33	4	6	12	13.67	Bacteria	Actinobacter	Thermoleopł	Solirubrobact	NA	NA
ASV_183	1	0.33	1.33	52.33	2.33	1	17.5	1.33	0	7	6.67	397	Bacteria	Firmicutes	Bacilli	Lactobacillale	Aerococcacea	Aerococcus
ASV_184	233.67	0	0	0.67	0.67	0	0	0	0	0	0	0	Bacteria	Actinobacter	MB-A2-108	NA	NA	NA
ASV_185	0	0.33	0.33	0	33.67	378	3	40.33	13	0	2	2	Bacteria	Firmicutes	Sulfobacillia	Sulfobacillale	Sulfobacillace	Sulfobacillus
ASV_186	0.33	0	0	0.33	4	0	0	18.33	3	27	374.67	7	Bacteria	Actinobacter	Rubrobacteri	Rubrobactera	Rubrobacteri	Rubrobacter
ASV_187	0.33	0	0	0	2.67	0	0	18.67	0	21	365.33	3	Bacteria	Proteobacter	Alphaproteol	Rhizobiales	NA	NA
ASV_188	0	0.33	0	0	4.67	0	0.5	20	5	26	366.33	3	Bacteria	Actinobacter	Thermoleopł	Solirubrobact	67-14	NA
ASV_190	48	68.33	39	55	1	0.67	0	1.67	0	7	0	2.33	Archaea	Crenarchaeo	Thermoprote	Sulfolobales	Sulfolobacea	NA
ASV_191	0	0.33	0	0	2	0	0.5	17.33	3	37	363.67	3.33	Bacteria	Actinobacter	Acidimicrobii	NA	NA	NA
ASV_192	4	0.33	0	0	0	0	0	4	0	0	0.33	0.33	Bacteria	Firmicutes	Bacilli	Bacillales	Bacillaceae	NA
ASV_193	2	2	0.33	2.33	1.67	0	6	0	3	0	0.67	1.67	Bacteria	Proteobacter	Gammaprote	Pseudomona	Moraxellacea	Acinetobacter
ASV_194	37	10.33	33	23.67	14	28	52	27.67	0	15	9.67	17.67	Bacteria	Bacteroidota	Bacteroidia	Chitinophaga	Chitinophaga	NA
ASV_196	1.67	2	0	0.33	97.33	77	32.5	107.33	6	4	2.67	2	Bacteria	Firmicutes	Sulfobacillia	Sulfobacillale	Sulfobacillace	Sulfobacillus
ASV_197	0	1	0	0	0.33	0	0	0	0	0	0.33	5	Bacteria	Actinobacter	MB-A2-108	NA	NA	NA
ASV_198	2.67	0.33	2	0	0	0	14	0.33	0	0	0.67	264.67	Bacteria	Firmicutes	Bacilli	Lactobacillale	Lactobacillace	Weissella
ASV_199	0.33	0	0	0	5	2.67	0	0	2762	0	2	0	Bacteria	Bacteroidota	Bacteroidia	Sphingobacte	Sphingobacte	Pedobacter
ASV_200	44.33	52	39.33	50.67	1	0.33	0	1	0	2	0	0.33	Archaea	Crenarchaeo	Thermoprote	Sulfolobales	Sulfolobacea	NA
ASV_201	0.67	0	0	0	0	0	0	9	0	0	0	0	Bacteria	Proteobacter	Gammaprote	Burkholderia	Comamonad	Tepidimonas
ASV_202	0	2.33	0	0	0.67	0	0	0	2	0	0.67	3.67	Bacteria	Acidobacteri	Holophagae	Subgroup 7	NA	NA
ASV_203	2.67	1.33	2.33	0	0	0	10.5	2.33	0	0	1.33	241	Bacteria	Actinobacter	Actinobacter	Micrococcale	Micrococcace	Kocuria
ASV_204	1.67	0	1	0	1.33	0.33	12	1	0	8	224.33	164.67	Bacteria	Proteobacter	Alphaproteol	Sphingomona	Sphingomona	Sphingomonas
ASV_205	0	0.67	0	0	0.67	0	0	0	3	0	0.33	5	Bacteria	Actinobacter	Actinobacter	Micrococcale	Microbacteri	Pseudoclavibacter
ASV_207	170	1.33	26.33	8.67	1.67	0	0	0	0	0	0	0	Bacteria	Proteobacter	Gammaprote	Enterobacter	Pasteurellace	Haemophilus
ASV_208	2.33	0	0	0	0	0	0	9.33	0	0	0	0	Bacteria	Acidobacteri	Vicinamibact	Vicinamibact	NA	NA
ASV_209	0	0.33	0	0	0.33	0	0	0	0	0	1.33	5	Bacteria	Actinobacter	Actinobacter	Frankiales	NA	NA
ASV_210	0	0.33	1.33	0	0	0.33	0	0.33	5	5	0.33	0	Bacteria	Firmicutes	Bacilli	Alicyclobacill	Alicyclobacill	Alicyclobacillus
ASV_211	0.33	0	3	0	6.33	0	7.5	16	7	23	294	2.67	Bacteria	Actinobacter	Actinobacter	Frankiales	Geodermato	Blastococcus
ASV_212	2	0	0.67	0	1.67	1.33	18	2	0	8	9	411.33	Bacteria	Actinobacter	Thermoleopł	Gaiellales	NA	NA
ASV_214	227	0	0	0	0	1.33	3	2	0	0	2	2	Bacteria	Proteobacter	Gammaprote	Pseudomona	Pseudomona	Pseudomonas
ASV_215	0.67	1	0.33	29.33	53.33	145.33	2.5	0.67	3	0	2.33	1.67	Bacteria	Firmicutes	Sulfobacillia	Sulfobacillale	Sulfobacillace	Sulfobacillus
ASV_216	0	2	0	0	0.33	0	0.5	0	2	0	1.33	4	Bacteria	Actinobacter	Actinobacter	Euzebyales	Euzebyaceae	NA
ASV_217	2	1.33	0	0	0	0	0	0.33	0	0	1.33	310	Bacteria	RCP2-54	NA	NA	NA	NA
ASV_219	0.33	0.33	49.33	1.33	1	0	0	1.67	0	2217	2	2	Bacteria	Proteobacter	Gammaprote	Burkholderia	Burkholderia	Ralstonia
ASV_220	1.33	1	1.33	0	59.33	87.33	2.5	65.67	4	0	1.67	1.33	Bacteria	Firmicutes	Sulfobacillia	Sulfobacillale	Sulfobacillace	Sulfobacillus
ASV_221	0.67	3	0.33	0.67	217.33	64.33	34.5	10.67	3	0	3.33	2.33	Bacteria	Actinobacter	Actinobacter	Corynebacter	Mycobacteria	Mycobacterium
ASV_222	1.33	0	0	0	1.33	0	0.5	3	0	6	345	8	Bacteria	Actinobacter	Actinobacter	Euzebyales	Euzebyaceae	NA
ASV_223	54	49.33	33	36	0.33	0.67	0	1.33	0	0	0.67	0.67	Archaea	Crenarchaeo	Thermoprote	Sulfolobales	Sulfolobacea	Stygiolobus
ASV_224	2.33	1.33	0	1.33	0	0.67	0	0	3	2	0	0.33	Bacteria	Firmicutes	YNPFFP2	NA	NA	NA
ASV_225	0.67	1.67	0	0	0	8	0.5	0	3	5	0.67	3	Bacteria	Proteobacter	Gammaprote	Burkholderia	Oxalobactera	Undibacterium
ASV_226	108.33	24.67	15.67	29	0	0.67	0	2.67	0	0	0	0.33	Archaea	Crenarchaeo	Thermoprote	Sulfolobales	Sulfolobacea	Stygiolobus
ASV_227	0.67	1	16.33	0.67	103.33	29	79	24.33	0	2	1.33	0.33	Bacteria	Actinobacter	Actinobacter	Corynebacter	Mycobacteria	Mycobacterium
ASV_228	92	3	1.67	0	0.67	1.33	1.5	0.67	0	11	1	0.33	Bacteria	Actinobacter	Actinobacter	Streptomyce	Streptomyce	Streptomyces
ASV_229	28.33	5.67	12.33	1	14.67	2.67	7	40.33	4	0	2	19.67	Bacteria	Acidobacteri	Vicinamibact	Vicinamibact	NA	NA
ASV_230	169.33	1	0.33	0	1.67	0	0	0	0	0	0	0	Bacteria	Actinobacter	Thermoleopł	Solirubrobact	67-14	NA
ASV_231	10	211	0	6	3	0.33	0	0	0	0	0	0	Bacteria	Firmicutes	Bacilli	Caldalkalibaci	Caldalkalibaci	Caldalkalibacillus
ASV_232	0	0	0	0	2.67	0	0	11.33	4	9	271.33	3.33	Bacteria	Proteobacter	Alphaproteol	Rhizobiales	NA	NA
ASV_233	1.67	2.33	0	2.33	1.33	0	1	2.67	0	6	245.67	8.67	Bacteria	Actinobacter	Actinobacter	Corynebacter	Corynebacter	Turicella
ASV_234	29	10	14.67	2	12.67	4.67	17.5	36.33	6	0	3.33	18.67	Bacteria	Acidobacteri	Vicinamibact	Vicinamibact	NA	NA
ASV_235	5.67	4.33	229.33	0	0.33	2	26	0	0	0	4.33	8.67	Bacteria	Firmicutes	Bacilli	Staphylococc	Staphylococc	Staphylococcus
ASV_236	53.67	3.67	2.67	0	0.33	0	5	1.67	0	0	11	136	Bacteria	Proteobacter	Gammaprote	Burkholderia	Comamonad	NA
ASV_237	0.67	0	1.33	0	3	0	18	2.67	0	8	12	348.33	Bacteria	Actinobacter	Thermoleopł	Solirubrobact	67-14	NA
ASV_238	5.67	0.33	10	0	4.33	0	0.5	1.67	8	12	227	2	Bacteria	Firmicutes	Bacilli	Bacillales	Bacillaceae	NA
ASV_239	1.67	1	0	5.67	0	2.33	0	0.33	0	0	0	1.67	Bacteria	Actinobacter	Acidimicrobii	IMCC26256	NA	NA
ASV_241	0.33	1.67	2	0.33	14.67	192.33	9	5.67	10	0	0.67	2.67	Bacteria	Actinobacter	Acidimicrobii	NA	NA	NA
ASV_242	19.33	11.33	10.67	19	15.67	12	34	13.67	1	4	16	9.67	Bacteria	Actinobacter	Thermoleopł	Gaiellales	Gaiellaceae	Gaiella
ASV_243	0.33	0	53	0.33	0	0	0.5	0.33	0	1731	2.33	2.67	Bacteria	Actinobacter	Actinobacter	Micrococcale	Cellulomonac	Actinotalea
ASV_244	0	1.67	0	0	0.33	0	0	0.67	0	0	0.67	3.67	Bacteria	Actinobacter	Thermoleopł	Solirubrobact	67-14	NA
ASV_245	0	0.33	0	0	0.33	0	0	0	0	0	0	5.33	Bacteria	Proteobacter	Gammaprote	Burkholderia	Oxalobactera	Massilia
ASV_246	1.33	0	0.33	0	0	0	0	2.33	0	6	286	8.33	Bacteria	Proteobacter	Alphaproteol	Rhizobiales	Beijerinckiace	Microvirga
ASV_248	60.67	1	0.33	1	2	2.67	11.5	7.33	3	13	135	1.33	Bacteria	Actinobacter	Rubrobacteri	Rubrobactera	Rubrobacteri	Rubrobacter
ASV_249	1.33	0.67	0.33	1	4.33	14.67	50	100	0	3	1.67	2	Bacteria	Dependentia	Babeliae	Babeliales	Babeliaceae	NA
ASV_250	0	0	0	0	1.33	0	0	11.67	4	18	254.67	3	Bacteria	Chloroflexi	Chloroflexia	Kallotenuales	AKIW781	NA
ASV_251	0.67	0	0	0.67	2	0	0.5	15.33	2	19	252.67	2	Bacteria	Actinobacter	Thermoleopł	Solirubrobact	67-14	NA
ASV_252	0	0.33	0	0	0	0	0	0	3	0	0.33	2.67	Bacteria	Chloroflexi	OLB14	NA	NA	NA
ASV_253	0	224.33	0	0	0	0.67	0	0	0	0	0	1	Bacteria	Cyanobacteri	Sericytochroı	NA	NA	NA
ASV_254	1.67	0	206.33	0	0	0	28.5	0	0	0	7.33	8	Bacteria	Proteobacter	Alphaproteol	Rhizobiales	Rhizobiaceae	NA
ASV_255	0.67	0.67	0	0.33	0	2.33	0	1	10	0	0	0.33	Bacteria	Firmicutes	Sulfobacillia	Sulfobacillale	Sulfobacillace	NA
ASV_256	0.33	0	0.33	0.33	51.67	71.67	25	26.33	0	0	1	0	Bacteria	Firmicutes	Sulfobacillia	Sulfobacillale	Sulfobacillace	Sulfobacillus
ASV_257	0	1.33	0	0	0	0	0.5	0	2	0	1	2.33	Bacteria	Chloroflexi	Anaerolineae	Ardenticaten	Ardenticaten	NA
ASV_258	0	1.67	0	0	0.33	0	0	0	0	0	0.33	3.33	Bacteria	Actinobacter	Actinobacter	Micrococcale	Intrasporangi	Oryzihumus
ASV_259	0	205	0	0	0	1.67	0	0	0	0	0	2	Bacteria	Actinobacter	Thermoleopł	Gaiellales	NA	NA
ASV_260	11.67	0.33	2.33	0	0	0	11.5	0.67	0	0	2	108.67	Bacteria	Proteobacter	Gammaprote	Enterobacter	Enterobacter	Escherichia-Shigella
ASV_261	0.67	3.33	0.67	6.33	0	1.67	9.5	0.33	0	5	2.33	180	Bacteria	Actinobacter	Actinobacter	Micrococcale	Dermabacter	Brachybacterium
ASV_262	1.67	0.67	0	0.33	0.67	143	2.5	15.33	0	0	0.67	1	Bacteria	Proteobacter	Alphaproteol	Acetobactera	Acetobactera	NA
ASV_263	0	1.33	0	0	0	0	0	0	0	0	1.67	2	Bacteria	Actinobacter	Actinobacter	Corynebacter	Corynebacter	Corynebacterium
ASV_264	0	0.33	0	0	1.67	0.33	0	12	0	23	236.67	4	Bacteria	Actinobacter	Thermoleopł	Gaiellales	NA	NA
ASV_265	12.33	25.33	21.67	17.33	0	1	0.5	2	0	0	0	0.33	Archaea	Crenarchaeo	Thermoprote	Sulfolobales	Sulfolobacea	Stygiolobus
ASV_266	1.67	0	2.67	0	1	1.67	18.5	0.67	0	2	165.67	104.33	Bacteria	Proteobacter	Alphaproteol	Rhodobacter	Rhodobacter	Amaricoccus
ASV_267	41.33	42	9.67	29.67	0.67	0	0	1.33	0	2	0.33	0	Archaea	Crenarchaeo	Thermoprote	Sulfolobales	Sulfolobacea	Stygiolobus
ASV_268	0	1	0	0	0.67	0.33	0	0	3	0	1	2.33	Bacteria	Bacteroidota	Bacteroidia	Flavobacteria	Weeksellacea	Empedobacter
ASV_270	2.33	0.33	111	0	0.33	0.33	20	0	0	0	3	6	Bacteria	Firmicutes	Bacilli	Lactobacillale	Enterococcac	Enterococcus
ASV_271	0.33	0	0	0	2.67	0	1	13	2	7	231.33	1.33	Bacteria	Actinobacter	Thermoleopł	Solirubrobact	67-14	NA
ASV_272	0	0.33	0	0	0	0	0	0	0	0	0.67	0.33	Bacteria	Cyanobacteri	Sericytochroı	NA	NA	NA
ASV_273	29.33	15.33	10	13	5	19.33	35.5	10	3	6	6.33	15	Bacteria	Actinobacter	Thermoleopł	Gaiellales	NA	NA
ASV_274	2.67	4.67	2.67	0.67	0	0	3.5	0.67	0	0	0.33	165.33	Bacteria	Firmicutes	Bacilli	Staphylococc	Staphylococc	Staphylococcus
ASV_275	0.33	0.67	0	0.33	38.33	176	8.5	0.67	5	0	1.67	2	Bacteria	Actinobacter	Acidimicrobii	Acidimicrobia	Acidimicrobia	Acidimicrobium
ASV_276	16	5	12.67	19.67	5.67	24.67	41.5	17	3	0	5.33	9.67	Bacteria	Actinobacter	Thermoleopł	Solirubrobact	Solirubrobact	Solirubrobacter
ASV_278	1	0	115.33	16.33	0	13.67	8.5	3.67	2	2	1.33	1.67	Bacteria	Actinobacter	Thermoleopł	Solirubrobact	Solirubrobact	Conexibacter
ASV_279	0	0	0	0	0.33	0	0.5	4	0	0	25.67	0.33	Bacteria	Proteobacter	Gammaprote	Burkholderia	Rhodocyclace	Zoogloea
ASV_281	2.33	2.67	2	0	0	0	3.5	0.67	0	0	1	178.33	Bacteria	Firmicutes	Bacilli	Bacillales	Bacillaceae	Aeribacillus
ASV_282	1.33	1.67	0	0.33	7.67	74	25.5	55.67	0	0	0.33	0.67	Bacteria	Actinobacter	Actinobacter	Frankiales	Acidotherma	Acidothermus
ASV_283	0.67	0	0	0	3	3.33	0	0	1622	0	3.67	1	Bacteria	Bacteroidota	Bacteroidia	Flavobacteria	Weeksellacea	Cloacibacterium
ASV_284	0.67	0	1.33	0	1	0	1.5	5.67	2	7	64	4	Bacteria	Proteobacter	Gammaprote	Burkholderia	Comamonad	Hydrogenophaga
ASV_285	0	0	7	0	1.33	0	0	3	0	2063	5	1.67	Bacteria	Actinobacter	Thermoleopł	Solirubrobact	Solirubrobact	NA
ASV_286	0.67	0	0	0	0.33	0	1	2.67	0	0	240.67	7	Bacteria	Proteobacter	Gammaprote	Burkholderia	Comamonad	Hydrogenophaga
ASV_287	0.67	16	0.67	0	3.33	6.67	35	2	8	0	4	219	Bacteria	Proteobacter	Gammaprote	Pseudomona	Moraxellacea	Acinetobacter
ASV_288	3	0	0.67	0	2.67	1	35	0.67	0	0	413.33	5.33	Bacteria	Proteobacter	Gammaprote	Enterobacter	Enterobacter	NA
ASV_289	1	0.33	0	0.33	4	103	25.5	1.67	4	0	0.67	1.67	Bacteria	RCP2-54	NA	NA	NA	NA
ASV_290	20.67	8.33	18.33	12.33	2.33	18.67	27	12.33	2	11	6.33	8	Bacteria	Actinobacter	Thermoleopł	Solirubrobact	Solirubrobact	NA
ASV_291	11.33	11.33	11.67	1.67	7	1.67	15.5	12.67	2	3	1.33	27	Bacteria	Chloroflexi	TK10	NA	NA	NA
ASV_292	0	0.33	0	0	1	0.67	2.5	2.33	2	7	48.67	4.33	Bacteria	Proteobacter	Gammaprote	Burkholderia	Oxalobactera	Massilia
ASV_293	4	1	4.33	0.33	1	1	4	4.33	0	0	0.33	1	Bacteria	Actinobacter	Thermoleopł	Gaiellales	Gaiellaceae	Gaiella
ASV_294	0	0.33	0	0	2	0	0	10	2	16	199	2.67	Bacteria	Actinobacter	Actinobacter	Pseudonocar	Pseudonocar	NA
ASV_295	10.33	0	105	45	0.33	1	12	0.33	0	0	3	3.67	Bacteria	Proteobacter	Gammaprote	Pseudomona	Pseudomona	Pseudomonas
ASV_296	1.33	1	0.33	0	0.33	5.33	0.5	148.33	1	0	1	0.33	Bacteria	Firmicutes	Bacilli	Alicyclobacill	Alicyclobacill	Alicyclobacillus
ASV_297	0	0	0	0	2	0	0	8.33	2	12	208.33	1	Bacteria	Chloroflexi	Chloroflexia	Kallotenuales	Kallotenuace	Kallotenue
ASV_298	1.33	0	2	0	2.33	0.67	14	2	0	12	6	256.67	Bacteria	Actinobacter	Actinobacter	0319-7L14	NA	NA
ASV_299	0	0.67	0	0	2.33	0	0	8.67	1	19	188	3	Bacteria	Actinobacter	Actinobacter	Propionibact	Nocardioidac	Nocardioides
ASV_300	0	1.33	0	0	0.33	0	0	0	0	0	0.33	1.67	Bacteria	Proteobacter	Alphaproteol	Rhizobiales	NA	NA
ASV_301	2	15	9	7.67	0.33	0	2.5	67.33	2	0	5.33	31.33	Bacteria	Firmicutes	Bacilli	Bacillales	Bacillaceae	Ornithinibacillus
ASV_302	2.33	0	1.67	0	1.67	2	15.5	0.33	0	0	405.33	2.67	Bacteria	Proteobacter	Gammaprote	Enterobacter	Enterobacter	NA
ASV_303	1	0.67	0.33	0.67	9.33	62.67	9.5	39.67	0	0	1	1	Bacteria	Actinobacter	Acidimicrobii	Acidimicrobia	Acidimicrobia	Aciditerrimonas
ASV_304	0	1.33	0.33	0	0	0.33	0	0	0	0	0	0.33	Bacteria	Firmicutes	NA	NA	NA	NA
ASV_305	0	0.33	0	0	1.33	0	0	10.33	2	4	198	2	Bacteria	Proteobacter	Alphaproteol	Sphingomona	Sphingomona	Ellin6055
ASV_306	11	5	5.67	26.33	10.67	5.67	15.5	7.33	3	4	10	1.33	Bacteria	Acidobacteri	Blastocatellia	Blastocatellal	Blastocatellac	Blastocatella
ASV_307	0.33	0	0	0	0.33	0	107	118	0	4	1.67	0.33	Archaea	Thermoplasn	Thermoplasn	Thermoplasn	Ferroplasmac	Ferroplasma
ASV_308	19.33	22	8.67	12.33	1	4	25	8.33	1	2	3.67	9	Bacteria	Proteobacter	Alphaproteol	Sphingomona	Sphingomona	NA
ASV_309	1.67	0.33	5.67	0	1.33	0.33	1.5	5.67	13	5	141.67	1.33	Bacteria	Actinobacter	Thermoleopł	Solirubrobact	67-14	NA
ASV_310	0.33	1	0.33	0.33	0	0.33	0.5	0	0	0	0.67	3.33	Bacteria	Actinobacter	Thermoleopł	Solirubrobact	67-14	NA
ASV_311	0.33	0	0	60.67	0	5	0	1.67	0	0	0	0.67	Bacteria	Actinobacter	Actinobacter	NA	NA	NA
ASV_313	0.33	0	0	0	4.33	73	8.5	44.33	2	0	1	1.33	Bacteria	RCP2-54	NA	NA	NA	NA
ASV_314	1	0	0.33	0	1.33	0.67	0	3.33	0	4	53.33	4.67	Bacteria	Proteobacter	Gammaprote	Burkholderia	Comamonad	Paucibacter
ASV_315	26.67	3.33	12.67	9.33	2	6	3	13.67	0	0	5.33	12.67	Bacteria	Proteobacter	Gammaprote	Nitrosococca	Nitrosococca	wb1-P19
ASV_316	0.67	0	5.67	0	51	67.67	1.5	0	6	0	0.33	0.33	Bacteria	Firmicutes	Sulfobacillia	Sulfobacillale	Sulfobacillace	NA
ASV_318	116.33	0.33	0	0.67	0	0	0	0	0	0	0	0	Bacteria	Gemmatimor	S0134 terrest	NA	NA	NA
ASV_319	0	173	0	0	0	1.33	0	0	0	0	0	2	Bacteria	Cyanobacteri	Sericytochroı	NA	NA	NA
ASV_320	0.33	0.33	0	0	0	0	0	2	0	0	1	171.67	Bacteria	Actinobacter	Actinobacter	Streptomyce	Streptomyce	Streptomyces

ASV_321	20.33	67	10.33	15.67	0	0	0	1.33	0	2	1	0.33	Archaea	Crenarchaeo	Thermoprote	Sulfolobales	Sulfolobacea	Stygiolobus
ASV_322	0	0	0.33	0	0	0	0	11.33	0	11	192.67	1.33	Bacteria	Gemmatimor	Longimicrobi	Longimicrobi	Longimicrobi	NA
ASV_323	2	0	4	0	0.33	0.33	7	0.67	0	0	1.67	59	Bacteria	Bacteroidota	Bacteroidia	Flavobacteria	Weeksellacea	Cloacibacterium
ASV_324	20.67	6.33	12.67	10	6	13.33	32	11.67	3	5	2.33	7.33	Bacteria	Actinobacter	Thermoleoph	Solirubrobact	67-14	NA
ASV_325	0.33	0	7.33	0	0.67	0	0	2	0	1509	24.67	1	Bacteria	Actinobacter	Thermoleoph	Solirubrobact	Solirubrobact	NA
ASV_326	2.33	6.67	1.67	0	0	0	6.5	2	0	15	0.67	117.67	Bacteria	Proteobacter	Gammaprote	Burkholderia	Burkholderia	Ralstonia
ASV_327	121.67	1	0	0.67	0	0	0	0.33	0	0	0	1.33	Bacteria	Firmicutes	Bacilli	Staphylococc	Staphylococc	Staphylococcus
ASV_328	1	0	0	0	0.33	0	0	3.33	0	0	0	0	Bacteria	Acidobacteri	Vicinamibact	Vicinamibact	NA	NA
ASV_329	0.67	0.33	20	0.33	0	1	0	0.33	3	0	0	0	Bacteria	Firmicutes	Sulfobacillia	Sulfobacillale	Sulfobacillace	Sulfobacillus
ASV_330	0	0	0	0	1.67	0	0	14	5	19	170.33	2.33	Bacteria	Actinobacter	Thermoleoph	Solirubrobact	67-14	NA
ASV_331	101.33	0	0	0.67	0	0	0	0	0	0	0	0	Bacteria	Proteobacter	Gammaprote	Pseudomona	Pseudomona	Pseudomonas
ASV_332	0.33	0	0	0	2.67	0	0	9.33	0	12	166.67	2.67	Bacteria	Actinobacter	Actinobacter	Micrococcale	Dermacoccac	Dermacoccus
ASV_333	0	0.33	4	0	1	0	0	0.33	0	1709	3.67	1	Bacteria	Firmicutes	Bacilli	Thermicanale	Thermicanac	Thermicanus
ASV_334	17.67	5.33	13	11	5.33	11	26	8.67	3	10	5.67	8.33	Bacteria	Actinobacter	Thermoleoph	Gaiellales	Gaiellaceae	Gaiella
ASV_335	1.33	0	0	0	0	0.33	0	1.67	0	5	176.33	3.67	Bacteria	Proteobacter	Alphaproteol	Caulobactera	Caulobactera	Phenylobacterium
ASV_336	0	0.67	0	1	0	0	0	0	0	0	0	2.33	Bacteria	Acidobacteri	Vicinamibact	Vicinamibact	Vicinamibact	NA
ASV_337	2	0	0	0	0.67	0	0	2.33	0	4	188	4.33	Bacteria	Proteobacter	Alphaproteol	Sphingomona	Sphingomona	Sphingomonas
ASV_338	0	0	0	0	1.33	3.33	0	0.33	1226	0	1.33	0	Bacteria	Actinobacter	Thermoleoph	Solirubrobact	67-14	NA
ASV_339	0	1	0	0	0	0	0	0	0	0	0	1.33	Bacteria	Actinobacter	Thermoleoph	Solirubrobact	67-14	NA
ASV_340	0	0.33	0.67	0	3	0.67	0	0.67	1240	0	1.67	0	Bacteria	Actinobacter	Thermoleoph	Solirubrobact	67-14	NA
ASV_341	12	7.67	4.33	11	4.33	7.67	27	8.67	2	3	4.33	2	Bacteria	Proteobacter	Alphaproteol	Sphingomona	Sphingomona	Sphingomonas
ASV_342	0.33	0	0.33	0.33	44.33	79.33	3.5	2.33	3	0	1	0.67	Bacteria	Firmicutes	Sulfobacillia	Sulfobacillale	Sulfobacillace	Sulfobacillus
ASV_344	71	0.33	0	5	0.33	0.33	3	0	43	0	0	2.33	Bacteria	Proteobacter	Alphaproteol	Sphingomona	Sphingomona	Sphingomonas
ASV_345	98	0.33	1.33	0.67	0.33	0	0	0	0	0	0.67	0	Bacteria	Actinobacter	Rubrobacteri	Rubrobactera	Rubrobacteri	Rubrobacter
ASV_346	98.67	1.67	8.67	0.67	0	0	1.5	0.67	0	0	0	0	Bacteria	Proteobacter	Gammaprote	Pseudomona	Pseudomona	Pseudomonas
ASV_347	113.33	0.67	0	0	0	0	0	0	0	0	0.67	0	Bacteria	Proteobacter	Gammaprote	Burkholderia	Comamonada	Simplicispira
ASV_348	99.67	0	0	0.33	0.67	0	0	0	0	0	0.33	0	Bacteria	Actinobacter	Rubrobacteri	Rubrobactera	Rubrobacteri	Rubrobacter
ASV_349	0.33	0	0	0	0.33	0	0	6.33	1	10	161.67	3.33	Bacteria	Chloroflexi	TK10	NA	NA	NA
ASV_350	0	0.33	0	0	1	0	0	21.33	2	8	137.33	2.33	Bacteria	Actinobacter	Acidimicrobii	IMCC26256	NA	NA
ASV_351	0.33	0.33	0.33	0.33	35	51	1	17	1	0	0	0.67	Bacteria	Firmicutes	Sulfobacillia	Sulfobacillale	Sulfobacillace	Sulfobacillus
ASV_352	0.33	91.67	0.67	0	0.67	2.67	4	0	1	0	73	1.33	Bacteria	Proteobacter	Gammaprote	Pseudomona	Pseudomona	Pseudomonas
ASV_353	20	6.33	7.33	12.33	3.67	12.33	35.5	5.33	1	6	5.33	6	Bacteria	Actinobacter	Thermoleoph	Solirubrobact	67-14	NA
ASV_354	0	0.67	0	0.33	8	81.67	3	0	4	0	0.33	1.67	Bacteria	Actinobacter	Acidimicrobii	IMCC26256	NA	NA
ASV_355	0	0	0	0	1.67	0	0	10	3	14	129	1	Bacteria	Actinobacter	Thermoleoph	NA	NA	NA
ASV_356	27	38	1.67	0.33	0.33	0.67	2	1	17	61	8.33	43.33	Bacteria	Proteobacter	Gammaprote	Burkholderia	Burkholderia	Ralstonia
ASV_357	0.67	0	61.33	0	0.33	0.67	12	0	0	299	3.67	35.67	Bacteria	Actinobacter	Actinobacter	Micrococcale	Microbacteri	NA
ASV_358	25	6.67	5.67	19.67	0.67	3.67	4.5	8.33	2	0	4.33	3.33	Bacteria	Actinobacter	Thermoleoph	Solirubrobact	67-14	NA
ASV_359	0	128.33	0	0	0	0.67	0	0	0	0	0	0.33	Bacteria	Proteobacter	Gammaprote	Pseudomona	Halomonada	Halomonas
ASV_360	0	0	0	0	0	0	0	0.33	0	0	0.67	2.33	Bacteria	Actinobacter	Acidimicrobii	IMCC26256	NA	NA
ASV_361	90.67	0.33	0.33	0	0	0	0	0	0	0	0	0	Bacteria	Proteobacter	Alphaproteol	Rhizobiales	Beijerinckiac	Methylobacterium-Methylorubrum
ASV_362	3	0	3.33	0	3	1.67	40	0	0	0	247	6.67	Bacteria	Bacteroidota	Bacteroidia	Chitinophaga	Chitinophaga	Taibaiella
ASV_363	0	0.33	0	0	51	76	4	1.67	6	2	0.33	1	Bacteria	Firmicutes	Sulfobacillia	Sulfobacillale	Sulfobacillace	NA
ASV_364	5	93.33	0	1.33	0.67	0	0.5	0	4	12	0	11.33	Bacteria	Proteobacter	Gammaprote	Burkholderia	Burkholderia	Ralstonia
ASV_365	0	2.67	0	9.67	0	0	0.5	0	0	0	0	3.67	Bacteria	Fusobacterio	Fusobacteriia	Fusobacterial	Fusobacteria	Fusobacterium
ASV_366	0.33	2	7	0	7.33	1.67	9	2	11	8	5	1	Bacteria	Bacteroidota	Bacteroidia	Cytophagales	Hymenobact	Adhaeribacter
ASV_367	0.67	0.33	0	0.33	0	8.67	0	0	0	0	0	0.67	Bacteria	Proteobacter	Alphaproteol	Elsterales	NA	NA
ASV_369	24.33	13.33	1	19.33	1	3.33	19.5	0	3	0	1	1	Bacteria	Proteobacter	Alphaproteol	Sphingomona	Sphingomona	Sphingomonas
ASV_370	0.33	0	0	0	0.67	0	0	0.33	0	6	154.67	4	Bacteria	Actinobacter	Acidimicrobii	Microtrichale	Ilumatobacte	CL500-29 marine group
ASV_371	0	1	0.33	0	2	2	5.5	6	4	9	135.33	3.33	Bacteria	Firmicutes	Bacilli	Lactobacillale	Streptococca	Streptococcus
ASV_372	0.67	46	0.33	0	0.33	1	6	2.67	0	3	2	62.67	Bacteria	Actinobacter	Rubrobacteri	Rubrobactera	Rubrobacteri	Rubrobacter
ASV_373	81.33	5	1	0.67	0	0	1.5	0	0	0	0	0.67	Bacteria	Proteobacter	Gammaprote	Pseudomona	Pseudomona	Pseudomonas
ASV_374	1	0	0	0	0	0	0	2	0	5	150.67	4	Bacteria	Firmicutes	Bacilli	Paenibacillale	Paenibacillac	NA
ASV_376	0	1	0	0	0	0	0	0	0	0	0	2.33	Bacteria	Actinobacter	Thermoleoph	Gaiellales	NA	NA
ASV_377	3	6	48.67	31.67	0	0	0	0	0	0	0	0	Archaea	Crenarchaeo	Thermoprote	Sulfolobales	Sulfolobacea	NA
ASV_378	0.67	1.67	0	0	0	1.33	0	0	0	0	0	1	Bacteria	RCP2-54	NA	NA	NA	NA
ASV_379	0.33	0	8.33	0	0	0	1	0.67	0	0	0.67	0.67	Bacteria	Proteobacter	Alphaproteol	Sphingomona	Sphingomona	Sphingobium
ASV_380	0.67	0.67	0	0	28.33	55.67	2.5	0	2	0	0.33	0.33	Bacteria	Chloroflexi	AD3	NA	NA	NA
ASV_382	0	0	0	0	1.67	2	1.5	0	971	0	1.33	0	Bacteria	Actinobacter	Thermoleoph	Gaiellales	NA	NA
ASV_383	66.67	0	0.67	0	0	0.33	1	0.67	6	4	1.33	0.33	Bacteria	Proteobacter	Alphaproteol	Rhizobiales	Beijerinckiac	Methylobacterium-Methylorubrum
ASV_384	45.33	7	6.33	5.67	0.33	1	0	0	0	0	0	0.67	Archaea	Crenarchaeo	Thermoprote	Sulfolobales	Sulfolobacea	Stygiolobus
ASV_385	0.33	0.67	1	0	0	0	2.5	0.67	0	0	0	109.33	Bacteria	Actinobacter	MB-A2-108	NA	NA	NA
ASV_386	85	2	0.33	0	0.67	0	0	0	0	0	0	0	Bacteria	Proteobacter	Gammaprote	Enterobacter	Idiomarinace	Idiomarina
ASV_387	0	0	0	0	0.67	1.33	0	0	985	0	0.33	0	Bacteria	Proteobacter	Alphaproteol	Rhizobiales	Beijerinckiac	Microvirga
ASV_388	0	1.33	0	0	0	0	0	0.67	0	2	0.67	125.67	Bacteria	Proteobacter	Gammaprote	Pseudomona	Halomonada	Halomonas
ASV_389	0.33	0	0.33	0.33	0	0.33	0	0	0	0	0	0.33	Bacteria	Actinobacter	Actinobacter	Frankiales	Acidotherma	Acidothermus
ASV_390	0	0	0	0	1	0	0	6.33	1	6	136	2	Bacteria	Dadabacteria	Dadabacterii	Dadabacteria	NA	NA
ASV_391	0.33	0	23	0	1	0	1	0.33	0	839	0.67	1	Bacteria	Proteobacter	Gammaprote	Burkholderia	Nitrosomona	NA
ASV_392	23.33	11.33	1.33	18.33	0.33	5	13.5	1.33	0	0	1.33	2.67	Bacteria	Actinobacter	Actinobacter	0319-7L14	NA	NA
ASV_393	0	0	0	0	0	0	0	5	2	10	126	0.67	Bacteria	Actinobacter	Rubrobacteri	Rubrobactera	Rubrobacteri	Rubrobacter
ASV_394	11.33	6.33	1	0	0	0	0.5	0.67	3	18	1	105	Bacteria	Proteobacter	Gammaprote	Burkholderia	Burkholderia	Ralstonia
ASV_395	1.67	0	109.67	0	0	0.67	21.5	0	0	0	3.33	4.33	Bacteria	Bacteroidota	Bacteroidia	Flavobacteria	Weeksellacea	Chryseobacterium
ASV_396	7.67	1.67	4.67	13	3.67	10.67	37.5	10.33	0	0	2.33	7.67	Bacteria	Chloroflexi	KD4-96	NA	NA	NA
ASV_397	0	0.67	0	0	0	0	0	0	0	0	0.33	1.33	Bacteria	Actinobacter	Actinobacter	Frankiales	Geodermato	Geodermatophilus
ASV_398	0	1	0	0	0	0	0.5	0	0	0	0	1	Bacteria	Firmicutes	Bacilli	Lactobacillale	Streptococca	Streptococcus
ASV_399	11.67	4.33	14.33	7.33	3	9	18.5	10	0	23	3.33	6.33	Bacteria	Actinobacter	Acidimicrobii	Microtrichale	Ilumatobacte	NA
ASV_400	0	0	0	0	1	0	0	0	0	0	0	3	Bacteria	Actinobacter	Thermoleoph	Gaiellales	NA	NA
ASV_401	0	0	5	0	15.33	94	2	1.33	3	0	1	0	Bacteria	Firmicutes	Sulfobacillia	Sulfobacillale	Sulfobacillace	Sulfobacillus
ASV_402	0	1	0	0	0	0	0	0	0	0	0	0.33	Bacteria	Actinobacter	Actinobacter	0319-7L14	NA	NA
ASV_403	86	1.67	0.67	0.67	0	0.33	6	0	0	0	0.33	0.33	Bacteria	Proteobacter	Alphaproteol	Azospirillales	Azospirillace	Skermanella
ASV_404	1.33	35	24.33	34.33	0	0	0	0.67	0	0	0.33	0.33	Archaea	Crenarchaeo	Thermoprote	Sulfolobales	Sulfolobacea	NA
ASV_405	0.33	1.67	0	5	0	0	0	0.33	0	0	70	2	Bacteria	Actinobacter	Rubrobacteri	Rubrobactera	Rubrobacteri	Rubrobacter
ASV_406	9	10.67	7	20.33	0.67	3.67	21.5	4	0	0	0.67	4.33	Bacteria	Actinobacter	Actinobacter	0319-7L14	NA	NA
ASV_407	1	0	0	0	0	0	0	0.67	0	0	0.67	111.33	Bacteria	Firmicutes	Bacilli	Lactobacillale	Streptococca	Streptococcus
ASV_408	0	0	0	0	2.33	0	0	5.67	0	4	124	1.33	Bacteria	Actinobacter	Actinobacter	Euzebyales	Euzebyaceae	NA
ASV_409	0.67	0	83.67	0	0	0.33	8	0	0	4	0.33	1	Bacteria	Actinobacter	Actinobacter	Actinomyceta	Actinomyceta	Actinomyces
ASV_410	0	0	0.33	0	1.33	2.33	0	0	876	0	0.33	0	Bacteria	Actinobacter	Thermoleoph	Solirubrobact	Solirubrobact	Solirubrobacter
ASV_411	1.33	0	1.67	0	0	0	0.5	0.33	0	0	0	0	Bacteria	Proteobacter	Alphaproteol	Sphingomona	Sphingomona	Sphingobium
ASV_412	79.33	0	0	1	1	0	0	0	0	0	0	0	Bacteria	Firmicutes	Bacilli	Lactobacillale	Carnobacteri	Trichococcus
ASV_413	0	0	0	74	0	0.33	0	0	0	0	1.33	0	Bacteria	Actinobacter	Actinobacter	Micromonos	Micromonos	NA
ASV_414	70.67	0.67	2.33	0.67	0	0	0	0	0	0	0.33	0	Bacteria	Proteobacter	Gammaprote	Pseudomona	Pseudomona	Pseudomonas
ASV_415	0	0	0.33	0	0	1	0	0	2	0	0	0	Bacteria	Firmicutes	Sulfobacillia	Sulfobacillale	Sulfobacillace	Sulfobacillus
ASV_416	1.33	0	1.33	0	0.67	0.33	6.5	1	0	2	2.67	160.33	Bacteria	Actinobacter	Actinobacter	Streptospora	Streptospora	Motilibacter
ASV_417	0.67	0	0	0	0.67	0	0	3.33	0	6	121	2.67	Bacteria	Actinobacter	Actinobacter	Micrococcale	Micrococcace	NA
ASV_418	1	0	0.33	0	0.67	1.33	14	0	0	0	224	2	Bacteria	Proteobacter	Gammaprote	Enterobacter	Enterobacter	Klebsiella
ASV_419	0.33	0	1.67	0.33	1.33	0.67	12	0	0	0	231	2.67	Bacteria	Proteobacter	Gammaprote	Enterobacter	Enterobacter	NA
ASV_420	12.67	4	13	10.33	4	5.67	19	2.33	4	6	5	3	Bacteria	Actinobacter	Thermoleoph	Gaiellales	NA	NA
ASV_421	0.33	0	0	0	0.67	0.67	6.5	1	0	0	1.33	154.33	Bacteria	Firmicutes	Negativicutes	Veillonellales	Veillonellace	Veillonella
ASV_422	1	0	2	0	0	0	5.5	0.67	0	0	3.67	153.67	Bacteria	Proteobacter	Gammaprote	Burkholderia	Comamonada	NA
ASV_423	1.67	0	93.67	0	0	0	18	0	0	0	3.67	5.67	Bacteria	Bacteroidota	Bacteroidia	Flavobacteria	Weeksellacea	NA
ASV_424	0	0.33	0	0	0	0	0	0	2	0	0	0.33	Bacteria	Proteobacter	Alphaproteol	Sphingomona	Sphingomona	Ellin6055
ASV_425	0	0	0.33	0	1.33	2.67	4.5	130	0	0	0	1	Bacteria	Firmicutes	Sulfobacillia	Sulfobacillale	Sulfobacillace	Sulfobacillus
ASV_426	0	0	0	0	12.33	41.33	0.5	0.33	0	0	0	0	Bacteria	Firmicutes	Sulfobacillia	Sulfobacillale	Sulfobacillace	Sulfobacillus
ASV_427	0	0	0	0	15	43	0.5	0.67	0	0	0.33	0	Bacteria	Actinobacter	Acidimicrobii	NA	NA	NA
ASV_428	0.67	0.33	1	0	0	0	1.5	0	0	0	0	82.33	Bacteria	Firmicutes	Bacilli	Caldalkalibaci	Caldalkalibaci	Caldalkalibacillus
ASV_429	2.33	4.33	7.67	1.33	4.67	1.33	10.5	4	2	0	4.33	18.67	Bacteria	Actinobacter	Actinobacter	Propionibact	Nocardioidac	Nocardioides
ASV_430	60.67	0	0	0.33	0	0	0	0	0	0	0	0	Bacteria	Chloroflexi	TK10	NA	NA	NA
ASV_432	0	0	0	0	0	0	0	0	0	0	1	1	Bacteria	Actinobacter	Actinobacter	Propionibact	Nocardioidac	Nocardioides
ASV_433	12.33	4.33	12.67	4	3	5	14	5	0	19	1.33	1	Bacteria	Actinobacter	Thermoleoph	Solirubrobact	Solirubrobact	Conexibacter
ASV_434	0	0	0	0	19.67	4	30.5	26.33	0	0	0.33	44	Bacteria	Actinobacter	Actinobacter	Corynebacte	Mycobacteria	Mycobacterium
ASV_435	0.33	0	1	0	1	0.67	5	1.33	0	0	2.33	140	Bacteria	Actinobacter	Actinobacter	Corynebacte	Corynebacte	Corynebacterium
ASV_436	0	0	9	0	0	0	0	2	0	923	2.33	0.33	Bacteria	Actinobacter	Thermoleoph	Solirubrobact	67-14	NA
ASV_437	0	95	0	0	0	0.33	0	0	0	0	0	1	Bacteria	Proteobacter	Gammaprote	Burkholderia	Comamonada	Sphaerotilus
ASV_438	0.33	0	76	0	0	0.67	15	0	0	0	3.67	5.33	Bacteria	Actinobacter	Actinobacter	Micrococcale	Micrococcace	Kocuria
ASV_439	62	1	4	3.67	0	0.67	0	0.33	0	0	0.33	0.33	Archaea	Crenarchaeo	Thermoprote	Sulfolobales	Sulfolobacea	Stygiolobus
ASV_441	1	0	0.67	0	1	0	3.5	0	0	4	3.33	136.33	Bacteria	Actinobacter	Actinobacter	Corynebacte	Corynebacte	Corynebacterium
ASV_442	0	77	0	0	0	0	0	1	0	0	0	0	Archaea	Crenarchaeo	Nitrososphae	Group 1.1c	NA	NA
ASV_444	4.33	30.33	4	2.67	1	0.33	3	10	7	0	3.67	9.33	Bacteria	Acidobacteri	Vicinamibact	Vicinamibact	Vicinamibact	NA
ASV_445	7.33	8.33	4.67	7.33	0	0.67	6.5	5.67	3	0	0.67	2.67	Bacteria	Acidobacteri	Blastocatellia	Pyrinomonad	Pyrinomonad	RB41
ASV_446	10	2.33	4.33	6.33	4.33	6.67	15	5.33	0	0	4	10.33	Bacteria	Actinobacter	Thermoleoph	Solirubrobact	67-14	NA
ASV_447	0	0	0.33	0	0.33	0.33	5	0.67	0	3	3	134	Bacteria	Proteobacter	Gammaprote	Burkholderia	Comamonada	NA
ASV_448	0.33	0.33	34.33	27.33	0	0	0	0	0	0	0	0.33	Archaea	Crenarchaeo	Thermoprote	Sulfolobales	Sulfolobacea	Stygiolobus
ASV_449	0.33	3	0	0	0	0	0	2	0	4	105.33	2.33	Bacteria	Firmicutes	Bacilli	Bacillales	Bacillaceae	Aeribacillus
ASV_450	0.33	1	0.33	0	0	0	1	0	0	0	1.33	1.33	Bacteria	Firmicutes	Bacilli	Staphylococc	Staphylococc	Staphylococcus
ASV_451	0	0	0	0.33	16.33	34	4	0.67	1	0	0	0.33	Bacteria	Firmicutes	Sulfobacillia	Sulfobacillale	Sulfobacillace	Sulfobacillus
ASV_452	10	3.33	4.33	5.67	1	1.67	14.5	5.67	0	2	1.67	5	Bacteria	Actinobacter	Acidimicrobii	Microtrichale	Ilumatobacte	NA
ASV_453	0.67	0.33	0	0	0	0	3.5	0.67	0	0	0	78.33	Bacteria	Firmicutes	Bacilli	Staphylococc	Staphylococc	Nosocomiicoccus
ASV_454	1.33	0	0	0	0.33	0	0	5.67	0	5	112.33	0.33	Bacteria	Actinobacter	Thermoleoph	Solirubrobact	67-14	NA
ASV_455	0	0	0	0	0	0	0	4.67	0	6	95	0	Bacteria	Actinobacter	Actinobacter	0319-7L14	NA	NA
ASV_456	0	1	1.33	0.67	0.33	2.33	0	4.67	0	5	1	1	Bacteria	Proteobacter	Gammaprote	Pseudomona	Pseudomona	Pseudomonas
ASV_457	57	0	3.33	1	0	0	0.5	0	0	0	0.33	0	Bacteria	Proteobacter	Gammaprote	Pseudomona	Pseudomona	Pseudomonas
ASV_458	0	0	0	1.33	0	13.33	0	0	0	0	0	0	Bacteria	RCP2-54	NA	NA	NA	NA
ASV_459	1	84	0	0	0	0	0	0	0	0	0	0.33	Bacteria	Proteobacter	Alphaproteol	Rhizobiales	Beijerinckiac	Methylobacterium-Methylorubrum
ASV_461	0.67	0	0	0	0	0	0	0.67	0	0	112	2.67	Bacteria	Proteobacter	Gammaprote	Enterobacter	Enterobacter	NA
ASV_462	0.33	0	1.33	0	0	0	0	0	0	0	0.33	1	Bacteria	Bacteroidota	Bacteroidia	Flavobacteria	Weeksellacea	Cloacibacterium
ASV_463	0	0	0	0	1.67	0	0	4.67	1	6	93.67	0.67	Bacteria	NA	NA	NA	NA	NA
ASV_464	0.33	0	0.33	0	0	0.33	5	0.33	0	2	2	137.67	Bacteria	Actinobacter	Actinobacter	Actinomyceta	Actinomyceta	Actinomyces
ASV_466	64	1	0	0.33	0	0	0	0	0	0	0.33	1.67	Bacteria	Proteobacter	Gammaprote	Burkholderia	Comamonada	NA
ASV_467	12.33	7	4.33	7	0	2	33.5	1.67	0	0	3.33	6.67	Bacteria	Actinobacter	Thermoleoph	Gaiellales	Gaiellaceae	Gaiella
ASV_468	30	3	0	4.67	0	0	0	0	0	0	0.33	3.67	Bacteria	Actinobacter	Actinobacter	Corynebacte	Corynebacte	Turicella
ASV_469	0	0	0	0	1	2.67	0	2	0	9	3	1	Bacteria	Actinobacter	Thermoleoph	Solirubrobact	Solirubrobact	NA
ASV_470	0	0.33	0	0	16.67	53.67	0	0	0	0	0.33	0.33	Bacteria	Firmicutes	Sulfobacillia	Sulfobacillale	Sulfobacillace	Sulfobacillus
ASV_471	0	0	0	0	1.67	0	0	10.33	0	6	91	1	Bacteria	Proteobacter	Gammaprote	Pseudomona	Moraxellacea	Acinetobacter
ASV_472	0	0.67	0	0	0.67	0	0	0	0	0	0.67	2.67	Bacteria	Actinobacter	Actinobacter	Pseudonocar	Pseudonocar	Pseudonocardia
ASV_473	1.33	1	2.67	0.67	0.33	0.67	0	0	0	7	0.67	0	Bacteria	Firmicutes	Bacilli	Lactobacillale	Listeriaceae	Brochothrix
ASV_474	0	77	0	0	0	0.33	0	0	0	0	0	0.33	Bacteria	Firmicutes	Clostridia	Eubacteriales	Garciellaceae	Rhabdanaerobium
ASV_475	0.67	2	18	12.67	0	0.33	0	0.67	0	5	1	0.33	Archaea	Crenarchaeo	Thermoprote	Sulfolobales	Sulfolobacea	NA
ASV_476	0	0	0	0	0.67	0.67	0	0.33	690	0	0.67	0	Bacteria	Actinobacter	Actinobacter	Corynebacte	Corynebacte	Corynebacterium

ASV_477	1.33	1	1	0.67	1	1.33	9	0.33	0	0	0	0	Bacteria	Actinobacter	Actinobacter	Micrococcale	Micrococcace	Pseudarthrobacter
ASV_478	2.67	0	52	0	0	0	0	0	0	0	0	0	Bacteria	Proteobacter	Gammaprote	Pseudomona	Pseudomona	Pseudomonas
ASV_479	0	0	0	0	34.33	27	1.5	0	0	0	0.33	0.33	Archaea	Thermoplasn	Thermoplasn	Thermoplasn	Thermoplasn	Thermoplasma
ASV_480	7.67	12.67	4.67	5	7	2	7.5	7	0	6	6.33	4.33	Bacteria	Gemmatimor	Gemmatimor	Gemmatimor	Gemmatimor	NA
ASV_481	0	0.33	0	0	0	1.33	0	0.33	0	9	2.33	4	Bacteria	Actinobacter	Thermoleoph	Solirubrobact	67-14	NA
ASV_482	5.67	17.67	2	0.67	0.33	1.33	9	0	0	0	0	0.33	Bacteria	Actinobacter	Actinobacter	Corynebacter	Corynebacter	Corynebacterium
ASV_483	7	1.33	0.33	8.67	3	9.67	21.5	6	2	0	3.67	5	Bacteria	Actinobacter	Actinobacter	0319-7L14	NA	NA
ASV_484	1	0	54	0	0	0	0	0	0	2	0.67	0	Bacteria	Actinobacter	Actinobacter	0319-7L14	NA	NA
ASV_485	0	79	0	0.33	0	1	1	1.33	2	0	0	0	Bacteria	Proteobacter	Gammaprote	Burkholderia	Oxalobactera	Massilia
ASV_486	2.33	2	3.33	6.67	4.67	3.67	21	2.33	0	0	4.33	11	Bacteria	Actinobacter	Thermoleoph	Solirubrobact	Solirubrobact	Conexibacter
ASV_487	0.33	0	0	0.67	4.67	1	0	12.67	0	0	0	0.67	Bacteria	Firmicutes	Bacilli	Alicyclobacilla	Alicyclobacilla	Acidibacillus
ASV_488	0	0.67	0	0	0	0	0	0	0	0	0	0.67	Bacteria	Chloroflexi	KD4-96	NA	NA	NA
ASV_489	10	5.33	7.67	7.67	1.67	2.33	14	4.67	1	2	2	2.33	Bacteria	Chloroflexi	Chloroflexia	Thermomicro	JG30-KF-CM4	NA
ASV_490	0	0	0.67	0	0.67	1	0	0	663	0	0	0	Bacteria	Actinobacter	Thermoleoph	Solirubrobact	67-14	NA
ASV_491	10.33	7	1.67	9.33	3.67	1	18.5	1	0	3	4	4	Bacteria	Acidobacteri	Vicinamibact	Vicinamibact	NA	NA
ASV_492	0	0	0	0	0	0	0	0	0	0	0	0	Archaea	Crenarchaeo	Nitrososphae	Group 1.1c	NA	NA
ASV_493	0.33	0	60.33	0	0	0	0	0	0	3	0	0	Bacteria	Proteobacter	Gammaprote	Pseudomona	Pseudomona	Pseudomonas
ASV_494	70.33	0	0.67	0	0	0	0	0.33	0	0	0	0.33	Archaea	Crenarchaeo	Thermoprote	Sulfolobales	Sulfolobacea	Stygiolobus
ASV_495	0	0	0	0	0	0	0	0	0	0	0	0.67	Bacteria	Actinobacter	Thermoleoph	Solirubrobact	67-14	NA
ASV_496	0.67	0	0	0	0	0	0	2	0	0	0	0	Bacteria	Bacteroidota	Bacteroidia	Cytophagales	Microscillace	Ohtaekwangia
ASV_497	7	0.67	6.33	10	1.67	6.67	16	4	2	2	2	5	Bacteria	Actinobacter	Actinobacter	0319-7L14	NA	NA
ASV_498	2	4.33	2.67	0	0	0	0.5	1.67	0	0	2.67	1.33	Bacteria	Actinobacter	Actinobacter	Euzebyales	Euzebyaceae	NA
ASV_499	1.67	1	0	0	0	0	0	0	0	0	0	0	Bacteria	Chloroflexi	Ktedonobact	Ktedonobact	Ktedonobact	NA
ASV_500	6.33	1.33	2.67	6.67	2	7.33	11.5	7	0	0	2.33	5.33	Bacteria	Actinobacter	Thermoleoph	Solirubrobact	Solirubrobact	Conexibacter
ASV_501	0	0	0	0	0	0	0	0	0	0	0	1	Bacteria	Actinobacter	Actinobacter	Frankiales	Sporichthyac	Sporichthya
ASV_502	0	77	0	0.33	0	0	0	2	0	0	0	0	Bacteria	Proteobacter	Alphaproteol	Rhizobiales	Xanthobacter	Rhodopseudomonas
ASV_503	51.33	0	4.67	0.67	0	0	1.5	0	0	0	0	0.67	Bacteria	Proteobacter	Gammaprote	Pseudomona	Pseudomona	Pseudomonas
ASV_504	0.33	0.33	0	0	5	1.67	14	5.67	0	0	2.33	64.33	Bacteria	Actinobacter	Actinobacter	Corynebacter	Mycobacteria	Mycobacterium
ASV_505	0.67	0	0.33	0	0.67	1.67	13.5	0	0	0	158	3.67	Bacteria	Gemmatimor	Longimicrobi	Longimicrobi	Longimicrobi	NA
ASV_506	8.33	2	3	5	4.33	5.67	16.5	6.33	0	3	2.33	7	Bacteria	Actinobacter	Acidimicrobii	Microtrichale	Iamiaceae	Iamia
ASV_507	8	3.33	3	7.33	4	5.33	18.5	7.67	5	4	6	4.33	Archaea	Crenarchaeo	Nitrososphae	Nitrososphae	Nitrososphae	Candidatus Nitrocosmicus
ASV_508	0	0.33	0	0	0	0	0	0	6	0	1.67	12.67	Bacteria	Proteobacter	Gammaprote	Pseudomona	Moraxellacea	Enhydrobacter
ASV_509	8.67	3.67	9	6.33	0	7	14	4.33	0	3	2	3	Bacteria	Chloroflexi	Chloroflexia	Thermomicro	JG30-KF-CM4	NA
ASV_510	0.67	0	0.67	12	0.33	0	10.5	0	0	0	1	0	Bacteria	Bacteroidota	Bacteroidia	Chitinophaga	Chitinophaga	Hydrotalea
ASV_511	6	3	3.67	5.67	3.67	5.67	17.5	9	2	5	2	3	Bacteria	Actinobacter	Thermoleoph	Solirubrobact	Solirubrobact	Conexibacter
ASV_512	0	71.67	0	0	0	0.33	0	0	0	0	0	0.67	Bacteria	Actinobacter	Actinobacter	Corynebacter	Nocardiaceae	Nocardia
ASV_513	0	0	0	0	0.67	0	0	3.33	0	7	77.33	1	Bacteria	Proteobacter	Gammaprote	Nitrosococca	Nitrosococca	SZB85
ASV_514	0	0	55	0	0	0	0	0	0	3	0	0	Bacteria	Chloroflexi	KD4-96	NA	NA	NA
ASV_515	0	0	0.67	0	0	5	0	0	0	0	0	0.33	Bacteria	Proteobacter	Alphaproteol	Acetobactera	Acetobactera	Acidicaldus
ASV_516	8.33	6.33	2.33	4.33	2.67	4	7	3	5	4	1.67	6.33	Bacteria	Actinobacter	Thermoleoph	Solirubrobact	67-14	NA
ASV_517	0	0	0	0	0	0	0	0	0	0	0	1.67	Bacteria	Acidobacteri	Blastocatellia	Pyrinomonad	Pyrinomonad	RB41
ASV_518	5.67	61.33	0	1.33	0	0	0	0	0	0	0	0.67	Bacteria	Proteobacter	Gammaprote	Enterobacter	Idiomarinace	Idiomarina
ASV_519	6.33	0.33	70	0.33	0	0	6.5	0	0	0	2.33	1	Bacteria	Proteobacter	Gammaprote	Pseudomona	Pseudomona	Pseudomonas
ASV_520	0	0	0	0	1	0	0	5.67	1	6	76	0.33	Bacteria	Actinobacter	Thermoleoph	Gaiellales	NA	NA
ASV_521	0	0	0	0	0.33	1	1	0	608	0	0	0	Bacteria	Proteobacter	Gammaprote	Burkholderia	Comamonada	Tepidimonas
ASV_522	1.33	0.33	0.33	0	24.67	6	40.5	20	0	0	0.33	1.33	Bacteria	Actinobacter	Actinobacter	Corynebacter	Mycobacteria	Mycobacterium
ASV_523	0.33	0	51.33	0	0	0	0	0	0	0	0	0	Bacteria	Proteobacter	Gammaprote	Pseudomona	Pseudomona	Pseudomonas
ASV_524	0.33	0.67	0.33	0	2.67	36.33	6.5	26	1	0	0.33	0	Bacteria	Firmicutes	Sulfobacillia	Sulfobacillale	Sulfobacillace	Sulfobacillus
ASV_525	1.67	0	1.33	0	1.33	1	21.5	0	0	0	139	3	Bacteria	Proteobacter	Alphaproteol	Rhizobiales	Xanthobacter	NA
ASV_526	0	0	0	0	4.33	0.67	0	0	0	0	0	2.67	Bacteria	Actinobacter	Rubrobacteri	Rubrobactera	Rubrobacteri	Rubrobacter
ASV_527	0	0.33	0	0	0	0	0	0	21	0	0	1.67	Bacteria	Actinobacter	Rubrobacteri	Rubrobactera	Rubrobacteri	Rubrobacter
ASV_528	0	0	0	0	0	0.67	0	0	0	0	0	0.67	Bacteria	Acidobacteri	Acidobacteria	Acidobacteria	Acidobacteria	Acidobacterium
ASV_529	0	72.67	0	0	0	0.33	0	0	0	0	0	0	Bacteria	Proteobacter	Alphaproteol	Rhizobiales	Rhizobiaceae	Falsochrobactrum
ASV_530	0	0	0	0	1.67	0.33	0	0	576	0	0	0	Bacteria	Proteobacter	Gammaprote	Enterobacter	Shewanellace	Shewanella
ASV_531	0	0	0	0	0.67	6.33	1.5	4.33	1	3	78.33	0.33	Bacteria	Actinobacter	Thermoleoph	Solirubrobact	67-14	NA
ASV_532	0	0	0	4	0	5	0	0	0	0	0	0	Bacteria	Firmicutes	Sulfobacillia	Sulfobacillale	Sulfobacillace	NA
ASV_533	0.67	0	0.67	0	0	0	2	0.67	0	0	0	61.33	Bacteria	Proteobacter	Gammaprote	Burkholderia	Comamonada	NA
ASV_534	0	0	0	0	0.67	0	0	3.67	0	2	75.33	0.33	Bacteria	Firmicutes	Bacilli	Staphylococc	Staphylococc	Staphylococcus
ASV_535	0	0	28.33	12.33	0	0	6.5	0	0	0	0	0	Bacteria	Proteobacter	Alphaproteol	Sphingomona	Sphingomona	NA
ASV_536	0	0	0	51	0	0.33	0	0	0	0	0	0	Bacteria	Proteobacter	Alphaproteol	Sphingomona	Sphingomona	Porphyrobacter
ASV_537	8	5.33	4.67	3	3	5.33	16.5	4	2	3	1.33	3.67	Bacteria	Chloroflexi	KD4-96	NA	NA	NA
ASV_538	0	36.33	4	0	1.33	0	0.5	20.67	2	4	1.33	8.33	Bacteria	Actinobacter	Thermoleoph	Solirubrobact	67-14	NA
ASV_539	7.67	3.67	1.33	6	2	2	10.5	3.33	0	4	0.67	3	Bacteria	Proteobacter	Alphaproteol	Sphingomona	Sphingomona	Sphingomonas
ASV_540	6	0.67	15	3.33	5	5	14	2.67	0	21	2.33	2.67	Bacteria	Actinobacter	Rubrobacteri	Rubrobactera	Rubrobacteri	Rubrobacter
ASV_541	0	1	10.33	32.33	0	0	0	0	0	0	0	0	Archaea	Crenarchaeo	Thermoprote	Thermoprote	Thermoprote	Vulcanisaeta
ASV_542	0.67	0	0	0.33	13.67	13.67	6	13.67	0	0	0	0.33	Bacteria	Firmicutes	Bacilli	Alicyclobacilla	Alicyclobacilla	Acidibacillus
ASV_543	5.67	3.67	3.33	11.67	7	2	4.5	1	0	9	5	0.33	Bacteria	Acidobacteri	Blastocatellia	Blastocatellal	Blastocatellac	Blastocatella
ASV_545	8	3.33	1.33	5.67	0.67	3	17.5	1.33	1	0	2.33	0.67	Bacteria	Proteobacter	Gammaprote	Xanthomona	Xanthomona	Luteimonas
ASV_546	9.67	3	6.67	4.33	1	5.33	9	3	0	15	2.33	3	Bacteria	Actinobacter	Actinobacter	Frankiales	NA	NA
ASV_547	0.33	0	0	0	20.67	4	36	20	0	0	0	1.33	Bacteria	Actinobacter	Actinobacter	Corynebacter	Mycobacteria	Mycobacterium
ASV_548	0	0	44.67	0	0	0	0	0	0	0	0	0	Bacteria	Proteobacter	Gammaprote	Legionellales	Legionellacea	Legionella
ASV_549	7.33	3	5.67	4.67	5	5	11.5	4.33	2	0	1.67	4	Bacteria	Actinobacter	Actinobacter	Frankiales	Geodermatop	NA
ASV_550	1	0.33	0	0	0	0	0	0	0	0	0	66.67	Bacteria	Firmicutes	Clostridia	Oscillospirale	Ruminococca	Ruminococcus
ASV_551	5.33	2	3.33	6.33	1	8.67	14.5	5.67	0	2	3	3.33	Bacteria	Actinobacter	Actinobacter	Propionibact	Nocardioidac	Nocardioides
ASV_552	0	0	0	0	6	7.33	17	40.33	0	0	0.67	0.67	Archaea	Thermoplasn	Thermoplasn	Thermoplasn	Thermoplasn	A-plasma
ASV_553	0	0	0	0	0.67	1.33	0	0	525	0	0	0	Bacteria	Actinobacter	Actinobacter	Frankiales	NA	NA
ASV_554	8.33	3.33	5.67	5	1.67	5.67	7	2.33	0	3	4.33	4.33	Bacteria	Actinobacter	Thermoleoph	Solirubrobact	Solirubrobact	Solirubrobacter
ASV_555	0	0	0	0	0.67	0	0	3.67	3	4	74.33	0.67	Bacteria	Actinobacter	Actinobacter	NA	NA	NA
ASV_556	0.33	0	0	0	0	0	0	0	0	0	0	0	Bacteria	Firmicutes	YNPFFP2	NA	NA	NA
ASV_557	0.33	0.33	0	0	0	3.33	0.5	0	2	0	0	0	Bacteria	Firmicutes	NA	NA	NA	NA
ASV_558	0.67	0	0.67	0	0	0	1.5	0.33	0	0	0.33	54.67	Bacteria	Actinobacter	Actinobacter	Micrococcale	Micrococcace	Micrococcus
ASV_559	5.33	4.33	14	3.33	1	3	39.5	0.33	0	0	0.33	2	Bacteria	Proteobacter	Gammaprote	Pseudomona	Moraxellacea	Acinetobacter
ASV_560	7.67	5	0	3.33	4	14.67	49.5	0.33	0	0	0.33	0	Bacteria	Actinobacter	Rubrobacteri	Rubrobactera	Rubrobacteri	Rubrobacter
ASV_561	0	0	12.67	0	0.33	0	0	17	0	278	0	1	Bacteria	Proteobacter	Gammaprote	Pseudomona	Halomonada	Halomonas
ASV_562	0	0	0	0	0.67	0.33	0	0	512	0	0.33	0	Bacteria	Actinobacter	Thermoleoph	Solirubrobact	67-14	NA
ASV_563	0	0	0	0	0	0	0	0	0	0	0	0.67	Bacteria	Actinobacter	MB-A2-108	NA	NA	NA
ASV_564	0	32.33	6	0.33	0.33	0	0.5	15	4	0	2	7	Bacteria	Actinobacter	Acidimicrobii	IMCC26256	NA	NA
ASV_565	4.67	2	3.33	4.33	2.67	4.33	14.5	6.67	0	2	1	3	Bacteria	Actinobacter	Thermoleoph	Solirubrobact	Solirubrobact	NA
ASV_566	1	0	0.33	0	0	0	5	0.67	0	0	0.67	92.67	Bacteria	Actinobacter	Actinobacter	Micrococcale	Brevibacteria	Brevibacterium
ASV_567	51.67	0.33	0	0	0	0	0.5	0.67	0	0	0.33	0	Bacteria	Deinococcota	Deinococci	Thermales	Thermaceae	Meiothermus
ASV_568	0	0	1.33	0	0	0	0	0	0	0	0.33	0	Bacteria	Proteobacter	Alphaproteol	Caulobactera	Hyphomonac	SWB02
ASV_569	0	0	0	0	0.33	0	0	0	0	0	0	1.67	Bacteria	Actinobacter	Actinobacter	0319-7L14	NA	NA
ASV_570	0	2	0.33	1.33	0	0	0	0.33	0	0	0	1.67	Bacteria	Actinobacter	Thermoleoph	Solirubrobact	Solirubrobact	NA
ASV_571	0.33	0.33	0.67	0	0	0	5.5	0.67	0	0	0.33	48	Bacteria	Actinobacter	Actinobacter	Propionibact	Propionibact	Cutibacterium
ASV_572	11	9.67	2.33	4.33	0.67	1	0	0	0	0	0	0	Archaea	Crenarchaeo	Thermoprote	Sulfolobales	Sulfolobacea	Stygiolobus
ASV_573	0	0	0	0	1.33	14	13	20	0	2	0	0.33	Bacteria	Actinobacter	Actinobacter	Frankiales	Acidotherma	Acidothermus
ASV_574	0	0	0.33	0	0	0	1	0.67	0	0	0.33	0.67	Bacteria	Bacteroidota	Bacteroidia	Flavobacteria	Weeksellacea	Chryseobacterium
ASV_575	2	4	4.33	1.33	2	3	10.5	5	3	0	0.33	8	Bacteria	Actinobacter	Acidimicrobii	NA	NA	NA
ASV_576	4	4.33	7.33	7	8.33	1.67	6	0.67	0	6	4	0.33	Bacteria	Acidobacteri	Blastocatellia	Blastocatellal	Blastocatellac	Blastocatella
ASV_577	0	0.67	0.33	0	3	30	0	0.33	0	0	0	1	Bacteria	Firmicutes	Sulfobacillia	Sulfobacillale	Sulfobacillace	Sulfobacillus
ASV_578	15	0	1.67	0	0	0	0	0	0	0	1.67	35.67	Bacteria	Proteobacter	Gammaprote	Burkholderia	Comamonada	NA
ASV_579	0	0	0	0	0	0	0	0.33	0	0	0	0	Bacteria	Actinobacter	Thermoleoph	Gaiellales	NA	NA
ASV_580	0	0	0	0	0.33	0	2.5	0	0	0	0.67	84.67	Bacteria	Proteobacter	Gammaprote	Pseudomona	Moraxellacea	Enhydrobacter
ASV_581	1.33	0	0.67	0	0	0	2.5	0	0	0	0.33	0	Bacteria	Proteobacter	Alphaproteol	Rhizobiales	Beijerinckiace	Methylobacterium-Methylorubrum
ASV_582	0	49.67	0	0	0	0.33	0	0	0	0	0	0	Bacteria	Chloroflexi	Chloroflexia	Kallotenuales	AKIW781	NA
ASV_583	2	0.33	7	2	3	6	14	4.33	3	6	3.33	3.33	Bacteria	Verrucomicro	Verrucomicro	Chthoniobact	Chthoniobact	Chthoniobacter
ASV_584	3	0.33	45.67	0	0.33	0	4.5	0	0	0	1	1.67	Bacteria	Proteobacter	Gammaprote	Pseudomona	Pseudomona	Pseudomonas
ASV_585	0	0.33	0	0	0	0	0	0	0	0	0	0.67	Bacteria	Acidobacteri	Holophagae	Subgroup 7	NA	NA
ASV_586	0	0	0	0	0	0	0	0	0	0	79	2.33	Bacteria	Proteobacter	Gammaprote	Burkholderia	Comamonada	Caenimonas
ASV_587	34.67	0	0.67	0	0	0	0	0	0	0	0	0	Bacteria	Actinobacter	MB-A2-108	NA	NA	NA
ASV_588	5	2.33	2.33	3.33	1	6.33	26	2	2	2	0.67	3.33	Bacteria	Actinobacter	Thermoleoph	Gaiellales	Gaiellaceae	Gaiella
ASV_589	0.33	0	51	0	0	0.33	8.5	0	0	0	1.67	1.67	Bacteria	Firmicutes	Bacilli	Lactobacillale	Carnobacteri	Granulicatella
ASV_590	0.67	0.67	0	0.67	0	0.33	0	0	0	0	0	0	Bacteria	Armatimonac	Chthonomon	Chthonomon	Chthonomon	Chthonomonas
ASV_591	4	0	45.33	0.67	0	0.67	3	0	0	0	1	1.67	Bacteria	Proteobacter	Gammaprote	Pseudomona	Pseudomona	Pseudomonas
ASV_592	0	0	0.33	0	0	0	1	0	0	0	0	0.33	Bacteria	Actinobacter	Actinobacter	Pseudonocar	Pseudonocar	Actinomycetospora
ASV_593	0.67	0	0	0	0.67	0	7	0.67	0	0	123	2	Bacteria	Proteobacter	Gammaprote	Enterobacter	Enterobacter	Klebsiella
ASV_594	0.67	0	0	0	0	0	0	1.67	0	0	0	0	Bacteria	Proteobacter	Alphaproteol	Rhodobacter	Rhodobacter	Paracoccus
ASV_595	0	0	0.67	0	0	0	0.5	0	0	0	0.67	1	Bacteria	Proteobacter	Gammaprote	Burkholderia	Comamonada	NA
ASV_596	0	52.67	0	0	0	1.33	0	0	0	0	0	0.33	Bacteria	Acidobacteri	Blastocatellia	Blastocatellal	Blastocatellac	NA
ASV_597	0	0	0	0	0	0	0	0	1	0	0	0	Bacteria	Firmicutes	YNPFFP2	NA	NA	NA
ASV_598	6	2.33	1.67	3.33	0.33	6.33	24.5	2.33	5	0	1	2.33	Bacteria	Actinobacter	Acidimicrobii	IMCC26256	NA	NA
ASV_599	0	0	0	0	0.33	0.33	0	0	465	0	0	0	Bacteria	Chloroflexi	Chloroflexia	Kallotenuales	AKIW781	NA
ASV_600	0.33	0.33	0	0	0	7.33	0	0	1	0	0	0	Bacteria	Actinobacter	Acidimicrobii	IMCC26256	NA	NA
ASV_601	0	0	0	0	0.33	0	0	5	2	3	58	0.67	Bacteria	Chloroflexi	JG30-KF-CM6	NA	NA	NA
ASV_602	0	0	0	0	0	0	0	0.67	0	0	0	0	Bacteria	Firmicutes	Bacilli	Bacillales	Bacillaceae	Bacillus
ASV_603	0	0	0.67	0	1	0	2.5	0	0	2	1	82.67	Bacteria	Firmicutes	Negativicutes	Veillonellales	Veillonellacea	Veillonella
ASV_604	3	1	3	1.67	10.33	5	5.5	1.33	2	3	10.33	1.67	Bacteria	Acidobacteri	Blastocatellia	Blastocatellal	Blastocatellac	Blastocatella
ASV_605	0	0	0	0	0	0.33	8	33.33	0	0	0	0	Archaea	Thermoplasn	Thermoplasn	Thermoplasn	Thermoplasn	A-plasma
ASV_606	6.67	3	6	2.67	2.33	3.33	5.5	6	2	0	1	3.33	Bacteria	Actinobacter	Acidimicrobii	NA	NA	NA
ASV_607	0	0	0	0	0	0	0	2	0	0	0	0	Bacteria	Actinobacter	Actinobacter	Streptomyce	Streptomyce	Streptomyces
ASV_608	0.33	0	41	0	0	0	0	0	0	0	0	0	Bacteria	Proteobacter	Gammaprote	Pseudomona	Pseudomona	Pseudomonas
ASV_609	0.33	0.33	0	0.33	0	0	0	0	0	0	0	0	Bacteria	Firmicutes	YNPFFP2	NA	NA	NA
ASV_610	0.33	0	41	0	1	2	2.5	0.67	0	0	3	7.67	NA	NA	NA	NA	NA	NA
ASV_611	3.33	2	2	3.33	3.33	7.33	25	4.33	0	4	0	0.33	Bacteria	Actinobacter	Acidimicrobii	Microtrichale	Ilumatobacte	NA
ASV_612	0	0	0	0	0.33	0.33	0	0	446	0	0.67	0	Bacteria	Proteobacter	Alphaproteol	Rhodobacter	Rhodobacter	Rubellimicrobium
ASV_613	4.67	1.67	3.33	5	2	3.33	13	3.67	0	3	2	1.33	Bacteria	Actinobacter	Thermoleoph	Gaiellales	Gaiellaceae	Gaiella
ASV_614	0.33	0.67	0.33	0	0	5	0.5	0	0	0	0	0	Bacteria	Chloroflexi	Ktedonobact	Ktedonobact	Ktedonobact	NA
ASV_615	0	0	0	0	0	2	0	0	0	0	0	0.33	Bacteria	Firmicutes	Sulfobacillia	Sulfobacillale	Sulfobacillace	NA
ASV_616	0	0.33	0	0	0	0.33	0	0	0	5	1	0.67	Bacteria	Actinobacter	Thermoleoph	Solirubrobact	67-14	NA
ASV_617	0.33	0	0.33	0	0	0	0	0	0	2	0.33	50	Bacteria	Actinobacter	Actinobacter	Micrococcale	Micrococcace	Nesterenkonia
ASV_618	25	0.33	6.67	5.67	0	0	1	0	0	0	0	0	Bacteria	Proteobacter	Gammaprote	Pseudomona	Pseudomona	Pseudomonas
ASV_619	0	0	0	0	1	0	0	3	0	5	58	1.33	Bacteria	Actinobacter	Actinobacter	Propionibact	Nocardioidac	Marmoricola
ASV_620	0	0	0	0	0	0.67	0	0	0	0	0.67	1.67	Bacteria	Proteobacter	Gammaprote	Pseudomona	Pseudomona	Pseudomonas
ASV_621	0.33	0	1	0	0.67	0.33	2	0	0	0	87.67	1	Bacteria	Proteobacter	Gammaprote	Enterobacter	Enterobacter	NA
ASV_622	0	0	0	0	0	0	0	0	0	0	0	0.67	Bacteria	Firmicutes	Clostridia	Lachnospirale	Lachnospirac	Dorea
ASV_623	4.67	1.33	2.67	2.33	1.67	7	15	8	3	6	0.67	2.33	Bacteria	Actinobacter	Thermoleoph	Solirubrobact	Solirubrobact	Conexibacter
ASV_624	0.67	0	0	0	17.33	25.33	0.5	1.33	0	0	0	0.67	Archaea	Crenarchaeo	Thermoprote	Sulfolobales	Sulfolobacea	Acidianus

ASV_625	4.67	37.67	0	0.67	0.33	0	0	0	0	0	0	0	Bacteria	Actinobacter	Actinobacter	Corynebacter	Nocardiaceae	Rhodococcus
ASV_626	4.33	2.33	6.33	4.67	1	4	8	4	2	3	1.33	1	Bacteria	Actinobacter	Thermoleoph	Solirubrobact	67-14	NA
ASV_627	43	0	0	0	0	0.33	0	0.33	0	0	0.33	0	Archaea	Crenarchaeo	Thermoprote	Sulfolobales	Sulfolobacea	Stygiolobus
ASV_628	0	0	0.67	0	0	0	2	0	0	0	0	39.67	Bacteria	Actinobacter	Actinobacter	Micrococcale	Micrococcace	Kocuria
ASV_629	0.67	0	0.33	0	0.33	0	1	0	0	2	1.67	68.33	Bacteria	Proteobacter	Gammaprote	Burkholderia	Comamonad	Extensimonas
ASV_630	0	0	0.33	0	0.33	0	2.5	0	0	2	0.67	79.33	Bacteria	Firmicutes	Negativicutes	Veillonellales	Veillonellacea	Veillonella
ASV_631	0	0	0	0	0.33	0	0	4.33	0	3	47.33	0.33	Bacteria	Cyanobacteri	Cyanobacteri	Cyanobacteri	Chroococcidi	Chroococcidiopsis SAG 2023
ASV_632	27.33	0.33	6	1.67	0	0	2.5	0	0	0	0	0	Bacteria	Proteobacter	Gammaprote	Pseudomona	Pseudomona	Pseudomonas
ASV_633	2.67	0.67	1	2.33	0	1	2	3.67	0	0	2	0.33	Bacteria	Proteobacter	Gammaprote	Xanthomona	Rhodanobact	Pseudofulvimonas
ASV_634	3.67	0	0	32.67	0	0	0	1	0	0	0	0	Bacteria	Proteobacter	Alphaproteol	Rhodobacter	Rhodobacter	Paracoccus
ASV_635	45.33	0	0.67	0	0	0	1.5	1	0	0	0.67	1	Bacteria	Proteobacter	Alphaproteol	Rhizobiales	Beijerinckiace	Methylobacterium-Methylorubrum
ASV_636	0.67	0	0	0	0	0	0	0	0	0	0	0	Bacteria	Chloroflexi	Ktedonobact	Ktedonobact	Ktedonobact	NA
ASV_637	4.33	1.33	1.67	3.33	2.33	8.33	15.5	3.33	0	0	1	3.67	Bacteria	Chloroflexi	Gitt-GS-136	NA	NA	NA
ASV_638	0	0	0	0.33	3	22	2	0	0	0	0	0	Bacteria	Acidobacteri	Acidobacteria	Acidobacteria	Acidobacteria	NA
ASV_639	7	2	5.33	2	0	7.67	12	1.33	0	0	0.67	2.33	Bacteria	Actinobacter	Thermoleoph	Solirubrobact	67-14	NA
ASV_640	0	0	0	0	0	2.67	0	0	0	0	0	0	Bacteria	Firmicutes	Sulfobacillia	Sulfobacillale	Sulfobacillace	Sulfobacillus
ASV_641	0	0	0	0	1.33	0	0	0	413	0	0.67	0	Bacteria	Chloroflexi	TK10	NA	NA	NA
ASV_642	0	0.33	0	0	0	0	0	0	0	0	0	0.67	Bacteria	Proteobacter	Alphaproteol	Caulobactera	Caulobactera	PMMR1
ASV_643	1.67	3	0	2.67	2.67	12.33	36.5	0	0	2	0	0	Bacteria	Armatimonac	NA	NA	NA	NA
ASV_644	0.33	0	0	0	1	0	0	3	0	3	50.67	1	Bacteria	Actinobacter	Actinobacter	Pseudonocar	Pseudonocar	Amycolatopsis
ASV_645	1.33	8.67	2	2	0.33	0.67	3	1.67	0	4	1	0.67	Bacteria	Proteobacter	Gammaprote	Burkholderia	Oxalobactera	Massilia
ASV_646	1.33	4.67	40.67	0	0	0	3	0	0	0	1	3	Bacteria	Firmicutes	Bacilli	Staphylococc	Staphylococc	Staphylococcus
ASV_647	0	48.67	0	0	0	1	0	0	0	0	0	0.33	Bacteria	Deinococcota	Deinococci	Thermales	Thermaceae	Thermus
ASV_648	0	0	0	0	0	5	0	0	0	0	0	0	Bacteria	Firmicutes	Sulfobacillia	Sulfobacillale	Sulfobacillace	Sulfobacillus
ASV_649	1.67	37.67	0.33	0.67	0	0	0	0	0	0	1	4	Bacteria	Proteobacter	Gammaprote	Burkholderia	Burkholderia	Ralstonia
ASV_650	0	0.33	0	27.33	0	0	0	0	0	0	0	0	Bacteria	Chloroflexi	Ktedonobact	Ktedonobact	Ktedonobact	1921-2
ASV_651	8	4.67	2.67	3.33	1	1.33	14	3	0	2	1.67	4.33	Bacteria	Actinobacter	Actinobacter	Frankiales	NA	NA
ASV_652	0.33	0	0	0	0	0	0	0	0	0	0	0	Bacteria	Firmicutes	Sulfobacillia	Sulfobacillale	Sulfobacillace	NA
ASV_653	0	0	0	0	0	0	0	0	0	0	0	0	Bacteria	Actinobacter	Thermoleoph	Gaiellales	NA	NA
ASV_654	0	0	0	0	0	0	0.5	0	0	0	0	4.67	Bacteria	Actinobacter	Actinobacter	Micrococcale	Micrococcace	Micrococcus
ASV_656	0	0	0.67	0	0	0	1.5	0	0	0	1	1	Bacteria	Proteobacter	Gammaprote	Burkholderia	Comamonad	Delftia
ASV_657	0	0.33	0	0	0	0	0	0	0	0	0	0.67	Bacteria	Actinobacter	NA	NA	NA	NA
ASV_658	0	0	0	0	0	0	0	0	0	0	0	0.67	Bacteria	Actinobacter	Acidimicrobii	IMCC26256	NA	NA
ASV_659	0.67	22.67	0	0.67	0	0.67	4	12	0	0	0	0	Bacteria	Proteobacter	Gammaprote	Burkholderia	Alcaligenacea	Alcaligenes
ASV_660	0	0	0	0.67	0	0	0	0	0	0	0	0	Bacteria	Chloroflexi	Ktedonobact	Ktedonobact	Ktedonobact	NA
ASV_661	0	0	0	2	0	13.67	0	0	0	0	0	0	Bacteria	Firmicutes	Sulfobacillia	Sulfobacillale	Sulfobacillace	NA
ASV_662	3.67	1	2.33	3	4	1.33	19	2	2	2	1	1.33	Bacteria	Actinobacter	Actinobacter	Propionibact	Nocardioidac	Nocardioides
ASV_663	5.67	5.67	0.33	8	2.67	0	1	0	0	0	3.33	3.67	Bacteria	Proteobacter	Gammaprote	Diplorickettsi	Diplorickettsi	NA
ASV_664	1	0	0.67	0	1.33	0	12.5	0	0	0	79	2.33	Bacteria	Actinobacter	Actinobacter	Pseudonocar	Pseudonocar	Pseudonocardia
ASV_665	4	14.33	3.67	0	1	0	0.5	3.67	2	0	2.67	2.67	Bacteria	Firmicutes	Bacilli	Staphylococc	Staphylococc	Staphylococcus
ASV_666	25.67	1.33	0	6.33	0	0	0	0.67	0	0	0	0	Bacteria	Proteobacter	Gammaprote	Burkholderia	Comamonad	Acidovorax
ASV_667	1	0	8.33	0	0	0	0	0	0	388	0.67	0	Bacteria	Proteobacter	Gammaprote	Burkholderia	Burkholderia	Ralstonia
ASV_668	0	0.67	0	0	0	1	0	0	0	0	0	0	Bacteria	Proteobacter	Alphaproteol	Rhodospirilla	NA	NA
ASV_669	4.33	1.33	3	5	4.67	2.67	9	0	2	0	3	0	Bacteria	Actinobacter	Thermoleoph	Gaiellales	Gaiellaceae	Gaiella
ASV_670	0	0.33	0	0	0.67	12.33	0.5	0	0	0	0	0	Bacteria	Actinobacter	Acidimicrobii	Acidimicrobia	Acidimicrobia	Acidimicrobium
ASV_671	0	0	0	0	0.33	0	3	0.33	0	0	0.33	61.33	Bacteria	Proteobacter	Gammaprote	Burkholderia	Comamonad	NA
ASV_672	0	6.33	0.33	0	1.33	0	0	6	0	3	38	1.67	Bacteria	Actinobacter	Thermoleoph	Gaiellales	NA	NA
ASV_673	2.67	1	3.33	1.33	2.33	7	8.5	6	0	2	2.33	3.33	Bacteria	Acidobacteri	Blastocatellia	Blastocatellal	Blastocatellac	JGI 0001001-H03
ASV_674	0	0	0	0	0	0	0	0	0	0	57.67	1	Bacteria	Actinobacter	Actinobacter	0319-7L14	NA	NA
ASV_675	0	0	0	0	0	0	0	2	2	6	53.33	0	Bacteria	Actinobacter	NA	NA	NA	NA
ASV_676	1.33	1	0.67	1.33	2	0.33	5.5	7	0	0	0.33	2.67	Bacteria	Chloroflexi	TK10	NA	NA	NA
ASV_677	0	0	0	0	0	0	0	2.33	0	5	52	1	Bacteria	Actinobacter	Actinobacter	0319-7L14	NA	NA
ASV_678	0	0	0	0.33	1.33	0	0	0	0	0	0	0	Bacteria	Chloroflexi	Ktedonobact	Ktedonobact	Ktedonobact	NA
ASV_679	0	0	0	0	0	0	0	0.33	0	0	0	0	Bacteria	Proteobacter	Gammaprote	Burkholderia	Hydrogenoph	Hydrogenophilus
ASV_680	3	3	1.33	4.33	3.33	1	11	1	3	2	4.33	3	Bacteria	Proteobacter	Gammaprote	Xanthomona	Xanthomona	Arenimonas
ASV_682	5	1	1.67	0.67	4.67	0.67	5.5	7.33	0	0	0	1.67	Bacteria	Acidobacteri	Vicinamibact	Vicinamibact	Vicinamibact	NA
ASV_684	0.33	0	12	21	0	3.67	1.5	0	0	0	0.67	0	Bacteria	Chloroflexi	Ktedonobact	Ktedonobact	Ktedonobact	1921-2
ASV_685	0	0	0	0	0	0	0	0.33	0	0	2.67	44.67	Bacteria	Proteobacter	Gammaprote	Pseudomona	Halomonada	Halomonas
ASV_686	0	0	0	0	0.33	0	0	1.67	1	4	49.67	0.67	Bacteria	Proteobacter	Alphaproteol	Rhizobiales	Xanthobacter	NA
ASV_687	40.33	0	0	0	0	0	1	0	0	0	0.67	0	Bacteria	Deinococcota	Deinococci	Thermales	Thermaceae	Meiothermus
ASV_689	8.33	8	1.33	4.33	4.33	0	3.5	1.33	0	0	5	0.67	Bacteria	Abditibacteri	Abditibacteri	Abditibacteri	Abditibacteri	Abditibacterium
ASV_690	0	15.33	1	0	1.33	0	0	12.33	2	4	2.33	6	Bacteria	Proteobacter	Alphaproteol	NA	NA	NA
ASV_691	0.67	0	0	0	0	0	2	0.67	0	0	1.67	68.67	Bacteria	Actinobacter	Thermoleoph	Solirubrobact	67-14	NA
ASV_692	6	2	4	2.33	1	3.67	5	2	0	4	0	1	Bacteria	Chloroflexi	Chloroflexia	Thermomicro	JG30-KF-CM4	NA
ASV_693	2.33	1.33	2.33	1.67	1.33	9.33	15.5	7	2	0	1.67	2	Bacteria	Chloroflexi	Gitt-GS-136	NA	NA	NA
ASV_694	0	0.67	0	0	8	9.67	6.5	1	0	0	0.33	0	Bacteria	Firmicutes	Sulfobacillia	Sulfobacillale	Sulfobacillace	Sulfobacillus
ASV_695	4	4.33	5.67	1.33	0.33	1.67	5	2.33	0	3	1.67	1.67	Bacteria	Chloroflexi	TK10	NA	NA	NA
ASV_696	0	0	0	0	0.67	0.33	0	0.67	0	2	49.67	1.67	Bacteria	Actinobacter	Actinobacter	Corynebacter	Mycobacteria	Mycobacterium
ASV_697	2	31	0	0.67	0.33	0	0	0	0	0	0	0	Bacteria	Firmicutes	Bacilli	Staphylococc	Staphylococc	Staphylococcus
ASV_698	0	0.67	2	0.33	1.33	0.67	4	0.33	0	375	0.67	0	Bacteria	Firmicutes	Bacilli	Thermicanale	Thermicanac	Thermicanus
ASV_699	1.33	0	1.67	1	0.33	0	0	2.33	0	290	0	0	Bacteria	Firmicutes	Bacilli	Exiguobacter	Exiguobacter	Exiguobacterium
ASV_700	4.67	1.33	3.67	3.33	1	2	11	2	0	2	1	3	Bacteria	Actinobacter	Thermoleoph	Solirubrobact	67-14	NA
ASV_701	0	0	0	0	0	0	0	0.67	0	0	50.33	1	Bacteria	Actinobacter	Actinobacter	Micrococcale	Microbacteri	Agromyces
ASV_702	0.33	1.67	0	3	0	0	0	0	0	0	0	4	Bacteria	Proteobacter	Gammaprote	Xanthomona	Xanthomona	NA
ASV_703	0.67	0	0	0	0	0	0	0	0	0	0	0	Bacteria	Actinobacter	Acidimicrobii	NA	NA	NA
ASV_704	0	0	6	0	0	0	0	0.67	0	331	0	0.33	Bacteria	Proteobacter	Gammaprote	Burkholderia	Burkholderia	Ralstonia
ASV_705	1	0	0	0	0	0	15	0	0	0	82.67	0.67	Bacteria	Firmicutes	Bacilli	Bacillales	Bacillaceae	Aeribacillus
ASV_706	0	0	26.67	0	0	0	0	0	0	0	0	0	Bacteria	Proteobacter	Gammaprote	Pseudomona	Pseudomona	Pseudomonas
ASV_707	0	0	26	0	0	0	0	0	0	0	0	0	Bacteria	Proteobacter	Gammaprote	Pseudomona	Pseudomona	Pseudomonas
ASV_708	4	2	0	3.33	3	4.33	9	3.33	0	0	2.67	4.67	Bacteria	Actinobacter	Thermoleoph	Solirubrobact	67-14	NA
ASV_709	36	0	0	0	0	0.33	1.5	0	0	0	0	0.33	Bacteria	Proteobacter	Gammaprote	Burkholderia	Burkholderia	Ralstonia
ASV_710	0	0	0	0	0.33	6	0	41.67	0	0	0	0	Bacteria	Firmicutes	Sulfobacillia	Sulfobacillale	Sulfobacillace	Sulfobacillus
ASV_711	3	3	3.67	2	1.67	3.67	4.5	2	3	2	1.67	3.67	Bacteria	Actinobacter	Actinobacter	Propionibact	Nocardioidac	Marmoricola
ASV_712	0.33	0	0	1.33	0	0.67	0	0	0	0	0	0	Bacteria	RCP2-54	NA	NA	NA	NA
ASV_713	6	0.33	3.33	2	0.67	3.67	2.5	2.67	2	0	0.67	2.33	Bacteria	Actinobacter	Thermoleoph	Gaiellales	Gaiellaceae	Gaiella
ASV_714	3.67	1	2	3	2.33	6	4	4.67	2	4	1.33	1.33	Bacteria	Actinobacter	Acidimicrobii	Microtrichale	NA	NA
ASV_715	1.33	4.67	0	0	0	0	0	0	1	7	0	3.33	Bacteria	Proteobacter	Gammaprote	Burkholderia	Burkholderia	Ralstonia
ASV_717	0	0	0.33	0	0.33	1	4	0	0	0	85.67	0.33	Bacteria	Proteobacter	Gammaprote	Enterobacter	Enterobacter	NA
ASV_718	8	0.33	2	2	1.67	2.67	10.5	2	0	0	0.67	2.33	Bacteria	Gemmatimor	Longimicrobi	Longimicrobi	Longimicrobi	NA
ASV_719	0	0	0	0	0.33	0	1	0.67	0	2	57	0.33	Bacteria	Proteobacter	Gammaprote	Enterobacter	Enterobacter	Klebsiella
ASV_720	0.33	0	0	0	0	0	0	2.67	0	0	0	0	Bacteria	Proteobacter	Gammaprote	Enterobacter	NA	NA
ASV_721	25	0.33	0	0	0	0	0	0	0	0	0	9.33	Bacteria	Proteobacter	Gammaprote	Burkholderia	Comamonad	NA
ASV_722	0	0	29	0	0	0.67	0	0	0	0	0	0	Bacteria	Proteobacter	Alphaproteol	Rhizobiales	Beijerinckiace	Methylobacterium-Methylorubrum
ASV_723	0	0	0	0	1	0	0	0	0	0	0	0.67	Bacteria	Proteobacter	Alphaproteol	Rhodobacter	Rhodobacter	Rubellimicrobium
ASV_724	3.33	0	25.67	0	0	0	0	0	0	0	0	0	Bacteria	Proteobacter	Gammaprote	Pseudomona	Pseudomona	Pseudomonas
ASV_725	4.33	2.67	0	2.33	0.33	0	0.5	0	0	0	0	0	Bacteria	Proteobacter	Gammaprote	Enterobacter	Yersiniaceae	Yersinia
ASV_726	0	34.33	0.33	1.67	1	0	0	0	0	0	0	0	Archaea	Crenarchaeo	Thermoprote	Sulfolobales	Sulfolobacea	NA
ASV_727	0	0	0	0	0.67	0.67	0	0	322	0	0.67	0	Bacteria	Proteobacter	Alphaproteol	Rhizobiales	Beijerinckiace	Microvirga
ASV_728	8.33	3	0	8	0	0.67	1	0	0	0	0	0	Bacteria	Armatimonac	NA	NA	NA	NA
ASV_729	0	0	0	0	0.67	0	0	1.67	2	5	41.67	0.67	Bacteria	Actinobacter	Thermoleoph	Gaiellales	NA	NA
ASV_730	0	0	0	0	0	0	0	0	0	0	0	0.67	Bacteria	RCP2-54	NA	NA	NA	NA
ASV_732	0	0	0	0	0.67	1.33	0	0	301	5	0	0	Bacteria	Firmicutes	Bacilli	Paenibacillale	Paenibacillac	Paenibacillus
ASV_733	0	34.33	0	0	0	0.33	0	0	0	0	0	0.67	Bacteria	Firmicutes	Bacilli	Bacillales	Bacillaceae	Anoxybacillus
ASV_734	5.67	0.33	0.33	3.67	0.67	5.33	6.5	2	0	0	0.33	4.33	Bacteria	Actinobacter	Actinobacter	0319-7L14	NA	NA
ASV_735	6.67	2.33	1	4.67	5	0.33	2.5	0.33	0	0	1.67	1.33	Bacteria	Abditibacteri	Abditibacteri	Abditibacteri	Abditibacteri	Abditibacterium
ASV_736	5	1	3.67	3.67	1.33	2.67	9.5	2.67	1	5	2.33	0.67	Bacteria	Actinobacter	Rubrobacteri	Rubrobactera	Rubrobacteri	Rubrobacter
ASV_737	1	5.33	0	0	0.33	0	0.5	0	0	0	0.67	24.33	Bacteria	Firmicutes	Bacilli	Staphylococc	Staphylococc	Staphylococcus
ASV_738	0.33	0	0	0	0	0	0	0.33	0	0	0	0	Bacteria	Gemmatimor	Longimicrobi	Longimicrobi	Longimicrobi	NA
ASV_739	0.33	19	0	0	0	0	0	12.67	0	0	2.33	1.33	Bacteria	Proteobacter	Gammaprote	Pseudomona	Halomonada	Halomonas
ASV_740	2	0	25	0.33	0	0	0	0	0	0	0	0	Bacteria	Proteobacter	Gammaprote	Pseudomona	Pseudomona	Pseudomonas
ASV_741	0.33	0.33	6	0	7.33	0	9	1.33	6	0	5.33	0.67	Bacteria	Actinobacter	Actinobacter	Nitriliruptora	Nitriliruptora	NA
ASV_742	0	0	0	0	0	0.33	0	0	0	0	0	0	Archaea	Crenarchaeo	Nitrososphae	Group 1.1c	NA	NA
ASV_743	4	1.67	5.67	2	2.33	2.67	5	3	1	7	1.67	0	Bacteria	Actinobacter	Acidimicrobii	NA	NA	NA
ASV_744	0	33	0.67	0	0	0	0	1.33	0	0	1.33	0.67	Bacteria	Proteobacter	Gammaprote	Pseudomona	Halomonada	Halomonas
ASV_745	0	33.33	0	0	0	0.33	0	0	0	0	0	0	Bacteria	Bacteroidota	Bacteroidia	Flavobacteria	Weeksellacea	Moheibacter
ASV_746	0	0	0	0	0.67	0	0	1.33	0	5	38.33	0.33	Bacteria	Actinobacter	Actinobacter	Euzebyales	Euzebyaceae	NA
ASV_747	1.33	29	0	0	1.33	0	0	0	0	0	0	0	Bacteria	Proteobacter	Gammaprote	Enterobacter	Idiomarinace	Idiomarina
ASV_748	9	2.33	4.33	2.33	0.67	1	1.5	0.67	0	3	0.33	1	Bacteria	Bacteroidota	Bacteroidia	Chitinophaga	Chitinophaga	NA
ASV_749	3.33	1.67	2.67	2.67	2.33	4.67	6	2.67	0	4	1	0	Bacteria	Acidobacteri	Blastocatellia	Blastocatellal	Blastocatellac	Blastocatella
ASV_750	6.67	6.33	0	1.33	3	5	23	0	0	0	0	0	Bacteria	Proteobacter	Alphaproteol	Sphingomona	Sphingomona	Sphingomonas
ASV_751	0	0	0	0	1	0	0	2.67	0	2	41.67	0	Bacteria	NA	NA	NA	NA	NA
ASV_752	3.33	2	2.33	1.67	0.67	1.67	5	1	0	2	0	0.67	Bacteria	Actinobacter	Thermoleoph	Gaiellales	NA	NA
ASV_753	1.67	2	7.67	0.33	0.67	1.67	2.5	2	0	6	0.33	0.33	Bacteria	Patescibacter	Saccharimon	Saccharimon	NA	NA
ASV_754	1.67	0.67	6.67	1.67	2.33	1	8	0	0	11	1.67	0	Bacteria	Acidobacteri	Blastocatellia	Blastocatellal	Blastocatellac	JGI 0001001-H03
ASV_755	0	0	0.33	0	0	0	0	0	0	0	0	1	Bacteria	Actinobacter	Rubrobacteri	Rubrobactera	Rubrobacteri	Rubrobacter
ASV_757	0.67	0	0.33	0	0	0	1	0	0	0	0	0	Bacteria	Firmicutes	Bacilli	Staphylococc	Staphylococc	Staphylococcus
ASV_758	6.67	2	1	2.33	1	2.33	2.5	1.67	1	4	1	1	Bacteria	Actinobacter	Thermoleoph	Solirubrobact	67-14	NA
ASV_759	0	0	0	0	0	0	0	1	0	0	39	3	Bacteria	Proteobacter	Gammaprote	Pseudomona	Moraxellacea	Enhydrobacter
ASV_760	4.33	0.67	6.33	2	0.67	1.67	2.5	3.33	0	0	1	1.67	Bacteria	Actinobacter	Acidimicrobii	NA	NA	NA
ASV_761	4	1	2.33	3.33	1.33	5.67	6.5	2	0	0	1.33	3	Bacteria	Actinobacter	Thermoleoph	Solirubrobact	Solirubrobact	Conexibacter
ASV_762	0.67	0	35.33	0	0	0	7	0	0	0	2	1	Bacteria	Proteobacter	Gammaprote	Pseudomona	Moraxellacea	Psychrobacter
ASV_763	10	3.67	0	5.33	3.67	0.67	1	0	0	0	0	3	Bacteria	Patescibacter	Saccharimon	Saccharimon	Saccharimon	GTL1
ASV_764	0	0	0	0	0.33	0	0	0	0	0	0	0	Bacteria	Firmicutes	Negativicutes	Veillonellales	NA	NA
ASV_765	0.67	1	1	1.67	0	0	0	0.67	0	0	0	0	Archaea	Crenarchaeo	Thermoprote	Sulfolobales	Sulfolobacea	Stygiolobus
ASV_766	0	0.33	0	0	0	0	0	0	0	0	0	0	Bacteria	Chloroflexi	Ktedonobact	Ktedonobact	Ktedonobact	NA
ASV_767	28.33	0	0.33	0	0	0	0	0	0	0	0	0	Bacteria	Actinobacter	Rubrobacteri	Rubrobactera	Rubrobacteri	Rubrobacter
ASV_768	0	0	28.67	0	0	0	0	0	0	2	0	0	Bacteria	Proteobacter	Gammaprote	Pseudomona	Pseudomona	Pseudomonas
ASV_769	0	1	0	0	0	0	0	47.67	0	0	0	0	Bacteria	Bacteroidota	Bacteroidia	Chitinophaga	Chitinophaga	NA
ASV_770	0	0.33	0	0	0.33	3.33	0	0	0	0	0	0	Bacteria	Actinobacter	Acidimicrobii	IMCC26256	NA	NA
ASV_771	0	0	0	0	0.33	0.33	0	0	286	0	0.33	0	Bacteria	Chloroflexi	Chloroflexia	Kallotenuales	AKIW781	NA
ASV_772	3	5	0.67	20.33	0.67	0	0	0	0	0	0	0	Archaea	Crenarchaeo	Thermoprote	Sulfolobales	Sulfolobacea	Stygiolobus
ASV_773	0	0	0	0	0.33	0	0	1.67	0	2	38.33	0.33	Bacteria	Actinobacter	Thermoleoph	Gaiellales	NA	NA
ASV_774	0	36.67	0	0	0	0.67	0	0	0	0	0	0.67	Archaea	Crenarchaeo	Nitrososphae	Nitrososphae	Nitrososphae	NA
ASV_775	0	0	0	0	1	16.67	1	5.33	0	0	0.33	0	Bacteria	Actinobacter	Acidimicrobii	NA	NA	NA
ASV_776	0	0	24.33	0	0	0	3	0	0	3	1	1.33	Bacteria	Bacteroidota	Bacteroidia	Flavobacteria	Weeksellacea	NA
ASV_777	0.33	0.67	3.67	0	7.33	0	8.5	0	8	0	4.67	0.33	Bacteria	Proteobacter	Gammaprote	Burkholderia	TRA3-20	NA
ASV_778	1.33	0	0.33	0.67	0	8	30	1.33	0	0	1.67	0.67	Bacteria	Acidobacteri	Blastocatellia	Pyrinomonad	Pyrinomonad	RB41

ASV_779	0	0	0	0	1	30	1	0	2	0	0	0	Bacteria	Firmicutes	Sulfobacillia	Sulfobacillale	Sulfobacillace	Sulfobacillus
ASV_780	0	1	0	0	0	1.33	0	0	0	4	0.67	0	Bacteria	Actinobacter	Actinobacter	Pseudonocar	Pseudonocar	Kibdelosporangium
ASV_781	25.67	0	0	0	0	0	0	0	0	0	0	0	Bacteria	Chloroflexi	Chloroflexia	Thermobacul	Thermobacul	Thermobaculum
ASV_782	3.67	0.67	1	0.67	1	0.33	1.5	8	0	0	0	1.67	Bacteria	Verrucomicr	Verrucomicr	Pedosphaera	Pedosphaera	NA
ASV_783	0.67	0	0.33	0	0	0.67	6.5	0	0	0	72.33	1.33	Bacteria	Proteobacter	Gammaprote	Burkholderia	Comamonada	NA
ASV_784	1	0.33	4.67	3.67	2	2.33	6.5	1	0	4	1.33	0.67	Bacteria	Chloroflexi	KD4-96	NA	NA	NA
ASV_785	3.67	0	28.33	0.33	0	0	3	0	0	0	0.33	0.33	Bacteria	Proteobacter	Gammaprote	Pseudomona	Pseudomona	Pseudomonas
ASV_786	0.33	0.33	0.33	0	0	0	0.5	0	0	0	0	29.67	Bacteria	Actinobacter	MB-A2-108	NA	NA	NA
ASV_787	4.67	1.67	4.67	0.67	2	2	3.5	1.67	0	2	1	0.33	Bacteria	Gemmatimor	Gemmatimor	Gemmatimor	Gemmatimor	NA
ASV_788	3	2.33	0.67	4	0	3.33	1	2.67	0	0	1	3.33	Bacteria	Actinobacter	Actinobacter	0319-7L14	NA	NA
ASV_789	5.33	3.67	0.33	0.33	1.33	1.67	0.5	5.33	4	0	0	1.33	Bacteria	Chloroflexi	P2-11E	NA	NA	NA
ASV_790	0	0.33	0	0	0	0	0	0	0	0	0.33	36.33	Bacteria	Proteobacter	Gammaprote	Pseudomona	Moraxellacea	Acinetobacter
ASV_791	0	0	0	0	0	0	0	1.33	0	2	42.33	0	Bacteria	Firmicutes	Bacilli	Staphylococc	Staphylococc	Staphylococcus
ASV_792	0	0	0	0	0	0	0	0	0	0	0	0	Bacteria	Proteobacter	Alphaproteol	NA	NA	NA
ASV_793	0	0	0	0	0	0	0	0	0	0	0	38.33	Bacteria	Actinobacter	Actinobacter	Micrococcale	Dermacoccac	NA
ASV_794	0	0	0	0	4	14.67	0	0	0	0	0	0.33	Bacteria	Firmicutes	Sulfobacillia	Sulfobacillale	Sulfobacillace	Sulfobacillus
ASV_795	0	1.67	1.33	2.33	1.67	1.33	4	2.33	0	0	0.33	4	Bacteria	Actinobacter	Actinobacter	0319-7L14	NA	NA
ASV_796	2	10	3	0	0.67	0	0	1.33	3	0	1.33	0.33	Bacteria	Proteobacter	Alphaproteol	Rhodobacter	Rhodobacter	Paracoccus
ASV_797	0	0.33	0	0.33	3	13.67	0	28.33	0	0	0	0.67	Bacteria	Firmicutes	Sulfobacillia	Sulfobacillale	Sulfobacillace	Sulfobacillus
ASV_798	21	0	0	0.33	0	0	0	0	0	0	0	0	Archaea	Crenarchaeo	Thermoprote	Sulfolobales	Sulfolobacea	NA
ASV_799	1	0	25.67	0	0	0	6	0	0	0	1.33	1	Bacteria	Proteobacter	Alphaproteol	Caulobactera	Hyphomonac	SWB02
ASV_800	0.33	0	0	0	0	0	0	45.67	0	0	0	0	Bacteria	Actinobacter	NA	NA	NA	NA
ASV_802	0.67	0	0	1	1.33	6.67	0	0	0	0	0	0	Bacteria	Actinobacter	Thermoleoph	Solirubrobact	Solirubrobact	NA
ASV_803	0	0.33	3	0	2	0.33	6	1.67	6	0	15.33	5.33	Bacteria	Actinobacter	Actinobacter	Micrococcale	Dermacoccac	Dermacoccus
ASV_804	1.33	2	0.67	4.67	0.67	2.67	10.5	2	0	0	1	1.67	Bacteria	Actinobacter	Actinobacter	0319-7L14	NA	NA
ASV_805	1.33	0.33	2.33	2	0.67	0.33	2.5	3.33	0	0	0	1	Bacteria	Chloroflexi	NA	NA	NA	NA
ASV_806	4.33	0.33	2.33	1.33	1	4	6.5	1.67	0	0	1	0	Bacteria	Actinobacter	Thermoleoph	Gaiellales	NA	NA
ASV_807	1.67	1	1	4	2.67	6	3.5	1.67	0	0	1	2	Bacteria	Actinobacter	Thermoleoph	Solirubrobact	67-14	NA
ASV_808	2	0.33	0	1.67	1.33	7.67	6.5	3.33	0	0	1.33	2	Bacteria	Proteobacter	Alphaproteol	Sphingomona	Sphingomona	Sphingomonas
ASV_809	0	29.67	0	0	0	0	0	0	0	2	0	0	Bacteria	Proteobacter	Alphaproteol	Rhizobiales	Rhizobiaceae	NA
ASV_810	0	0	0	0	0	0	0	0	0	0	0	1.33	Bacteria	Actinobacter	Actinobacter	0319-7L14	NA	NA
ASV_811	2.67	3.33	1.67	3.33	2	1	2.5	2	0	0	2.67	0.33	Bacteria	Actinobacter	Actinobacter	Propionibact	Nocardioidac	Marmoricola
ASV_812	0	0	0.33	0	0	0.33	1.5	0	0	0	0	27.67	Bacteria	Firmicutes	Bacilli	Staphylococc	Staphylococc	Nosocomiicoccus
ASV_813	6.33	6	0.33	3.67	3	0	0	0	0	0	0	2.33	Bacteria	Proteobacter	Alphaproteol	Sphingomona	Sphingomona	NA
ASV_814	6	0	0.33	0	1	1.33	1.5	5	0	0	0.67	0.33	Bacteria	Chloroflexi	TK10	NA	NA	NA
ASV_815	1	25	0	0.33	1.67	0	0	0	0	0	0	0	Archaea	Halobacterot	Methanosarc	Methanosarc	Methanosarc	Methanosarcina
ASV_816	0	0	0	0	0	0	0	1.33	0	5	39.67	0.33	Bacteria	Proteobacter	Gammaprote	Pseudomona	Halomonada	Chromohalobacter
ASV_817	0	0.33	0	0	0	0	0	0	0	0	0	0	Bacteria	Proteobacter	Gammaprote	Pseudomona	Moraxellacea	Acinetobacter
ASV_818	0	27.67	0	0	0	1	0	0	0	0	0	0.33	Bacteria	Actinobacter	Actinobacter	Corynebacter	Corynebacter	Corynebacterium
ASV_819	1	0.33	1.33	0.33	0.33	1.33	13.5	3.33	0	0	0.67	5.33	Bacteria	Actinobacter	Actinobacter	Propionibact	Nocardioidac	Nocardioides
ASV_820	0	0	0	0	0	0	0	0	261	0	0	0	Bacteria	Proteobacter	Gammaprote	Pseudomona	Pseudomona	Pseudomonas
ASV_821	0	0	0	0	0.33	0	0	1	1	4	37	0	Bacteria	Firmicutes	Bacilli	Staphylococc	Staphylococc	Jeotgalicoccus
ASV_822	0	0	0	0	1.33	10.33	0	0	0	0	0	0	Bacteria	Firmicutes	Sulfobacillia	Sulfobacillale	Sulfobacillace	Sulfobacillus
ASV_823	20.67	0	0	0.33	0	0	0	0	0	0	0	0	Bacteria	Actinobacter	Thermoleoph	Gaiellales	NA	NA
ASV_824	0.67	0	25	0	0	0	0	0	0	0	0	0	Bacteria	Proteobacter	Gammaprote	Pseudomona	Pseudomona	Pseudomonas
ASV_825	0	11.33	0.67	0	1.67	0	0	4	2	0	1.33	0.67	Bacteria	Proteobacter	Alphaproteol	Rhodobacter	Rhodobacter	Paracoccus
ASV_826	0.33	0	0	0.33	3.33	1.33	0	0	0	0	0	0.33	Bacteria	Actinobacter	Actinobacter	Frankiales	Acidotherma	Acidothermus
ASV_827	0.33	0	0	0	0	0	1	0	253	0	0.33	0	Bacteria	Bacteroidota	Bacteroidia	Flavobacteria	Weeksellacea	Cloacibacterium
ASV_828	20.33	0	0	0	0	0	0	0	0	0	0	0	Bacteria	Proteobacter	Gammaprote	Pseudomona	Pseudomona	Pseudomonas
ASV_829	0	0.33	0	0	0	0	0	0	0	0	0.33	0	Bacteria	Proteobacter	Alphaproteol	Rhizobiales	Xanthobacter	NA
ASV_830	0	0	0	0	0.67	0	0	0	254	0	0	0	Bacteria	Gemmatimor	Longimicrobi	Longimicrobi	Longimicrobi	NA
ASV_831	0	0	32.67	0	0	0	2	0	0	0	1	0	Bacteria	Proteobacter	Alphaproteol	Rhizobiales	Rhizobiaceae	NA
ASV_832	0	6	0.33	0	0.67	0	0	2	3	0	23.67	1.67	Bacteria	Proteobacter	Gammaprote	Pseudomona	Halomonada	Chromohalobacter
ASV_833	0	0	0	0	0	0	0	0	0	0	0	0	Bacteria	Firmicutes	Sulfobacillia	Sulfobacillale	Sulfobacillace	Sulfobacillus
ASV_834	23.67	0.33	0	0	0	0	0	0	0	0	1	0	Bacteria	Deinococcota	Deinococci	Thermales	Thermaceae	Meiothermus
ASV_835	0	0	0	0	0	0.33	0	0	0	0	0	0	Bacteria	Chloroflexi	Ktedonobact	Ktedonobact	Ktedonobact	NA
ASV_836	0	0.67	0	0	0.67	7	1	0	2	0	0	0	Bacteria	Actinobacter	Acidimicrobii	NA	NA	NA
ASV_837	0	0	0	0	0	0	0	0.33	0	0	33	1	Bacteria	Proteobacter	Gammaprote	Burkholderia	Comamonada	NA
ASV_838	0	0	0	0	0	0	1.5	0.33	0	0	0.67	32	Bacteria	Proteobacter	Gammaprote	Burkholderia	Comamonada	Comamonas
ASV_839	4.67	3	0	2.67	1.33	3.33	19.5	0	0	0	0.33	0.67	Bacteria	Acidobacteri	Blastocatellia	Blastocatellal	Blastocatellac	Blastocatella
ASV_840	1	0.33	4.67	0	0.67	0.67	3.5	0.33	0	8	0.67	0.67	Bacteria	Verrucomicr	Verrucomicr	Chthoniobact	Chthoniobact	Chthoniobacter
ASV_841	0.67	0.67	0	0	0	0	0	0	0	0	0	0	Bacteria	Deinococcota	Deinococci	Thermales	Thermaceae	Meiothermus
ASV_842	0	0	0	0	14.67	22	0.5	0.67	2	0	0	0.33	Bacteria	Firmicutes	Sulfobacillia	Sulfobacillale	Sulfobacillace	Sulfobacillus
ASV_843	2	1	1.33	5.33	1.33	1	3.5	0.67	0	11	2	0	Bacteria	Planctomyce	Phycisphaera	Tepidisphaer	WD2101 soil	NA
ASV_844	0.33	0	0	0	0	0	0	0.33	0	0	32.67	1	Bacteria	Actinobacter	Actinobacter	Euzebyales	Euzebyaceae	NA
ASV_845	0	0	0.33	0	0	0	0	0	0	0	0	0	Bacteria	Armatimonac	Chthonomon	Chthonomon	Chthonomon	Chthonomonas
ASV_846	0	0	0	0	2	5.67	0	0	0	0	0	0.33	Bacteria	Actinobacter	Acidimicrobii	NA	NA	NA
ASV_847	0	0	0	0	1	0	0	1.67	0	2	33.33	0.33	Bacteria	Gemmatimor	Longimicrobi	Longimicrobi	Longimicrobi	NA
ASV_848	0	0	0	0	0	0	0	0	232	0	1.33	0	Bacteria	Actinobacter	Thermoleoph	Gaiellales	NA	NA
ASV_849	3.67	0.67	3.67	0.33	0.33	0.67	3.5	0.67	0	0	0	0.33	Bacteria	Actinobacter	Thermoleoph	Gaiellales	Gaiellaceae	Gaiella
ASV_850	0	0	24.33	0	0	0	7	0	0	0	1	1	Bacteria	Gemmatimor	Gemmatimor	Gemmatimor	Gemmatimor	NA
ASV_851	0.33	0	0	0	0	0	0	0	0	0	0	0	Bacteria	Deinococcota	Deinococci	Thermales	Thermaceae	Meiothermus
ASV_852	0	0	0	0	0	0	0	0	0	0	0	0.33	Bacteria	Proteobacter	Gammaprote	Burkholderia	Oxalobactera	Undibacterium
ASV_853	3.33	0.67	2.67	1.33	1.67	0.67	3	1.33	0	0	1	2.67	Bacteria	Proteobacter	Alphaproteol	Rhizobiales	Xanthobacter	Pseudorhodoplanes
ASV_854	0	0	3.67	0	7.67	0	7.5	0	6	0	3.67	0	Bacteria	Gemmatimor	Longimicrobi	Longimicrobi	Longimicrobi	YC-ZSS-LKJ147
ASV_855	0	0	0	0	3	6.67	0	0	0	0	0	0	Bacteria	Actinobacter	Actinobacter	Frankiales	Acidotherma	Acidothermus
ASV_856	2.33	1	0	1.67	2	4.67	21.5	0	0	0	0	0	Bacteria	Firmicutes	Negativicutes	Veillonellales	Veillonellacea	Dialister
ASV_857	4	3.33	0	2.33	2	0	1.5	0	0	0	0.33	1	Bacteria	Proteobacter	Gammaprote	Burkholderia	SC-I-84	NA
ASV_858	6	0	2.33	1	0	0.67	1	1	0	0	0	0.33	Bacteria	Patescibacter	NA	NA	NA	NA
ASV_859	1.33	0.33	1	0.67	0.67	1.67	6	3.67	0	0	1	3	Bacteria	Patescibacter	Saccharimona	Saccharimona	NA	NA
ASV_860	0	0	0	26.33	0	0	0	0	0	0	0	0	Archaea	Crenarchaeo	Nitrososphae	Nitrososphae	Nitrososphae	Candidatus Nitrocosmicus
ASV_861	0.67	0.33	0.33	1.67	4	5	8.5	6	0	4	3	2	Bacteria	Acidobacteri	Vicinamibact	Vicinamibact	Vicinamibact	Luteitalea
ASV_862	0	0	0	0	0	2	0	0	0	0	0	0	Bacteria	Firmicutes	Sulfobacillia	Sulfobacillale	Sulfobacillace	Sulfobacillus
ASV_863	0	0	0	0	0	0	0	0	0	0	0	0	Bacteria	Actinobacter	Actinobacter	Euzebyales	Euzebyaceae	NA
ASV_864	4.67	0	4	1.67	0	1	0.5	2	0	0	0	3.33	Bacteria	Chloroflexi	Gitt-GS-136	NA	NA	NA
ASV_865	0	1.33	1.33	8.67	0.67	0	0	0	0	0	0	2	Bacteria	Proteobacter	Alphaproteol	Sphingomona	Sphingomona	Sphingomonas
ASV_866	0	0	0	0	0.33	0	0	1	0	5	33	0	Bacteria	Chloroflexi	Chloroflexia	Kallotenuales	Kallotenuace	Kallotenue
ASV_868	0	0	0	0.33	0	0.33	0	0	0	0	0	0	Bacteria	Actinobacter	Actinobacter	NA	NA	NA
ASV_869	4	0.67	1.67	2	1	0.67	3	2.67	1	2	1.33	1.67	Bacteria	Actinobacter	Acidimicrobii	Microtrichale	Ilumatobacte	NA
ASV_870	0	0	0	0	0	0	0.5	0	0	0	0	21	Bacteria	Actinobacter	Actinobacter	Micrococcale	Micrococcace	Micrococcus
ASV_871	0	0	0	0	0	0	0	0	0	0	0	0	Archaea	Crenarchaeo	Nitrososphae	NA	NA	NA
ASV_872	1.33	0.67	0.33	3	2	4.33	4.5	3	0	0	0.67	3	Bacteria	Actinobacter	Thermoleoph	Solirubrobact	Solirubrobact	Solirubrobacter
ASV_873	0	0	0	0	0.67	0	0	2.67	0	0	31.67	0	Bacteria	Actinobacter	Actinobacter	Pseudonocar	Pseudonocar	Amycolatopsis
ASV_874	0	0	0	0	1.33	0.33	11.5	21.33	0	0	0	0.67	Bacteria	Actinobacter	Actinobacter	Corynebacter	Mycobacteria	Mycobacterium
ASV_875	0.67	0.33	2.33	3	1.33	3.33	3.5	3.33	0	0	1.33	1	Bacteria	Myxococcota	Polyangia	Polyangiales	Polyangiacea	Pajaroellobacter
ASV_876	22.33	0	0	0.33	0	0	0	0	0	0	0	0	Bacteria	Deinococcota	Deinococci	Thermales	Thermaceae	Meiothermus
ASV_877	0	0	0	0	0	0	0	0	0	0	0	0.33	Bacteria	Proteobacter	Gammaprote	NA	NA	NA
ASV_878	1.33	0.67	1	2.67	0.67	1.33	4	2.33	0	2	1	1.67	Bacteria	Actinobacter	Actinobacter	Propionibact	Nocardioidac	NA
ASV_879	0	0	14.67	0	0	0	0	0	0	0	0	0	Bacteria	Proteobacter	Alphaproteol	Rhizobiales	Beijerinckiace	Methylobacterium-Methylorubrum
ASV_880	4.67	0.67	2.67	2	1	2.33	2.5	1.33	0	0	0.33	1.33	Bacteria	Actinobacter	Thermoleoph	Solirubrobact	Solirubrobact	NA
ASV_881	22	0.33	0	0	0	0	0	0	0	0	0.33	0	Bacteria	Deinococcota	Deinococci	Thermales	Thermaceae	Meiothermus
ASV_882	0	0.33	0	0	0.33	0	0	2	0	5	25.67	0.67	Bacteria	Actinobacter	MB-A2-108	NA	NA	NA
ASV_883	0	11	2.67	0	1	0.33	1.5	5	0	0	0.33	3	Bacteria	Abditibacteri	Abditibacteri	Abditibacteri	Abditibacteri	Abditibacterium
ASV_884	0	0	0	0	0	0	2	0	0	0	0	41.33	Bacteria	Actinobacter	Actinobacter	Propionibact	Propionibact	NA
ASV_885	0	0	7.33	0	0	0	0	0	0	202	0	0	Bacteria	Proteobacter	Gammaprote	Burkholderia	Burkholderia	Ralstonia
ASV_886	0.33	0	0	0	0	0	0	1	0	0	0	0	Bacteria	Proteobacter	Gammaprote	Burkholderia	Comamonada	[Polyangium] brachysporum group
ASV_887	0	23	0	0	0	0	0	0	0	0	0	0.33	Bacteria	Deinococcota	Deinococci	Deinococcale	Deinococcace	Deinococcus
ASV_888	0.67	0.33	1	1	1.67	4	3	3	0	0	0.67	1.67	Bacteria	Actinobacter	Thermoleoph	Solirubrobact	67-14	NA
ASV_889	0	0	0	0	4	11	2	0.33	0	0	0	0	Bacteria	Firmicutes	Sulfobacillia	Sulfobacillale	Sulfobacillace	Sulfobacillus
ASV_890	0.67	0	20.67	0	0	0	1.5	0	0	0	1.33	0.33	Bacteria	Proteobacter	Alphaproteol	Rhizobiales	Rhizobiaceae	NA
ASV_891	0	0.33	0	0	1.33	4.33	27.5	0	3	0	2.33	0.33	Bacteria	Proteobacter	Gammaprote	Pseudomona	Moraxellacea	Acinetobacter
ASV_892	0	0	0	0	1.67	10.33	3.5	0.33	2	0	0	0	Bacteria	Actinobacter	Acidimicrobii	Acidimicrobia	Acidimicrobia	Aciditerrimonas
ASV_893	17	0	0	0	0	0	2	0	0	0	0	0	Bacteria	Proteobacter	Alphaproteol	Sphingomona	Sphingomona	Sphingomonas
ASV_894	2	0.33	1.33	3	1	3.67	5	2	0	3	1	1	Bacteria	Actinobacter	Thermoleoph	Solirubrobact	Solirubrobact	Solirubrobacter
ASV_895	0	0	2.67	0.33	2.67	0	7.5	0.67	8	0	6	0	Bacteria	Actinobacter	Thermoleoph	Solirubrobact	67-14	NA
ASV_896	1.33	0	0.67	2.33	1.33	0.67	7.5	1	0	0	0.67	1	Bacteria	Chloroflexi	TK10	NA	NA	NA
ASV_897	1.67	1	3.33	2	1.33	1	3.5	1	0	5	0	2.33	Bacteria	Chloroflexi	Gitt-GS-136	NA	NA	NA
ASV_898	4	0.33	4.67	0	0.33	0	0.5	0	2	0	3.67	0.33	Bacteria	Actinobacter	Rubrobacteri	Rubrobactera	Rubrobacteri	Rubrobacter
ASV_899	20.67	0	0.33	0	0	0	1	0.33	0	0	0	0	Bacteria	Proteobacter	Alphaproteol	Rhizobiales	Beijerinckiace	Methylobacterium-Methylorubrum
ASV_900	0	0	0.33	0	9.33	21	0.5	0	0	0	0	0.33	Bacteria	Firmicutes	Sulfobacillia	Sulfobacillale	Sulfobacillace	NA
ASV_901	0	0	0	0	0	0.67	0.5	0	214	0	0.33	0	Bacteria	Firmicutes	Bacilli	Lactobacillale	Streptococca	Streptococcus
ASV_902	0.33	0	0	0	0.67	0	0	1.33	0	0	27.33	0	Bacteria	Actinobacter	Thermoleoph	Solirubrobact	67-14	NA
ASV_903	0.33	0	0	0	1.33	1.33	0	0	0	0	0	0	Archaea	Thermoplasm	Thermoplasm	BSLdp215	NA	NA
ASV_904	0	0.33	0	0	0	0.33	0	0	0	0	0	0	Bacteria	Firmicutes	Sulfobacillia	Sulfobacillale	Sulfobacillace	Sulfobacillus
ASV_905	0.33	0	0	0	0.67	8.33	0	0	0	0	0	0.33	Bacteria	Verrucomicr	Verrucomicr	Methylacidip	Methylacidip	Candidatus Methylacidiphilum
ASV_906	1.67	0	2.33	0	0	0	0	0	0	0	16.33	0.33	Bacteria	Firmicutes	Bacilli	Bacillales	Bacillaceae	NA
ASV_907	2	0.67	1.67	0.33	0	1.67	3.5	0.67	0	0	1	1.67	Bacteria	Actinobacter	Acidimicrobii	NA	NA	NA
ASV_908	1	0	0	1.33	0	0	0	0	0	0	0	0	Bacteria	Patescibacter	Saccharimona	Saccharimona	NA	NA
ASV_909	0	0	0	0	0	0	0	0	0	0	0	0	Bacteria	Acidobacteri	Holophagae	Subgroup 7	NA	NA
ASV_910	0	0	0	0	5	12	0	0	0	0	0	0.33	Bacteria	Actinobacter	Acidimicrobii	IMCC26256	NA	NA
ASV_911	0	0	0	0	14.33	11	0	0	0	0	0	0	Archaea	Thermoplasm	Thermoplasm	BSLdp215	NA	NA
ASV_913	0	0	4.67	0	3.33	0	9	0.33	4	0	3	0	Bacteria	Proteobacter	Alphaproteol	Azospirillales	Azospirillacea	NA
ASV_914	3	0	0	1.33	1.67	2	8.5	3.33	0	0	1	1.33	Bacteria	Proteobacter	Alphaproteol	Sphingomona	Sphingomona	Sphingomonas
ASV_916	3.67	0	2.33	1.33	2.33	0.67	4	1.67	0	10	0	0	Bacteria	Actinobacter	Acidimicrobii	Microtrichale	Ilumatobacte	NA
ASV_917	0	12	0.67	0	1	0	0	7.33	3	0	0.33	4	Bacteria	Actinobacter	Actinobacter	Streptomyce	Streptomyce	Streptomyces
ASV_918	0	0	0	0.67	0	0	0	0	0	0	0	0	Bacteria	Firmicutes	Sulfobacillia	Sulfobacillale	Sulfobacillace	NA
ASV_919	5.33	0	4.33	0	0.33	0	0	0.33	4	0	3	0.33	Bacteria	Actinobacter	Rubrobacteri	Rubrobactera	Rubrobacteri	Rubrobacter
ASV_920	3	0.33	0.33	2.33	1.33	6.33	14.5	0	0	0	0.67	0	Bacteria	Actinobacter	Actinobacter	0319-7L14	NA	NA
ASV_921	0	0	0	0	0	0	0	31.33	0	0	0	0	Bacteria	Proteobacter	Gammaprote	Burkholderia	Comamonada	NA
ASV_923	0.33	0	0	0	0	0	0.5	0	0	3	0	37	Bacteria	Actinobacter	Actinobacter	Corynebacter	Corynebacter	Corynebacterium
ASV_924	9	0	5.33	1	0	0	0	0.67	0	0	0	0	Bacteria	Proteobacter	Gammaprote	Pseudomona	Pseudomona	Pseudomonas
ASV_925	0	0	0	0	0	0	0	0	0	0	0.33	24.67	Bacteria	RCP2-54	NA	NA	NA	NA
ASV_926	0	0	0	0	0.33	0.33	0	0	188	0	0	0	Bacteria	Actinobacter	Thermoleoph	Solirubrobact	Solirubrobact	Solirubrobacter
ASV_927	0	0	0	0	0	0	1	0.33	0	0	0	19	Bacteria	Firmicutes	Bacilli	Caldalkalibaci	Caldalkalibaci	Caldalkalibacillus
ASV_928	0	0	0	0	0	0	0	0	0	0	0	0	Bacteria	Chloroflexi	Chloroflexia	Kallotenuales	AKIW781	NA
ASV_929	0	23.67	0	0	0	0.33	0	0	0	0	0	0	Bacteria	Proteobacter	Gammaprote	Burkholderia	Hydrogenoph	Tepidiphilus
ASV_930	0.33	0	22	0	0	0.67	2.5	0	0	0	0.67	0.67	Bacteria	Proteobacter	Gammaprote	Xanthomona	Xanthomona	SN8

ASV_931	0.33	0.67	6.67	1.33	0	0	1.5	0.33	0	5	0	0	Bacteria	Patescibacter	Saccharimon	Saccharimon	Saccharimon	TM7a
ASV_932	0	0	0	18.33	0	0	0	0	0	0	0	0	Bacteria	Proteobacter	Alphaproteol	Sphingomona	Sphingomona	Porphyrobacter
ASV_933	0	0.33	4.67	0	1.67	0.33	5.5	0	1	0	2	0	Bacteria	Bacteroidota	Bacteroidia	Chitinophaga	Chitinophaga	NA
ASV_934	0	0	0	0	0	0	0	29.67	0	0	0	0	Bacteria	Actinobacter	Thermoleopł	Gaiellales	NA	NA
ASV_935	0	0	2.33	0	0	0	0	0.67	0	225	0	0	Bacteria	Bacteroidota	Bacteroidia	Chitinophaga	Chitinophaga	NA
ASV_936	0	0	0	0	4.67	1	12.5	12.67	0	0	0	0	Bacteria	Actinobacter	Actinobacter	Corynebacter	Mycobacteria	Mycobacterium
ASV_937	0.33	0	0	0	0	0	0.5	0	0	0	0	18	Bacteria	Actinobacter	Actinobacter	Corynebacter	Corynebacter	Lawsonella
ASV_938	2	0	4	0	3.33	0	0	0	2	0	4.33	0	Bacteria	Actinobacter	Rubrobacteri	Rubrobactera	Rubrobacteri	Rubrobacter
ASV_939	0	0	0	0	0	0	1	0	0	0	0	20.67	Bacteria	Firmicutes	Bacilli	Lactobacillale	Lactobacillace	Weissella
ASV_940	0	0	0	0	1.33	14	0.5	0	0	0	0	0	Bacteria	Firmicutes	Sulfobacillia	Sulfobacillale	Sulfobacillace	Sulfobacillus
ASV_941	0	0	0	0	4.67	22.33	0.5	0	0	0	0	0	Bacteria	Firmicutes	Sulfobacillia	Sulfobacillale	Sulfobacillace	Sulfobacillus
ASV_942	0	0.33	0	0.33	0	0	0	0	0	0	0	0	Bacteria	Chloroflexi	Ktedonobact	Ktedonobact	Ktedonobact	NA
ASV_943	0	1	0	0	0	0.67	0	0.33	0	0	1.33	0	Bacteria	Actinobacter	Thermoleopł	Solirubrobact	67-14	NA
ASV_944	5	2	0	1.33	1	0	0.5	0	0	0	0	3	Bacteria	Proteobacter	Gammaprote	Diplorickettsi	Diplorickettsi	NA
ASV_945	2.33	0.67	3.67	1.33	0	1.33	2	1	0	0	1.33	2	Bacteria	Actinobacter	Thermoleopł	Solirubrobact	67-14	NA
ASV_946	1	2.33	0.67	2	0.67	2.33	1.5	2	2	0	0.33	0.67	Bacteria	Chloroflexi	Chloroflexia	Thermobacul	Thermobacul	Thermobaculum
ASV_947	0	17.67	0	0	0	0	0	0.33	0	0	0	2	Bacteria	Proteobacter	Gammaprote	Pseudomona	Halomonada	Halomonas
ASV_948	0.33	0.33	0	0	0	0	0.5	0	0	0	0	19	Bacteria	Actinobacter	Actinobacter	Micrococcale	Micrococcace	Micrococcus
ASV_949	0	0	0	0	0	0	0	0	0	0	0	0	Bacteria	Firmicutes	Bacilli	Staphylococc	Staphylococc	Staphylococcus
ASV_950	2.67	2	0	2.33	0.67	1.67	3	2	2	0	1.33	2	Bacteria	Proteobacter	Alphaproteol	Sphingomona	Sphingomona	Sphingomonas
ASV_951	0	0	0	0	0	0	0	0	0	0	0	25.67	Bacteria	Proteobacter	Gammaprote	Burkholderia	Comamonada	Comamonas
ASV_952	0	0	0	0	0.33	0	0	0.67	0	2	27.33	0.33	Bacteria	Actinobacter	Actinobacter	Pseudonocar	Pseudonocar	Amycolatopsis
ASV_953	0	0	0	0	0.67	0	0	0	176	0	0	0	Bacteria	Proteobacter	Gammaprote	Pseudomona	Pseudomona	Pseudomonas
ASV_954	0	0	0	0	0	15.67	0	0	0	0	0	0	Bacteria	Firmicutes	Sulfobacillia	Sulfobacillale	Sulfobacillace	Sulfobacillus
ASV_955	0	0	0	0	0	0.33	0	0.33	3	0	0	0	Archaea	Crenarchaeo	Nitrososphae	Group 1.1c	NA	NA
ASV_956	0	8.33	0.67	0	1	0	0	6	4	0	0.67	5	Bacteria	Chloroflexi	Chloroflexia	Chloroflexale	Roseiflexacea	NA
ASV_957	5.67	0	0.33	2	1	1.33	3.5	2.67	0	0	1	2.33	Bacteria	Actinobacter	Thermoleopł	Solirubrobact	67-14	NA
ASV_958	0.67	0	0.33	0	0	0	1.5	0	0	0	18	22	Bacteria	Proteobacter	Gammaprote	Burkholderia	Comamonada	NA
ASV_959	2.33	2	2	3	1	2	1.5	0.33	0	5	0.67	0.67	Bacteria	Actinobacter	Thermoleopł	Solirubrobact	67-14	NA
ASV_961	0	0.33	0.33	0	0	0.33	0	0	0	0	0	0	Bacteria	Firmicutes	Sulfobacillia	Sulfobacillale	Sulfobacillace	NA
ASV_962	0	0	3.33	0.33	2.67	0	7.5	0.33	3	0	3	0.33	Bacteria	Actinobacter	Actinobacter	Euzebyales	Euzebyaceae	NA
ASV_963	1	0	0	0	0	0	5	0	0	0	0	28	Bacteria	Planctomyce	Planctomyce	Pirellulales	Pirellulaceae	Pir4 lineage
ASV_964	0	0	2.67	0	2	0	5.5	0	4	2	2	0.33	Bacteria	Actinobacter	Acidimicrobii	NA	NA	NA
ASV_965	0	0	0	0	0	0	0	0	0	0	0	0	Bacteria	Proteobacter	Gammaprote	Pseudomona	Moraxellacea	Acinetobacter
ASV_966	0	0	2.67	0	4	0	9.5	0	5	0	3	0	Bacteria	Gemmatimor	Longimicrobi	Longimicrobi	Longimicrobi	NA
ASV_967	0	0.33	0	0	1	0	0	0	0	0	0	0	Bacteria	Actinobacter	Acidimicrobii	Acidimicrobia	Acidimicrobia	Ferrimicrobium
ASV_968	0	0	0	0.33	2	1	6	17	0	0	0.33	0.33	Bacteria	Actinobacter	Actinobacter	Corynebacter	Mycobacteria	Mycobacterium
ASV_969	4.67	0.67	0.33	2	1.67	1.33	2	0.67	0	0	0.33	1.67	Bacteria	Actinobacter	Actinobacter	Frankiales	NA	NA
ASV_970	1.33	0.33	0.33	1.33	0.67	2.67	9	3	0	0	0.33	0	Bacteria	Actinobacter	Acidimicrobii	IMCC26256	NA	NA
ASV_972	3.33	0	0.67	0.67	0.67	3.67	10	5.33	0	0	0.67	1.67	Bacteria	Chloroflexi	Gitt-GS-136	NA	NA	NA
ASV_973	2.67	1.33	1	1.33	0.33	1.33	1	1.33	0	2	0.33	0.67	Bacteria	Actinobacter	Rubrobacteri	Rubrobactera	Rubrobacteri	Rubrobacter
ASV_974	0	8	1	0	0.67	0	0.5	4	2	0	2.33	1	Bacteria	Actinobacter	Actinobacter	Euzebyales	Euzebyaceae	NA
ASV_975	3	1.67	0.33	3.67	0	0.67	0	1.67	0	0	0.67	1.33	Bacteria	Chloroflexi	Gitt-GS-136	NA	NA	NA
ASV_976	0.33	0	0.33	0	0.33	0	3	0	0	0	42.33	0	Bacteria	Proteobacter	Gammaprote	Enterobacter	Enterobacter	NA
ASV_977	0	0.67	0	0	0	0	0	21.33	0	0	0.67	0	Bacteria	Actinobacter	Thermoleopł	Gaiellales	NA	NA
ASV_978	2.67	0.67	1.33	0	1	2.67	11	0	5	0	1.33	0	Bacteria	Firmicutes	Bacilli	Staphylococc	Staphylococc	Jeotgalicoccus
ASV_979	3.33	9.67	1.33	0	0	0	0	0	0	0	1.33	0.67	Bacteria	Firmicutes	Bacilli	Staphylococc	Staphylococc	Staphylococcus
ASV_980	1.33	0	2.33	1	0.33	1.67	7.5	1.33	0	0	0.33	2.33	Bacteria	Chloroflexi	KD4-96	NA	NA	NA
ASV_981	0	0	0	3.33	0.67	0.33	0	0	0	0	0	0	Bacteria	Actinobacter	Acidimicrobii	IMCC26256	NA	NA
ASV_982	0	0	0	0	0.33	0	0	0	0	0	0.33	0	Bacteria	Actinobacter	Acidimicrobii	IMCC26256	NA	NA
ASV_983	0	9.67	1.67	0	0	0	0	4	1	0	0	2	Bacteria	Acidobacteri	Blastocatellia	Pyrinomonad	Pyrinomonad	RB41
ASV_984	0.33	0	1.33	0	1.33	0	0	0	0	0	9.33	0.33	Bacteria	Firmicutes	Bacilli	Staphylococc	Staphylococc	Staphylococcus
ASV_985	0	0.33	3.67	0	17.67	3.33	2	1	0	0	0.67	0.33	Bacteria	Actinobacter	Actinobacter	Corynebacter	NA	NA
ASV_986	0	0	0	0	0	0	0	0	0	0	0	0	Bacteria	Chloroflexi	Chloroflexia	Kallotenuales	AKIW781	NA
ASV_987	0	0	0	0	0	0	0	38.67	0	0	0	0	Bacteria	Proteobacter	Gammaprote	Pseudomona	Pseudomona	Pseudomonas
ASV_988	2.67	2	0	1.33	1	0.33	0	1.33	0	0	0	1.67	Bacteria	Acidobacteri	Blastocatellia	Blastocatellal	Blastocatellac	Stenotrophobacter
ASV_989	0	0	0	0	0	0	0	0	0	0	30	0.33	Bacteria	Actinobacter	Actinobacter	Euzebyales	Euzebyaceae	NA
ASV_990	0	0.67	0	0.67	1.33	1.67	5	1.33	2	0	1.67	1.33	Bacteria	Actinobacter	Thermoleopł	Solirubrobact	Solirubrobact	Solirubrobacter
ASV_991	0.33	3	1	0.67	0.33	0	0	2.33	0	0	0	1.33	Bacteria	Armatimonac	Chthonomon	Chthonomon	NA	NA
ASV_992	14	0	0	0	0	0	0	0	0	0	0	0	Bacteria	Actinobacter	Actinobacter	Corynebacter	Corynebacter	Corynebacterium
ASV_993	0	0	0	0	0	0	0	0.67	0	0	23.67	0.33	Bacteria	Proteobacter	Gammaprote	Enterobacter	Enterobacter	NA
ASV_994	0	0	0	0	0	0	0	0	0	0	27	0	Bacteria	NA	NA	NA	NA	NA
ASV_995	3.33	0	2.67	0	0	0	0	0	2	0	4	0	Bacteria	Actinobacter	Actinobacter	Micrococcale	Dermacoccac	Dermacoccus
ASV_996	1.33	0	1	1	0.67	4.67	5	3	1	0	0.33	0.33	Bacteria	Gemmatimor	Gemmatimor	Gemmatimor	Gemmatimor	NA
ASV_997	0.33	0	1.67	0	4	0.33	3	0	6	0	3.33	0	Bacteria	Actinobacter	Actinobacter	Pseudonocar	Pseudonocar	Actinomycetospora
ASV_998	1	0	0.33	2	0	1	5	3	2	0	1.33	0.33	Bacteria	Actinobacter	Acidimicrobii	NA	NA	NA
ASV_999	0	0	0	0	0	5.33	0	0	0	0	0	0	Bacteria	Proteobacter	Alphaproteol	Acetobactera	Acetobactera	Acidiphilium
ASV_1000	4.33	2.33	2.33	3	0.33	0.67	3	2	0	0	0	1.33	Bacteria	Actinobacter	Thermoleopł	Solirubrobact	67-14	NA
ASV_1001	1.33	0.67	2	1.33	1	1	3.5	1.67	0	5	1	0.67	Bacteria	Actinobacter	Rubrobacteri	Rubrobactera	Rubrobacteri	Rubrobacter
ASV_1002	0	0	0	0	0	0	0	0	0	0	27	0.67	Bacteria	Proteobacter	Alphaproteol	Caulobactera	Caulobactera	Phenylobacterium
ASV_1004	0	0	0	0	0	0	0	0	165	0	0.67	0	Bacteria	Actinobacter	Thermoleopł	Solirubrobact	Solirubrobact	Solirubrobacter
ASV_1005	0.33	0	0	0	1.67	3	0.5	0	0	0	0	0	Bacteria	Acidobacteri	Acidobacteria	Acidobacteria	Acidobacteria	NA
ASV_1006	0	0	0	0.33	0	0	0	0	0	0	0	0	Bacteria	Firmicutes	Sulfobacillia	Sulfobacillale	Sulfobacillace	NA
ASV_1007	0	0	0	0	0	9.67	0.5	0	1	0	0	0	Bacteria	Proteobacter	Gammaprote	Acidithiobacil	Acidithiobacil	Acidithiobacillus
ASV_1008	0	17.67	0	0	0	0	0	0	0	0	0	0	Bacteria	Proteobacter	Alphaproteol	Rhizobiales	Rhizobiaceae	NA
ASV_1009	5.33	0.33	0.33	1	0.67	1.33	1.5	1	0	0	0	0.33	Bacteria	Patescibacter	Saccharimon	Saccharimon	NA	NA
ASV_1010	0.33	0	0	12.67	0	0	0	0	0	0	0	0	Bacteria	Firmicutes	Negativicutes	Veillonellales	Veillonellacea	Veillonella
ASV_1011	3.33	1.67	1.33	1.67	0	0.33	6	2	0	0	0.67	1.67	Bacteria	Proteobacter	Gammaprote	Burkholderia	TRA3-20	NA
ASV_1012	1	0.67	1.67	0.67	1	0.67	3.5	3.33	0	0	0	2.33	Bacteria	Myxococcota	Polyangia	Haliangiales	Haliangiaceae	Haliangium
ASV_1013	0.33	0.67	0	1.33	0.67	3	4.5	2.33	2	0	0.33	2	Bacteria	Actinobacter	Thermoleopł	Solirubrobact	Solirubrobact	NA
ASV_1014	0	0	19	0	0	0	2	0	0	0	0	1	Bacteria	Campylobact	Campylobact	Campylobact	Sulfurospirilla	Sulfurospirillum
ASV_1015	0	0	0	0	0	0	0.5	0	0	0	0.33	29.33	Bacteria	Proteobacter	Gammaprote	Pseudomona	Moraxellacea	Enhydrobacter
ASV_1016	0.33	0.67	0	1.33	0.67	0.33	3.5	0	0	0	0.33	0.33	Bacteria	Actinobacter	Actinobacter	Propionibact	Nocardioidac	Nocardioides
ASV_1017	0	0	0	0	4.33	5	0	0	0	0	0	0	Bacteria	Firmicutes	Sulfobacillia	Sulfobacillale	Sulfobacillace	Sulfobacillus
ASV_1018	0	0	0	0	0	0	0	0	0	0	0	0	Bacteria	RCP2-54	NA	NA	NA	NA
ASV_1019	0	9	1.33	0	0.67	0	0	5.67	0	0	1	2.67	Bacteria	Actinobacter	Actinobacter	0319-7L14	NA	NA
ASV_1020	0	0	0	0	0.67	7.67	0	0	0	0	0	0	Bacteria	Firmicutes	Sulfobacillia	Sulfobacillale	Sulfobacillace	Sulfobacillus
ASV_1021	0	0	0	0	1	2	0	10	0	0	0	0	Archaea	Thermoplasn	Thermoplasn	Thermoplasn	Thermoplasn	A-plasma
ASV_1022	0	0	0	0	0	0	0	0.33	0	0	19.33	0	Bacteria	Actinobacter	Actinobacter	NA	NA	NA
ASV_1023	0	0	0	0	0	6	25	0	0	0	0	0.67	Bacteria	Actinobacter	Actinobacter	Micrococcale	Micrococcace	Kocuria
ASV_1024	0	8	0.33	0	0	0	0	4	1	0	1	2.67	Bacteria	Firmicutes	Bacilli	Staphylococc	Staphylococc	Staphylococcus
ASV_1025	3	0.67	0.33	2	0	0.67	1.5	0	0	0	0	0.33	Bacteria	Chloroflexi	TK10	NA	NA	NA
ASV_1026	0	9	0.67	0	1.33	0	0.5	3	1	0	0.33	2.33	Bacteria	Actinobacter	Actinobacter	Euzebyales	Euzebyaceae	Euzebya
ASV_1027	0.33	0.33	3.67	0	3	0	5.5	0.67	6	2	1.67	0	Bacteria	Actinobacter	Rubrobacteri	Rubrobactera	Rubrobacteri	Rubrobacter
ASV_1028	0	0.67	0	1.33	0	0	0	0	0	0	0	0.67	Bacteria	Actinobacter	Actinobacter	Micrococcale	Micrococcace	Nesterenkonia
ASV_1029	2	0.67	1	1	0	0.33	2	2	0	0	0	1.67	Bacteria	Armatimonac	NA	NA	NA	NA
ASV_1030	0	0	2.67	0	5	0	7.5	0	4	0	3.67	0	Bacteria	Proteobacter	Alphaproteol	Tistrellales	Geminicocca	Candidatus Alysiosphaera
ASV_1031	0	0	0	0	0	0	0	0	0	0	0	0	Bacteria	Firmicutes	Bacilli	Exiguobacter	Exiguobacter	Exiguobacterium
ASV_1032	13.33	0	0	0	0	0	0	0	0	0	0	0	Bacteria	Chloroflexi	Chloroflexia	Kallotenuales	AKIW781	NA
ASV_1033	1.33	1.67	1.67	0.33	0.67	0	1	0	0	0	0.33	1.33	Bacteria	Actinobacter	Thermoleopł	Gaiellales	Gaiellaceae	Gaiella
ASV_1034	0.67	0	12.67	0	0	0.33	0	0	0	0	0	0	Bacteria	Actinobacter	Actinobacter	Streptomyce	Streptomyce	Streptacidiphilus
ASV_1036	0	0	0	0	0	0.67	0	0	172	0	0	0	Bacteria	Gemmatimor	Longimicrobi	Longimicrobi	Longimicrobi	NA
ASV_1037	3.67	0.67	2.33	1	0	1.67	4.5	0.33	0	0	0.67	0.33	Bacteria	Nitrospirota	Nitrospiria	Nitrospirales	Nitrospiracea	Nitrospira
ASV_1038	1	0.67	1	0.67	1.67	1.67	3	3	0	0	0	0.33	Bacteria	Gemmatimor	Gemmatimor	Gemmatimor	Gemmatimor	NA
ASV_1039	3.33	0	3	1.33	0.67	1.33	3	0	0	0	0.33	0.67	Bacteria	Actinobacter	Thermoleopł	Solirubrobact	67-14	NA
ASV_1040	0.67	0.33	3.67	1.33	0	0	0.5	0.33	0	3	0	0	Bacteria	Verrucomicro	Verrucomicro	Chthoniobact	Chthoniobact	Chthoniobacter
ASV_1041	0	0	18	0	0	0	3.5	0	0	0	0	0.67	Bacteria	Proteobacter	Alphaproteol	Rhizobiales	Beijerinckiace	Methylobacterium-Methylorubrum
ASV_1042	0	0	2.33	0.33	3.67	0.33	6	0.67	4	0	2.33	1	Bacteria	Actinobacter	Thermoleopł	Solirubrobact	67-14	NA
ASV_1044	0	0	0	0	0	0	0	0	0	0	0	0	Bacteria	Chloroflexi	Anaerolineae	Ardenticaten	Ardenticaten	NA
ASV_1045	0	0	0	0	0	0	0	0	0	0	0	0	Bacteria	Chloroflexi	Ktedonobact	Ktedonobact	Ktedonobact	Thermogemmatispora
ASV_1046	1.33	0	2	0	1.33	0	5.5	0.67	3	0	4	0	Bacteria	Actinobacter	Actinobacter	Pseudonocar	Pseudonocar	Amycolatopsis
ASV_1047	0.33	0.67	0	0	0.33	4	18.5	0.33	0	0	0.67	0	Bacteria	Actinobacter	Actinobacter	Micromonos	Micromonos	NA
ASV_1048	15.33	0	0	0	0	0	0	0	0	0	0	0	Bacteria	Proteobacter	Gammaprote	Burkholderia	Comamonada	Acidovorax
ASV_1049	0	0	0	0	0	0	0	0	0	0	0	0	Bacteria	Chloroflexi	Dehalococco	S085	NA	NA
ASV_1050	2.67	1.33	0.67	4.67	3	0.33	2.5	0.67	0	0	0.67	0	Bacteria	Abditibacteri	Abditibacteri	Abditibacteri	Abditibacteri	Abditibacterium
ASV_1051	0	0	0	0	0.33	18.67	0	0	0	0	0	0	Bacteria	Actinobacter	Acidimicrobii	Acidimicrobia	Acidimicrobia	Ferrimicrobium
ASV_1052	1.33	0.33	8	0	0	0	2.5	0.67	0	0	0	0.33	Bacteria	Patescibacter	Saccharimon	Saccharimon	NA	NA
ASV_1053	2.33	0	4.33	0	0	0.33	0.5	0	0	0	0	0.33	Bacteria	Actinobacter	MB-A2-108	NA	NA	NA
ASV_1054	1	0.33	0	0	0	4.67	20.5	0	0	0	0	0	Bacteria	Bacteroidota	Bacteroidia	Bacteroidales	Prevotellacea	Prevotella_7
ASV_1055	0	5.33	1	0	0.33	0	0	7	0	0	0.33	2	Bacteria	Actinobacter	Thermoleopł	Solirubrobact	67-14	NA
ASV_1057	0	0	0	0	0	0	0	18	0	0	0	0.33	Archaea	Thermoplasn	Thermoplasn	Thermoplasn	Thermoplasn	A-plasma
ASV_1058	0.67	0	10.33	0	0	0	2	0	0	0	0.67	2.33	Bacteria	Proteobacter	Gammaprote	Burkholderia	Comamonada	NA
ASV_1059	0	0	0	0	0	0	0	0	0	0	20.67	0.33	Bacteria	Actinobacter	Actinobacter	0319-7L14	NA	NA
ASV_1060	0.33	0	0	0.33	0	0	0.5	1.67	0	0	0	0	Bacteria	Proteobacter	Alphaproteol	Sphingomona	Sphingomona	Sphingomonas
ASV_1061	0	0	0	0	0	0	0	0	0	0	0	0.33	Bacteria	Actinobacter	Actinobacter	0319-7L14	NA	NA
ASV_1062	2.33	0.33	1	0.33	1	2.33	3	1.33	0	0	0	1.33	Bacteria	Actinobacter	Thermoleopł	Solirubrobact	Solirubrobact	NA
ASV_1063	2.67	2	0	2	0	0.33	1	0	0	0	0	2	Bacteria	Chloroflexi	TK10	NA	NA	NA
ASV_1064	0	0	0	0	0	0	0	0	145	0	0.33	0	Bacteria	Actinobacter	Rubrobacteri	Rubrobactera	Rubrobacteri	Rubrobacter
ASV_1065	10.33	0	0	0	0	0	0	0	0	0	0	0	Bacteria	Proteobacter	Gammaprote	Burkholderia	Comamonada	Limnohabitans
ASV_1066	0	0.67	2.67	0	2.67	0	8	0.33	2	0	2	0	Bacteria	Firmicutes	Bacilli	Bacillales	NA	NA
ASV_1067	0	0	0	0	0	0	0	0	0	0	0	17.67	Bacteria	Proteobacter	Gammaprote	Pseudomona	Moraxellacea	Acinetobacter
ASV_1068	0.33	0	18.67	0	0	0	2.5	0	0	0	0.33	0.33	Bacteria	Proteobacter	Gammaprote	Gammaprote	Unknown Fai	Candidatus Berkiella
ASV_1069	0	0	0	0	5.67	12	1	0	0	0	0	0	Bacteria	Chloroflexi	AD3	NA	NA	NA
ASV_1070	0	0	2	0	1.33	0.33	4	0	0	0	1.33	0	Bacteria	Proteobacter	Gammaprote	Burkholderia	NA	NA
ASV_1071	0	0	0	0	0	0	0	0	0	0	29.67	0.33	Bacteria	Proteobacter	Gammaprote	Enterobacter	Enterobacter	NA
ASV_1072	0	0	0	0	0	0	0	0	0	0	0	0.33	Bacteria	Actinobacter	Thermoleopł	Gaiellales	NA	NA
ASV_1073	7.67	0	2.33	0	0.33	0	1.5	0	0	0	1	0	Bacteria	Actinobacter	Actinobacter	Pseudonocar	Pseudonocar	Amycolatopsis
ASV_1074	1	0.33	0	1.33	0.67	1.33	5.5	1.33	0	0	0.33	1	Bacteria	Actinobacter	Actinobacter	Frankiales	NA	NA
ASV_1075	1	0	0	1.33	1	5.67	18	0	0	0	0	0	Bacteria	Bacteroidota	Bacteroidia	Bacteroidales	Prevotellacea	Prevotella_7
ASV_1076	0	0.33	0	0	2.33	1	3	1.67	0	0	1	1.33	Bacteria	Proteobacter	Alphaproteol	Sphingomona	Sphingomona	Sphingomonas
ASV_1077	0.33	0	0	0	0.33	0	1.5	0	0	0	33.67	0	Bacteria	Proteobacter	Gammaprote	Enterobacter	Enterobacter	NA
ASV_1078	1	0	1	0.67	0.67	0	2.5	3.33	0	0	0.33	0.33	Bacteria	Chloroflexi	TK10	NA	NA	NA
ASV_1080	0	6.33	2	0	1	0	0	3.33	0	0	0.33	1.33	Bacteria	Proteobacter	Alphaproteol	Sphingomona	Sphingomona	Sphingomonas
ASV_1081	2	0	1	0.67	0	0.33	2.5	1.33	0	0	1.33	1.33	Bacteria	Actinobacter	Thermoleopł	Solirubrobact	67-14	NA
ASV_1082	0.33	0	0	0	0	3.67	22	0	0	0	0	0	Bacteria	Firmicutes	Bacilli	Lactobacillale	Streptococca	Streptococcus
ASV_1083	4.33	0.33	4	0	0.33	0	0	0	3	0	0.67	0	Bacteria	Actinobacter	Thermoleopł	Solirubrobact	Solirubrobact	Solirubrobacter
ASV_1084	0	0	0	0	0	0	0	0	127	0	0	0	Bacteria	Actinobacter	Thermoleopł	Gaiellales	NA	NA

ASV_1085	0	0	0	0.33	0	0	0	0	0	0	0	0	Bacteria	Firmicutes	Sulfobacillia	Sulfobacillale	Sulfobacillace	Sulfobacillus
ASV_1086	0	0	0	0	0	0	0	0	0	0	0.33	0	Bacteria	Acidobacteri	Holophagae	Subgroup 7	NA	NA
ASV_1087	0	0	2.33	0	3.33	0	7.5	0	4	0	1.67	0	Bacteria	Actinobacter	Acidimicrobii	NA	NA	NA
ASV_1088	12.67	0.33	0.33	0	0	0	1	0	0	0	0	0.67	Bacteria	Proteobacter	Gammaprote	Burkholderia	Burkholderia	Ralstonia
ASV_1089	1	0.33	0	0	0	0	0	0	0	0	0	1	Bacteria	Proteobacter	Alphaproteol	Rhodobacter	Rhodobacter	Paracoccus
ASV_1090	0.33	1	0	0.33	0	0	0	6	0	0	2	6.67	Bacteria	Proteobacter	Gammaprote	Pseudomona	Halomonada	Halomonas
ASV_1091	11	0.33	0	0	0	0	0	0	0	0	0	0	Bacteria	Chloroflexi	Chloroflexia	Thermomicro	JG30-KF-CM4	NA
ASV_1092	0	2	1.67	0.67	0	0.67	1	2.33	0	0	0	1	Bacteria	Actinobacter	Thermoleoph	Gaiellales	Gaiellaceae	Gaiella
ASV_1093	2	1.67	0	2.67	1.33	0	1.5	0	0	0	0.67	0	Bacteria	Actinobacter	Rubrobacteri	Rubrobactera	Rubrobacteri	Rubrobacter
ASV_1094	0.33	0.33	0	0	0.67	0.67	1.5	0	0	0	0	1	Bacteria	Actinobacter	Actinobacter	Propionibact	Nocardioidac	Nocardioides
ASV_1095	0.33	0	0	0.33	3.33	1.33	8	0.67	0	0	1.33	1.33	Bacteria	Actinobacter	Actinobacter	Propionibact	Nocardioidac	Aeromicrobium
ASV_1096	4.33	0.33	1	1	0	1	0	1.33	0	0	0.33	0	Bacteria	Proteobacter	Gammaprote	Nitrosococca	Nitrosococca	SZB85
ASV_1097	1.67	0	2	0.67	0	2.33	2	1.33	0	0	0	0.67	Bacteria	Actinobacter	Thermoleoph	Solirubrobact	67-14	NA
ASV_1098	8.33	0	0	0	0	0	0	0	0	0	0	0	Bacteria	Proteobacter	Gammaprote	Enterobacter	Pasteurellace	Haemophilus
ASV_1099	2.33	1.33	0.67	1.67	0.33	1	1.5	0.33	0	0	0	1	Bacteria	Actinobacter	Actinobacter	Propionibact	Nocardioidac	NA
ASV_1100	0	1	0.67	0	0	0	0	7	6	0	2	5.33	Bacteria	Actinobacter	Actinobacter	Corynebacte	Nocardiaceae	Nocardia
ASV_1101	0	0	0	0	0	0	0	1.33	0	0	17.33	0.67	Bacteria	Proteobacter	Gammaprote	Burkholderia	Comamonada	Hydrogenophaga
ASV_1102	0	0	0.67	0	0	0	0	0	0	0	0	0	Bacteria	Firmicutes	Bacilli	Alicyclobacilla	Alicyclobacilla	Alicyclobacillus
ASV_1103	0	0	0	0	0	0	0	0	0	2	19	0	Bacteria	Actinobacter	Actinobacter	Nitriliruptora	Nitriliruptora	NA
ASV_1104	0	0	0	0	0.33	0	0	1	0	0	17.67	0.33	Bacteria	Actinobacter	Actinobacter	Corynebacte	Dietziaceae	Dietzia
ASV_1105	0	0	0.67	0	0	0	1	0	0	0	0	0	Bacteria	Proteobacter	Alphaproteol	Rhodobacter	Rhodobacter	Rhodobacter
ASV_1106	0	0	0	0	0	0	0	0	0	0	0	0	Bacteria	Firmicutes	Sulfobacillia	Sulfobacillale	Sulfobacillace	Sulfobacillus
ASV_1107	0	0.33	0	0	0.33	3.33	0	0	0	0	0	0	Bacteria	Acidobacteri	Acidobacteria	Acidobacteria	Acidobacteria	NA
ASV_1108	0	0	0	0	0	0	0.5	0	0	0	0.67	21	Bacteria	Firmicutes	Bacilli	Lactobacillale	Aerococcacea	Aerococcus
ASV_1110	0	0	0	0	0	0	0	0	0	0	0	19	Bacteria	Firmicutes	Bacilli	Lactobacillale	Streptococca	Streptococcus
ASV_1111	2	0.33	0	0.67	0.33	1.33	5	1.67	0	0	1.33	1.33	Bacteria	Actinobacter	Thermoleoph	Solirubrobact	67-14	NA
ASV_1112	0	0	0.33	0	0	0	0	0	0	3	0	0	Bacteria	Firmicutes	Bacilli	Paenibacillale	Paenibacillac	Paenibacillus
ASV_1113	4.33	0	1	0.33	0	0	1.5	2	0	0	0.33	3	Bacteria	Proteobacter	Gammaprote	Legionellales	Legionellacea	Legionella
ASV_1114	0.33	0.67	1	0.33	0.67	1.33	3	0.67	0	0	1	0.67	Bacteria	Chloroflexi	Chloroflexia	Thermomicro	JG30-KF-CM4	NA
ASV_1115	0.33	0	0	0	0.33	5.33	0	11.33	0	0	0	0	Bacteria	Firmicutes	Bacilli	Alicyclobacilla	Alicyclobacilla	Acidibacillus
ASV_1116	0	0	0	0	0	0	0	0	0	0	0	0	Bacteria	Proteobacter	Gammaprote	Pseudomona	Moraxellacea	Enhydrobacter
ASV_1118	0	0	0	0	0	0	0	1.33	0	0	17	0	Bacteria	Gemmatimor	Longimicrobi	Longimicrobi	Longimicrobi	NA
ASV_1119	0.67	0	0.33	2	0.67	1.33	5	2.33	1	0	1.67	1.33	Bacteria	Actinobacter	Actinobacter	Frankiales	NA	NA
ASV_1120	0	0	0	0	0	1.67	0	0	0	0	0	0	Bacteria	Actinobacter	Acidimicrobii	NA	NA	NA
ASV_1121	0	0	0	0	0	0	0	16	0	0	0	0.33	Bacteria	Firmicutes	Sulfobacillia	Sulfobacillale	Sulfobacillace	Sulfobacillus
ASV_1122	1	0.67	4.33	0.67	0	1	2.5	0.67	0	0	0	0.67	Bacteria	Patescibacter	Saccharimona	Saccharimona	LWQ8	NA
ASV_1123	3.67	0.33	0.33	0	0	0.33	17.5	0	0	0	1	2.33	Bacteria	Bacteroidota	Bacteroidia	Flavobacteria	Flavobacteria	Flavobacterium
ASV_1124	0	0	16.67	0	0	0	3	0	0	0	0	0.33	Bacteria	Proteobacter	Gammaprote	Xanthomona	Xanthomona	Thermomonas
ASV_1125	0	0	0	0	0	0	0	23.33	0	0	0	0	Bacteria	Firmicutes	Bacilli	Alicyclobacilla	Alicyclobacilla	Acidibacillus
ASV_1126	0	0	12	0	0	0	0	0	0	0	0	0	Bacteria	Chloroflexi	NA	NA	NA	NA
ASV_1127	0	0	0	0	0	0	0	0.67	0	0	0.33	0	Bacteria	Gemmatimor	Gemmatimor	Gemmatimor	Gemmatimor	NA
ASV_1128	3.33	0	3.33	0	0.67	0	0	0	3	0	1.67	0	Bacteria	Firmicutes	Bacilli	Staphylococc	Staphylococc	Jeotgalicoccus
ASV_1129	0.67	0.33	2	0.33	1	1.33	3.5	1	0	2	0.33	1.33	Bacteria	Chloroflexi	KD4-96	NA	NA	NA
ASV_1130	0.33	0	2	0	2.33	0	5	0	2	0	1.67	0	Bacteria	Firmicutes	Bacilli	Staphylococc	Staphylococc	NA
ASV_1131	0	0	13.67	0	0	0	2	0	0	0	1	0	Bacteria	Proteobacter	Gammaprote	Pseudomona	Pseudomona	Pseudomonas
ASV_1132	0	0	0	0	0	0	0	17.67	0	0	0	0.33	Bacteria	Actinobacter	Rubrobacteri	Rubrobactera	Rubrobacteri	Rubrobacter
ASV_1133	1.67	1.67	0	2	0.33	2	13	0	0	0	0	0	Bacteria	Proteobacter	Alphaproteol	Rhizobiales	Xanthobacte	Afipia
ASV_1134	0	0	0	0	0.33	11	0	0	0	0	0	0	Bacteria	Proteobacter	Alphaproteol	Acetobactera	Acetobactera	Acidiphilium
ASV_1135	4	0	1	0.67	0	0.67	0	1.33	0	0	1	2	Bacteria	Actinobacter	Acidimicrobii	NA	NA	NA
ASV_1136	2	0	1.67	0.67	0	1.33	0	1.33	0	0	0.33	1.33	Bacteria	Patescibacter	Saccharimona	Saccharimona	NA	NA
ASV_1137	0.33	0	1.33	0	3.67	0	4	0	2	0	2	0.33	Bacteria	Actinobacter	Thermoleoph	Solirubrobact	67-14	NA
ASV_1139	0	0	0	0	0	0	0	0	0	0	0	0.67	Bacteria	Actinobacter	Actinobacter	0319-7L14	NA	NA
ASV_1140	3.33	0	0.33	0	1	0.33	0	3.33	0	0	0	0.67	Bacteria	Chloroflexi	TK10	NA	NA	NA
ASV_1141	0	0	12.67	0	0	0	1.5	0	0	0	0.33	1	Bacteria	Proteobacter	Gammaprote	Pseudomona	Pseudomona	Pseudomonas
ASV_1142	11	0	0	0.67	0.33	0	0	0	0	0	0	0	Bacteria	Proteobacter	Alphaproteol	Rhizobiales	Beijerinckiace	Methylobacterium-Methylorubrum
ASV_1143	0	0	0	0	0	0	0	0	5	0	0	12.33	Bacteria	Proteobacter	Gammaprote	Pseudomona	Halomonada	Halomonas
ASV_1144	2.33	2	2	2	1.33	0.33	2.5	0.33	0	0	0	1	Bacteria	Gemmatimor	Gemmatimor	Gemmatimor	Gemmatimor	NA
ASV_1145	2.33	0.67	0	0.67	0.67	3.33	7.5	0.67	0	0	0.67	0.33	Bacteria	Actinobacter	Actinobacter	Propionibact	Nocardioidac	Nocardioides
ASV_1146	7.67	1	0	0	0	0	0	0	0	0	0	0	Bacteria	Proteobacter	Gammaprote	Enterobacter	Idiomarinace	Idiomarina
ASV_1147	1.67	0.67	2.33	1	0	0.67	1.5	0	0	0	1.33	0.67	Bacteria	Actinobacter	Thermoleoph	Solirubrobact	Solirubrobact	Conexibacter
ASV_1148	0	0	0	0	0	0	0	23	0	0	1	0	Bacteria	Actinobacter	Actinobacter	Euzebyales	Euzebyaceae	NA
ASV_1149	0	0	0	0	0	0	0.5	0	0	0	0	12	Bacteria	Firmicutes	Bacilli	Bacillales	Bacillaceae	NA
ASV_1150	0	0	0	0	0	0	0	0	0	0	0	0	Bacteria	Actinobacter	Actinobacter	NA	NA	NA
ASV_1151	0	0	0	0	9.33	4.67	0	0	0	0	0	0	Bacteria	Firmicutes	Sulfobacillia	Sulfobacillale	Sulfobacillace	Sulfobacillus
ASV_1152	0	0	0	0	0	0	0	0	0	0	0	17.33	Bacteria	Firmicutes	Bacilli	Lactobacillale	Streptococca	Streptococcus
ASV_1153	2	0	3.67	0	1	0	0	0.33	0	0	1.33	0	Bacteria	Acidobacteri	Blastocatellia	Blastocatellal	Blastocatellac	Blastocatella
ASV_1154	0.33	0.33	0	0.67	2	1	6.5	1.33	2	0	1.67	0	Bacteria	Actinobacter	Thermoleoph	Solirubrobact	67-14	NA
ASV_1155	3	0	0	0	0	3.33	0.5	2	13	0	1	2.33	Bacteria	Proteobacter	Gammaprote	Burkholderia	Comamonada	NA
ASV_1156	9.67	0.67	0	0	0	0	0	0	0	0	0	0	Archaea	Crenarchaeo	Thermoprote	Sulfolobales	Sulfolobacea	Stygiolobus
ASV_1157	0	0	0	0	8	12.33	0	0	0	0	0.33	0	Bacteria	Firmicutes	Sulfobacillia	Sulfobacillale	Sulfobacillace	NA
ASV_1158	0	0	0	0	0	0	0	0	0	0	0	18	Bacteria	Firmicutes	Bacilli	Lactobacillale	Streptococca	Streptococcus
ASV_1159	2.33	2.33	1	0.67	1	1.67	2.5	0	0	0	0.67	0	Bacteria	Proteobacter	Alphaproteol	Sphingomona	Sphingomona	Sphingomonas
ASV_1160	1	0	0.33	1.67	1.33	2.67	8.5	1	0	0	0	0.67	Bacteria	Chloroflexi	KD4-96	NA	NA	NA
ASV_1161	8	0	0	0	0	0	0	0.33	0	0	0	0	Archaea	Crenarchaeo	Thermoprote	Sulfolobales	Sulfolobacea	Stygiolobus
ASV_1162	0	0	0	0	0	0	0	21.67	0	0	0	0	Bacteria	Actinobacter	Acidimicrobii	NA	NA	NA
ASV_1163	0	0	0	10.67	0	0	0	0	0	0	0	0	Bacteria	RCP2-54	NA	NA	NA	NA
ASV_1164	0.33	0	0	0	0	0	0.5	0	0	0	0	12.67	Bacteria	Firmicutes	Bacilli	Staphylococc	Staphylococc	Nosocomiicoccus
ASV_1165	0	16.33	0	0	0	0	0	0	0	0	0	0	Bacteria	Deinococcota	Deinococci	Thermales	Thermaceae	Thermus
ASV_1166	0	0	0.33	0	0	0	0.5	0	0	0	0	12.33	Bacteria	Proteobacter	Gammaprote	Pseudomona	Halomonada	Halomonas
ASV_1167	0	0	0	0	0	0	0	0	0	0	0	0	Bacteria	Firmicutes	Sulfobacillia	Sulfobacillale	Sulfobacillace	NA
ASV_1168	0	0	0	0	0	0	0	0	0	0	0	0	Bacteria	Proteobacter	Alphaproteol	Rhizobiales	Xanthobacte	NA
ASV_1169	1	0.67	1.67	1	0.33	1.33	3	0.33	0	4	0	0.67	Bacteria	Acidobacteri	Holophagae	Subgroup 7	NA	NA
ASV_1170	0	0	0	0	0	0	0	0	0	0	0	0	Bacteria	Actinobacter	Actinobacter	0319-7L14	NA	NA
ASV_1171	1.67	2.67	0.67	0.67	0.67	1.67	4.5	1.33	0	0	0.33	1.67	Bacteria	Actinobacter	Actinobacter	Micrococcale	Dermacoccac	Dermacoccus
ASV_1172	0	12	0	0	0	0.33	0	0	0	0	0	0	Bacteria	Firmicutes	Bacilli	Lactobacillale	Carnobacteri	Alloiococcus
ASV_1173	0	0	0	0	0	0	0	0	0	0	0	0	Bacteria	Firmicutes	Bacilli	Staphylococc	Staphylococc	Staphylococcus
ASV_1174	3	0.33	0.33	2.33	1.67	0.33	1	0	0	0	1	0.33	Bacteria	Proteobacter	Alphaproteol	Sphingomona	Sphingomona	Sphingomonas
ASV_1175	0	0	0	0	0	0	0	0	0	0	0	0	Bacteria	Acidobacteri	Acidobacteria	Subgroup 13	NA	NA
ASV_1176	0	0	0	0	0	0	0	0	0	0	0	0.33	Bacteria	Proteobacter	Gammaprote	Burkholderia	NA	NA
ASV_1177	1.67	3.33	0.33	1.33	0.33	0.33	2.5	0	0	0	0	1.33	Bacteria	Gemmatimor	Gemmatimor	Gemmatimor	Gemmatimor	NA
ASV_1178	0	0	0	0	0	0	0	0	0	0	0	0	Bacteria	RCP2-54	NA	NA	NA	NA
ASV_1179	0	0	13	0	0	0	1	0	0	0	0	0	Bacteria	Firmicutes	Bacilli	Lactobacillale	Enterococcac	Enterococcus
ASV_1180	0	0	0	0	0	0	0	0	0	4	0	21	Bacteria	NA	NA	NA	NA	NA
ASV_1181	0	0	0	0	0	0	0	1	0	0	14	0	Bacteria	Planctomyce	Phycisphaera	Tepidisphaer	WD2101 soil	NA
ASV_1182	0	0	0	0	0.33	0	0	0	0	0	17.33	0	Bacteria	Firmicutes	Bacilli	Lactobacillale	Lactobacillac	Leuconostoc
ASV_1183	0.33	0	1.67	0	2	1.67	7.5	0.33	0	0	1	0	Bacteria	Acidobacteri	Vicinamibact	Vicinamibact	NA	NA
ASV_1184	0.33	0	0	0	0	0	0	0	0	0	16	0	Bacteria	Proteobacter	Gammaprote	Burkholderia	Comamonada	Caenimonas
ASV_1185	0	5	1.33	0	0.67	0	7	2	2	0	0	1	Bacteria	Proteobacter	Alphaproteol	Caulobactera	Caulobactera	Brevundimonas
ASV_1186	0	13.33	0	0	0	0	0	0	0	0	0	0	Bacteria	Cyanobacteri	Sericytochro	NA	NA	NA
ASV_1187	0.67	0.33	0	1.67	0	0	1.5	0.67	0	0	0.33	0.33	Bacteria	Chloroflexi	Chloroflexia	Chloroflexale	Herpetosipho	Herpetosiphon
ASV_1188	1	0.33	0	0.67	0.67	2	8.5	1.33	2	0	0	0.67	Bacteria	Proteobacter	Alphaproteol	Sphingomona	Sphingomona	Sphingomonas
ASV_1189	0	0	0	0.67	0	0	0	0	0	0	0	0	Bacteria	Firmicutes	Sulfobacillia	Sulfobacillale	Sulfobacillace	Sulfobacillus
ASV_1190	1.67	0	2	0.33	0.33	0.67	0.5	1	0	0	0	2	Bacteria	Actinobacter	Acidimicrobii	Microtrichale	Iamiaceae	Iamia
ASV_1191	0	0	0	0	0.33	0	0	0	0	0	0	0	Bacteria	Proteobacter	Gammaprote	Gammaprote	Unknown Fai	Acidibacter
ASV_1192	9.67	0	0	0	0	0	0	0	0	0	0	0	Bacteria	Proteobacter	Gammaprote	Enterobacter	NA	NA
ASV_1193	0	0	0	0	3	4.33	0.5	0	0	0	0	0	Bacteria	Proteobacter	Gammaprote	Gammaprote	Unknown Fai	Acidibacter
ASV_1194	1.33	1.33	0	0.67	0.33	1.67	12	0	0	0	0	0	Bacteria	Firmicutes	Negativicutes	Veillonellales	Veillonellacea	Dialister
ASV_1195	0	6.67	0	0	0.33	0	0	5	1	0	1	2.67	Bacteria	Proteobacter	Alphaproteol	Rhizobiales	Pleomorphor	Pleomorphomonas
ASV_1196	0	0	0	0	0	0	0	0	0	0	16.67	0.33	Bacteria	Proteobacter	Gammaprote	Enterobacter	Enterobacter	Buttiauxella
ASV_1197	8	0	0	0	0	0	0.5	0	0	0	0	0	Bacteria	Proteobacter	Gammaprote	Pseudomona	Cellvibrionac	Cellvibrio
ASV_1198	1	0.67	1.33	1.33	0	1	2	1	0	0	1	1.33	Bacteria	Actinobacter	Thermoleoph	Solirubrobact	67-14	NA
ASV_1199	1.33	1.67	2.67	0.67	0	0.67	1	0.67	0	0	0	0.33	Bacteria	Patescibacter	Saccharimona	Saccharimona	NA	NA
ASV_1200	0.67	0	0	0	0	0	12	0	0	0	18	1	Bacteria	Actinobacter	Acidimicrobii	Microtrichale	Ilumatobacte	CL500-29 marine group
ASV_1201	0.67	0	0	0	0	0	2	0	0	0	26	0.67	Bacteria	Proteobacter	Gammaprote	Enterobacter	Enterobacter	NA
ASV_1202	0	0	0.33	0	0	0	0	0	0	0	0	0	Bacteria	Proteobacter	Gammaprote	Burkholderia	Nitrosomona	GOUTA6
ASV_1203	0	0.33	0	0	0.67	3.67	10.5	0	3	0	1.67	0	Bacteria	Firmicutes	Bacilli	Lactobacillale	Streptococca	Streptococcus
ASV_1204	2	0	0.33	0.67	2	0	3	1.33	0	0	2	0	Bacteria	Chloroflexi	JG30-KF-CM6	NA	NA	NA
ASV_1205	0.67	0.33	0.67	0.33	0.33	1.67	2	0.67	0	2	0	0.67	Bacteria	Actinobacter	Acidimicrobii	Microtrichale	Ilumatobacte	NA
ASV_1206	0.33	0	0	0	0	0	0	0	0	0	0	0	Bacteria	Firmicutes	NA	NA	NA	NA
ASV_1207	0	0	0	0	0	0	0	0	0	0	0	0.33	Bacteria	Proteobacter	Gammaprote	Burkholderia	Oxalobactera	Massilia
ASV_1208	0	0	0	0	0	0	0	0	0	0	0	0.67	Bacteria	Actinobacter	Actinobacter	Euzebyales	Euzebyaceae	NA
ASV_1209	2.33	0.33	1	1	0	1.33	0	0	1	0	0	1.67	Bacteria	Actinobacter	Acidimicrobii	NA	NA	NA
ASV_1210	0	0	1.67	0	2.67	0	1.5	0	2	0	2	0	Bacteria	Actinobacter	Acidimicrobii	IMCC26256	NA	NA
ASV_1211	0.33	0	0	0	0	0	0	0	0	0	0	0	Bacteria	Actinobacter	Thermoleoph	Solirubrobact	67-14	NA
ASV_1212	0	11.67	0	0	0	0	0	0	0	0	0	0	Bacteria	Bacteroidota	Bacteroidia	Flavobacteria	Weeksellacea	Moheibacter
ASV_1213	0	0	0	0	0	0	0	0.67	0	0	13.33	0.33	Bacteria	Actinobacter	Actinobacter	NA	NA	NA
ASV_1214	2.67	0	3.33	0	0.33	0	0	0	0	0	0.33	0	Bacteria	Gemmatimor	Longimicrobi	Longimicrobi	Longimicrobi	NA
ASV_1215	0	0	3	9.33	0	0	0	0	0	0	0	0	Archaea	Crenarchaeo	Thermoprote	Sulfolobales	Sulfolobacea	NA
ASV_1216	0.33	2.33	1	0.33	0	0	0.5	0.67	0	0	0	4	Bacteria	Actinobacter	Thermoleoph	Gaiellales	Gaiellaceae	Gaiella
ASV_1217	0	0	0	0	0	0	0	0	101	0	0	0	Bacteria	Chloroflexi	Chloroflexia	Kallotenuales	AKIW781	NA
ASV_1218	0	0	0	0	0	0	1	0	0	0	0	19.67	Bacteria	Actinobacter	MB-A2-108	NA	NA	NA
ASV_1219	0	0	0	0	0	0	0	0	0	0	0	14	Bacteria	Actinobacter	Actinobacter	Corynebacte	Corynebacte	Lawsonella
ASV_1220	0	0	0	0	0	0	0	1.33	0	0	13.67	0	Bacteria	Actinobacter	Rubrobacteri	Rubrobactera	Rubrobacteri	Rubrobacter
ASV_1221	1	1	1	1	0	0.33	0	0	0	0	0.33	1.33	Bacteria	Patescibacter	Saccharimona	Saccharimona	NA	NA
ASV_1222	1.67	0.67	0	1.33	0	0	0.5	0	0	0	0	0	Bacteria	Gemmatimor	Gemmatimor	Gemmatimor	Gemmatimor	NA
ASV_1223	1	0.33	0.67	0	0	1.33	2	1	0	3	1	0.33	Bacteria	Actinobacter	Actinobacter	Propionibact	Nocardioidac	Kribbella
ASV_1225	0.33	1.33	0	0	0	0	0	14	0	0	0	0	Bacteria	Firmicutes	Bacilli	Lactobacillale	Streptococca	Lactococcus
ASV_1226	6	0	0	0	0	0	0	0.33	0	0	0	0	Archaea	Crenarchaeo	Thermoprote	Sulfolobales	Sulfolobacea	Stygiolobus
ASV_1227	0.67	0	8.33	0	0	0	4.5	0	0	0	0	1.67	Bacteria	Planctomyce	Planctomyce	Pirellulales	Pirellulaceae	NA
ASV_1228	0	0	0	0	0	0	0.5	0	0	0	0	6.67	Bacteria	Actinobacter	MB-A2-108	NA	NA	NA
ASV_1229	0	0	0	0	0	0	0	0	0	0	0	0	Archaea	Crenarchaeo	Thermoprote	Sulfolobales	Sulfolobacea	NA
ASV_1230	0.33	0	0	0.67	0.33	0.67	3.5	0.33	0	0	0.67	0.33	Bacteria	Myxococcota	Polyangia	Haliangiales	Haliangiaceae	Haliangium
ASV_1231	0.33	0	0	5.67	0	0	0	0	0	0	0	0	Bacteria	Proteobacter	Gammaprote	Pseudomona	Moraxellacea	Acinetobacter
ASV_1232	0	0	0	0	0	0	0	0	0	0	0	0	Bacteria	RCP2-54	NA	NA	NA	NA
ASV_1233	0	10.33	0	0	0	0	0	0	0	0	0	0	Bacteria	Firmicutes	Bacilli	Bacillales	Bacillaceae	Geobacillus
ASV_1234	0.67	1	1	1	0	0.67	1	0.33	0	0	0	0.67	Bacteria	Actinobacter	Thermoleoph	Solirubrobact	67-14	NA
ASV_1235	0.33	0	0.33	0.33	0	0	0.5	0.67	0	0	0	0	Bacteria	Actinobacter	Thermoleoph	Gaiellales	Gaiellaceae	Gaiella

ASV_1236	0	0	0	0	0	0	0	0	0	0	0	0	Bacteria	Chloroflexi	Chloroflexia	Thermomicrc	JG30-KF-CM4	NA
ASV_1237	0	0	0	0	0	0	1	0	0	0	0	20	Bacteria	Actinobacter	Thermoleoph	Solirubrobact	67-14	NA
ASV_1239	0	6.67	1	1.33	0	0	0	0	0	0	0	0	Archaea	Crenarchaeo	Thermoprote	Sulfolobales	Sulfolobacea	Stygiolobus
ASV_1240	0	0	0	0	0	0	0.5	0	0	0	0	22	Bacteria	Actinobacter	Actinobacter	Corynebacter	Corynebacter	Corynebacterium
ASV_1241	0	0	0	0	0	0	0	0.67	0	0	14.33	0.33	Bacteria	Actinobacter	Thermoleoph	Solirubrobact	67-14	NA
ASV_1242	0	0	0	0	0	0	0	0	0	0	0	0	Bacteria	Actinobacter	Acidimicrobii	NA	NA	NA
ASV_1243	0	0.33	0	0.33	1.67	2	9	0	0	0	0	0.67	Bacteria	Actinobacter	Actinobacter	Micrococcale	Dermabacter	Brachybacterium
ASV_1244	0.67	0	0.67	0.33	1	2.33	2.5	1.33	0	0	1.33	0.67	Bacteria	Actinobacter	Thermoleoph	Solirubrobact	Solirubrobact	Solirubrobacter
ASV_1245	2	1.33	0	1.67	0	0.67	1	2.67	0	0	0.33	0.67	Bacteria	Verrucomicro	Verrucomicro	Verrucomicro	Verrucomicro	NA
ASV_1246	0	0	0	0	0	0	0	0	98	0	0.33	0	Bacteria	Proteobacter	Gammaprote	Burkholderia	Comamonada	Tepidimonas
ASV_1247	0	0	0	2	0	0	0	0	0	0	0	0	Bacteria	Firmicutes	Negativicutes	Veillonellales	Veillonellacea	Veillonella
ASV_1248	0.67	0	0	1	0.67	2.67	4	2	0	0	0	0.67	Bacteria	Verrucomicro	Verrucomicro	Chthoniobact	Chthoniobact	LD29
ASV_1250	2.33	0	4.33	0.33	0.33	0	0	0	2	0	0.67	0	Bacteria	Actinobacter	Actinobacter	0319-7L14	NA	NA
ASV_1251	1	0.67	1	1.33	1.33	0.33	0.5	1	0	0	1	0	Bacteria	Actinobacter	Thermoleoph	Solirubrobact	67-14	NA
ASV_1252	3	1.33	0	1	0	0.67	2	1	0	0	0	0.33	Bacteria	Firmicutes	Bacilli	Paenibacillale	Paenibacillac	NA
ASV_1253	0.67	0.33	1	1	0.33	0.33	2	0	0	2	0	0	Bacteria	Acidobacteri	Blastocatellia	Blastocatellal	Blastocatellac	NA
ASV_1254	1.33	2.33	1.67	2	0	0	0	0	0	0	0	0	Archaea	Crenarchaeo	Thermoprote	Sulfolobales	Sulfolobacea	Stygiolobus
ASV_1255	0	0	11.33	0	0	0	2	0	0	0	0.33	0.33	Bacteria	Actinobacter	Acidimicrobii	NA	NA	NA
ASV_1257	1.33	0	0.33	0.33	1.33	1.33	3	1	0	0	0	2	Bacteria	Patescibacter	Saccharimon	Saccharimon	NA	NA
ASV_1258	2.67	0.33	0	1.67	0	0	0.5	0	0	0	0	0	Bacteria	Actinobacter	Thermoleoph	Gaiellales	Gaiellaceae	Gaiella
ASV_1259	3.33	2.33	0	0.67	1	0	0	0	0	0	0	0	Bacteria	Abditibacteri	Abditibacteri	Abditibacteri	Abditibacteri	Abditibacterium
ASV_1260	0	0	0	0	0.33	0	0	0	96	0	0	0	Bacteria	Proteobacter	Gammaprote	Pseudomona	Pseudomona	Pseudomonas
ASV_1261	2	1	1.67	0	0	0.33	0.5	0.33	0	0	0	0.33	Bacteria	Verrucomicro	Verrucomicro	Chthoniobact	Chthoniobact	Chthoniobacter
ASV_1262	0	11.33	0	0	0	0	0	0	0	0	0	0	Bacteria	Proteobacter	Alphaproteol	Rhodobacter	Rhodobacter	Defluviimonas
ASV_1263	0	1.33	0	0	0	11.67	0	1.33	0	0	0	0.33	Bacteria	Actinobacter	Acidimicrobii	Acidimicrobia	Acidimicrobia	Ferrimicrobium
ASV_1264	1.33	0	2.67	0	0.33	0	0	0	0	2	3	0	Bacteria	Actinobacter	Thermoleoph	Solirubrobact	67-14	NA
ASV_1265	0.67	0.33	1.33	0.33	0.33	0	0	1.33	0	0	0	1.67	Bacteria	Chloroflexi	TK10	NA	NA	NA
ASV_1266	0	0	0	0	0	0.67	0	0	0	0	0	0	Bacteria	Firmicutes	Bacilli	Alicyclobacill	Alicyclobacill	Alicyclobacillus
ASV_1267	0	7.67	0.33	0	0	0	0	3	0	0	0.33	0.33	Bacteria	Bacteroidota	Bacteroidia	Cytophagales	Hymenobact	Hymenobacter
ASV_1268	0.33	0	0	0	0	0	0	0	0	0	13	0	Bacteria	Actinobacter	Actinobacter	Euzebyales	Euzebyaceae	NA
ASV_1270	0	4.33	0.67	0	0	0	0	2	0	0	0.33	0.67	Bacteria	Actinobacter	Thermoleoph	Solirubrobact	67-14	NA
ASV_1271	0	0	0	0	0	0	0	1	0	0	13	0	Bacteria	Actinobacter	Rubrobacteri	Rubrobacter	Rubrobacteri	Rubrobacter
ASV_1272	0	0.67	0.33	0.67	0.67	0.33	2.5	0	0	3	0	0	Bacteria	Actinobacter	Actinobacter	Propionibact	Nocardioidac	Nocardioides
ASV_1273	0	0	0.33	0.33	1	1	7	1.67	0	0	0.67	1.67	Bacteria	Patescibacter	NA	NA	NA	NA
ASV_1274	0.33	0	0	0	0.67	3.33	2	1.67	0	0	0	0	Bacteria	Actinobacter	Acidimicrobii	NA	NA	NA
ASV_1276	0	0	0	0	0	0	0	0	0	0	0.33	0	Bacteria	Firmicutes	Bacilli	Staphylococc	Staphylococc	Staphylococcus
ASV_1278	1.67	0.67	1.33	1.67	0	1	0.5	1	0	0	0	0	Bacteria	Gemmatimor	Gemmatimor	Gemmatimor	Gemmatimor	NA
ASV_1279	0	0	0	0	2.33	1	7	4	0	0	0.33	0	Bacteria	Actinobacter	Actinobacter	Corynebacter	Mycobacteria	Mycobacterium
ASV_1281	0	0	0	0	0	0	0	0	0	0	0	0	Bacteria	Proteobacter	Alphaproteol	Rhizobiales	Xanthobacter	NA
ASV_1282	0	0	0	0	0	0	0	0	0	0	0	0	Bacteria	RCP2-54	NA	NA	NA	NA
ASV_1283	0	0	0	0.33	0	0	0	0	0	0	0	0	Archaea	Crenarchaeo	Thermoprote	Sulfolobales	Sulfolobacea	NA
ASV_1284	0	0	0	0	0	0	0	1	0	2	7	0	Bacteria	Actinobacter	Thermoleoph	Gaiellales	NA	NA
ASV_1285	0	0	0	0	0	0	0	0	0	0	0	0	Bacteria	Bacteroidota	Bacteroidia	Bacteroidales	Dysgonomon	Dysgonomonas
ASV_1286	0	0	0	0	0	0	0	13.67	0	0	0	0	Bacteria	Actinobacter	Thermoleoph	Solirubrobact	Solirubrobact	NA
ASV_1287	1.67	0.33	1	0	0.67	1	2	0.67	0	0	0	1	Bacteria	Actinobacter	Thermoleoph	Gaiellales	Gaiellaceae	Gaiella
ASV_1288	2.67	1.33	0.67	2	0	1.33	3.5	0.33	0	0	0	0	Bacteria	Myxococcota	Polyangia	Haliangiales	Haliangiaceae	Haliangium
ASV_1289	0.33	0	0	0	0	0	0	0.33	0	0	15.33	0	Bacteria	Firmicutes	Bacilli	Bacillales	Planococcace	NA
ASV_1290	0	0	0	0	1.33	11.33	1.5	0.67	0	0	0	0	Archaea	Thermoplasn	Thermoplasn	Thermoplasn	Thermoplasn	A-plasma
ASV_1291	0	0	0.67	0	0.33	0	1	0.33	0	0	0.33	1.67	Bacteria	Actinobacter	Thermoleoph	Gaiellales	Gaiellaceae	Gaiella
ASV_1292	0	0	1.67	0	0.33	0	3.5	0	0	0	3	0	Bacteria	Actinobacter	Actinobacter	Euzebyales	Euzebyaceae	NA
ASV_1293	0	0	0	0	0	0	0	0	0	0	17.33	0.67	Bacteria	Actinobacter	Actinobacter	Corynebacter	Corynebacter	Turicella
ASV_1294	0	0	0	0	0	0	0	18.67	0	0	0	0	Bacteria	Dependentia	Babeliae	Babeliales	Babeliaceae	NA
ASV_1295	2	0.33	1.67	0.33	0.67	0	0.5	0.33	0	3	0	0	Bacteria	Gemmatimor	Gemmatimor	Gemmatimor	Gemmatimor	NA
ASV_1296	0	0	0	0	0.33	0	0	0	93	0	0	0	Bacteria	Firmicutes	Bacilli	Staphylococc	Staphylococc	Staphylococcus
ASV_1297	0	0	0	0	0	0	0	0	0	0	0	0	Bacteria	Chloroflexi	Ktedonobact	Ktedonobact	Ktedonobact	Thermogemmatispora
ASV_1298	2	6	0.33	0	0.33	0	0	0	0	0	0	1	Bacteria	Firmicutes	Bacilli	Staphylococc	Staphylococc	Staphylococcus
ASV_1299	0	0	0	0	0.67	2.67	11.5	0	4	0	1.33	0	Bacteria	Actinobacter	Actinobacter	NA	NA	NA
ASV_1300	0	0.33	10.33	0	0	0	2.5	0	0	0	0	0	Bacteria	Proteobacter	Alphaproteol	Rhizobiales	Beijerinckiace	Methylobacterium-Methylorubrum
ASV_1301	0	0	0	0	0	0	0	0	0	0	0	13.67	Bacteria	Firmicutes	Bacilli	Lactobacillale	Streptococca	Streptococcus
ASV_1302	8.33	0	0	0	0	0	0	0	0	0	0	0	Bacteria	Proteobacter	Gammaprote	Enterobacter	Pasteurellace	Haemophilus
ASV_1303	1.67	0	0.67	0	0	0	2	0	0	0	0	0	Bacteria	Patescibacter	Saccharimon	Saccharimon	Saccharimon	TM7a
ASV_1304	7.33	0	0.33	0	0	0	0	0	0	0	0	0	Archaea	Crenarchaeo	Thermoprote	Sulfolobales	Sulfolobacea	Stygiolobus
ASV_1305	0	0	10	0	0	0	2.5	0	0	0	1	0	Bacteria	Actinobacter	Actinobacter	Corynebacter	Corynebacter	Corynebacterium
ASV_1306	0	0	0	7.33	0	0	0	0	0	0	0	0	Bacteria	Actinobacter	Actinobacter	Micrococcale	Micrococcace	Nesterenkonia
ASV_1307	0	0	0	0	0	0.33	0	0	0	0	0	17.33	Bacteria	Proteobacter	Gammaprote	Pseudomona	Moraxellacea	Enhydrobacter
ASV_1308	0	0	0	0	0	0	0	0	0	0	0	0	Bacteria	Firmicutes	Sulfobacillia	Sulfobacillale	Sulfobacillace	Sulfobacillus
ASV_1309	1	0	0.33	0.33	1.33	1.33	1	2.33	0	3	0	0	Bacteria	Chloroflexi	Chloroflexia	Thermomicrc	JG30-KF-CM4	NA
ASV_1310	1	0.67	1.33	0.67	0.67	0.67	1.5	0.33	0	5	0.67	0.33	Bacteria	Actinobacter	Rubrobacteri	Rubrobacter	Rubrobacteri	Rubrobacter
ASV_1312	2	0	0.33	0	1.33	1.67	2	1.33	0	0	0.33	1	Bacteria	Acidobacteri	Blastocatellia	Pyrinomonac	Pyrinomonad	RB41
ASV_1313	0.33	0	0	0	0	0	1.5	0	0	0	0	0.67	Bacteria	Actinobacter	Actinobacter	Streptomyce	Streptomyce	Streptomyces
ASV_1314	1.67	0.33	1.67	0.67	0	0.67	2.5	0	0	0	0	0.33	Bacteria	Patescibacter	NA	NA	NA	NA
ASV_1315	0.33	0	1	0.67	0.67	0.33	6	0.67	0	0	0.67	0.33	Bacteria	Actinobacter	Actinobacter	Propionibact	Nocardioidac	Aeromicrobium
ASV_1316	0	0	0	0	0	0	0	0	0	109	0	0	Bacteria	Actinobacter	Thermoleoph	Solirubrobact	67-14	NA
ASV_1317	2.33	0	4	0	0.67	0	0	0.33	0	0	0.67	0	Bacteria	Gemmatimor	Gemmatimor	Gemmatimor	Gemmatimor	NA
ASV_1318	0	1	0.33	0	0	1.33	3	1	0	0	0	1	Bacteria	Actinobacter	Thermoleoph	Solirubrobact	Solirubrobact	Solirubrobacter
ASV_1320	0	0	0	0	0	0	0	0	0	0	0	7.67	Bacteria	RCP2-54	NA	NA	NA	NA
ASV_1321	0	7.67	0	0	0	0.33	0	0	0	0	0	0	Bacteria	Actinobacter	Actinobacter	Micrococcale	Micrococcace	Kocuria
ASV_1322	0	9	0	0	0	0	0	0	0	0	0	0	Bacteria	Firmicutes	Bacilli	Bacillales	Bacillaceae	Geobacillus
ASV_1323	0	0	0	0	1.67	1	0.5	3.33	0	0	0	0	Bacteria	Actinobacter	Acidimicrobii	NA	NA	NA
ASV_1324	0	0	0	0	0	0	0	0	0	0	0	0.33	Bacteria	Actinobacter	Actinobacter	0319-7L14	NA	NA
ASV_1325	2	0	1.67	0	0.67	0	0	0	0	0	1	0	Bacteria	Actinobacter	Thermoleoph	Solirubrobact	Solirubrobact	Solirubrobacter
ASV_1326	1.33	0	0.33	0	0	0	2	1.33	0	0	0.67	2.67	Bacteria	Actinobacter	Actinobacter	Frankiales	Sporichthyac	NA
ASV_1327	2.33	0.33	1	1.33	0	0	0.5	2	0	2	0	0.67	Bacteria	Actinobacter	Acidimicrobii	Microtrichale	Ilumatobacte	NA
ASV_1328	0	0	0.33	0	0	0	0.5	0	0	0	0	4.33	Bacteria	Actinobacter	Actinobacter	Propionibact	Propionibact	Cutibacterium
ASV_1329	0	0	0	0	0	0	0	15.67	0	0	0	0	Bacteria	NA	NA	NA	NA	NA
ASV_1330	0	0	0	0	0	0	0	0	0	0	0	0	Bacteria	Actinobacter	Acidimicrobii	IMCC26256	NA	NA
ASV_1331	0	0	0	0	0	0	0	0.67	0	0	0	0	Bacteria	Proteobacter	Gammaprote	Burkholderia	Comamonada	Paucibacter
ASV_1332	0	0	0	0	0	0	0	0	0	0	0	0	Bacteria	Firmicutes	Sulfobacillia	Sulfobacillale	Sulfobacillace	Sulfobacillus
ASV_1333	1.67	0.33	0.67	0	0.33	0.67	0.5	0.33	0	0	0	0.33	Bacteria	Actinobacter	Thermoleoph	Solirubrobact	67-14	NA
ASV_1334	0.67	3	0.67	2.33	0	0	0	0	0	0	0	0	Archaea	Crenarchaeo	Thermoprote	Sulfolobales	Sulfolobacea	Stygiolobus
ASV_1335	1.33	0	0.33	0	0.67	0.33	2	0.33	0	0	1	0.33	Bacteria	Actinobacter	Actinobacter	Propionibact	Nocardioidac	Nocardioides
ASV_1336	0.33	0.33	0.67	1.33	0	1.33	3	1	0	0	0.67	0	Bacteria	Actinobacter	Thermoleoph	Solirubrobact	Solirubrobact	Solirubrobacter
ASV_1337	0.33	0	0	1	0.33	3.67	2	2	0	0	1	0.33	Bacteria	Actinobacter	Acidimicrobii	NA	NA	NA
ASV_1338	0	0	1.33	0	0	0	2.5	0	3	0	0.33	0.33	Bacteria	Actinobacter	Actinobacter	Euzebyales	Euzebyaceae	NA
ASV_1339	1.67	0	1	0.67	0	0.67	0	2	0	0	0.33	1.33	Bacteria	Actinobacter	Acidimicrobii	NA	NA	NA
ASV_1340	1	0	0	0.33	0	0.67	3	1.67	0	3	1.67	1.33	Bacteria	Actinobacter	Actinobacter	Frankiales	NA	NA
ASV_1341	0	0	0	0	0	0	0	0	0	0	12.67	1.67	Bacteria	Proteobacter	Gammaprote	Pseudomona	Moraxellacea	Enhydrobacter
ASV_1342	0	0	0	0	2	1	1.5	4.33	0	0	0	0	Archaea	Thermoplasn	Thermoplasn	Thermoplasn	Thermoplasn	A-plasma
ASV_1343	7.33	0	0	0.67	0	0	0	0	0	0	0	0	Bacteria	Proteobacter	Alphaproteol	Rhizobiales	Beijerinckiace	Methylobacterium-Methylorubrum
ASV_1344	0	0	0	0	0	0	0	0.67	0	0	0	0	Bacteria	Firmicutes	Bacilli	Bacillales	Bacillaceae	NA
ASV_1345	0	0	0	0	3	1.67	0	0	0	0	0	0	Bacteria	Firmicutes	Sulfobacillia	Sulfobacillale	Sulfobacillace	Sulfobacillus
ASV_1346	0	0	0	0	0	0	0	15	0	0	0	0	Bacteria	Actinobacter	Rubrobacteri	Rubrobacter	Rubrobacteri	Rubrobacter
ASV_1347	1.67	0.33	1.67	0	0.33	0	0	0	2	0	0.33	0	Bacteria	Actinobacter	Actinobacter	Micrococcale	Micrococcace	Rothia
ASV_1348	0	0	0	0	0	0	0	12	0	0	1.33	0	Bacteria	Actinobacter	Thermoleoph	Solirubrobact	67-14	NA
ASV_1350	0	0.33	0	0.33	0	2	9	0	0	0	1	0	Bacteria	Actinobacter	Actinobacter	Nitriliruptora	Nitriliruptora	NA
ASV_1351	0	0	0	0	0	0	0	0	0	0	0	16.67	Bacteria	Actinobacter	Actinobacter	Corynebacter	Corynebacter	Corynebacterium
ASV_1352	0	10.33	0	0	0	0	0	0	0	0	0	0	Archaea	Crenarchaeo	Thermoprote	Sulfolobales	Sulfolobacea	Stygiolobus
ASV_1353	0	0.67	2.67	0	0	0	0	0	0	0	0	0	Archaea	Crenarchaeo	Thermoprote	Sulfolobales	Sulfolobacea	Stygiolobus
ASV_1354	0	0	0.33	0	0	0	0	0	0	105	0	0	Bacteria	Proteobacter	Gammaprote	Pseudomona	Halomonada	Halomonas
ASV_1355	0	0	0	0	0	0	0	1.33	0	0	12	0	Bacteria	Actinobacter	Actinobacter	Pseudonocar	Pseudonocar	NA
ASV_1356	1.33	0	0	1	0.67	1.33	7	0	0	0	0	0	Bacteria	Proteobacter	Gammaprote	Burkholderia	Comamonada	Tepidimonas
ASV_1357	0	0	0	0	0	0	0	0	0	0	0	0	Bacteria	Proteobacter	Gammaprote	Burkholderia	Comamonada	Paucibacter
ASV_1358	0	0	10.67	0	0	0	3	0	0	0	0	0.33	Bacteria	Bacteroidota	Bacteroidia	Flavobacteria	Weeksellacea	NA
ASV_1359	0	0	0	0	0	0	1	0	0	0	0	16.33	Bacteria	Actinobacter	Actinobacter	Micrococcale	Microbacteri	Microbacterium
ASV_1361	1.67	1	0	0.67	0.67	1	0.5	0	0	0	0	1.67	Bacteria	Actinobacter	Thermoleoph	Solirubrobact	67-14	NA
ASV_1362	0.33	1	0.67	0.33	0.67	1.33	2	1.33	0	0	0.67	0.33	Bacteria	Actinobacter	Thermoleoph	Gaiellales	NA	NA
ASV_1363	0.67	0.67	1	1	2	0.67	0	0	0	0	1.33	0	Bacteria	Actinobacter	Actinobacter	Euzebyales	Euzebyaceae	NA
ASV_1364	0	0	0	0	3	1.33	3	0.33	0	0	2	0.33	Bacteria	Bacteroidota	Bacteroidia	Chitinophaga	Chitinophaga	NA
ASV_1365	0	0	0	0	0	0	0	0	0	0	0	15.67	Bacteria	Proteobacter	Gammaprote	Pseudomona	Moraxellacea	Acinetobacter
ASV_1367	0	0	0	0	0	0	0	0	0	0	14.67	0.33	Bacteria	Armatimonac	NA	NA	NA	NA
ASV_1368	0	0	0	0	0	0	0.5	0	0	0	0	8	Bacteria	Acidobacteri	Blastocatellia	Pyrinomonac	Pyrinomonad	RB41
ASV_1369	5.67	0	0	0	0	0	0	0	0	0	0	0	Bacteria	Proteobacter	Alphaproteol	Rhizobiales	Beijerinckiace	Methylobacterium-Methylorubrum
ASV_1370	0	0	0	3.33	0	0.33	2.5	0.33	0	0	0	0	Bacteria	Planctomyce	Planctomyce	Planctomyce	NA	NA
ASV_1371	0	5.33	0.33	0	0	0	0	2.33	0	0	0.67	0.67	Bacteria	Actinobacter	Thermoleoph	Solirubrobact	67-14	NA
ASV_1372	0	0	0	0	0	0	0	0	0	0	0	11	Bacteria	Firmicutes	Bacilli	Lactobacillale	Streptococca	Streptococcus
ASV_1373	0	0	0	0	0	0	0	0	0	0	11	0	Bacteria	Actinobacter	Rubrobacteri	Rubrobacter	Rubrobacteri	Rubrobacter
ASV_1374	2	1	2.67	1.67	0	0	2.5	0.33	0	3	0	0	Bacteria	Actinobacter	Rubrobacteri	Rubrobacter	Rubrobacteri	Rubrobacter
ASV_1375	0.33	0	0	0	0	3.33	6	0	0	0	0	0	Bacteria	Bacteroidota	Bacteroidia	Bacteroidales	Prevotellacea	Prevotella
ASV_1376	0.67	0	0	0	2	0	0.5	2	0	0	0	0	Bacteria	Acidobacteri	Vicinamibact	Vicinamibact	Vicinamibact	NA
ASV_1377	5.67	0	0.67	0	0	0	0	0	0	0	2	0	Bacteria	Deinococcota	Deinococci	Thermales	Thermaceae	Meiothermus
ASV_1378	1.33	0	0.67	0.33	0.33	0.67	1.5	0.33	0	0	0	0.33	Bacteria	Actinobacter	Actinobacter	Propionibact	Nocardioidac	Nocardioides
ASV_1379	0.33	3	1.67	3.67	0	0	0	0	0	0	0	0	Archaea	Crenarchaeo	Thermoprote	Sulfolobales	Sulfolobacea	Stygiolobus
ASV_1380	0	0	9.33	0	0	0	0	0	0	0	0	0	Bacteria	Proteobacter	Gammaprote	Pseudomona	Pseudomona	Pseudomonas
ASV_1381	0	0.67	0.67	0.33	0.67	1	1.5	0.33	0	0	0	1.33	Bacteria	Proteobacter	Alphaproteol	Sphingomona	Sphingomona	Sphingomonas
ASV_1382	0	8.67	0	0	0	0.33	0	0	0	0	0	0	Bacteria	Chloroflexi	Chloroflexia	Thermomicrc	JG30-KF-CM4	NA
ASV_1383	0	0	0	0	0.33	8	1	0	0	0	0	0	Bacteria	Nitrospirota	Leptospirillia	Leptospirillal	Leptospirillac	Leptospirillum
ASV_1384	2	0.33	1	0	0.33	0	0	0	0	0	0	0	Bacteria	Actinobacter	Thermoleoph	Solirubrobact	67-14	NA
ASV_1385	0.67	0	2	0	1.33	0	0.5	0	2	0	2	0	Bacteria	Proteobacter	Alphaproteol	Sphingomona	Sphingomona	NA
ASV_1386	0.33	0	0	0	0	0	0.5	0	0	0	20	0	Bacteria	Deinococcota	Deinococci	Thermales	Thermaceae	Meiothermus
ASV_1387	0.33	1.67	0	0.33	0.67	2.67	7	0	0	0	0	0	Bacteria	Firmicutes	Negativicutes	Veillonellales	Veillonellacea	Dialister
ASV_1388	0	0	0	0	2	3.67	1.5	0.67	0	0	0	0	Archaea	Thermoplasn	Thermoplasn	Thermoplasn	Thermoplasn	A-plasma
ASV_1389	0	0	0	0	0	0	0.5	0	0	0	0	12.67	Bacteria	Firmicutes	Bacilli	Bacillales	Bacillaceae	NA
ASV_1390	0	0	0	0	0	0	0.5	0	0	0	0	14.67	Bacteria	Firmicutes	Negativicutes	Veillonellales	Veillonellacea	Veillonella
ASV_1391	0.33	0	0	2	0.33	1.33	2.5	1	0	0	0.33	1	Bacteria	Actinobacter	Thermoleoph	Gaiellales	Gaiellaceae	Gaiella
ASV_1392	0	0	1.33	0	1.67	0	2	0.33	2	0	1	0	Bacteria	Actinobacter	Thermoleoph	Solirubrobact	67-14	NA
ASV_1393	0	0	7.33	0	0	0	0	0	0	0	0	0	Bacteria	Proteobacter	Alphaproteol	Rhizobiales	Beijerinckiace	Methylobacterium-Methylorubrum
ASV_1394	0	0	0	0	0	0	0	0	0	0	0	0	Bacteria	Firmicutes	Sulfobacillia	Sulfobacillale	Sulfobacillace	NA

ASV_1395	0	0	0	0	0	0	0	0	0	0	0	0	Bacteria	Chloroflexi	TK10	NA	NA	NA
ASV_1396	2.67	0	0.67	0.67	0	0	0	0.33	0	0	0.67	1.33	Bacteria	Actinobacter	Acidimicrobii	IMCC26256	NA	NA
ASV_1397	1	0	0.67	1	0	0.33	2	1.67	0	0	0	0	Bacteria	Actinobacter	Thermoleoph	Gaiellales	Gaiellaceae	Gaiella
ASV_1398	1.33	1	1.67	1.67	0	0.33	0	0	0	0	0	0	Archaea	Crenarchaeo	Thermoprote	Sulfolobales	Sulfolobacea	Stygiolobus
ASV_1399	0	0	0.67	0	0.67	0	3.5	0	3	0	0.67	0	Bacteria	Actinobacter	Actinobacter	Euzebyales	Euzebyaceae	NA
ASV_1400	0	0	0	0	0	0	3.5	0	0	0	17	0.67	Bacteria	Bacteroidota	Bacteroidia	Chitinophaga	Chitinophaga	Taibaiella
ASV_1401	6.33	0	0	0	0	0	0	0	0	0	0	0	Bacteria	Proteobacter	Gammaprote	Pseudomona	Pseudomona	Pseudomonas
ASV_1402	1.33	2.67	0.67	0.33	0	0	1.5	0	0	0	0	0.67	Bacteria	Proteobacter	Gammaprote	Burkholderia	A21b	NA
ASV_1403	0.67	0.33	0.67	0	0	1.67	0.5	1.33	0	2	0	0	Bacteria	Verrucomicro	Verrucomicro	Chthoniobact	Chthoniobact	Chthoniobacter
ASV_1404	0.67	0	0.67	0.67	0	1.67	3.5	1.67	0	0	0	0.67	Bacteria	Actinobacter	Actinobacter	Propionibact	Nocardioidac	NA
ASV_1405	0	4.33	0.33	0	0.33	0	0	3.33	0	0	0	0.67	Bacteria	Proteobacter	Alphaproteol	Sphingomona	Sphingomona	MN 122.2a
ASV_1406	0	10.67	0	0	0	0	0	0	0	0	0	0	Bacteria	Proteobacter	Gammaprote	Burkholderia	Comamonada	Schlegelella
ASV_1407	0	0	0	0	0	0	0	0	0	0	6.33	0	Bacteria	Actinobacter	Thermoleoph	Solirubrobact	67-14	NA
ASV_1408	0.33	0	2.33	1.67	0	0	0.5	0	0	6	0.67	0	Bacteria	Planctomyce	Phycisphaera	Tepidisphaer	WD2101 soil	NA
ASV_1409	6	0.33	0	0	0	0	0	0	0	0	0	0	Archaea	Crenarchaeo	Thermoprote	Sulfolobales	Sulfolobacea	Stygiolobus
ASV_1410	0.67	0	7.33	0	0	0	1	0	0	0	1.33	0	Bacteria	Firmicutes	Bacilli	Bacillales	Bacillaceae	Ornithinibacillus
ASV_1411	0	0	0	0	0	0	3	0	0	0	20.67	0	Bacteria	Proteobacter	Gammaprote	Burkholderia	Comamonada	NA
ASV_1412	0	0	0	0	0	0	0	0	0	0	0	0	Bacteria	Bacteroidota	Bacteroidia	Chitinophaga	NA	NA
ASV_1413	0	0	0	0	0	0	0	0	0	0	0	10.67	Bacteria	Proteobacter	Gammaprote	Pseudomona	Moraxellacea	Acinetobacter
ASV_1415	1.67	1	0	0.67	0.33	0.33	1	0	0	0	0	0.67	Bacteria	Actinobacter	Thermoleoph	Solirubrobact	67-14	NA
ASV_1416	0	0	0	0	0	0	0	0	0	0	0	0	Bacteria	NA	NA	NA	NA	NA
ASV_1417	3.67	0	0	0	0	0	0	0	0	0	0	0	Archaea	Crenarchaeo	Thermoprote	Sulfolobales	Sulfolobacea	Stygiolobus
ASV_1418	0	0	0	0	0	0	0	0	0	0	0	0	Bacteria	Chloroflexi	Ktedonobact	Ktedonobact	Ktedonobact	FCPS473
ASV_1419	0	0	0	0	0	0	0	10	0	0	0	0	Bacteria	Proteobacter	Gammaprote	Burkholderia	Neisseriaceae	Neisseria
ASV_1420	0	0	0	0	0	0	0	0	0	0	13.67	0	Bacteria	Bacteroidota	Bacteroidia	Sphingobacte	Sphingobacte	Mucilaginibacter
ASV_1421	0.33	1.33	1.67	0	0	0	2	1.33	0	0	0	0	Bacteria	Chloroflexi	TK10	NA	NA	NA
ASV_1422	0.67	0	0	1	0.33	1	2.5	1	0	0	0	0.67	Bacteria	Actinobacter	Actinobacter	Frankiales	NA	NA
ASV_1423	0	0	0	0	3	0	0	0	0	0	6.33	0	Bacteria	Actinobacter	Thermoleoph	Solirubrobact	67-14	NA
ASV_1424	0	5	1	0	0	0	0	2.67	0	0	0.33	0.67	Bacteria	Actinobacter	Thermoleoph	Solirubrobact	Solirubrobact	NA
ASV_1425	0	0	0	0	0	0	0	0	0	0	0	0	Bacteria	Chloroflexi	OLB14	NA	NA	NA
ASV_1426	5.33	0	0	0	0	0	0	0	0	0	0	0	Bacteria	Actinobacter	Rubrobacteri	Rubrobactera	Rubrobacteri	Rubrobacter
ASV_1427	0	4.33	1	0.33	0	0.33	0.5	1.67	0	0	0.33	1	Bacteria	Proteobacter	Alphaproteol	Sphingomona	Sphingomona	MN 122.2a
ASV_1428	1	0.33	0	0	0.33	1.33	2	1	0	0	0.67	0.67	Bacteria	Actinobacter	Actinobacter	Frankiales	NA	NA
ASV_1429	0.33	0	0.33	1	1.33	1	2.5	1.67	0	0	0.33	0	Bacteria	Patescibacter	NA	NA	NA	NA
ASV_1430	0	0	0	0	0	0	0	0	0	0	0	0	Bacteria	Firmicutes	YNPFFP2	NA	NA	NA
ASV_1431	0	0	0	4.67	0	0	0	0	0	0	0	0	Bacteria	Bacteroidota	Bacteroidia	Bacteroidales	Prevotellacea	Prevotella
ASV_1432	0	0.67	0	0	0	0	0	0	0	0	0	0	Bacteria	Actinobacter	Actinobacter	Frankiales	Sporichthyac	Sporichthya
ASV_1433	0	0	0	0	0	0	0	0.67	0	2	9.33	0	Bacteria	Actinobacter	Thermoleoph	Solirubrobact	67-14	NA
ASV_1434	0	0	0	0	0	0	0	0	0	0	11	0	Bacteria	Patescibacter	Saccharimon	Saccharimon	NA	NA
ASV_1435	2	0	0	1.33	0	0.33	0.5	0	0	0	0.33	0	Bacteria	Actinobacter	Actinobacter	NA	NA	NA
ASV_1436	0.67	0.33	0.33	0.67	0.67	0.33	1	0	0	0	0.67	0.67	Bacteria	Actinobacter	Thermoleoph	Solirubrobact	67-14	NA
ASV_1437	4	0	2	0	0	0	0	0	0	0	0	0	Bacteria	Proteobacter	Gammaprote	Pseudomona	Pseudomona	Pseudomonas
ASV_1438	7	0	0	0	0	0	0	0	0	0	0	0	Bacteria	Firmicutes	Bacilli	Staphylococc	Staphylococc	Staphylococcus
ASV_1439	0	0	0	0	0	0	0	0	0	90	0	0	Bacteria	Actinobacter	Thermoleoph	Solirubrobact	Solirubrobact	NA
ASV_1440	0	0	0	0	0	0	0	0	0	0	0	0	Bacteria	Firmicutes	NA	NA	NA	NA
ASV_1441	5.67	0	0	0	0	0.33	0	0	0	0	0	0	Bacteria	Acidobacteri	Vicinamibact	Vicinamibact	Vicinamibact	NA
ASV_1442	1	0	1	1.33	0	0.67	2	0	0	0	0	0.67	Bacteria	Actinobacter	Thermoleoph	Solirubrobact	67-14	NA
ASV_1443	0.33	0	2.67	0.33	0	0.33	2	0	0	0	0	0.33	Bacteria	Patescibacter	Saccharimon	Saccharimon	NA	NA
ASV_1444	0	0	0	0	0	0	0.5	0	0	0	0	7	Bacteria	Firmicutes	Bacilli	Bacillales	Bacillaceae	NA
ASV_1445	0	0	0	0	0	0	0	0	0	2	0	0	Bacteria	Actinobacter	Thermoleoph	Solirubrobact	67-14	NA
ASV_1447	0	0	0	0	0	0	0	0	0	0	9.33	0	Bacteria	Firmicutes	Bacilli	Staphylococc	Staphylococc	Staphylococcus
ASV_1448	0.67	0.67	1	0.67	0	0.33	0.5	0.33	0	0	0	0.67	Bacteria	Actinobacter	Acidimicrobii	NA	NA	NA
ASV_1449	0.33	0	2	0	1	0	1.5	0	1	0	0.67	0	Bacteria	Actinobacter	Rubrobacteri	Rubrobactera	Rubrobacteri	Rubrobacter
ASV_1450	0.67	0	0.67	0	0.33	0.67	3	1	0	0	1	0.67	Bacteria	Acidobacteri	Vicinamibact	Vicinamibact	Vicinamibact	NA
ASV_1451	1.67	0	0.67	1	0.67	1.33	1	0.67	0	0	0	0	Bacteria	Chloroflexi	Chloroflexia	Thermomicro	JG30-KF-CM4	NA
ASV_1452	0.67	1	1	0	0.33	0.33	1.5	1	0	4	0.67	1.67	Bacteria	Actinobacter	Actinobacter	Frankiales	NA	NA
ASV_1453	0	0	0	0	0	0	0	0	0	2	1	0	Bacteria	Actinobacter	Actinobacter	Propionibact	Propionibact	Cutibacterium
ASV_1454	0	0	0	0	0	0	0	0	0	99	0	0	Bacteria	Firmicutes	Bacilli	Thermicanale	Thermicanac	Thermicanus
ASV_1455	0	0	0	0	0	0	0	0.33	0	0	8	0	Bacteria	Actinobacter	Actinobacter	Pseudonocar	Pseudonocar	Amycolatopsis
ASV_1456	1.67	0	2	0	0	0	3.5	0	0	0	0.33	0	Bacteria	Actinobacter	Actinobacter	Euzebyales	Euzebyaceae	NA
ASV_1457	1	1.33	0.33	1.33	0	0.33	1.5	1	0	0	0	0	Archaea	Crenarchaeo	Nitrososphae	Nitrososphae	Nitrososphae	Candidatus Nitrocosmicus
ASV_1458	0.67	0	0	0.67	0.67	0.67	1	1	0	0	0	1	Bacteria	Acidobacteri	Holophagae	Subgroup 7	NA	NA
ASV_1459	0	0	0	1	0	1	2.5	2	0	0	0.33	0	Bacteria	Patescibacter	Saccharimon	Saccharimon	NA	NA
ASV_1460	2	1.33	0	0.33	0	0	0.5	0	0	0	0	0	Bacteria	Actinobacter	Actinobacter	Actinomyceta	Actinomyceta	Flaviflexus
ASV_1461	0	5.33	0	0	0.33	0	0	1.67	0	0	1	2.67	Bacteria	Actinobacter	Thermoleoph	Gaiellales	NA	NA
ASV_1462	0	0	0	0	0	0	0	0	0	0	10	0	Bacteria	Actinobacter	Actinobacter	Micrococcale	Dermacoccac	Dermacoccus
ASV_1463	0.33	0.67	0.33	1.67	0.33	0	1.5	0	0	0	0.67	0.33	Bacteria	Gemmatimor	Gemmatimor	Gemmatimor	Gemmatimor	NA
ASV_1464	1.67	0	0.33	0	0	0.33	0.5	0.67	0	0	0.67	0.33	Bacteria	Chloroflexi	KD4-96	NA	NA	NA
ASV_1465	0	0	1.33	0	2	0	4	0	0	0	4.33	0	Bacteria	Bacteroidota	Bacteroidia	Chitinophaga	Chitinophaga	Segetibacter
ASV_1466	0.67	0.67	0.67	0	0	0	0	0	0	2	1	1.67	Bacteria	Actinobacter	Actinobacter	Propionibact	Propionibact	Cutibacterium
ASV_1467	0	0	0	0	3	3	0.5	2	0	0	0	0	Archaea	Thermoplasn	Thermoplasn	Thermoplasn	Thermogymn	NA
ASV_1468	0	4.67	1	0	0.33	0	0	1	0	0	0	0	Bacteria	Proteobacter	Alphaproteol	Rhizobiales	Xanthobacter	Bradyrhizobium
ASV_1469	0	0	0	0	0	0	0	0	0	0	0	0	Bacteria	Actinobacter	Acidimicrobii	NA	NA	NA
ASV_1470	0	0	0	0	0	0	0	0	0	0	0	0	Bacteria	Bacteroidota	Bacteroidia	Flavobacteria	Weeksellacea	Wautersiella
ASV_1471	1.67	0.33	0.67	1.33	0.33	0	0.5	0	0	0	0	1	Bacteria	Actinobacter	Thermoleoph	Solirubrobact	67-14	NA
ASV_1472	1	0	2	0.33	0	0.33	0	0	0	0	0	0	Bacteria	Actinobacter	MB-A2-108	NA	NA	NA
ASV_1473	0.67	1	0	0.33	0	1	6	0	0	0	0	0.67	Bacteria	Proteobacter	Alphaproteol	Rhizobiales	Rhizobiaceae	Brucella
ASV_1474	0.33	0	0.33	0.33	0	0.33	0	0.33	0	0	0	0.33	Bacteria	Actinobacter	Thermoleoph	Solirubrobact	67-14	NA
ASV_1475	5.33	0	0.33	0	0	0	0	0	0	0	0	0	Archaea	Crenarchaeo	Thermoprote	Sulfolobales	Sulfolobacea	Stygiolobus
ASV_1476	0	0	0	0	0	1.67	9.5	0	0	0	0.33	0	Bacteria	Proteobacter	Alphaproteol	Rhizobiales	NA	NA
ASV_1477	0	0	0	0	0	0	0	0	0	2	10.67	0.67	Bacteria	Firmicutes	Bacilli	Paenibacillale	Paenibacillac	Paenibacillus
ASV_1478	0	0	0	0	0	0	0	0	0	0	0	0	Bacteria	Firmicutes	Bacilli	Alicyclobacill	Alicyclobacill	Alicyclobacillus
ASV_1479	0	0	0	0	0	0	0	0	0	0	0	0	Bacteria	Firmicutes	Bacilli	Bacillales	Bacillaceae	Aeribacillus
ASV_1480	1.33	0	1	0	0	0	2	0	0	0	0	0	Bacteria	Firmicutes	Clostridia	Oscillospirale	Ruminococca	Ruminococcus
ASV_1481	1.67	0.33	0.33	1	0.33	0.33	0	0.67	0	0	0	0.33	Bacteria	Bacteroidota	Bacteroidia	Chitinophaga	NA	NA
ASV_1482	0	0	0	0	2	0	2.5	5	0	0	0	0.33	Bacteria	Actinobacter	Actinobacter	Corynebacter	Mycobacteria	Mycobacterium
ASV_1483	7.33	0	0	0	0	0	0	0	0	0	0	0	Bacteria	Gemmatimor	S0134 terrest	NA	NA	NA
ASV_1484	1	0.33	3	0	0.33	0	0	0	0	0	0.67	0	Bacteria	Actinobacter	Thermoleoph	Solirubrobact	67-14	NA
ASV_1485	3.67	0	2.67	0.67	0	0	0	0	0	0	0	0	Bacteria	Proteobacter	Gammaprote	Pseudomona	Pseudomona	Pseudomonas
ASV_1486	1	0	1.33	0.67	0	0	2	0.33	0	0	0	0	Bacteria	Chloroflexi	Chloroflexia	Thermomicro	JG30-KF-CM4	NA
ASV_1487	0.67	0	1.67	0	0.33	0.33	1.5	0.67	0	0	0	0	Bacteria	Chloroflexi	Gitt-GS-136	NA	NA	NA
ASV_1488	1.33	0	0.67	0	0.33	1.33	1.5	1	0	0	0.33	0.67	Bacteria	Actinobacter	Actinobacter	Propionibact	Nocardioidac	Nocardioides
ASV_1489	0	0	0	0	0	0	0	0	0	0	0	0	Bacteria	Firmicutes	Sulfobacillia	Sulfobacillale	Sulfobacillace	NA
ASV_1490	0	0	0	0	0	0	0	0	0	0	0	0	Bacteria	Bacteroidota	Bacteroidia	Flavobacteria	Weeksellacea	Cloacibacterium
ASV_1491	0.33	0	0.67	0	0	0.33	0	1.33	0	0	0	1	Bacteria	Bacteroidota	Bacteroidia	Sphingobacte	KD3-93	NA
ASV_1492	1.33	0	1.33	0	0	0	0	1	0	0	0.67	1.33	Bacteria	Gemmatimor	Longimicrobi	Longimicrobi	Longimicrobi	YC-ZSS-LKJ147
ASV_1493	0	0.33	0	1	0	0	0	0	0	0	0	1.67	Bacteria	Actinobacter	Actinobacter	Micrococcale	Dermabacter	Brachybacterium
ASV_1494	0	0	0	0	0.33	0	0	0	65	0	0	0	Bacteria	Actinobacter	Thermoleoph	Gaiellales	NA	NA
ASV_1495	0.33	0.33	0.33	0	1.33	0	0.5	1	0	0	0.33	0.33	Bacteria	Acidobacteri	Vicinamibact	Vicinamibact	Vicinamibact	NA
ASV_1496	0	0	0	0	0	0	0	9.33	0	0	0	0	Bacteria	NA	NA	NA	NA	NA
ASV_1497	1.33	0.33	0	1	0.67	1	2	0.67	0	0	0	1.33	Bacteria	Patescibacter	NA	NA	NA	NA
ASV_1498	0.33	0	1	0	2	0	1.5	0	0	0	2	0	Bacteria	Proteobacter	Gammaprote	Pseudomona	Pseudomona	Pseudomonas
ASV_1499	0.33	0	0	0.33	0.67	0.33	2	0.33	0	0	0.67	0	Bacteria	Myxococcota	Polyangia	Haliangiales	Haliangiaceae	Haliangium
ASV_1500	0	0	0	0	0	0	0	0	0	0	8.67	0	Bacteria	Proteobacter	Gammaprote	Enterobacter	Enterobacter	NA
ASV_1501	0	0	7.33	0	0	0	1	0	0	0	0	0	Bacteria	Proteobacter	Alphaproteol	Rhizobiales	Rhizobiaceae	Allorhizobium-Neorhizobium-Pararhizobium-Rhizobium
ASV_1502	0	0	0	0	0	0	0	0	0	0	0	0.67	Bacteria	Proteobacter	Gammaprote	Pseudomona	Moraxellacea	Acinetobacter
ASV_1503	0	0	0	0	0	0	0	0	0	0	0	7	Bacteria	Actinobacter	MB-A2-108	NA	NA	NA
ASV_1504	2.67	0	0.67	0	0	0	0	1	0	0	0.33	0.67	Bacteria	Nitrospirota	Nitrospiria	Nitrospirales	Nitrospiracea	Nitrospira
ASV_1505	1.33	0	0.33	1.33	0	1	1.5	0.33	0	0	0.33	0	Bacteria	Actinobacter	Actinobacter	0319-7L14	NA	NA
ASV_1506	0.67	0	0.33	0	0.33	0	0	0.33	0	0	0	0	Bacteria	Planctomyce	Planctomyce	Pirellulales	Pirellulaceae	Pirellula
ASV_1507	0.67	0	0	0	0.67	0.33	1.5	0.67	0	0	1	0.33	Bacteria	Actinobacter	Thermoleoph	Solirubrobact	67-14	NA
ASV_1508	0.33	0	0	1	1	1	2	1.33	0	0	0.33	0	Bacteria	Actinobacter	Actinobacter	Propionibact	Nocardioidac	Marmoricola
ASV_1509	0.67	0	1	0.67	0.33	1.33	0.5	0.67	0	0	0	0.67	Bacteria	Actinobacter	Thermoleoph	Gaiellales	Gaiellaceae	Gaiella
ASV_1510	0	0	0	0	0	0	0	0	0	0	0	0	Archaea	Crenarchaeo	Nitrososphae	Group 1.1c	NA	NA
ASV_1511	0	0	0	0	0	0	0	0	0	0	11	0	Bacteria	Actinobacter	Thermoleoph	Solirubrobact	67-14	NA
ASV_1512	1.33	1	0	1.67	0	0.67	0.5	0	0	0	0	2.67	Bacteria	Proteobacter	Gammaprote	Xanthomona	Xanthomona	Arenimonas
ASV_1513	1	0	1	1.33	0.33	0.33	0.5	1	0	0	0.33	0.33	Bacteria	Actinobacter	Thermoleoph	Solirubrobact	Solirubrobact	Solirubrobacter
ASV_1514	0	0	0	0.33	0	0.33	0.5	1.33	0	0	0	0	Bacteria	Actinobacter	Acidimicrobii	Microtrichale	Iamiaceae	Iamia
ASV_1515	0	0	0	0.33	0.33	0.33	2.5	0.67	0	0	0	0.33	Bacteria	Verrucomicro	Verrucomicro	Verrucomicro	Verrucomicro	NA
ASV_1516	0	0	0	0	0	0	0	0	0	0	0	0	Bacteria	Actinobacter	Thermoleoph	Gaiellales	NA	NA
ASV_1517	0	0	0	0	0	0	1	0	0	0	0.33	12.33	Bacteria	Firmicutes	Bacilli	Lactobacillale	Aerococcacea	Aerococcus
ASV_1518	0	0	0	0	0	0	0	0	0	0	0	0	Bacteria	Actinobacter	Actinobacter	Corynebacter	Corynebacter	Corynebacterium
ASV_1519	0.67	0	0.67	0	1	0	3.5	0	0	0	0.67	0.67	Bacteria	Actinobacter	Actinobacter	Euzebyales	Euzebyaceae	NA
ASV_1520	0	0.33	0	0	0	0	0	0	0	0	0	0.33	Bacteria	Actinobacter	Actinobacter	Frankiales	NA	NA
ASV_1521	5	0	0	0	0	0	0	0	0	0	0	0	Archaea	Crenarchaeo	Thermoprote	Sulfolobales	Sulfolobacea	Stygiolobus
ASV_1522	0	0	0	5.67	0	0	0	0	0	0	0	0	Bacteria	Acidobacteri	Acidobacteria	Solibacterales	Solibacterace	Candidatus Solibacter
ASV_1523	0	0	0	0	0	0	0	0	0	0	0	0	Bacteria	Firmicutes	Bacilli	Bacillales	Bacillaceae	Geobacillus
ASV_1524	0	0	0	5.67	0	0	0	0	0	0	0	0	Bacteria	Proteobacter	Gammaprote	Burkholderia	Comamonada	Curvibacter
ASV_1525	0.67	0	0.67	0.67	0	0.67	0.5	0.67	0	0	0.33	0	Bacteria	Proteobacter	Gammaprote	Xanthomona	Xanthomona	Lysobacter
ASV_1526	0	0	2.33	0	1.67	0	1.5	0	2	0	0	0	Bacteria	Actinobacter	Thermoleoph	Solirubrobact	Solirubrobact	Parviterribacter
ASV_1527	0	0	0	0	0	0	0	0	0	0	0	0	Bacteria	Acidobacteri	Acidobacteria	Acidobacteria	Koribacterace	Candidatus Koribacter
ASV_1528	4.33	0.33	0.67	0	0	0	0.5	0	0	0	0	0	Archaea	Crenarchaeo	Thermoprote	Sulfolobales	Sulfolobacea	Stygiolobus
ASV_1529	4.67	0	1	0	0	0	0	0.33	0	0	0	0	Archaea	Crenarchaeo	Thermoprote	Sulfolobales	Sulfolobacea	Stygiolobus
ASV_1530	0	0	7	0	0	0	1.5	0	0	0	0.33	0	Bacteria	Proteobacter	Gammaprote	Pseudomona	Pseudomona	Pseudomonas
ASV_1531	0	0	0	0	0	0	0	0	0	0	4.33	0	Bacteria	Armatimonac	NA	NA	NA	NA
ASV_1532	0	0	0	0	0	0.33	0	0	0	0	0	0	Bacteria	Firmicutes	Sulfobacillia	Sulfobacillale	Sulfobacillace	NA
ASV_1533	0	0	0	0	0	0	0	0.33	0	0	7.67	0	Bacteria	Actinobacter	Actinobacter	Propionibact	Nocardioidac	Nocardioides
ASV_1534	0.33	0.67	1.33	0	0	1	1	0.33	0	0	0.33	1	Bacteria	Myxococcota	Polyangia	Haliangiales	Haliangiaceae	Haliangium
ASV_1535	0.33	0	0	0	0	1	3.5	0.67	0	0	0	0	Bacteria	Myxococcota	Polyangia	Haliangiales	Haliangiaceae	Haliangium
ASV_1536	0	0	0	0	0	0	0	0	0	0	0	0	Bacteria	Firmicutes	Bacilli	Alicyclobacill	Alicyclobacill	Acidibacillus
ASV_1537	1.33	0	1.33	0	0	0	0	0	0	0	0.33	0	Bacteria	Actinobacter	Acidimicrobii	IMCC26256	NA	NA
ASV_1538	0	0	0	0.67	2.67	0.33	5	0	0	0	2.33	0	Bacteria	Abditibacteri	Abditibacteri	Abditibacteri	Abditibacteri	Abditibacterium
ASV_1539	0	0	0	0	0	5.33	0	0	0	0	0	0	Bacteria	Firmicutes	Sulfobacillia	Sulfobacillale	Sulfobacillace	NA
ASV_1540	5	0.33	1	0	0	0	0	0	0	0	0	0	Archaea	Crenarchaeo	Thermoprote	Sulfolobales	Sulfolobacea	Stygiolobus
ASV_1541	0	0	0	0	0	0	0	0	0	0	0	6.33	Bacteria	Firmicutes	Bacilli	Staphylococc	Staphylococc	Staphylococcus
ASV_1542	0	0	6	0	0	0	1.5	0	0	0	0	0.33	Bacteria	Firmicutes	Clostridia	Oscillospirale	Ethanoligene	Ethanoligenens
ASV_1543	0.67	0	2	0	0	0.33	1	0.33	0	0	0	0	Bacteria	Chloroflexi	TK10	NA	NA	NA

ASV_1544	0.67	0.33	1	0.67	0	0.33	0	1.33	0	0	0.33	0	Bacteria	Sumerlaeota	Sumerlaeia	Sumerlaeales	Sumerlaeace	Sumerlaea
ASV_1545	0.33	0.67	0.33	0.33	0.67	0.67	0.5	0	0	0	0	0	Bacteria	Patescibacter	Saccharimon	Saccharimon	NA	NA
ASV_1546	0.33	0	0.67	0.67	0.33	1.67	2.5	0.33	0	0	0	0.33	Bacteria	Nitrospirota	Nitrospiria	Nitrospirales	Nitrospiracea	Nitrospira
ASV_1547	0	0	0	0	0	0	0	0	0	0	0	0	Bacteria	Actinobacter	Actinobacter	0319-7L14	NA	NA
ASV_1548	6.67	0	0	0	0	0	0	0	0	0	0	0	Bacteria	Firmicutes	Bacilli	Staphylococc	Staphylococc	Staphylococcus
ASV_1549	0	0	0	0	0	0	0	0	0	0	0	0	Bacteria	NA	NA	NA	NA	NA
ASV_1550	1	0	0.67	0	0	0	0	0.33	0	0	1	1	Bacteria	Actinobacter	Actinobacter	Euzebyales	Euzebyaceae	NA
ASV_1551	1.33	2.33	1	1.33	0	1	1	1.33	0	0	0	0	Bacteria	Planctomycet	Planctomycet	Pirellulales	Pirellulaceae	Pirellula
ASV_1552	0.33	0	0	0	0.67	1	1	0.33	0	0	0	0.33	Bacteria	Actinobacter	Acidimicrobii	NA	NA	NA
ASV_1553	0	4.67	0.67	0	0	0	0	1.33	0	0	0.67	0	Bacteria	Proteobacter	Alphaproteol	Rhizobiales	Beijerinckiace	Psychroglaciecola
ASV_1554	0	0	0	0	0	0	0	3	0	0	0	0	Bacteria	Actinobacter	Acidimicrobii	Acidimicrobia	Acidimicrobia	Ferrimicrobium
ASV_1555	0	0	0	0	0	0	0	0.33	0	0	8.33	0	Bacteria	Actinobacter	Actinobacter	Euzebyales	Euzebyaceae	NA
ASV_1556	0.67	0	2	0	0	0	0	0	1	0	0	0	Bacteria	Actinobacter	Thermoleoph	Solirubrobact	67-14	NA
ASV_1557	0	1	0.33	0.33	0	1.33	2	0	0	0	0	0.67	Bacteria	Patescibacter	Saccharimon	Saccharimon	NA	NA
ASV_1558	0.67	0	0	0	1.33	1.67	1.5	1	0	0	0.33	0.67	Bacteria	Chloroflexi	KD4-96	NA	NA	NA
ASV_1559	1	0	0.67	0.67	1	0.67	1.5	0	0	0	1.33	0	Bacteria	Proteobacter	Alphaproteol	Rhizobiales	Beijerinckiace	NA
ASV_1560	0	3.33	0	0	0	0	0	1	0	0	0	0	Bacteria	Proteobacter	Gammaprote	Burkholderia	Alcaligenacea	Achromobacter
ASV_1561	0	0	0	0	0	0	0	0	0	0	0	0	Bacteria	Firmicutes	Sulfobacillia	Sulfobacillale	Sulfobacillace	NA
ASV_1562	0.33	0	0	0	0.67	0	0.5	0.33	0	0	0	1	Bacteria	Actinobacter	Actinobacter	Frankiales	NA	NA
ASV_1563	1	0.33	0	0	0	0	0.5	0.33	0	0	0.33	0.33	Bacteria	Proteobacter	Gammaprote	Burkholderia	Burkholderia	Lautropia
ASV_1564	0.67	0	0	0.67	0.33	1	3	0	0	0	0	0.67	Bacteria	Actinobacter	Thermoleoph	Solirubrobact	Solirubrobact	Solirubrobacter
ASV_1565	0	0.33	0.33	0.67	0.67	0	1	0.33	0	0	0.33	0.67	Bacteria	Acidobacteri	Acidobacteria	Bryobacteral	Bryobacterac	Bryobacter
ASV_1566	0.33	0.67	0	2	0.33	0.33	1	0	0	0	0	0.33	Bacteria	Actinobacter	Thermoleoph	NA	NA	NA
ASV_1567	0	0.67	0.33	0.67	0.67	0.33	1.5	1.33	0	0	0	0.33	Bacteria	Verrucomicro	Verrucomicro	Chthoniobact	Chthoniobact	Chthoniobacter
ASV_1568	0	0	0	0	8.33	0	0	0	0	0	0	0	Bacteria	NA	NA	NA	NA	NA
ASV_1569	0	0.67	0	1	0.67	0	0	0	0	0	0	0	Bacteria	Acidobacteri	Blastocatellia	Blastocatellal	Blastocatellac	Blastocatella
ASV_1570	0	0	7.33	0	0	0	0	0	0	0	0	0	Bacteria	Proteobacter	Alphaproteol	Rhizobiales	Beijerinckiace	Bosea
ASV_1571	0	0	0	0	0	0	0	0	0	0	0	0	Bacteria	Actinobacter	Acidimicrobii	NA	NA	NA
ASV_1572	0.67	0	0.33	0	0	0	0	1	0	0	0	0.33	Bacteria	Patescibacter	Saccharimon	Saccharimon	LWQ8	NA
ASV_1573	1.67	0	1.67	0	0	0	0	0	1	0	0.33	0	Bacteria	Firmicutes	Bacilli	Thermicanale	Thermicanac	Thermicanus
ASV_1574	1.67	0	0.33	1	0.67	0	0	0.67	0	0	0.33	0.33	Bacteria	Acidobacteri	Vicinamibact	Vicinamibact	NA	NA
ASV_1575	3.67	0	0	0	0	0	0	0	0	0	0	0	Bacteria	Deinococcota	Deinococci	Thermales	Thermaceae	Meiothermus
ASV_1576	0	0	0	0	0	0	0	0	0	0	0	6.33	Bacteria	Firmicutes	Bacilli	Staphylococc	Staphylococc	Staphylococcus
ASV_1577	0	0	0	0	0	0	0	0	0	0	0	0	Bacteria	Actinobacter	Actinobacter	0319-7L14	NA	NA
ASV_1578	0	0	8	0	0	0	0.5	0	0	0	0	0.33	Bacteria	Firmicutes	Sulfobacillia	Sulfobacillale	Sulfobacillace	NA
ASV_1579	2.33	0.67	0	0.33	0	0	0	0	0	0	0	0.67	Bacteria	Chloroflexi	KD4-96	NA	NA	NA
ASV_1580	1	0	0	0	0.67	0.33	0	0	2	3	1.67	0	Bacteria	Actinobacter	Actinobacter	Pseudonocar	Pseudonocar	NA
ASV_1581	0.33	1	1	1.33	0	0	0	0	0	0	0	0	Archaea	Crenarchaeo	Thermoprote	Sulfolobales	Sulfolobacea	Stygiolobus
ASV_1582	5	0.67	0	0	0	0	0	0	0	0	0	0	Archaea	Crenarchaeo	Thermoprote	Sulfolobales	Sulfolobacea	Stygiolobus
ASV_1583	0	0	0	0	0	1	8.5	0	2	0	0.33	0	Bacteria	Proteobacter	Alphaproteol	Sphingomona	Sphingomona	NA
ASV_1584	0	5.67	0	0	0	0	0	0	0	0	0	0	Bacteria	Actinobacter	Actinobacter	Micrococcale	Microbacteri	Microbacterium
ASV_1585	0	0	0	0	3.67	4	0	0	0	0	0	0	Bacteria	Actinobacter	Acidimicrobii	NA	NA	NA
ASV_1586	0	0	0	0	0	0	0	0	0	0	0	7.67	Bacteria	Actinobacter	Actinobacter	Actinomyceta	Actinomyceta	Actinomyces
ASV_1587	0	0	0	0	0	0	0	0	0	0	0	0	Bacteria	Actinobacter	Acidimicrobii	NA	NA	NA
ASV_1588	0	0	0.33	0	0	0	0	0	3	0	1	0	Bacteria	Planctomycet	Planctomycet	Pirellulales	Pirellulaceae	NA
ASV_1589	1.67	0.67	0.33	1	0	0.33	0.5	1	0	0	0	0.67	Bacteria	Planctomycet	Planctomycet	Pirellulales	Pirellulaceae	Pirellula
ASV_1590	0	0.67	0.67	0	0	0	1	0	0	0	0.33	0	Bacteria	Firmicutes	Bacilli	Staphylococc	Staphylococc	NA
ASV_1591	0	0	0	0	0.67	7.33	0	0	0	0	0	0	Bacteria	Firmicutes	Sulfobacillia	Sulfobacillale	Sulfobacillace	NA
ASV_1592	0	0	0.33	0	0	0	1	0	0	0	18.33	0	Bacteria	Proteobacter	Gammaprote	Enterobacter	Enterobacter	NA
ASV_1593	0.33	0	2	0.33	0.33	0	0	0	0	0	0	0	Bacteria	Acidobacteri	Blastocatellia	Pyrinomonad	Pyrinomonad	RB41
ASV_1594	0	0	0	0	0	0	0	0	0	0	0	0	Bacteria	Actinobacter	Rubrobacteri	Rubrobactera	Rubrobacteri	Rubrobacter
ASV_1595	0	0	0	0	1	0.67	3.5	2	0	0	0	0	Bacteria	Actinobacter	Actinobacter	Frankiales	Acidotherma	Acidothermus
ASV_1596	8.33	0	0	0	0	0	0	0	0	0	0	0	Bacteria	Proteobacter	Gammaprote	Pseudomona	Cellvibrionac	Cellvibrio
ASV_1597	4.33	0	0.67	0	0	0	0	0	0	0	0	0	Bacteria	Proteobacter	Alphaproteol	Rhizobiales	Beijerinckiace	Methylobacterium-Methylorubrum
ASV_1598	0	0.67	0	0	0	0	0	0	0	39	0	0	Bacteria	Firmicutes	Bacilli	Thermicanale	Thermicanac	Thermicanus
ASV_1599	0	0	0	0	2.33	3.67	0	0	0	0	0	0	Bacteria	Firmicutes	Sulfobacillia	Sulfobacillale	Sulfobacillace	Sulfobacillus
ASV_1600	0	0	0	0	0.67	1.67	0	0	0	0	0	0	Bacteria	Firmicutes	Bacilli	Alicyclobacill	Alicyclobacill	Acidibacillus
ASV_1601	0	0	0	0	0.33	0	0	0	0	0	0	0	Bacteria	Actinobacter	Actinobacter	NA	NA	NA
ASV_1602	0	0	0	0	0	0	0	1	0	3	4.67	0.33	Bacteria	Chloroflexi	Chloroflexia	Kallotenuales	Kallotenuace	Kallotenue
ASV_1603	0	0	0	0	0	0	0	0.33	0	0	7	0	Bacteria	Actinobacter	Rubrobacteri	Rubrobactera	Rubrobacteri	Rubrobacter
ASV_1604	0	1	0	0	0	0	0	0	0	0	0	0	Bacteria	Firmicutes	Bacilli	Bacillales	Bacillaceae	Anoxybacillus
ASV_1605	5	0	0	0	0	0	0	0	0	0	0	0	Bacteria	Gemmatimor	S0134 terrest	NA	NA	NA
ASV_1606	1	0.67	0	0.33	0	0	0.5	0	0	2	0	0.33	Bacteria	Acidobacteri	Holophagae	Subgroup 7	NA	NA
ASV_1607	0.33	1.33	1	0.33	0	1	0.5	0	0	0	0	0	Bacteria	Actinobacter	Actinobacter	Propionibact	Nocardioidac	Nocardioides
ASV_1608	0	0.33	0.67	0	1	0.67	0	0.33	0	2	0	0.33	Bacteria	Bacteroidota	Bacteroidia	Chitinophaga	Chitinophaga	Segetibacter
ASV_1609	4.67	0	2	0	0	0	0	0	0	0	0	0	Archaea	Crenarchaeo	Thermoprote	Sulfolobales	Sulfolobacea	Stygiolobus
ASV_1610	0	0	0	1	0	0.33	3	0.67	0	0	0	1.67	Bacteria	Actinobacter	Actinobacter	Frankiales	NA	NA
ASV_1611	0.67	0.33	0.33	1.33	0	0	0	0	0	0	0	0	Bacteria	Planctomycet	Phycisphaera	Tepidisphaer	WD2101 soil	NA
ASV_1612	0	0	0	0	0	0	0	0	0	0	0	0	Bacteria	Actinobacter	Actinobacter	Pseudonocar	Pseudonocar	NA
ASV_1613	0	0	0	0	0	0	0	0	0	0	5.67	0	Bacteria	Actinobacter	Actinobacter	Euzebyales	Euzebyaceae	NA
ASV_1614	0	0	0	0	0	0	0	0	0	0	0	0	Bacteria	Actinobacter	Actinobacter	Euzebyales	Euzebyaceae	NA
ASV_1615	1.33	0	0	0	0	0.33	1	0	0	0	0	0	Bacteria	Proteobacter	Gammaprote	Xanthomona	Xanthomona	Lysobacter
ASV_1616	0.67	0	0.33	0	0.67	0.67	1.5	0.67	0	0	0.67	0	Bacteria	Actinobacter	Thermoleoph	Solirubrobact	67-14	NA
ASV_1617	0	0	0	1.33	1	0.33	0.5	0.33	0	0	0.33	0.67	Bacteria	Actinobacter	Thermoleoph	Solirubrobact	67-14	NA
ASV_1618	5	0	0	0	0	0	0	0	0	0	0	0	Archaea	Crenarchaeo	Thermoprote	Sulfolobales	Sulfolobacea	Stygiolobus
ASV_1619	0.33	0	0.33	0.67	0	1.33	1.5	1	0	0	0	0.67	Bacteria	Actinobacter	Actinobacter	Propionibact	Nocardioidac	Nocardioides
ASV_1620	1.33	0	0	0.67	0.67	0	0.5	0	0	0	0.33	0	Bacteria	Abditibacteri	Abditibacteri	Abditibacteri	Abditibacteri	Abditibacterium
ASV_1621	4.33	0	0.33	0	0	0	0	0	0	0	0	0	Bacteria	Proteobacter	Gammaprote	Burkholderia	Burkholderia	Ralstonia
ASV_1622	4.33	0.33	0	0	0	0	0	0	0	0	0	0	Archaea	Crenarchaeo	Thermoprote	Sulfolobales	Sulfolobacea	Stygiolobus
ASV_1623	0.33	0	0	0	0	0	0	0	0	0	0	0	Bacteria	Firmicutes	Clostridia	Clostridiales	Clostridiacea	Clostridium sensu stricto 13
ASV_1624	0	0	0	0	0	0	0	0	0	0	0	0	Bacteria	Firmicutes	YNPFFP2	NA	NA	NA
ASV_1625	0	0	0	0	6.67	0	0	0	0	0	0	0	Bacteria	Proteobacter	Alphaproteol	Rhizobiales	Rhizobiaceae	NA
ASV_1626	0	0	0	0	0	0	0	0	60	0	0	0	Bacteria	Bacteroidota	Bacteroidia	Sphingobacte	Sphingobacte	Pedobacter
ASV_1627	0	0	0	0	0	0	0	0	0	0	0	0	Bacteria	RCP2-54	NA	NA	NA	NA
ASV_1628	2	0	0	0.67	0	0	1	0.33	0	0	0	1	Bacteria	Planctomycet	Planctomycet	Pirellulales	Pirellulaceae	Pirellula
ASV_1629	0.33	0	0.67	0.33	0.33	0	1	0	0	2	0	0	Bacteria	Verrucomicro	Verrucomicro	Chthoniobact	Chthoniobact	Chthoniobacter
ASV_1630	0	0	0	0	0.33	1.33	2	2.33	0	0	0	0.67	Bacteria	Actinobacter	Acidimicrobii	IMCC26256	NA	NA
ASV_1631	0.33	0	0	0.33	0	2	1.5	1	2	0	0	0.33	Bacteria	Actinobacter	Thermoleoph	Solirubrobact	Solirubrobact	Solirubrobacter
ASV_1632	7	0	0	0	0	0	0	0	0	0	0.33	0	Bacteria	Proteobacter	Gammaprote	Burkholderia	Burkholderia	Ralstonia
ASV_1633	0	0.33	0	0	0.33	0.67	5.5	0	0	0	0.33	0	Bacteria	Actinobacter	MB-A2-108	NA	NA	NA
ASV_1634	0	0	0.33	0	0	0	0	0	0	0	0	0	Bacteria	Actinobacter	Actinobacter	Micrococcale	Microbacteri	Microbacterium
ASV_1635	0	0	0	0	0	0	0	0	0	0	0	0.33	Bacteria	Actinobacter	Actinobacter	0319-7L14	NA	NA
ASV_1636	1	0	1.33	0	0	0.67	2	1	0	0	0	0	Bacteria	Proteobacter	Alphaproteol	Rhizobiales	Beijerinckiace	Psychroglaciecola
ASV_1637	0	0	0	0.33	1	0.33	6	0	0	0	0	0.33	Bacteria	Chloroflexi	TK10	NA	NA	NA
ASV_1638	0	2.33	0.33	0.33	0	0	0	2	0	0	0	1	Bacteria	Proteobacter	Alphaproteol	Sphingomona	Sphingomona	Ellin6055
ASV_1639	0.33	0	0	0.33	0.67	0.67	2	0.67	0	0	0	0	Bacteria	Actinobacter	Thermoleoph	Solirubrobact	Solirubrobact	Conexibacter
ASV_1640	0	0	0	0	0	0	0	0	0	0	0	0	Bacteria	Actinobacter	Actinobacter	0319-7L14	NA	NA
ASV_1641	0	0	0	0	0	0	0	0	0	0	8.33	0	Bacteria	Proteobacter	Gammaprote	Enterobacter	Enterobacter	Buttiauxella
ASV_1642	1	0	0	0	0.33	0	0	0	0	0	0.33	0	Bacteria	Actinobacter	Thermoleoph	Gaiellales	NA	NA
ASV_1643	0.67	0	0.33	0	0.67	1	1	1	0	0	0.67	0.67	Bacteria	Actinobacter	Thermoleoph	Solirubrobact	NA	NA
ASV_1644	0	0	1	0	0	1.33	2	0.67	0	0	0	0.67	Bacteria	Verrucomicro	Verrucomicro	Chthoniobact	Chthoniobact	Candidatus Udaeobacter
ASV_1645	5.67	0.33	0.33	0	0	0	0	0	0	0	0	0	Archaea	Crenarchaeo	Thermoprote	Sulfolobales	Sulfolobacea	Stygiolobus
ASV_1646	0	0.67	0	0	0	1.33	3	0	0	0	0.67	0	Bacteria	Firmicutes	Bacilli	Bacillales	Planococcace	NA
ASV_1648	0	0	0	0	0	0	0	0	0	0	8.67	0	Bacteria	Actinobacter	NA	NA	NA	NA
ASV_1649	1.33	0	1.33	0	0	0	0.5	0	6	0	0	0	Bacteria	Actinobacter	Actinobacter	Streptomyce	Streptomyce	E1B-B3-114
ASV_1650	1	0	1.33	0	0	0	0	0	2	0	0.33	0	Bacteria	Actinobacter	Thermoleoph	Gaiellales	NA	NA
ASV_1651	0.67	0	0	1.33	0	1.33	3	1	0	0	0	0.67	Bacteria	Actinobacter	Thermoleoph	Solirubrobact	67-14	NA
ASV_1652	0	0	0.33	0	0.33	1	1	1.33	0	3	0	0.67	Bacteria	Acidobacteri	Blastocatellia	Blastocatellal	Blastocatellac	Stenotrophobacter
ASV_1653	0.33	1	1.33	1	0.67	0	1	0	0	0	0	0	Bacteria	Abditibacteri	Abditibacteri	Abditibacteri	Abditibacteri	Abditibacterium
ASV_1654	0	0	0	0	0	7.33	2	0	0	0	0	0	Bacteria	NA	NA	NA	NA	NA
ASV_1655	0	0	0.67	0	2	2.33	0	0	0	0	0	0	Archaea	Thermoplasn	Thermoplasn	Thermoplasn	Thermoplasn	A-plasma
ASV_1656	5	0.67	0	0	0	0	0	0	0	0	0	0	Bacteria	Chloroflexi	Chloroflexia	Thermomicro	JG30-KF-CM4	NA
ASV_1657	1	0	0	0	0	0	0.5	0	0	0	0	0.67	Bacteria	Bdellovibrion	Bdellovibrion	Bacteriovora	Bacteriovora	Peredibacter
ASV_1658	0.33	0	0.67	0.33	0	0.33	0	1.33	0	0	0.33	0.67	Bacteria	Bdellovibrion	Bdellovibrion	Bacteriovora	Bacteriovora	Peredibacter
ASV_1659	0.33	0	2	0.33	0	0	0	1	0	3	0.67	0	Bacteria	Patescibacter	Saccharimon	Saccharimon	NA	NA
ASV_1660	6.33	0	0	0	0	0	0	0	0	0	0	0	Bacteria	Firmicutes	Bacilli	Staphylococc	Staphylococc	Staphylococcus
ASV_1661	0	0.33	0	0	0	0	0	0	0	0	0	0	Bacteria	Firmicutes	Sulfobacillia	Sulfobacillale	Sulfobacillace	NA
ASV_1662	0	0	0.33	5	0	0	0	0	0	0	0	0	Bacteria	Actinobacter	Thermoleoph	Solirubrobact	Solirubrobact	Conexibacter
ASV_1663	0	0	0	0	0	0	0	0	0	0	0	0	Bacteria	Planctomycet	Planctomycet	Isosphaerales	Isosphaerace	Aquisphaera
ASV_1664	1	0.67	1	0.33	0	0.33	0.5	0.67	0	0	0	0.33	Bacteria	Acidobacteri	Holophagae	Subgroup 7	NA	NA
ASV_1665	1	0	1.67	0	0	0.33	0.5	0.33	0	0	0	0	Bacteria	Myxococcota	Polyangia	Polyangiales	Polyangiacea	Pajaroellobacter
ASV_1666	0.33	0	0	0.33	0	1.67	4	1.33	0	0	0	0	Bacteria	Chloroflexi	KD4-96	NA	NA	NA
ASV_1667	1.33	0.67	0	0.67	0	0	4.5	0	0	0	0	0.67	Bacteria	Actinobacter	MB-A2-108	NA	NA	NA
ASV_1668	1.33	0.33	0	1.33	0	0	0	0	0	0	0	0	Bacteria	Chloroflexi	Chloroflexia	Thermomicro	Thermomicro	Sphaerobacter
ASV_1669	0.33	0	1	0	0	0	0.5	0	0	0	0.33	0	Bacteria	Actinobacter	Actinobacter	NA	NA	NA
ASV_1670	0	0	0	0	0	0	0	0	0	0	0	0	Bacteria	Proteobacter	Gammaprote	Burkholderia	Comamonada	NA
ASV_1673	0	0	0	0	0	0	0	0	0	0	8	0	Bacteria	Proteobacter	Alphaproteol	Rhizobiales	Beijerinckiace	Microvirga
ASV_1674	0.67	0	0	0	0	0.67	1.5	0	0	0	0	0	Bacteria	Actinobacter	Thermoleoph	Gaiellales	NA	NA
ASV_1675	0	0	2	0.33	0	0.33	1	0.33	0	4	0	0.33	Bacteria	Verrucomicro	Verrucomicro	Chthoniobact	Chthoniobact	Chthoniobacter
ASV_1676	0	0	0.67	0	2	1.33	0.5	0	0	0	0	0	Bacteria	Actinobacter	Actinobacter	Corynebacte	NA	NA
ASV_1677	0	0.67	0.33	1	0	1	0	0	0	0	0	0	Bacteria	Proteobacter	Gammaprote	Pseudomona	Marinobacte	Marinobacter
ASV_1678	0	0	0	0	1.33	0.33	2.5	1.67	0	0	0	0	Bacteria	Actinobacter	MB-A2-108	NA	NA	NA
ASV_1680	0	0	0	0.33	0	0	1.5	0.33	0	0	0	0.33	Bacteria	Acidobacteri	Acidobacteria	Bryobacteral	Bryobacterac	Bryobacter
ASV_1681	0	0	0	0	0	0	0	0	0	0	0	0	Bacteria	Actinobacter	Acidimicrobii	IMCC26256	NA	NA
ASV_1682	1.33	0	0.33	0	0	0.33	1.5	0	0	0	0	0	Bacteria	Gemmatimor	Gemmatimor	Gemmatimor	Gemmatimor	NA
ASV_1683	1	1.33	0	0	1	0	0	0	0	0	0	0.67	Bacteria	Actinobacter	Thermoleoph	NA	NA	NA
ASV_1684	0	0	0	0	0	0	0	0	0	0	0	0	Bacteria	Actinobacter	Rubrobacteri	Rubrobactera	Rubrobacteri	Rubrobacter
ASV_1685	5.67	0	0	0	0	0	0	0	0	0	0	0	Bacteria	Proteobacter	Gammaprote	Pseudomona	Pseudomona	Pseudomonas
ASV_1687	0	0	0.33	0	0	0	0	0	0	0	0	0	Bacteria	Proteobacter	Gammaprote	Burkholderia	Rhodocyclac	Azovibrio
ASV_1688	0	0	0	0	0	0	0	0	0	0	7.33	0	Bacteria	Actinobacter	Actinobacter	Corynebacte	Corynebacte	Turicella
ASV_1689	1.33	1.67	0	0.33	0.67	0	0	0	0	0	0	0	Bacteria	Myxococcota	Polyangia	Polyangiales	Polyangiacea	Pajaroellobacter
ASV_1690	1	0	2	0	0	0	0	0	0	0	1	0	Bacteria	Actinobacter	Actinobacter	0319-7L14	NA	NA
ASV_1691	1.33	0	0.67	0.67	0	0.33	0.5	0.33	0	0	0	0.67	Bacteria	Actinobacter	Thermoleoph	Solirubrobact	67-14	NA
ASV_1692	0.67	0	0	0.33	0.33	1	2	0.67	0	0	0	0.33	Bacteria	NA	NA	NA	NA	NA
ASV_1693	0	0	1	0.67	0	0	1.5	0.33	0	0	0	0	Bacteria	Patescibacter	Saccharimon	Saccharimon	NA	NA
ASV_1694	1	0.67	0	1	0	0.67	0	0	0	0	0	0	Bacteria	Proteobacter	Alphaproteol	Rhodobacter	Rhodobacter	NA
ASV_1695	0	0	0	0	0	0	0	0	0	0	0	0	Bacteria	Actinobacter	Thermoleoph	Gaiellales	Gaiellaceae	Gaiella

ASV_1696	0	0	0	0	0	0	0	0	0	0	0	6	Bacteria	Actinobacter	Actinobacter	Streptomyce	Streptomyce	Streptomyces
ASV_1697	0	0	0	0	0	0	0	0	0	0	0	0	Bacteria	Chloroflexi	Chloroflexia	Chloroflexale	Chloroflexace	Chloronema
ASV_1698	0.33	0	0	0.33	0.33	0.67	1	0	0	0	0	0	Bacteria	Actinobacter	Acidimicrobii	Microtrichale	Ilumatobacte	NA
ASV_1699	0.33	0	0	0	0.33	0	0	0	0	0	3.33	0	Bacteria	Actinobacter	Thermoleoph	Gaiellales	NA	NA
ASV_1700	0	0	0.67	0.67	0	0	0	0.33	0	0	0	1	Bacteria	Planctomyce	Planctomyce	Isosphaerales	Isosphaerace	NA
ASV_1701	0	0	0	0	0	6.33	0	0	0	0	0	0	Bacteria	Actinobacter	Acidimicrobii	IMCC26256	NA	NA
ASV_1702	0	0	0	0	0	0	0	8	0	0	0	0	Bacteria	Proteobacter	Gammaprote	Enterobacter	Aeromonada	Aeromonas
ASV_1703	0	0	0	0	0	0	0.5	0	0	0	0.33	10	Bacteria	Actinobacter	Thermoleoph	Solirubrobact	67-14	NA
ASV_1704	0	0	0	0	0	0	0	0	0	0	0	0	Bacteria	Proteobacter	Alphaproteol	Rhodobacter	Rhodobacter	Rubellimicrobium
ASV_1705	0	0	0	0	0	0	0	0	0	0	5.67	0	Bacteria	Proteobacter	Gammaprote	Burkholderia	Comamonada	Hydrogenophaga
ASV_1706	5.67	0	0	0	0	0	0	0	0	0	0	0	Bacteria	Gemmatimor	S0134 terrest	NA	NA	NA
ASV_1707	1.33	0	0.67	0.33	0	0.33	0.5	0.33	0	0	0	1	Bacteria	Actinobacter	Thermoleoph	Solirubrobact	Solirubrobact	Solirubrobacter
ASV_1708	0.33	0.33	0.33	0.33	0	0.33	0.5	0.33	0	0	0	0	Bacteria	Patescibacter	Saccharimon	Saccharimon	NA	NA
ASV_1709	0	0	0	0	0	0	0	0	0	0	0.33	0	Bacteria	Actinobacter	Actinobacter	Euzebyales	Euzebyaceae	NA
ASV_1710	0	0	0	0	0	0	0	8	0	0	0	0	Bacteria	Firmicutes	Sulfobacillia	Sulfobacillale	Sulfobacillace	Sulfobacillus
ASV_1711	0	0	0	0	0	0	0	0	0	0	0	0	Bacteria	Actinobacter	Actinobacter	Pseudonocar	Pseudonocar	NA
ASV_1712	0	0	0	0	0	0	7.5	0	0	0	0	0	Bacteria	Actinobacter	Actinobacter	Streptomyce	Streptomyce	Allostreptomyces
ASV_1713	0	0	0	0	0	0	0	0	0	0	0	0	Bacteria	Gemmatimor	Longimicrobi	Longimicrobi	Longimicrobi	NA
ASV_1714	0	0	0	0	8	0.33	0	0	0	0	0	0	Archaea	Thermoplasn	Thermoplasn	Thermoplasn	Thermoplasn	NA
ASV_1715	1.33	0	0	0	0	0	0	0	0	0	0	0.67	Bacteria	Planctomyce	Planctomyce	Pirellulales	Pirellulaceae	NA
ASV_1716	1	0	0.33	0	0	0	0.5	0.33	0	0	0	0	Bacteria	Proteobacter	Alphaproteol	Rhizobiales	Xanthobacter	NA
ASV_1717	0	0	0.33	0	1	0	1.5	0	0	0	1.67	0	Bacteria	Planctomyce	Planctomyce	Isosphaerales	Isosphaerace	NA
ASV_1718	0.67	0	1.33	0	0	0	0.5	0.33	0	0	0	0	Bacteria	Acidobacteri	Blastocatellia	Blastocatellal	Blastocatellac	Blastocatella
ASV_1719	0	0.67	3.33	0	0	0	0	0	0	3	0	0	Bacteria	Planctomyce	Planctomyce	Gemmatales	Gemmatacea	NA
ASV_1720	0	0	0	0	0	3	3	0	0	0	0	0	Bacteria	Proteobacter	Alphaproteol	Rhizobiales	Devosiaceae	Devosia
ASV_1721	0.33	0	0	0	0.33	0	0.5	0.67	0	0	0	0	Bacteria	Chloroflexi	Dehalococco	S085	NA	NA
ASV_1722	0	0	0	0	0	0	1	0	0	0	6	0	Bacteria	Proteobacter	Gammaprote	Enterobacter	Enterobacter	NA
ASV_1723	0	0	5.33	0	0	0	1	0	0	0	0	0	Bacteria	Proteobacter	Gammaprote	Pseudomona	Pseudomona	Pseudomonas
ASV_1724	0	0	0	2	0	0	0	0	0	0	0	0	Bacteria	Proteobacter	Gammaprote	Pseudomona	Moraxellacea	Acinetobacter
ASV_1725	0	0	0	0	0	0	0	0	0	0	1.67	5.67	Bacteria	Proteobacter	Gammaprote	Pseudomona	Moraxellacea	Enhydrobacter
ASV_1726	0	0	0	0	0	0	0	0	0	0	0	0	Archaea	Thermoplasn	Thermoplasn	Thermoplasn	Thermoplasn	NA
ASV_1727	1	0	1.33	0	0	0	0	0	1	0	0	0	Bacteria	Actinobacter	Acidimicrobii	NA	NA	NA
ASV_1728	0.67	0	0.67	0	0.67	0.33	1.5	0	2	0	0.33	0	Bacteria	Proteobacter	Gammaprote	Burkholderia	Oxalobactera	Massilia
ASV_1729	0.33	0	0.67	0.33	0	0	1.5	0.67	0	0	0	0.67	Bacteria	Patescibacter	NA	NA	NA	NA
ASV_1730	0.33	0	0.33	0	5.67	0	0	0	0	0	0	0	Bacteria	Actinobacter	Rubrobacteri	Rubrobactera	Rubrobacteri	Rubrobacter
ASV_1731	0.67	0	0	0	0	1	2.5	0.33	0	2	0	0	Bacteria	Actinobacter	Acidimicrobii	NA	NA	NA
ASV_1732	0	0.33	0	0.33	0.33	1	3	0.33	0	0	1	0	Bacteria	Bacteroidota	Bacteroidia	Sphingobacte	NA	NA
ASV_1733	0	0	0	0	0.33	0.33	3	1.33	0	0	0	0.33	Bacteria	Actinobacter	Actinobacter	Frankiales	NA	NA
ASV_1734	0	0	0	0	0	0	0	0	0	0	0	0	Bacteria	Firmicutes	Sulfobacillia	Sulfobacillale	Sulfobacillace	Sulfobacillus
ASV_1735	0	0	0	0	0	0	0	0.33	0	0	0	0	Bacteria	Actinobacter	Actinobacter	Frankiales	Geodermato	Modestobacter
ASV_1736	0	0	0.33	0	0	0	0	0	0	0	0	0	Bacteria	Actinobacter	Actinobacter	Micrococcale	NA	NA
ASV_1737	0	0	0	0	0	0	0	0	0	0	0.67	0	Bacteria	Actinobacter	Thermoleoph	NA	NA	NA
ASV_1738	0	0	0	0	0	0	0	0	0	0	0	0	Bacteria	Actinobacter	Thermoleoph	Gaiellales	NA	NA
ASV_1739	0	0	0	0	0	0	0	0	0	0	5.67	0	Bacteria	Proteobacter	Alphaproteol	Tistrellales	Geminicocca	Geminicoccus
ASV_1740	0	0	0	0	0	0	0	0	0	0	0	0	Bacteria	Firmicutes	Bacilli	Bacillales	Bacillaceae	Geobacillus
ASV_1741	1.33	0	0.33	0.67	0	0.33	1	0	0	0	0	0.33	Bacteria	Patescibacter	Saccharimon	Saccharimon	NA	NA
ASV_1742	0.67	1	1.67	0.67	0	0.67	2.5	0	0	0	0	0	Bacteria	Actinobacter	NA	NA	NA	NA
ASV_1743	0.33	0	1.33	0	0	0	0	0.33	0	2	0	0	Bacteria	Patescibacter	Saccharimon	Saccharimon	NA	NA
ASV_1744	1	0	0	0	0	0.67	0	0.33	2	0	1.33	0.67	Bacteria	Firmicutes	Bacilli	Bacillales	Bacillaceae	Geobacillus
ASV_1745	0.33	0	0	0	0	0	0	0	0	0	4.33	0	Bacteria	Firmicutes	Bacilli	Staphylococc	Staphylococc	Staphylococcus
ASV_1746	0	0	0.67	0	0.67	0	1	0	0	0	0.67	0	Bacteria	Proteobacter	Gammaprote	Burkholderia	TRA3-20	NA
ASV_1747	0	0.67	0	0	0	0	0	0	25	0	0	0	Archaea	Crenarchaeo	Nitrososphae	Group 1.1c	NA	NA
ASV_1748	0	0	0	0	0	3	5.5	0	0	0	0	0	Bacteria	Actinobacter	Rubrobacteri	Rubrobactera	Rubrobacteri	Rubrobacter
ASV_1749	0	0	0	0	0	0	0	9.67	0	0	0	0	Bacteria	Proteobacter	Gammaprote	Burkholderia	Alcaligenacea	Alcaligenes
ASV_1750	0	0	0	0	0	0	0	0	0	0	0	0	Bacteria	Actinobacter	Thermoleoph	Solirubrobact	67-14	NA
ASV_1751	0	0	0	1.67	0	0	0.5	0	0	2	0	0	Bacteria	Actinobacter	Thermoleoph	NA	NA	NA
ASV_1752	0.67	0	0.33	0.67	0	0	2.5	0	0	0	0	0.67	Bacteria	Bacteroidota	Bacteroidia	Sphingobacte	Sphingobacte	Pedobacter
ASV_1753	0.33	0	1	0	0	1.33	1.5	0	0	2	0	0	Bacteria	Verrucomicro	Verrucomicro	Chthoniobact	Chthoniobact	Chthoniobacter
ASV_1754	0.33	0	0	0	0	0	1	1	0	0	0.33	0	Bacteria	Actinobacter	Actinobacter	Frankiales	NA	NA
ASV_1755	0.33	0.33	0.33	1	0	0	1.5	0	0	0	0	0	Bacteria	Actinobacter	Thermoleoph	Solirubrobact	Solirubrobact	Solirubrobacter
ASV_1756	0.33	0	0	0	0.33	1.33	1.5	1.33	0	0	0	0.33	Bacteria	Verrucomicro	Verrucomicro	Verrucomicro	Verrucomicro	Verrucomicrobium
ASV_1757	0.33	0	0	0	1	0.67	0.5	1	0	0	0	0	Bacteria	Acidobacteri	Blastocatellia	Blastocatellal	Blastocatellac	Blastocatella
ASV_1758	1.33	1.33	0	1.33	0	0	0	0	0	0	0	0	Bacteria	Actinobacter	Rubrobacteri	Rubrobactera	Rubrobacteri	Rubrobacter
ASV_1759	0	0	0	0	0	0	0.5	0	0	0	0	0	Bacteria	Actinobacter	Actinobacter	Frankiales	Sporichthyac	NA
ASV_1760	0	1	0.33	0	0	0	0	0	0	0	0	0	Archaea	Crenarchaeo	Thermoprote	Sulfolobales	Sulfolobacea	Stygiolobus
ASV_1761	0	0	0	0	9	0	0	0.33	0	0	0	0	Bacteria	Firmicutes	Bacilli	Bacillales	Bacillaceae	Bacillus
ASV_1762	0	0	0	0	0	0	0	0	0	0	0	0	Bacteria	Bacteroidota	Bacteroidia	Flavobacteria	Weeksellacea	Cloacibacterium
ASV_1763	0	0	0	0	0	0	0	1	0	0	3.67	0	Bacteria	Gemmatimor	Longimicrobi	Longimicrobi	Longimicrobi	NA
ASV_1764	0	0	0	0	0	0	0	0	0	0	0	7.33	Bacteria	Proteobacter	Gammaprote	Burkholderia	Burkholderia	Ralstonia
ASV_1765	0	0	0	0	0	0	0	0	0	0	0	0	Bacteria	Proteobacter	Alphaproteol	Caulobactera	Caulobactera	PMMR1
ASV_1766	0.33	0.67	0	0	0	0	0	0.67	0	0	0	0.67	Bacteria	Chloroflexi	Gitt-GS-136	NA	NA	NA
ASV_1767	0.33	0	0.33	0.33	0.33	0.67	1.5	0.67	0	0	0.33	0	Bacteria	Actinobacter	Actinobacter	Frankiales	Sporichthyac	NA
ASV_1768	0	0	1	0	0.33	0.33	2	0	0	0	0.67	0	Bacteria	Planctomyce	Phycisphaera	Tepidisphaer	WD2101 soil	NA
ASV_1769	3.33	0.67	0	0	0	0	0.5	0	0	0	0	0.67	Bacteria	Bacteroidota	Bacteroidia	Cytophagales	Spirosomace	Pseudarcicella
ASV_1770	4.67	0	0	0	0	0	0	0.33	0	0	0.67	0	Bacteria	Proteobacter	Gammaprote	Burkholderia	Burkholderia	Ralstonia
ASV_1771	0.33	0	0	0	0	0	2	0	0	0	7.33	0	Bacteria	Bacteroidota	Bacteroidia	Chitinophaga	Chitinophaga	Taibaiella
ASV_1772	0	0	0	0	0	0	0	0	0	0	0	0	Archaea	Thermoplasn	Thermoplasn	Thermoplasn	Thermoplasn	A-plasma
ASV_1773	0	0.33	0	0	0	0	0	0	0	0	0	0	Bacteria	Proteobacter	Alphaproteol	Rhizobiales	Beijerinckiac	Methylovirgula
ASV_1774	0	3.33	1	0	0	0	0	0.67	0	0	0.33	0.67	Bacteria	Actinobacter	Rubrobacteri	Rubrobactera	Rubrobacteri	Rubrobacter
ASV_1775	0	0	0	0	0	0	0	6	0	0	0	0	Bacteria	Chloroflexi	Anaerolineae	Ardenticaten	Ardenticaten	NA
ASV_1776	0	0	0.33	0	0	0	0	0	0	57	0	0	Bacteria	Firmicutes	Bacilli	Thermicanale	Thermicanac	Thermicanus
ASV_1777	0	0	0	0	0.33	0.67	0	0	0	0	0	0	Bacteria	Firmicutes	Sulfobacillia	Sulfobacillale	Sulfobacillace	Sulfobacillus
ASV_1778	1	0.33	0	0.33	0	0.33	0.5	0.33	0	0	0.67	0.33	Bacteria	Planctomyce	Phycisphaera	Tepidisphaer	WD2101 soil	NA
ASV_1779	1.33	0	1	0	0.33	0	0.5	0	0	0	0	0	Bacteria	Chloroflexi	Chloroflexia	Chloroflexale	Herpetosipho	Herpetosiphon
ASV_1780	1.33	0	0.33	0	0	0	0.5	0	0	0	0.67	0	Bacteria	Patescibacter	Saccharimon	Saccharimon	LWQ8	NA
ASV_1781	1.67	0	0	0	0.33	0	0.5	0	0	0	0	0	Bacteria	Nitrospirota	Nitrospiria	Nitrospirales	Nitrospiracea	Nitrospira
ASV_1782	0	0	0.33	0	0	0.67	0.5	0.67	0	0	0	0	Bacteria	Chloroflexi	Chloroflexia	Thermomicro	JG30-KF-CM4	NA
ASV_1783	0.33	0	0.33	0	0.33	0.67	0.5	0	0	0	0	0.67	Bacteria	Actinobacter	Actinobacter	Frankiales	NA	NA
ASV_1784	0.33	0	0.33	0	0.33	1.33	1.5	1	0	0	0	0.33	Bacteria	Planctomyce	Phycisphaera	Phycisphaera	Phycisphaera	NA
ASV_1785	1	0	0	0.33	0	0	0	0	0	0	0	0	Archaea	Crenarchaeo	Thermoprote	Sulfolobales	Sulfolobacea	Stygiolobus
ASV_1786	0	0	0	0	0	0	0	0.33	0	0	0	4	Bacteria	Actinobacter	Actinobacter	Micrococcale	Dermacoccac	NA
ASV_1787	0	0.33	0	1.33	0	0	0.5	0	0	0	0	0.33	Bacteria	Chloroflexi	KD4-96	NA	NA	NA
ASV_1788	0	0.33	0.33	0	0	0	0	1	0	0	1	2.33	Bacteria	Actinobacter	Actinobacter	Corynebacter	Nocardiaceae	Nocardia
ASV_1789	0	0	0.33	0	0	0	1	0	0	0	0	0	Bacteria	Myxococcota	Polyangia	Haliangiales	Haliangiaceae	Haliangium
ASV_1790	0	0.67	0	0	0	0	0	1.33	0	0	0.33	0.33	Bacteria	Proteobacter	Gammaprote	Pseudomona	Moraxellacea	Acinetobacter
ASV_1791	0	0	0	0	0	0	0	0	0	0	0	0	Bacteria	Bacteroidota	Bacteroidia	Bacteroidales	Dysgonomon	Dysgonomonas
ASV_1792	0	0	0.33	0	1.33	0	1.5	0	1	0	1	0	Bacteria	Proteobacter	Gammaprote	Xanthomona	Xanthomona	Xanthomonas
ASV_1793	0	0	0	0	2.67	4.33	0	0	0	0	0	1	Archaea	Thermoplasn	Thermoplasn	NA	NA	NA
ASV_1794	0	0	0	0	0	0	0	0.33	0	0	5.67	0	Bacteria	Actinobacter	Actinobacter	Pseudonocar	Pseudonocar	NA
ASV_1795	0	0	0	0	0.33	0	2	0	0	0	1	0	Bacteria	Actinobacter	Actinobacter	0319-7L14	NA	NA
ASV_1796	0	0.33	1	0	0	0	0	0	0	0	0	0.33	Bacteria	Actinobacter	NA	NA	NA	NA
ASV_1797	0.67	0.33	0.67	0	0.33	0	0.5	0	0	0	0	1.33	Bacteria	Proteobacter	Gammaprote	Xanthomona	Xanthomona	NA
ASV_1798	0.33	0	0	0.33	0.33	0.67	0.5	1.67	0	0	0	0	Bacteria	Actinobacter	Thermoleoph	Solirubrobact	Solirubrobact	Solirubrobacter
ASV_1799	6	0	0	0	0	0	0	0	0	0	0.33	0	Bacteria	Proteobacter	Gammaprote	Burkholderia	Burkholderia	Ralstonia
ASV_1800	0	0	0.33	0	0	0	0	0	0	0	0	0	Bacteria	Firmicutes	Bacilli	Exiguobacter	Exiguobacter	Exiguobacterium
ASV_1801	1.33	0	1.33	0	0	0	0	0	0	0	0.33	0	Bacteria	Actinobacter	Thermoleoph	Solirubrobact	67-14	NA
ASV_1802	0.33	0	2.33	0	0	0	0	0.33	0	0	0	0	Bacteria	Planctomyce	Planctomyce	Gemmatales	Gemmatacea	Gemmata
ASV_1803	0.67	0.33	0	1.67	0	0	0	0	0	0	0.67	0	Bacteria	Actinobacter	Actinobacter	0319-7L14	NA	NA
ASV_1804	0	0	0.33	0	1.67	0.33	0	0.33	0	0	3	0	Bacteria	Proteobacter	Alphaproteol	Sphingomona	Sphingomona	Sphingomonas
ASV_1805	0	0	0	0.67	0	0	0	0	0	0	0	0	Bacteria	Actinobacter	Actinobacter	NA	NA	NA
ASV_1806	0	0	0	0	0	0	0	0	0	0	0	0	Bacteria	Actinobacter	Actinobacter	Micrococcale	Microbacteri	NA
ASV_1807	0	0	0	0	0.33	0	0	0	38	0	0	0	Bacteria	Actinobacter	Rubrobacteri	Rubrobactera	Rubrobacteri	Rubrobacter
ASV_1808	0	0	0	0	0	0.33	0	0	0	0	0	0	Bacteria	Actinobacter	Acidimicrobii	IMCC26256	NA	NA
ASV_1809	0	0	0	0	0	0	0	0	50	0	0	0	Bacteria	Actinobacter	Actinobacter	Corynebacter	Corynebacter	Corynebacterium
ASV_1810	0	0	0	0	0	0	0	0	0	0	0	0	Bacteria	Actinobacter	Actinobacter	Euzebyales	Euzebyaceae	NA
ASV_1811	0.33	0	0	0.33	0.67	0.33	0	0.67	0	0	0	0.67	Bacteria	Actinobacter	Acidimicrobii	Microtrichale	Iamiaceae	Iamia
ASV_1812	0	0	0	0	1.67	0	1	0	2	0	1.67	0	Bacteria	Planctomyce	Planctomyce	Isosphaerales	Isosphaerace	NA
ASV_1814	0	0	1	0	1	0	0.5	0	0	0	1.33	0	Bacteria	Chloroflexi	Dehalococco	S085	NA	NA
ASV_1815	0	0	0	0	0	0	8	0	0	0	0	0	Bacteria	Chloroflexi	Dehalococco	S085	NA	NA
ASV_1816	0	0	0	0	0	0	0	0	0	0	0	0.33	Bacteria	Actinobacter	Actinobacter	0319-7L14	NA	NA
ASV_1817	0.33	0	0.67	0	0	0	0	0	0	0	0	0	Bacteria	Actinobacter	Thermoleoph	Solirubrobact	67-14	NA
ASV_1818	0.67	0.33	1.33	0	0	0	0.5	1	0	0	0	0	Bacteria	Actinobacter	Thermoleoph	Solirubrobact	67-14	NA
ASV_1819	0.33	0	0.67	0.33	0	0.67	0.5	0.33	0	0	0	0	Bacteria	Patescibacter	Saccharimon	Saccharimon	NA	NA
ASV_1820	0.67	0	2.67	0	0	0	0	0	0	0	0	0	Bacteria	Proteobacter	Gammaprote	Pseudomona	Pseudomona	Pseudomonas
ASV_1821	1	0	0	0.33	0.33	0	1	0.67	0	0	0.33	0.33	Bacteria	Patescibacter	Saccharimon	Saccharimon	LWQ8	NA
ASV_1822	0	0	0.33	0	3.33	0.33	2	0.33	0	0	1.33	0	Bacteria	Acidobacteri	Blastocatellia	Blastocatellal	Blastocatellac	Blastocatella
ASV_1823	0	0	0	0	0	0	0	0	0	2	0	0	Bacteria	Firmicutes	Bacilli	Thermicanale	Thermicanac	Thermicanus
ASV_1824	0	0	0	0	0	0	0	0	43	0	0	0	Bacteria	Actinobacter	Thermoleoph	Solirubrobact	67-14	NA
ASV_1825	0	0	0	0	0.33	0	0	0.67	0	0	0	0	Bacteria	Chloroflexi	Chloroflexia	Thermomicro	JG30-KF-CM4	NA
ASV_1826	0	0	0.33	0	0.33	1	0.5	0.33	0	0	0.33	0.33	Bacteria	Actinobacter	Thermoleoph	Gaiellales	NA	NA
ASV_1827	0.67	0	0	1	0	0.33	0.5	0	0	0	0	0	Bacteria	Proteobacter	Gammaprote	Xanthomona	Xanthomona	Lysobacter
ASV_1828	0	0	0	0	0.67	0.33	3.5	0	0	0	0	0.67	Bacteria	Proteobacter	Gammaprote	Beggiatoales	Beggiatoacea	NA
ASV_1829	0.33	0	0	1	0	0.33	0.5	0.33	0	0	0	0.67	Archaea	Crenarchaeo	Nitrososphae	Nitrososphae	Nitrososphae	Candidatus Nitrocosmicus
ASV_1830	2.33	0.33	0	0	0	0	0	0	0	0	0	0	Bacteria	Deinococcota	Deinococci	Thermales	Thermaceae	Meiothermus
ASV_1831	0	0	0.67	0	1.33	0	2	0	1	0	0.67	0	Bacteria	Actinobacter	Thermoleoph	NA	NA	NA
ASV_1832	0	0	0	0	0	0	0	0	0	0	0	0	Bacteria	Actinobacter	Thermoleoph	Gaiellales	NA	NA
ASV_1833	0	0	0	0	0	0	0	0.33	34	0	0	0	Bacteria	Cyanobacteri	Cyanobacteri	NA	NA	NA
ASV_1834	0.33	0	0	0.33	0	0	1	0.33	0	2	0	0.33	Bacteria	Bdellovibrion	Bdellovibrion	Bacteriovora	Bacteriovora	Peredibacter
ASV_1835	0.33	0	1	0.33	0	0	0	0	0	2	0	0	Bacteria	Planctomyce	Planctomyce	Isosphaerales	Isosphaerace	NA
ASV_1836	1.67	0	0	0	0	0	3	0	0	0	0	0	Bacteria	Actinobacter	Actinobacter	Kineosporiale	Kineosporiac	Quadrisphaera
ASV_1837	0	0	0.33	0	0	0	0	0.33	0	0	0.33	2.67	Bacteria	Actinobacter	Thermoleoph	Solirubrobact	67-14	NA
ASV_1838	0	0	0	0	0.33	0.67	3	0	0	0	0	0	Bacteria	Actinobacter	Thermoleoph	Solirubrobact	67-14	NA
ASV_1839	0.33	0.33	0	1.33	0.67	0	0	0	0	0	0	0	Bacteria	Proteobacter	Gammaprote	Diplorickettsi	Diplorickettsi	NA
ASV_1840	0	0	0	0	0	0	0	0	32	0	0	0	Archaea	Crenarchaeo	Nitrososphae	Group 1.1c	NA	NA
ASV_1841	0	0	0	0	0	0	0	0	0	0	0	0	Bacteria	Firmicutes	Sulfobacillia	Sulfobacillale	Sulfobacillace	Sulfobacillus
ASV_1842	0	0.67	0	0	0	1.33	3	0	0	0	0.33	0	Bacteria	Acidobacteri	Blastocatellia	Pyrinomonac	Pyrinomonac	RB41
ASV_1843	0	0	0	0	0	7.67	0.5	0	0	0	0	0	Bacteria	Proteobacter	Gammaprote	Pseudomona	Pseudomona	Pseudomonas

ASV_1844	0	0	0	0	0	0	0	0.33	0	42	0	0	Bacteria	Firmicutes	Bacilli	Thermicanale	Thermicanac	Thermicanus
ASV_1845	0	0	0	0	0.67	2	1	2	0	0	0	0.33	Bacteria	Actinobacter	Acidimicrobii	Acidimicrobia	Acidimicrobia	Aciditerrimonas
ASV_1846	0.67	0	1	0	0	0	0.5	0	0	0	0	0	Bacteria	Patescibacter	Saccharimon	Saccharimon	NA	NA
ASV_1847	0.67	0.33	0.67	0.33	0	0	0	0.33	0	0	0	0	Bacteria	Actinobacter	Thermoleopl	Gaiellales	NA	NA
ASV_1848	1.67	0	0.67	0	0	0	1	0	0	0	0	0	Bacteria	Actinobacter	Actinobacter	Propionibact	Nocardioidac	Marmoricola
ASV_1849	0.33	0.67	0	0.67	0	0.33	0.5	0	0	2	0.33	0	Bacteria	Actinobacter	Actinobacter	Micromonos	Micromonos	Longispora
ASV_1850	1	0.67	0	1	0	0	0	0	0	0	0	0	Bacteria	Chloroflexi	Chloroflexia	Thermomicr	JG30-KF-CM4	NA
ASV_1851	0	0	0	0	0	0	0	0	0	0	0	0	Bacteria	Proteobacter	Gammaprote	Pseudomona	Moraxellacea	Acinetobacter
ASV_1852	0	0	0.67	1.67	0	0	0	0	0	0	0	0	Archaea	Crenarchaeo	Thermoprote	Thermoprote	Thermoprote	Vulcanisaeta
ASV_1853	0	0	0	0	0	0	0	0	0	0	0	0	Bacteria	Firmicutes	Sulfobacillia	Sulfobacillale	Sulfobacillac	NA
ASV_1854	0	0	3	0	0	0	1.5	0	0	0	0	0.67	Bacteria	Actinobacter	Actinobacter	Bifidobacteria	Bifidobacteria	NA
ASV_1855	0	0	0	0	0	0	0	0	0	0	0	0	Bacteria	Planctomyce	Planctomyce	Gemmatales	Gemmatacea	NA
ASV_1856	0.67	0	1	0	0.33	0	0	0.33	0	0	1	0	Bacteria	Gemmatimor	Longimicrobi	Longimicrobi	Longimicrobi	NA
ASV_1857	0	0	0.67	0	0	0	0	1	0	0	2	0	Bacteria	Deinococcota	Deinococci	Deinococcale	Deinococcac	Deinococcus
ASV_1858	1.33	0.33	0	1.33	0	0	0	0.33	0	0	0	0	Bacteria	Proteobacter	Alphaproteol	Sphingomona	Sphingomona	Qipengyuania
ASV_1859	0.67	0	0	0	0	0	2.5	0	0	0	0	0.67	Bacteria	Proteobacter	Alphaproteol	Sphingomona	Sphingomona	NA
ASV_1860	0	0	0	0	0	0	0	0	0	0	0	0	Archaea	Halobacterot	Methanosarc	Methanosarc	Methanosarc	Methanosarcina
ASV_1861	0	0	0	0	0	0	0	0	0	0	4.33	0	Bacteria	Firmicutes	Bacilli	Bacillales	Bacillaceae	Bacillus
ASV_1862	0	0	0	0	2.67	0	3.5	1.67	0	0	0	0.33	Bacteria	Actinobacter	Actinobacter	Corynebacte	NA	NA
ASV_1863	0	0	0	0	0	0	0	0	0	0	0	0	Bacteria	Actinobacter	Thermoleopl	Gaiellales	NA	NA
ASV_1864	0	0	0	0	0	0	0	0	0	0	0	0	Bacteria	Actinobacter	Actinobacter	Micrococcale	Microbacteria	Curtobacterium
ASV_1865	0.33	0	0.33	0	0	0	1	0	0	0	0	0	Bacteria	Actinobacter	Thermoleopl	Solirubrobact	Solirubrobact	Solirubrobacter
ASV_1866	0.67	0	1	0	0	0	0.5	0	0	0	0	0	Bacteria	Planctomyce	Planctomyce	Gemmatales	Gemmatacea	NA
ASV_1867	0	0	0	0	0	1.33	0.5	2.33	0	0	0	0	Bacteria	Firmicutes	Bacilli	Paenibacillale	Paenibacillac	NA
ASV_1868	0.33	0	1.67	0	0.33	0	0	0.33	0	3	0	0.67	Bacteria	Chloroflexi	Chloroflexia	Thermomicr	JG30-KF-CM4	NA
ASV_1869	0	0	0	0.33	0	0	4.5	0	0	0	0	1	Bacteria	Firmicutes	Bacilli	Bacillales	Bacillaceae	Aeribacillus
ASV_1870	0	0	0	0	0	0	0	0	0	0	0	6	Bacteria	Proteobacter	Gammaprote	Burkholderia	Comamonad	NA
ASV_1871	1.33	0	0.67	0	0	0.33	2	0	0	0	0	0.33	Bacteria	Chloroflexi	Chloroflexia	Thermomicr	AKYG1722	NA
ASV_1872	0.33	0	0	0.33	0	0.33	1	0	0	0	0	0	Bacteria	Actinobacter	Acidimicrobii	Acidimicrobia	Acidimicrobia	Aciditerrimonas
ASV_1873	0	0	0.33	0.33	0	1.67	2	0	0	0	0	0.33	Bacteria	Chloroflexi	Dehalococco	S085	NA	NA
ASV_1874	0	0	0	0	0	0	0	0	0	0	0.33	0	Bacteria	Actinobacter	Rubrobacteri	Rubrobactera	Rubrobacteri	Rubrobacter
ASV_1875	0	0	0	1.33	0	0.67	0.5	0	0	0	0	0	Bacteria	Proteobacter	Alphaproteol	Sphingomona	Sphingomona	Sphingomonas
ASV_1876	2.33	0	0	0	0	0	0	0	0	0	0.67	0	Bacteria	Firmicutes	Bacilli	Staphylococc	Staphylococc	Staphylococcus
ASV_1877	0	0	0	0	0	0	9.5	0	0	0	0	0	Bacteria	Proteobacter	Gammaprote	Xanthomona	Xanthomona	Pseudoxanthomonas
ASV_1878	0	0	0	0	0	1	3.5	0	0	0	0.67	0	Bacteria	Actinobacter	Thermoleopl	Gaiellales	NA	NA
ASV_1879	0	0	0.33	0.33	0	0.33	0.5	0.67	0	0	0	0.33	Bacteria	Proteobacter	Alphaproteol	Sphingomona	Sphingomona	Sphingomonas
ASV_1880	0	0.33	0.67	0	0	0	0	0	0	0	0	0	Bacteria	Chloroflexi	Chloroflexia	Kallotenuales	AKIW781	NA
ASV_1881	0.33	0	0	0.33	0	0.67	1	0	0	0	0	0	Bacteria	Actinobacter	Acidimicrobii	IMCC26256	NA	NA
ASV_1882	0	0	0.33	0	0.33	0	0.5	0.33	0	0	0	0	Bacteria	Actinobacter	Actinobacter	Propionibact	Nocardioidac	NA
ASV_1883	1	0.33	0	1	0	0	0	0	0	0	0	0	Bacteria	Proteobacter	Alphaproteol	Rhizobiales	Rhizobiaceae	NA
ASV_1884	0.67	0	3	0	0	0	1	0	0	0	0	1	Bacteria	NA	NA	NA	NA	NA
ASV_1885	0	3.33	0	0	0	0.33	0	0	0	0	0	0	Bacteria	Actinobacter	Actinobacter	Micrococcale	Microbacteria	Microbacterium
ASV_1886	0	0	0	0	1.33	0.33	2	0	0	0	0	0	Bacteria	Verrucomicro	Verrucomicro	Verrucomicro	Verrucomicro	Roseimicrobium
ASV_1887	0	0	3.67	0	0	0	0	0	0	0	0	0	Bacteria	Bacteroidota	Bacteroidia	Flavobacteria	Weeksellacea	Chryseobacterium
ASV_1888	0	0	0	0	0	0	0	0	38	0	0	0	Bacteria	Proteobacter	Gammaprote	Pseudomona	Pseudomona	Pseudomonas
ASV_1889	0	0	0	0	0	0	0	0	0	0	0	0	Bacteria	Actinobacter	Thermoleopl	Solirubrobact	NA	NA
ASV_1890	0	0	0	0	0	0	0	0	0	0	0	0	Bacteria	Proteobacter	Gammaprote	Pseudomona	Halomonada	Halomonas
ASV_1891	1.33	0.33	0	0	0.33	1	2	0	0	0	0	0	Bacteria	Actinobacter	Actinobacter	Micrococcale	Micrococcac	Nesterenkonia
ASV_1892	0.67	0	0	0	0	0.33	0	0.67	0	0	0	0	Bacteria	Actinobacter	Thermoleopl	Solirubrobact	67-14	NA
ASV_1893	0	5	0	0	0	0	0	0	0	0	0	0	Bacteria	Actinobacter	Rubrobacteri	Rubrobactera	Rubrobacteri	Rubrobacter
ASV_1894	0	0	0	0	0	0	0	0	0	0	0	0	Bacteria	Acidobacteri	Acidobacteria	Bryobacterale	Bryobacterac	Bryobacter
ASV_1895	0.33	0.67	0	0	0	0	0	0.33	0	0	0	0.33	Bacteria	Planctomyce	Phycisphaera	Phycisphaera	Phycisphaera	NA
ASV_1896	0.33	0.33	0	0	0	0.33	1	0	0	0	0	0.67	Bacteria	Acidobacteri	Holophagae	Subgroup 7	NA	NA
ASV_1897	0.33	2	0	0	0	0	0	0	0	0	0	0	Bacteria	Proteobacter	Gammaprote	Enterobacter	Idiomarinace	NA
ASV_1898	0.33	0	0	0.67	0.33	0	1.5	0	0	0	0	0	Bacteria	Chloroflexi	Anaerolineae	Caldilineales	Caldilineacea	NA
ASV_1899	0	0	0.33	0	0	0	0	1	0	2	0	0	Bacteria	Gemmatimor	Gemmatimor	Gemmatimor	Gemmatimor	Gemmatimonas
ASV_1900	0	0	0	0.33	0	0.33	0	0.67	0	0	0	0.33	Bacteria	Actinobacter	Acidimicrobii	NA	NA	NA
ASV_1901	0	0	0	0.67	0	0.33	1.5	0.67	0	0	0	1	Bacteria	Chloroflexi	Chloroflexia	Chloroflexale	Herpetosiphc	Herpetosiphon
ASV_1902	0.33	0.67	0	0.33	0	0.33	0	0	0	0	0	0	Bacteria	Proteobacter	Gammaprote	Burkholderia	Comamonad	Piscinibacter
ASV_1903	0	0.33	0	1	0	0	1.5	0.33	0	0	0	0.33	Bacteria	Proteobacter	Alphaproteol	Rhizobiales	NA	NA
ASV_1904	0	0	0	0	0	1	3	0	0	0	0	0	Bacteria	Proteobacter	Alphaproteol	Rhizobiales	Beijerinckiac	NA
ASV_1905	0	0	0	0	0	0	1	0	0	0	1	0.33	Bacteria	Actinobacter	NA	NA	NA	NA
ASV_1906	0	0	0	0	0	0	0	6	0	0	0	0	Bacteria	Actinobacter	Acidimicrobii	Microtrichale	NA	NA
ASV_1907	0	0	0	0	0	0	0	0	0	0	1.33	0	Bacteria	Actinobacter	Actinobacter	Nitriliruptora	Nitriliruptora	NA
ASV_1908	0	0	0	0	0	0	0	0	0	0	4	0	Bacteria	Actinobacter	Actinobacter	Nitriliruptora	Nitriliruptora	NA
ASV_1909	2	0	0.67	0	0	0	0	0	0	0	0.67	0	Bacteria	Actinobacter	Actinobacter	Micrococcale	Promicromor	Krasilnikoviella
ASV_1910	0.33	0	0	0.33	0.33	1.67	1.5	0.67	0	0	0	0	Bacteria	Patescibacter	Saccharimon	Saccharimon	LWQ8	NA
ASV_1911	0	0	0	0	0	0	0	0	0	0	0	0	Bacteria	Firmicutes	Sulfobacillia	Sulfobacillale	Sulfobacillac	NA
ASV_1912	0	0	0	0	0	0	0	0	0	0	0	4	Bacteria	Firmicutes	Bacilli	Bacillales	Bacillaceae	NA
ASV_1913	0	0	0	0	0	2.33	0.5	0	0	0	0	0	Bacteria	Actinobacter	Acidimicrobii	Acidimicrobia	Acidimicrobia	Aciditerrimonas
ASV_1914	0	0	0	0	0	0	0	0	0	0	6	0	Bacteria	Proteobacter	Alphaproteol	Rhizobiales	Rhizobiales Ir	Bauldia
ASV_1915	0	0	1.33	0	0.67	0	0	0	0	0	0	0	Bacteria	Acidobacteri	Holophagae	Subgroup 7	NA	NA
ASV_1916	0.33	0	0	0	0	0	0	1	0	0	0.33	0	Bacteria	Bdellovibrion	Bdellovibrion	Bacteriovora	Bacteriovora	Peredibacter
ASV_1917	0	0.33	0.67	0.33	1	0.33	0.5	0	0	0	2.33	0	Bacteria	Actinobacter	Thermoleopl	Gaiellales	Gaiellaceae	Gaiella
ASV_1918	0	0	0	0.33	0.33	0.33	0	0	0	0	0	0	Bacteria	Armatimonac	Armatimonac	Armatimonac	NA	NA
ASV_1919	0	0.33	0	1	0	0	0	0	0	0	0	0	Bacteria	Proteobacter	Alphaproteol	Sphingomona	Sphingomona	Sphingomonas
ASV_1920	0.33	0	0	0	0	0	0	0	0	0	0	0	Archaea	Crenarchaeo	Nitrososphae	Group 1.1c	NA	NA
ASV_1921	0	0	0	0	0	0	0	0	0	0	0	0	Archaea	Thermoplasn	Thermoplasn	Thermoplasn	Thermoplasm	A-plasma
ASV_1922	0	0	0	1.33	0	0	0	0	0	0	0	0	Bacteria	Proteobacter	Gammaprote	Pseudomona	Moraxellacea	Acinetobacter
ASV_1923	0	0	0	0	0	0	0	0	0	0	5.67	0	Bacteria	Bacteroidota	Bacteroidia	Sphingobacte	Sphingobacte	Mucilaginibacter
ASV_1924	0.33	0	0.67	0	0	0	0	0	0	0	0.33	0	Bacteria	Actinobacter	Thermoleopl	Gaiellales	NA	NA
ASV_1925	0	0	0	0	0	0	0	0	29	0	0	0	Bacteria	Actinobacter	Actinobacter	Streptomyce	Streptomyce	Streptomyces
ASV_1926	0.33	0	0	0.33	0	1	1	0	0	0	0	0	Bacteria	Actinobacter	Acidimicrobii	Microtrichale	Ilumatobacte	NA
ASV_1927	0	0	0.67	0	0	0.67	2.5	0	0	0	0	0	Bacteria	Planctomyce	Phycisphaera	Tepidisphaer	WD2101 soil	NA
ASV_1928	0	0	0	0	0	0.67	4	0	0	0	0	0	Bacteria	Proteobacter	Gammaprote	Enterobacter	Alteromonad	Alishewanella
ASV_1929	0	1	0	0	0	0	0	0.67	0	0	0	0.33	Bacteria	NA	NA	NA	NA	NA
ASV_1930	0	0.67	0.67	0	0	0	0	0	0	0	0	1.33	Archaea	Halobacterot	Halobacteria	Halobacterale	Halomicrobia	Halomicrobium
ASV_1931	0	0	0	0	0	0	0	0	0	0	0	0	Bacteria	Actinobacter	Acidimicrobii	IMCC26256	NA	NA
ASV_1932	0	0	0	0	0	0	0.5	0	0	0	0	3.33	Bacteria	Firmicutes	Bacilli	Bacillales	Bacillaceae	Aeribacillus
ASV_1933	2.67	0	0	0	0	0	0	0	0	0	0	0	Bacteria	Actinobacter	Actinobacter	Nitriliruptora	Nitriliruptora	NA
ASV_1934	1.33	0	0	0	0.33	0	0	0	0	2	1.33	0	Bacteria	Actinobacter	Actinobacter	Pseudonocar	Pseudonocar	Amycolatopsis
ASV_1935	0.33	0	0	0.33	0	0	0	0	0	0	0	0	Bacteria	Chloroflexi	Ktedonobact	C0119	NA	NA
ASV_1936	0.67	0	1	0.33	0	0	0	0	0	0	0	0	Bacteria	Proteobacter	Alphaproteol	Rhizobiales	Methyloligell	Methyloceanibacter
ASV_1937	1.33	0.33	0	1	0	0.67	0.5	0	0	0	0	0	Bacteria	Chloroflexi	Chloroflexia	Thermomicr	JG30-KF-CM4	NA
ASV_1938	0	0	1	0	0	0.33	0	0	0	0	0	0	Bacteria	Actinobacter	MB-A2-108	NA	NA	NA
ASV_1939	0	0	0.33	0	0	0	0	0	0	35	0	0	Bacteria	Firmicutes	Bacilli	Thermicanale	Thermicanac	Thermicanus
ASV_1940	0	0.33	0	0	0	0	0	0	0	0	0	0	Bacteria	Firmicutes	Bacilli	Thermicanale	Thermicanac	Thermicanus
ASV_1941	0	0	0	0	0	1	2.5	0	0	0	0	0	Bacteria	Actinobacter	Actinobacter	Corynebacte	Corynebacte	Corynebacterium
ASV_1942	0.67	0	0.33	0	0	0	0	0.33	0	0	0	0.33	Bacteria	Bacteroidota	Bacteroidia	Cytophagales	Hymenobact	Pontibacter
ASV_1943	0	0	0	0	0	0.33	1	0.33	0	0	0	0	Bacteria	Proteobacter	Alphaproteol	Acetobactera	Acetobactera	Belnapia
ASV_1944	0	0	0	0	0.33	0	0	2	0	0	0	0	Bacteria	Proteobacter	Alphaproteol	Azospirillales	Azospirillace	NA
ASV_1945	0	2.33	0.67	0	0.33	0	0	0	0	0	0	1	Bacteria	Actinobacter	NA	NA	NA	NA
ASV_1946	0	3.33	0	0	0	0	0	0	0	0	0	0	Bacteria	Bacteroidota	Bacteroidia	Bacteroidales	Prevotellacea	Prevotella_9
ASV_1947	0	0	0	0	0	1.33	3.5	0	0	0	0	0	Bacteria	Proteobacter	Alphaproteol	Sphingomona	Sphingomona	Sphingomonas
ASV_1948	0	0	0	0	0	0	0	0	0	0	0	0	Bacteria	Actinobacter	MB-A2-108	NA	NA	NA
ASV_1949	0	0	0	0	0	0	0	0	0	0	0	0	Bacteria	Proteobacter	Alphaproteol	Rhodobacter	Rhodobacter	Paracoccus
ASV_1950	0	0	3.67	0	0	0	1.5	0	0	0	0	0	Bacteria	Actinobacter	Actinobacter	Micrococcale	Microbacteria	Rathayibacter
ASV_1951	0	0	0	0.33	3.67	0	0	0	0	0	0	0	Bacteria	Acidobacteri	Acidobacteria	Bryobacterale	Bryobacterac	Bryobacter
ASV_1952	0	0	0	0	0.33	4.33	0.5	0	0	0	0	0	Bacteria	Actinobacter	Acidimicrobii	NA	NA	NA
ASV_1953	0	0	0	0	0	0	0	0	0	0	0	0	Bacteria	Firmicutes	Sulfobacillia	Sulfobacillale	Sulfobacillac	Sulfobacillus
ASV_1954	0	0.67	0	0	0	0	0	0	0	0	0	0.67	Bacteria	Proteobacter	Gammaprote	Legionellales	Legionellacea	Legionella
ASV_1955	0.67	0	0.67	0	0	0	2	0	0	2	0	0	Bacteria	Proteobacter	Alphaproteol	Rhizobiales	Beijerinckiac	Psychroglaciecola
ASV_1956	0	0	0.33	0	0	1	0.5	0.33	0	0	0	0	Bacteria	Proteobacter	Gammaprote	Burkholderia	SC-I-84	NA
ASV_1957	0	0	0	0.33	0	0	0	0.33	0	0	0	0	Bacteria	Verrucomicro	Verrucomicro	Chthoniobact	Chthoniobact	Candidatus Udaeobacter
ASV_1958	0	0	0.33	1	0	0	0.5	0.33	0	0	0	0	Bacteria	Actinobacter	Actinobacter	0319-7L14	NA	NA
ASV_1959	0	0	1	0.67	0	0.67	0.5	0.33	0	0	0	0	Bacteria	Actinobacter	Thermoleopl	Solirubrobact	Solirubrobact	Conexibacter
ASV_1960	0	0.67	0	0	0	0	0	0	0	0	0	0	Bacteria	Proteobacter	Gammaprote	Pseudomona	Moraxellacea	Acinetobacter
ASV_1961	0	0	0	0.33	0.33	0.33	1	0	0	0	0	0	Bacteria	Armatimonac	Armatimonac	Armatimonac	NA	NA
ASV_1962	0	0	0	1	0	1	1.5	0	0	0	0	0	Bacteria	Bdellovibrion	Oligoflexia	0319-6G20	NA	NA
ASV_1963	1.67	0	0	0	0	0.33	0	0	1	0	0	0	Bacteria	NA	NA	NA	NA	NA
ASV_1964	0	0	0	0	0	0	0	0.67	0	0	0	0	Bacteria	Firmicutes	Bacilli	Alicyclobacill	Alicyclobacill	NA
ASV_1965	0	0	0	0	0	0	0	0	0	0	0	0	Bacteria	Proteobacter	Gammaprote	Xanthomona	Xanthomona	Vulcaniibacterium
ASV_1966	0	0	0	0	0	0	0	0	0	0	0	0	Bacteria	Bacteroidota	Bacteroidia	Bacteroidales	Muribaculace	NA
ASV_1967	0	0	0	4	0	0	0	0	0	0	0	0	Bacteria	Proteobacter	Alphaproteol	Rhizobiales	Xanthobacte	NA
ASV_1969	0	0	0	0	0	0	0	0	21	0	0	0	Bacteria	Proteobacter	Gammaprote	Burkholderia	Methylophila	NA
ASV_1970	0	0	0	0	0	0	0	0	0	0	4	0	Bacteria	Actinobacter	Actinobacter	0319-7L14	NA	NA
ASV_1971	1.67	0	0	0	0	0	0	0	0	0	0	0	Bacteria	Gemmatimor	Longimicrobi	Longimicrobi	Longimicrobi	NA
ASV_1972	1	0.67	0	0	0	0.33	0	0	0	0	0	0	Bacteria	Planctomyce	Phycisphaera	Tepidisphaer	WD2101 soil	NA
ASV_1973	0.33	0	0.33	0	0	0	0	0	0	0	0	0	Bacteria	Actinobacter	Thermoleopl	Solirubrobact	67-14	NA
ASV_1974	0	0	0	0	0	1	1	0.33	0	0	0.67	0	Bacteria	Chloroflexi	Chloroflexia	Thermomicr	JG30-KF-CM4	NA
ASV_1975	0	0	0	0	0	1	0.5	1.33	0	0	0	0	Bacteria	RCP2-54	NA	NA	NA	NA
ASV_1976	0	0	0	0	0	0	0	0	0	0	0	0	Bacteria	Bacteroidota	Bacteroidia	Cytophagales	NA	NA
ASV_1977	0	0	0	0	0	0	0	0	0	0	0	0	Bacteria	Firmicutes	Bacilli	Exiguobacter	Exiguobacter	Exiguobacterium
ASV_1978	0	0	0.33	0	0	1.33	1	0.33	0	0	0	0.33	Bacteria	Acidobacteri	Vicinamibact	Vicinamibact	Vicinamibact	NA
ASV_1979	0	0	0	0	0	0	0	0	26	0	0	0	Bacteria	Actinobacter	Actinobacter	0319-7L14	NA	NA
ASV_1980	0	0	0	0	0	0	0	6.33	0	0	0	0	Bacteria	Chloroflexi	NA	NA	NA	NA
ASV_1981	0.67	0	1	0	0	0	0	0	0	0	0.33	0	Bacteria	Actinobacter	Actinobacter	Frankiales	Geodermatop	Geodermatophilus
ASV_1982	0.67	0	1.33	0	0	0	0	0	0	0	0	0	Bacteria	Actinobacter	Actinobacter	0319-7L14	NA	NA
ASV_1983	0	0	0	0	0	0	1	0	0	0	0	0	Bacteria	Bacteroidota	Bacteroidia	Chitinophaga	Chitinophaga	Flavisolibacter
ASV_1984	0	0	0.33	0	0	0	1	0	0	2	0	0	Bacteria	Acidobacteri	Blastocatellia	Blastocatellal	Blastocatellac	Blastocatella
ASV_1985	0	0	0	0	0	0.67	4	0	0	0	0	0	Bacteria	Actinobacter	Actinobacter	Corynebacte	Corynebacte	Corynebacterium
ASV_1986	0.33	0	0.33	1.33	0	0	0	0	0	0	0	0	Bacteria	Verrucomicro	Verrucomicro	Chthoniobact	Chthoniobact	Chthoniobacter
ASV_1987	0	0	0.33	0	0	0	0	0	0	0	0	0	Bacteria	Patescibacter	Saccharimon	Saccharimon	NA	NA
ASV_1988	0	0	0	0	0	0	0	0	0	0	0	0	Bacteria	Proteobacter	Gammaprote	Burkholderia	Oxalobactera	Herbaspirillum
ASV_1989	0	0	0	0	0	0	0	0	0	0	0	3	Bacteria	Proteobacter	Gammaprote	Pseudomona	Moraxellacea	Acinetobacter
ASV_1990	0	0	0	0	1.67	0	0	0	0	0	0	0	Bacteria	Actinobacter	Actinobacter	Pseudonocar	Pseudonocar	Actinomycetospora
ASV_1991	0	0	0	0	0	2.33	0	0	0	0	0	0	Bacteria	Chloroflexi	AD3	NA	NA	NA

ASV_1992	0	0	0	0	0	0	0	4.67	0	0	0	0	Bacteria	Actinobacter	Actinobacter	Pseudonocar	Pseudonocar	NA
ASV_1993	0	0	0	0	0	0	0	0.67	0	0	3	0	Bacteria	Actinobacter	Rubrobacteri	Rubrobactera	Rubrobacteri	Rubrobacter
ASV_1994	0	0	0	0	0	0	0	0	0	0	0	0	Bacteria	Actinobacter	Thermoleoph	Solirubrobact	Solirubrobact	Conexibacter
ASV_1995	0.33	0	0	0.33	0	0	0	0.33	0	0	0	0	Bacteria	Patescibacter	NA	NA	NA	NA
ASV_1996	0.67	0	2	0	0	0	0	0	0	0	0	0	Bacteria	Proteobacter	Gammaprote	Pseudomona	Pseudomona	Pseudomonas
ASV_1997	0.67	0	0	1	0	0.67	0.5	0.67	0	0	0	0	Bacteria	Chloroflexi	Chloroflexia	Thermomicro	JG30-KF-CM4	NA
ASV_1998	0	0	0	0	0	0	0	0	0	0	0	0	Bacteria	Actinobacter	Thermoleoph	Gaiellales	NA	NA
ASV_1999	0	0	0	0	0.33	0.33	1.5	0.33	0	0	0	0.33	Bacteria	Actinobacter	Thermoleoph	Gaiellales	NA	NA
ASV_2000	3	0	0	0	0	0	0	0	0	0	0	0	Bacteria	Firmicutes	Bacilli	Caldalkalibaci	Caldalkalibaci	Caldalkalibacillus
ASV_2001	0	0.33	0	0.33	0	0	0	0	0	0	0	0	Bacteria	Actinobacter	Actinobacter	Propionibact	Nocardioidac	Nocardioides
ASV_2002	0	0	0.33	0.33	0	0.33	0.5	0	0	0	0	0	Bacteria	Actinobacter	Thermoleoph	Solirubrobact	Solirubrobact	Solirubrobacter
ASV_2003	1	0	0	0	0	0	0	0.33	0	0	0	0	Bacteria	Proteobacter	Gammaprote	Burkholderia	Burkholderia	Ralstonia
ASV_2004	0	0	0	0	0	0	0	0	0	0	0	3.67	Bacteria	Firmicutes	Bacilli	Caldalkalibaci	Caldalkalibaci	Caldalkalibacillus
ASV_2005	0.67	0	0.33	0	0	0	0	0	0	0	0.33	0	Bacteria	Actinobacter	Thermoleoph	Gaiellales	NA	NA
ASV_2006	0	0	0	1	0	0	0	0	0	0	0	0	Bacteria	Actinobacter	Actinobacter	Pseudonocar	Pseudonocar	Crossiella
ASV_2007	1.67	0	0.33	0	0	0	0	0	0	0	0	0	Bacteria	Chloroflexi	Chloroflexia	Thermomicro	JG30-KF-CM4	NA
ASV_2008	0.67	0	0.33	0	0	0	0	0	0	0	0	0	Bacteria	Actinobacter	Acidimicrobii	NA	NA	NA
ASV_2009	0	0	0.67	0.33	0	0	0.5	0.33	0	0	0	0	Bacteria	Actinobacter	Actinobacter	Nitriliruptora	Nitriliruptora	NA
ASV_2010	0	0	0	0.67	0	0	0.5	0.67	0	0	0	0.67	Bacteria	Actinobacter	Acidimicrobii	Microtrichale	Ilumatobacte	NA
ASV_2011	0	0.67	0	0.33	0	0.33	1.5	0.33	0	0	0	0	Bacteria	Bdellovibrion	Bdellovibrion	Bacteriovora	Bacteriovora	Peredibacter
ASV_2012	0.67	0	0	0	1.33	0	0	1	0	0	0	0	Bacteria	Verrucomicro	Verrucomicro	Pedosphaera	Pedosphaera	NA
ASV_2013	0	0	0	0	0	0	0	0	0	0	0	2.67	Bacteria	Proteobacter	Gammaprote	Burkholderia	NA	NA
ASV_2014	2.67	0	0	0	0	0	0	0	0	0	0	0	Bacteria	Proteobacter	Gammaprote	Pseudomona	Cellvibrionac	Cellvibrio
ASV_2015	0	0	0	0	0	0	0	0	3	0	0	0	Bacteria	Actinobacter	Thermoleoph	Solirubrobact	NA	NA
ASV_2016	0	0	2.33	0	0	0	0	0	0	0	0	0	Bacteria	Planctomycet	Planctomycet	Gemmatales	Gemmatacea	NA
ASV_2017	0	0	0	0	0	0	0	2	0	0	0	0	Archaea	Thermoplasm	Thermoplasm	Thermoplasm	Thermoplasm	A-plasma
ASV_2019	0	0	0	0	0	0	0	4.33	0	0	0.33	0	Bacteria	NA	NA	NA	NA	NA
ASV_2020	0.67	0	0	0	0	0	0	0	0	6	0	0	Bacteria	Proteobacter	Gammaprote	Burkholderia	NA	NA
ASV_2021	0.33	0	0	0	0	0	0	0	0	0	0.33	0	Bacteria	Actinobacter	Thermoleoph	Solirubrobact	67-14	NA
ASV_2022	0	0	0	0	0.33	1	1	0	0	0	0	0	Bacteria	Chloroflexi	TK10	NA	NA	NA
ASV_2023	0	0.67	0.67	0	0	0.67	0	0	0	0	0	0	Bacteria	Actinobacter	Thermoleoph	Solirubrobact	67-14	NA
ASV_2024	1	0	0	1	0	0	0	0	0	0	0	0	Bacteria	Firmicutes	Bacilli	Paenibacillale	Paenibacillac	Paenibacillus
ASV_2025	0.33	0	0	0.33	0.33	0	0.5	0	0	0	1.33	0	Bacteria	Planctomycet	Phycisphaera	Tepidisphaer	WD2101 soil	NA
ASV_2027	0	0	0	0	0	0	0	0.67	0	0	0.67	0.33	Bacteria	Proteobacter	Gammaprote	Burkholderia	Oxalobactera	Telluria
ASV_2028	0	0	0	0	0.67	0.67	0.5	0.33	0	0	0	0.33	Bacteria	Patescibacter	NA	NA	NA	NA
ASV_2029	0	0	0	0	0	0.67	1	0.67	0	0	0	0	Bacteria	Verrucomicro	Chlamydiae	Chlamydiales	Chlamydiacea	NA
ASV_2030	0	0	0	0	0	0	0	0	0	0	0	0	Bacteria	Proteobacter	Alphaproteol	NA	NA	NA
ASV_2032	0	0	0	0	0	0	0	0	0	0	0	0	Bacteria	Firmicutes	NA	NA	NA	NA
ASV_2033	0	0	0	0	0	0.33	0	0	0	0	0	0	Bacteria	Firmicutes	Bacilli	Alicyclobacill	Alicyclobacill	Alicyclobacillus
ASV_2034	0.33	0	0	0.33	0.33	0	0	0	0	0	0	0.67	Bacteria	Actinobacter	Actinobacter	0319-7L14	NA	NA
ASV_2035	0	0	0.67	0	0	0	0	0	0	0	0	0	Bacteria	Proteobacter	Gammaprote	Xanthomona	Rhodanobact	Ahniella
ASV_2036	0	0	0.67	0	0	0	1.5	0	0	0	0	0.67	Bacteria	Bdellovibrion	Oligoflexia	0319-6G20	NA	NA
ASV_2037	0	0.33	0	0	0	2	2	0	0	0	0	0	Bacteria	Proteobacter	Gammaprote	Burkholderia	Gallionellace	NA
ASV_2038	0.33	0	0	0	0	0.33	1	0	0	0	0	0	Bacteria	Myxococcota	Myxococcia	Myxococcale	Myxococcace	NA
ASV_2039	0	0.33	0	0	0	3	0	0	0	0	0	0	Bacteria	Proteobacter	Gammaprote	Xanthomona	Xanthomona	Xanthomonas
ASV_2040	0	0	0	0	0	0	1	0	0	0	1.33	0	Bacteria	Firmicutes	Bacilli	Bacillales	Bacillaceae	NA
ASV_2041	0.33	0	0.67	0.33	0	0.33	1	0.33	0	0	0	0	Bacteria	Acidobacteri	Blastocatellia	Blastocatellal	Blastocatellac	Blastocatella
ASV_2042	0.67	0.33	0	0.33	0	0	0	0	0	0	0	0	Bacteria	Actinobacter	Thermoleoph	Solirubrobact	Solirubrobact	Solirubrobacter
ASV_2043	0	0	0.33	0	0	0	0	0	0	0	0	0	Bacteria	Actinobacter	Actinobacter	Propionibact	Nocardioidac	Nocardioides
ASV_2044	0	0	0	0	0	0	0	1.33	0	0	0	0	Bacteria	Planctomycet	Planctomycet	Gemmatales	Gemmatacea	Gemmata
ASV_2045	0	0	0	0	0	0	0	0	0	0	0	1	Bacteria	Firmicutes	Bacilli	Bacillales	Bacillaceae	Filobacillus
ASV_2046	0	0	0.67	0	0	0	0.5	0.33	0	0	0	0	Bacteria	Actinobacter	Actinobacter	Corynebacte	Mycobacteria	Mycobacterium
ASV_2047	0	0	1.67	0	0	0	0	0	0	0	0.33	0	Bacteria	Actinobacter	Actinobacter	NA	NA	NA
ASV_2048	0	0	0	0	0	0	0	0	20	0	0	0	Bacteria	Actinobacter	Rubrobacteri	Rubrobactera	Rubrobacteri	Rubrobacter
ASV_2049	0	0	0	0	0	0	0	0	0	0	0	0	Bacteria	Firmicutes	Sulfobacillia	Sulfobacillale	Sulfobacillace	Sulfobacillus
ASV_2050	0	0	0	0	0	0	0	0	0	0	0	0	Bacteria	Firmicutes	Bacilli	Staphylococc	Staphylococc	NA
ASV_2051	0	0	0	0	0	0	0	0	0	0	0	0	Bacteria	Actinobacter	Actinobacter	Corynebacte	Corynebacte	Corynebacterium
ASV_2052	0.33	0	0	0	0	0	0	0	0	0	0	0	Bacteria	Actinobacter	Actinobacter	Corynebacte	Corynebacte	Corynebacterium
ASV_2053	0	0	0	0.33	0	0	0	0	0	0	0	0	Bacteria	Actinobacter	Thermoleoph	Solirubrobact	Solirubrobact	Solirubrobacter
ASV_2054	0	0	0	0	0	0	0	0	0	0	0	0	Bacteria	Firmicutes	Bacilli	Staphylococc	Staphylococc	Staphylococcus
ASV_2055	0	0	0	0	1	0	0	0	0	0	0	0	Bacteria	Proteobacter	Alphaproteol	Sphingomona	Sphingomona	NA
ASV_2056	0	0	0	0	0	0.33	5	0	0	0	0	0	Bacteria	Actinobacter	Actinobacter	Micrococcale	Cellulomonac	Oerskovia
ASV_2057	0	0	0	0.33	0	0.33	1	0.33	0	0	0	0	Bacteria	Myxococcota	Polyangia	mle1-27	NA	NA
ASV_2058	0.33	0	0	0	0	0	0	0	0	0	0	0	Bacteria	Gemmatimor	Longimicrobi	Longimicrobi	Longimicrobi	NA
ASV_2059	0	0	0	0	0	1	2.5	0	0	0	0	0	Bacteria	Proteobacter	Alphaproteol	Caulobactera	Caulobactera	Brevundimonas
ASV_2060	0.33	0	0	0	0	0.67	2	0.67	0	0	0.33	0.33	Bacteria	Chloroflexi	Chloroflexia	Thermomicro	AKYG1722	NA
ASV_2061	0	0	0	0	0	0	0	0	0	0	0	0	Bacteria	Acidobacteri	Vicinamibact	Vicinamibact	NA	NA
ASV_2062	0	0	0	0	0	0	0	0	0	0	0	0	Bacteria	RCP2-54	NA	NA	NA	NA
ASV_2063	0	0	0	0.67	0	0	1	0.33	0	0	0	0.33	Bacteria	Myxococcota	Polyangia	Polyangiales	Polyangiacea	Pajaroellobacter
ASV_2064	0	0	0	0	0	0	0	0	0	0	0	0	Bacteria	Chloroflexi	AD3	NA	NA	NA
ASV_2065	0	0	0	0	0	0	0	0	0	0	0	0	Bacteria	Actinobacter	Actinobacter	Corynebacte	Corynebacte	Corynebacterium
ASV_2066	0	0	0	0	0	0	0	0	12	0	0	0	Archaea	Thermoplasm	Thermoplasm	Thermoplasm	Thermoplasm	A-plasma
ASV_2067	0	0	0	0	0	0	0	0	0	0	0	0	Bacteria	Actinobacter	Rubrobacteri	Rubrobactera	Rubrobacteri	Rubrobacter
ASV_2068	1	0	0	0	0	0	0	0	0	0	0	0	Bacteria	Planctomycet	Planctomycet	Isosphaerales	Isosphaerace	NA
ASV_2069	0	0	0	0.33	0	0	0	0	0	0	0	0	Bacteria	Actinobacter	Actinobacter	Frankiales	NA	NA
ASV_2070	0	0.67	0.33	0	0	0	0.5	0	0	0	0	0.33	Bacteria	Actinobacter	Acidimicrobii	IMCC26256	NA	NA
ASV_2071	0.33	0.33	0	0.33	0	0.33	0	0	0	0	0	0.33	Bacteria	Actinobacter	Thermoleoph	Gaiellales	NA	NA
ASV_2072	0.67	0	0	0.33	0	0	0	0.33	0	0	1	0	Bacteria	Actinobacter	Rubrobacteri	Rubrobactera	Rubrobacteri	Rubrobacter
ASV_2073	1.33	0	0	0	0	0	0	0	0	0	0	0	Bacteria	Proteobacter	Gammaprote	Pseudomona	Pseudomona	Pseudomonas
ASV_2075	0	0	0	0	0.67	0	1.5	0.33	0	0	0	0	Bacteria	Planctomycet	Phycisphaera	Tepidisphaer	WD2101 soil	NA
ASV_2076	1.33	0	0	0	0	0	0	0	0	0	0	0	Bacteria	Firmicutes	Bacilli	Staphylococc	Staphylococc	Staphylococcus
ASV_2077	0	0	0	0	0	0.33	1	0	0	0	0	0	Bacteria	Actinobacter	Actinobacter	Micrococcale	Intrasporangi	Knoellia
ASV_2078	0	0	0	0	0	0	0	0	0	0	0	0	Bacteria	Actinobacter	Thermoleoph	Solirubrobact	Solirubrobact	NA
ASV_2079	0	0	0	0	0	0	0	0	0	0	4.33	0	Bacteria	Proteobacter	Gammaprote	Burkholderia	Comamonada	Paucibacter
ASV_2080	0	0	0	0.33	0	0	1	0	0	0	0	0	Bacteria	Actinobacter	Thermoleoph	Gaiellales	NA	NA
ASV_2081	0.33	0	0	0	0	0	0	0	0	0	0	0	Bacteria	Proteobacter	Gammaprote	Burkholderia	TRA3-20	NA
ASV_2082	0.67	0.67	0	0	0	0	0	0	0	3	0	0	Bacteria	Chloroflexi	Chloroflexia	Thermomicro	JG30-KF-CM4	NA
ASV_2083	0	0	0.67	0	0	0	0	0	0	0	0	0	Bacteria	Patescibacter	Saccharimona	Saccharimona	NA	NA
ASV_2084	0	0.67	0	0	2.33	0	0	0.33	0	0	0	0	Bacteria	Actinobacter	Acidimicrobii	Acidimicrobia	Acidimicrobia	Ferrimicrobium
ASV_2085	0.33	0	0	0	0	0	0	0	0	2	0	2.67	Bacteria	Proteobacter	Gammaprote	Burkholderia	Comamonada	NA
ASV_2086	0	0	0	0	0.67	0	0	0	2	0	0	0	Bacteria	Proteobacter	Alphaproteol	Sphingomona	Sphingomona	Ellin6055
ASV_2087	0	0	0.67	0.33	0.33	0.33	0	0	0	0	0	0	Bacteria	Planctomycet	Phycisphaera	Tepidisphaer	WD2101 soil	NA
ASV_2088	0	0.33	0	1	0	0	0	0	0	0	0	0	Bacteria	Firmicutes	Bacilli	Alicyclobacill	Alicyclobacill	Tumebacillus
ASV_2089	0	0	0	0	0	3.33	0	0	0	0	0	0	Bacteria	Acidobacteri	Acidobacteria	Subgroup 2	NA	NA
ASV_2090	0	0	2	0	0	0	0	0	0	0	0.67	0	Archaea	Crenarchaeo	Bathyarchaei	NA	NA	NA
ASV_2091	0	0	0	0	0	0	0	0	0	0	0	0	Bacteria	Proteobacter	Gammaprote	Burkholderia	Oxalobactera	Janthinobacterium
ASV_2092	0.67	0.33	0	0.67	0	0	0	0	0	0	0	0	Bacteria	Proteobacter	Gammaprote	Methylococc	Methylomon	Methylobacter
ASV_2093	0	0	0.33	0	0	0	0	0	0	0	0	0	Bacteria	Actinobacter	Acidimicrobii	Microtrichale	NA	NA
ASV_2094	0.33	0	0	0	0	0.33	1.5	0	0	0	0	0	Bacteria	Bacteroidota	Bacteroidia	Sphingobacte	KD3-93	NA
ASV_2095	0.67	0.33	0	0.33	0	0	1	0.33	0	0	0	0	Bacteria	Actinobacter	Thermoleoph	Gaiellales	NA	NA
ASV_2096	0	0	0.67	0	0	0.33	0	0.33	0	0	0	0	Bacteria	Acidobacteri	Blastocatellia	Pyrinomonac	Pyrinomonad	RB41
ASV_2097	0.67	0	0	0	0	1	1	0.67	0	0	0.67	0.67	Bacteria	Planctomycet	Phycisphaera	Tepidisphaer	WD2101 soil	NA
ASV_2098	0	0	0	0	0	0	0	0	20	0	0	0	Bacteria	RCP2-54	NA	NA	NA	NA
ASV_2099	0	0	0.33	0	0	0	6	0	0	0	0	0	Bacteria	Actinobacter	Rubrobacteri	Rubrobactera	Rubrobacteri	Rubrobacter
ASV_2100	0	0	0	0	0	0	0	0	23	0	0	0	Bacteria	Actinobacter	Acidimicrobii	IMCC26256	NA	NA
ASV_2101	0	0	0	0	0	0	0	0	0	0	0	0	Bacteria	Firmicutes	Bacilli	Bacillales	NA	NA
ASV_2102	1	0	0	0	0	0	0	0	0	0	0	0	Bacteria	Actinobacter	Acidimicrobii	NA	NA	NA
ASV_2103	0	0	0.67	0	0	0	0.5	0	0	0	0	0	Bacteria	Actinobacter	Thermoleoph	Solirubrobact	NA	NA
ASV_2104	0.33	0	0	0	0	0	0	0	0	0	0	0	Bacteria	Bacteroidota	Bacteroidia	Chitinophaga	Chitinophaga	NA
ASV_2105	1	0	0.67	0	0	0	0	0	0	0	0.33	0	Bacteria	Armatimonac	Armatimonac	Armatimonac	Armatimonac	Armatimonas
ASV_2106	0	0	0.67	0	0	0.33	2.5	0	0	0	0	0	Bacteria	Proteobacter	Gammaprote	Burkholderia	Sutterellacea	NA
ASV_2107	0.33	0	0	0	0	0	0	0.33	0	0	0	0	Bacteria	Actinobacter	Thermoleoph	Solirubrobact	67-14	NA
ASV_2108	0.33	0.67	0	0.33	0	0.33	0	0	0	0	0	0	Bacteria	Verrucomicro	Verrucomicro	Chthoniobact	Chthoniobact	Chthoniobacter
ASV_2109	0	0	0.33	0	0	0.67	0	0	0	0	0	0	Bacteria	Verrucomicro	Verrucomicro	Chthoniobact	Chthoniobact	Candidatus Udaeobacter
ASV_2110	1.67	0	0	0	0	0	0	0	0	0	0	0	Bacteria	Firmicutes	Bacilli	Caldalkalibaci	Caldalkalibaci	Caldalkalibacillus
ASV_2111	0.33	0	0	0	0	0	0.5	0.67	0	0	0	0.33	Bacteria	Planctomycet	Planctomycet	Gemmatales	Gemmatacea	NA
ASV_2112	0	0	0	0	0	0	1	1	0	0	0	0	Bacteria	Firmicutes	Sulfobacillia	Sulfobacillale	Sulfobacillace	Sulfobacillus
ASV_2113	0	0	0	0	0	0	0	0	0	0	0	0	Bacteria	Bacteroidota	Bacteroidia	Bacteroidales	Dysgonomon	Dysgonomonas
ASV_2114	0	0	0	0	0	0	0	3	0	0	0	0	Bacteria	Proteobacter	Alphaproteol	Azospirillales	Azospirillacea	NA
ASV_2115	0.33	0	0.33	0	0	0	1	0	0	0	0	0	Bacteria	Actinobacter	Thermoleoph	Gaiellales	NA	NA
ASV_2116	0.67	0	0	0	0	0	0.5	0	0	0	0	0	Bacteria	Bacteroidota	Bacteroidia	Sphingobacte	Sphingobacte	Pedobacter
ASV_2117	0.67	0	0	0	0.67	0	0	0	0	0	0.33	0	Bacteria	Chloroflexi	Chloroflexia	Thermomicro	JG30-KF-CM4	NA
ASV_2118	0	0	0	0.33	0	0	0	0	0	0	0	0	Bacteria	Bacteroidota	Bacteroidia	Chitinophaga	Chitinophaga	Segetibacter
ASV_2119	0	0	0	0	0.33	0.67	0.5	0	0	2	0	0	Bacteria	Gemmatimor	Gemmatimor	Gemmatimor	Gemmatimor	NA
ASV_2120	0	0	0.33	0	0.67	0.33	0	0.67	0	0	0	0	Bacteria	Verrucomicro	Verrucomicro	Verrucomicro	Verrucomicro	NA
ASV_2121	0	0	0	1	0	0.67	0	0	0	0	0	0	Bacteria	Actinobacter	Thermoleoph	Gaiellales	Gaiellaceae	Gaiella
ASV_2122	0	0	0.33	0.33	0	0	0	0	0	0	0	0	Bacteria	Myxococcota	Polyangia	Haliangiales	Haliangiaceae	Haliangium
ASV_2123	0	0	0	0.33	0	0	2.5	0.33	0	0	0	0	Bacteria	Myxococcota	Polyangia	Polyangiales	BIrii41	NA
ASV_2124	0	0	0	1.67	0	0	0	0	0	0	0	0	Bacteria	Actinobacter	Actinobacter	0319-7L14	NA	NA
ASV_2125	0	0	0	0	0	0	0	0	0	0	0	0	Archaea	Crenarchaeo	Nitrososphae	NA	NA	NA
ASV_2126	0	0	0	0	0	0.67	2	0.33	0	0	0.67	0	Bacteria	Actinobacter	Rubrobacteri	Rubrobactera	Rubrobacteri	Rubrobacter
ASV_2127	0	0	0	0	0	0	0	0.33	0	0	2	0	Bacteria	Bacteroidota	Bacteroidia	Chitinophaga	Chitinophaga	Segetibacter
ASV_2128	0	0	0	0	0	0	0	0	0	0	0	0	Bacteria	Actinobacter	Actinobacter	Frankiales	Geodermato	Geodermatophilus
ASV_2129	0	0	0	0	0	0	0	0	0	0	3.33	0	Bacteria	Firmicutes	Bacilli	NA	NA	NA
ASV_2130	0	0	0	0	0	0	0	0	0	0	0	0	Bacteria	Actinobacter	Actinobacter	NA	NA	NA
ASV_2131	0	0	0	0	0	0	0	0	0	3	0.33	0	Bacteria	Actinobacter	Thermoleoph	Solirubrobact	67-14	NA
ASV_2132	0	0	0	0.33	0	0	0.5	0	0	0	0	0.33	Bacteria	Chloroflexi	Chloroflexia	Thermomicro	JG30-KF-CM4	NA
ASV_2133	0	0	0	0	0.67	1.33	0	0	0	0	0	0	Archaea	Crenarchaeo	Thermoprote	Sulfolobales	Sulfolobacea	Acidianus
ASV_2134	0	0	0	0	0	0	0	1.33	0	0	0	0	Bacteria	Proteobacter	Alphaproteol	Rhizobiales	Beijerinckiac	NA
ASV_2135	0	0	0	0	0	0	0	0	0	0	2.67	0	Bacteria	Proteobacter	Gammaprote	Pseudomona	Halomonada	Halomonas
ASV_2136	0.33	0	0	0	0.33	0	0	0.33	0	0	0	0	Bacteria	Verrucomicro	Verrucomicro	Pedosphaera	Pedosphaera	NA
ASV_2137	1	0	0	0	0	0	0	0	3	0	0	0	Archaea	Crenarchaeo	Thermoprote	Sulfolobales	Sulfolobacea	Stygiolobus
ASV_2138	0	0	0	0	0	0	0	0	2	0	0	1	Bacteria	Proteobacter	Gammaprote	Pseudomona	Moraxellacea	Enhydrobacter
ASV_2139	0	0	0	0	0	0	0	0	0	0	1.67	0	Bacteria	Planctomycet	Phycisphaera	Tepidisphaer	WD2101 soil	NA
ASV_2140	0.33	0	0	0.67	0	0	0	0	0	0	0	0.33	Bacteria	Abditibacteri	Abditibacteri	Abditibacteri	Abditibacteri	Abditibacterium
ASV_2141	0.33	0	0	0.33	0	0	1	0	0	0	0	0	Bacteria	Chloroflexi	Chloroflexia	Thermomicro	JG30-KF-CM4	NA
ASV_2142	0	0	0	0	0	0.33	0	0	0	0	0	0	Bacteria	Bacteroidota	Bacteroidia	Chitinophaga	Chitinophaga	Ferruginibacter

ASV_2143	0	0	0.67	0.33	0	0.33	0	0	0	0	0	0	Bacteria	Acidobacteri	Blastocatellia	Pyrinomonac	Pyrinomonac	RB41
ASV_2144	0	0	1	0	0	0.33	1.5	0	0	0	0	0	Bacteria	Acidobacteri	Blastocatellia	Pyrinomonac	Pyrinomonac	RB41
ASV_2145	0	0	0.33	0	0	0	0	0	0	0	0	0	Bacteria	Patescibacte	Saccharimon	Saccharimon	NA	NA
ASV_2146	0	0	0	0	0	0	1	0.33	0	0	0.33	0.33	Bacteria	Actinobacter	Thermoleoph	Solirubrobact	67-14	NA
ASV_2147	0.33	0	0	0	0	0	0	0	0	0	0	0	Bacteria	NA	NA	NA	NA	NA
ASV_2148	0.33	0	0	0	0	0	0	0	0	0	0	0	Bacteria	Firmicutes	Desulfitobact	Desulfitobact	Desulfitobact	Desulfosporosinus
ASV_2149	0	0	0	0	0	0	0	0	16	0	0	0	Bacteria	Proteobacter	Gammaprote	Pseudomona	Halomonadac	Halomonas
ASV_2150	0	0	0	0	0	0	0	0	0	0	0	0	Bacteria	Actinobacter	Actinobacter	Micrococcale	Microbacteri	NA
ASV_2151	0	0	0	0	0	0	0	0	0	0	0	0	Bacteria	Firmicutes	Bacilli	Bacillales	Bacillaceae	NA
ASV_2152	0	0	0	0	0	0	0	0	0	0	0	3.67	Bacteria	Firmicutes	Negativicutes	Veillonellales	NA	NA
ASV_2153	0	0	0	0	0	1	2.5	0	0	0	0	0	Bacteria	Firmicutes	Bacilli	Paenibacillale	Paenibacillac	Ammoniphilus
ASV_2154	0	0	0	0	0	0	0	0	19	0	0	0	Bacteria	Gemmatimor	S0134 terrest	NA	NA	NA
ASV_2155	0.67	0	0	0.33	0	0	0	0	0	0	0	0	Bacteria	Proteobacter	Gammaprote	Pseudomona	Pseudomona	Pseudomonas
ASV_2156	0.33	0	0	0	0	0	0	0	0	0	0	0.33	Bacteria	Gemmatimor	Gemmatimor	Gemmatimor	Gemmatimor	NA
ASV_2157	0	0	0.67	0	0	0	0	0	0	0	0	0	Bacteria	Chloroflexi	Anaerolineae	SBR1031	NA	NA
ASV_2158	0.33	0.33	0	1	0	0.33	0	0	0	0	0	0	Bacteria	Acidobacteri	Vicinamibact	Vicinamibact	NA	NA
ASV_2159	0.33	0	0	1.33	0	0	1	0	0	0	0	0	Bacteria	Planctomyce	Planctomyce	Pirellulales	Pirellulaceae	Pirellula
ASV_2160	0	0	0	0	0	0	0.5	0	0	0	0	0.67	Bacteria	Actinobacter	Acidimicrobii	Microtrichale	Ilumatobacte	CL500-29 marine group
ASV_2161	0	0	0	0	0.33	0.33	0	0	0	0	0.33	0	Bacteria	Gemmatimor	Gemmatimor	Gemmatimor	Gemmatimor	Gemmatimonas
ASV_2162	0	0	0.67	0.33	0	0	1	0	0	2	0	0	Bacteria	Proteobacter	Gammaprote	Gammaprote	Unknown Fa	Candidatus Ovatusbacter
ASV_2163	0	0.33	0.33	0	0	0	0	0	0	0	0	0	Bacteria	Abditibacteri	Abditibacteri	Abditibacteri	Abditibacteri	Abditibacterium
ASV_2164	0.67	0	0	0.33	0	0	0	0	0	0	0	0	Bacteria	Proteobacter	Alphaproteol	Rhizobiales	NA	NA
ASV_2165	0	0.33	0	0	0	0	0	0	0	0	0	2.67	Bacteria	Actinobacter	Actinobacter	Corynebacte	Mycobacteri	Mycobacterium
ASV_2166	0	0	0	0.33	0	0.67	1.5	0.67	0	0	0	0	Bacteria	Proteobacter	Alphaproteol	Sphingomona	Sphingomona	Sphingomonas
ASV_2167	0	0	0	0	0	0	0	0	0	0	0	0	Bacteria	Firmicutes	Sulfobacillia	Sulfobacillale	Sulfobacillac	NA
ASV_2168	0	0	0	0	0	0	0	0	0	0	0.67	1	Bacteria	Actinobacter	Actinobacter	Corynebacte	Mycobacteri	Mycobacterium
ASV_2169	0	0	0	0	0	0.33	3.5	0	0	0	0	0	Bacteria	Fusobacterio	Fusobacteriia	Fusobacteria	Fusobacteria	Fusobacterium
ASV_2170	0	0.33	1	0.33	0	0	0	0	0	0	0	0	Bacteria	NA	NA	NA	NA	NA
ASV_2171	0	0	0	0	0	0	0	0	0	0	0	0	Bacteria	Firmicutes	Clostridia	Peptostrepto	Family XI	Peptoniphilus
ASV_2172	0	0	0	0	0	0	0	0	0	0	0	0	Bacteria	Proteobacter	Gammaprote	Beggiatoales	Beggiatoacea	NA
ASV_2173	0	0	0	0	0	0	0	0	0	0	0	0	Bacteria	Firmicutes	Bacilli	Bacillales	Planococcace	Lysinibacillus
ASV_2174	0	0	0	0	0	2.67	1	0	0	0	0	0	Bacteria	Firmicutes	Bacilli	Alicyclobacill	Alicyclobacill	Alicyclobacillus
ASV_2175	0	0	0	0	0	0	2.5	0	0	0	0	0	Archaea	Crenarchaeo	Bathyarchaei	NA	NA	NA
ASV_2176	0	0	0	0	0.33	0	0	0	0	0	0	0	Archaea	Thermoplasm	Thermoplasm	Thermoplasm	Thermoplasm	A-plasma
ASV_2177	0	0	0	0	0	0.33	0	0	0	0	0	0	Bacteria	Actinobacter	Acidimicrobii	NA	NA	NA
ASV_2178	0	0	0	0	0	3.33	1	0	0	0	0	0	Bacteria	Firmicutes	Sulfobacillia	Sulfobacillale	Sulfobacillac	NA
ASV_2179	0	0	0	0	0	0	0	0	0	0	3.33	0	Bacteria	Actinobacter	Actinobacter	Pseudonocar	Pseudonocar	Amycolatopsis
ASV_2180	0	0	0	0	0	0	0	0	0	0	2	0	Bacteria	Actinobacter	Actinobacter	Euzebyales	Euzebyaceae	NA
ASV_2181	0	0.33	0	0	0	0	0	0	0	0	0	0	Bacteria	Gemmatimor	Gemmatimor	Gemmatimor	Gemmatimor	Gemmatimonas
ASV_2182	0.67	0	0	0.67	0	0	0	0	0	0	0	0	Bacteria	Chloroflexi	Chloroflexia	Thermomicro	JG30-KF-CM4	NA
ASV_2183	0	0	0.33	0	0	0	0.5	0	0	0	0	0	Bacteria	Proteobacter	Alphaproteol	Rhizobiales	Xanthobacte	Rhodoplanes
ASV_2184	0	0	0	0	0.67	0	1.5	0	0	0	1.33	0	Bacteria	Gemmatimor	Longimicrobi	Longimicrobi	Longimicrobi	Longimicrobium
ASV_2185	0.33	0	0	0.67	0	0	0	0	0	0	0	0	Bacteria	Proteobacter	Gammaprote	Burkholderia	Comamonad	Tibeticola
ASV_2186	0.33	0	0	0	0	0	2	0	0	0	0.67	0	Bacteria	Planctomyce	Planctomyce	Planctomyce	NA	NA
ASV_2187	0	0.33	0	0	0.67	0	0	0	0	0	0	0	Bacteria	Actinobacter	Acidimicrobii	Acidimicrobi	Acidimicrobi	Ferrimicrobium
ASV_2188	0	0	0	0	0	0	0	0	0	0	1.33	0.67	Bacteria	Proteobacter	Gammaprote	Burkholderia	Comamonad	NA
ASV_2189	0	0	0	0	0	0	0	0	0	0	3	0	Bacteria	Armatimonac	NA	NA	NA	NA
ASV_2190	0	0	0.33	0	0	1.33	0	1	0	0	0.33	0	Bacteria	Bacteroidota	Bacteroidia	Chitinophaga	Chitinophaga	Ferruginibacter
ASV_2191	0	0	0	0	0	0	0	0.33	0	0	0	0	Bacteria	Actinobacter	Thermoleoph	NA	NA	NA
ASV_2192	0	0	0	0	0	0	0	0	0	0	0	0.33	Archaea	Thermoplasm	Thermoplasm	Thermoplasm	Thermoplasm	A-plasma
ASV_2193	0	0	0	0	2.67	0	0	0	0	0	0	0	Bacteria	Actinobacter	MB-A2-108	NA	NA	NA
ASV_2194	0	0	0	0	0	0	0	0	0	0	0	0	Archaea	Thermoplasm	Thermoplasm	Thermoplasm	Thermoplasm	A-plasma
ASV_2195	0	0	0	0	0	0	0	0	0	0	0	0	Bacteria	Actinobacter	Actinobacter	NA	NA	NA
ASV_2196	0	0	0	0	0	0	0	0	0	0	0	0	Bacteria	Actinobacter	Actinobacter	Pseudonocar	Pseudonocar	NA
ASV_2197	1.67	0	0	0	0	0	0	0	0	0	0	0	Bacteria	Proteobacter	Gammaprote	Burkholderia	Alcaligenace	GKS98 freshwater group
ASV_2198	0	0	0	0	0	0	0.5	0	0	0	0	0.33	Bacteria	Myxococcota	Myxococcia	Myxococcale	Myxococcace	NA
ASV_2199	0	0	0	0	0	0.33	1	0	0	0	0	0	Bacteria	Chloroflexi	TK10	NA	NA	NA
ASV_2200	0.67	0	0	0	0	0	0	0	0	0	0	0	Bacteria	Proteobacter	Gammaprote	Legionellales	Legionellace	Legionella
ASV_2201	0.33	0	0.33	0.67	0	0	0.5	0	0	2	0	0	Bacteria	Actinobacter	Actinobacter	Propionibact	NA	NA
ASV_2202	0.33	0	0.33	0	0	0	0.5	0	0	0	0	0	Bacteria	Actinobacter	Thermoleoph	Solirubrobact	67-14	NA
ASV_2203	0	0	0	0	0	0.33	0	1	0	0	0	0	Bacteria	Bacteroidota	Bacteroidia	Chitinophaga	Chitinophaga	Flavisolibacter
ASV_2204	0	0.33	1	0.33	0	0	0	0	0	0	0	0	Bacteria	Proteobacter	Alphaproteol	Rhizobiales	Beijerinckiac	Methylobacterium-Methylorubrum
ASV_2205	1.67	0	0	0	0	0	0	0	0	0	0	0	Archaea	Crenarchaeo	Thermoprote	Sulfolobales	Sulfolobacea	Stygiolobus
ASV_2206	0	0	0	0	0	0	0	0	0	0	0	0	Bacteria	Actinobacter	Actinobacter	0319-7L14	NA	NA
ASV_2207	0	0.33	0	0	0	0	0	0	0	0	0	0	Bacteria	Firmicutes	Bacilli	Bacillales	NA	NA
ASV_2208	0	0	0	0.33	0	0	0	0	0	0	0	0	Bacteria	Actinobacter	Thermoleoph	Gaiellales	Gaiellaceae	Gaiella
ASV_2209	0	0	0	0	0	0	1	0	0	0	4.67	0	Bacteria	Chloroflexi	Chloroflexia	Kallotenuales	AKIW781	NA
ASV_2210	0	0	0	0	0	0	0	0	0	0	0	0	Bacteria	Proteobacter	Gammaprote	Pseudomona	Moraxellacea	Acinetobacter
ASV_2211	0	0	0	0	0	0.67	0.5	0	0	0	0	0	Bacteria	Chloroflexi	Ktedonobact	C0119	NA	NA
ASV_2212	0	0	0	0	0	0.67	0	1	0	0	0	0	Archaea	Thermoplasm	Thermoplasm	Thermoplasm	Thermoplasm	Cuniculiplasma
ASV_2213	0	0	0	0	0	0	0.5	0.33	0	0	0	0	Bacteria	Chloroflexi	Chloroflexia	Thermomicro	JG30-KF-CM4	NA
ASV_2214	0.67	0	0	0	0	0	0	0	0	0	0	0	Bacteria	Planctomyce	Planctomyce	Planctomyce	Rubinisphaer	NA
ASV_2215	0	0	0.33	0	0.33	0.33	0	0	0	0	0	0	Bacteria	Chloroflexi	TK10	NA	NA	NA
ASV_2216	0	0	0	0	0	1	0.5	0	0	0	0	0	Bacteria	Proteobacter	Gammaprote	Burkholderia	Comamonad	Polaromonas
ASV_2217	0	0	0	0	0	1	1.5	0	0	0	0	0	Bacteria	Actinobacter	Actinobacter	Corynebacte	Corynebacte	Corynebacterium
ASV_2218	0	0	0	0	0	0	0	0	0	0	0	0.67	Bacteria	Proteobacter	Gammaprote	Burkholderia	Comamonad	NA
ASV_2219	1.33	0	0	0	0	0	0	0	1	0	0	0	Archaea	Crenarchaeo	Thermoprote	Sulfolobales	Sulfolobacea	Stygiolobus
ASV_2220	1.33	0	0.33	0	0	0	0	0	0	0	0	0	Archaea	Crenarchaeo	Thermoprote	Sulfolobales	Sulfolobacea	Stygiolobus
ASV_2221	0	0	0	0	0	0	0	0	0	0	0	0	Bacteria	Actinobacter	Acidimicrobii	NA	NA	NA
ASV_2222	0	0	0	0	2	0	0	0	0	0	0	0	Bacteria	Proteobacter	Alphaproteol	Sphingomona	Sphingomona	Sphingomonas
ASV_2223	0	0	0	0	0	0	0	0	0	0	0	0	Bacteria	NA	NA	NA	NA	NA
ASV_2224	0	0	0	0	0	0	0	0	0	0	0	3	Bacteria	Firmicutes	Negativicutes	Veillonellales	NA	NA
ASV_2225	0	0	0	0	0	0	0	0	0	0	0	0	Archaea	Crenarchaeo	Thermoprote	Sulfolobales	Sulfolobacea	Stygiolobus
ASV_2226	0	0	0	0	0.33	2.33	0	0	0	0	0	0	Bacteria	Firmicutes	Sulfobacillia	Sulfobacillale	Sulfobacillac	Sulfobacillus
ASV_2227	0	0	0	0	0	0	0.5	0	0	0	0	0	Bacteria	Actinobacter	Acidimicrobii	NA	NA	NA
ASV_2228	0	0	0	0	0	0	0	0	0	0	2.67	0	Bacteria	Actinobacter	Actinobacter	Pseudonocar	Pseudonocar	Pseudonocardia
ASV_2229	0	0	0	0	0	0	0	0	0	0	0	0	Bacteria	Firmicutes	Bacilli	Lactobacillale	Lactobacillac	Lactobacillus
ASV_2230	0	0	0	0	0	0	0	0	0	0	0	0	Bacteria	Firmicutes	Bacilli	Thermicanale	Thermicanac	Thermicanus
ASV_2231	0.67	0	0	0	0	0	0	0	0	0	0	0.67	Bacteria	Actinobacter	Thermoleoph	Solirubrobact	Solirubrobact	Conexibacter
ASV_2232	0.33	0	0	0	0	0.67	0	0.33	0	0	0	0	Bacteria	NA	NA	NA	NA	NA
ASV_2233	0.33	0	0.67	0	0	0	1	0	0	0	0	0	Bacteria	Proteobacter	Gammaprote	Legionellales	Legionellace	Legionella
ASV_2234	0	0	0.33	0	0	0.33	0	0	0	0	0	0	Bacteria	Actinobacter	Acidimicrobii	Microtrichale	Ilumatobacte	NA
ASV_2235	0	0	0.33	0.33	0	0	0	0.67	0	0	0	0	Bacteria	Chloroflexi	TK10	NA	NA	NA
ASV_2236	0.33	0.67	0	0	0	0	0.5	0	0	0	0	0	Bacteria	Actinobacter	Acidimicrobii	Actinomarina	NA	NA
ASV_2237	0	0	0	0	0	0	0.5	0	0	0	0	0	Bacteria	Myxococcota	Myxococcia	Myxococcale	Anaeromyxol	Anaeromyxobacter
ASV_2238	0	0	1.67	0	0	0	0	0	0	0	0	0	Bacteria	Bacteroidota	Bacteroidia	Cytophagales	Spirosomace	Spirosoma
ASV_2239	0.67	0	0	0	0	0.33	0	0	0	0	0	0	Bacteria	Proteobacter	Gammaprote	Pseudomona	Pseudomona	Pseudomonas
ASV_2240	0	0	0	0	0	0	0	0.67	0	0	0	1	Bacteria	Firmicutes	Bacilli	Bacillales	Bacillaceae	Aeribacillus
ASV_2241	0	0	0.67	0	0	0	0	0	0	0	0	0	Bacteria	Planctomyce	Planctomyce	Planctomyce	Rubinisphaer	SH-PL14
ASV_2242	0	0	0	0	0	0	1	0	0	0	0	0.33	Bacteria	Proteobacter	Gammaprote	Burkholderia	Oxalobactera	NA
ASV_2243	0	0	0	0	0	0	0	0	0	0	0	0.33	Bacteria	Patescibacte	Saccharimon	Saccharimon	NA	NA
ASV_2244	0	0	0	0	0	2	0	0	0	0	0	0	Bacteria	Firmicutes	Sulfobacillia	Sulfobacillale	Sulfobacillac	Sulfobacillus
ASV_2245	0	0	0	0	0	0	0	0	0	0	2.67	0.67	Bacteria	Proteobacter	Gammaprote	Enterobacter	Yersiniaceae	NA
ASV_2246	0	0	0	0	0	0	0	0	0	0	0	0	Bacteria	Patescibacte	Saccharimon	Saccharimon	Saccharimon	TM7a
ASV_2247	0.33	0	0	0	0	0	0	0	0	0	0	0	Bacteria	Patescibacte	Saccharimon	Saccharimon	NA	NA
ASV_2248	0.33	0	0.33	0	0	0	0	0.67	0	0	0	0	Bacteria	Gemmatimor	S0134 terrest	NA	NA	NA
ASV_2249	0	1	0	0	0	0	0	0	0	0	0	0	Bacteria	Firmicutes	Bacilli	Staphylococc	Staphylococc	Staphylococcus
ASV_2250	0	0	0	0.33	0	0	0.5	0	0	0	0	0	Bacteria	Myxococcota	Polyangia	Haliangiales	Haliangiaceae	Haliangium
ASV_2251	0	0	3.33	0	0	0	0	0	0	0	0	0	Bacteria	Bacteroidota	Bacteroidia	Chitinophaga	Chitinophaga	Sediminibacterium
ASV_2252	0	0	0.33	0	0	0	0	0	0	0	0	0	Bacteria	Verrucomicro	Verrucomicro	Verrucomicro	Verrucomicro	NA
ASV_2253	0	0	0	0	0	0	0	0	0	0	0	0	Bacteria	Proteobacter	Alphaproteol	Rhizobiales	Methylopilac	Hansschlegelia
ASV_2254	0	0	0	0	0	0	0	0	0	0	0	0	Bacteria	Actinobacter	Thermoleoph	Solirubrobact	NA	NA
ASV_2255	0	0	0	0	0	0.33	0	0.33	0	0	0	0	Bacteria	Chloroflexi	Dehalococco	S085	NA	NA
ASV_2256	0	0	0	0	0	0.67	0	0	0	0	0	0	Bacteria	Proteobacter	Alphaproteol	Acetobactera	Acetobactera	Acidiphilium
ASV_2257	0	0	0	0	0	0	0	0	0	0	0	0	Bacteria	Actinobacter	Thermoleoph	Solirubrobact	67-14	NA
ASV_2258	0.33	0	0	0	0	0.67	0.5	0	0	0	0	0	Bacteria	Gemmatimor	Gemmatimor	Gemmatimor	Gemmatimor	Gemmatimonas
ASV_2259	0	0	0	0	0	0.67	0.5	0.33	0	0	0	0	Bacteria	Chloroflexi	KD4-96	NA	NA	NA
ASV_2260	0	0	0	0	0	0	0	0	0	0	2.67	0	Bacteria	Actinobacter	Acidimicrobii	Microtrichale	Ilumatobacte	CL500-29 marine group
ASV_2261	0	0	0	1	0	0	0	0	0	0	0	0.33	Bacteria	Actinobacter	Acidimicrobii	NA	NA	NA
ASV_2262	0	0	0	0	0	0	0	0	3	0	0	0	Bacteria	Actinobacter	Thermoleoph	Solirubrobact	67-14	NA
ASV_2263	0	0	0	0	0	0	0	0	0	0	0	0.33	Bacteria	Proteobacter	Gammaprote	Burkholderia	Comamonad	NA
ASV_2264	1.33	1.67	0	0	0	0	0	0	0	0	0	0	Bacteria	Firmicutes	Bacilli	Staphylococc	Staphylococc	Staphylococcus
ASV_2265	0	1	0	0	0	0	0	0	2	0	1	0	Bacteria	Actinobacter	Rubrobacteri	Rubrobacter	Rubrobacteri	Rubrobacter
ASV_2266	0	0.33	0	0.67	0.33	0	0	0	0	0	0	0	Bacteria	Actinobacter	Acidimicrobii	NA	NA	NA
ASV_2267	0	0	0	0	0	0	0	3.33	0	0	0	0	Bacteria	Proteobacter	Alphaproteol	NA	NA	NA
ASV_2268	0	0	0	0	0	0	0	0	0	0	2	0	Bacteria	Planctomyce	Planctomyce	Gemmatales	Gemmatacea	NA
ASV_2269	0	0	0	0	0	0	0	0	0	0	0	0	Bacteria	Firmicutes	Sulfobacillia	Sulfobacillale	Sulfobacillac	NA
ASV_2270	0	0	0	0	0	0	0	0	0	0	0	0	Bacteria	Proteobacter	Alphaproteol	Acetobactera	Acetobactera	Acidiphilium
ASV_2271	0.33	0	0	0.33	0	0	0	0	0	0	0	0	Bacteria	Bdellovibrion	Oligoflexia	0319-6G20	NA	NA
ASV_2272	0	0	0	0	0	0	2	0	0	0	0	0	Bacteria	Acidobacteri	Holophagae	Subgroup 7	NA	NA
ASV_2273	0.33	0	0.33	0	0	0	1	0	0	0	0	0	Bacteria	Actinobacter	Thermoleoph	Solirubrobact	67-14	NA
ASV_2274	0.33	0	0	0	0	0	0	0	0	0	0	0.33	Bacteria	Bdellovibrion	Bdellovibrion	Bacteriovora	Bacteriovora	Peredibacter
ASV_2275	0	0	0.33	0	0	0.33	0	0	0	0	0	0	Bacteria	Actinobacter	Acidimicrobii	NA	NA	NA
ASV_2276	0.33	0.33	0.33	0	0	0	0.5	0	0	0	0	0	Bacteria	Actinobacter	Acidimicrobii	NA	NA	NA
ASV_2277	0	0	0	0	0	0.33	0.5	0.33	0	0	0	0	Bacteria	Bacteroidota	Bacteroidia	Chitinophaga	NA	NA
ASV_2278	0.33	0	0	0	0	0.67	0	0.33	0	0	0	0	Bacteria	Planctomyce	Planctomyce	Gemmatales	Gemmatacea	Zavarzinella
ASV_2279	0	0	0.67	0	0	0	0	0	0	0	0	0	Bacteria	Chloroflexi	Chloroflexia	Thermomicro	JG30-KF-CM4	NA
ASV_2280	0.33	0	0	0.33	0	0	0	0	0	0	0	0	Bacteria	Verrucomicro	Verrucomicro	Chthoniobact	Terrimicrobi	Terrimicrobium
ASV_2281	0	0	0	0.33	0	0	0	0	0	0	0	0	Bacteria	Firmicutes	Bacilli	Staphylococc	Staphylococc	Staphylococcus
ASV_2282	0	0	0	0.33	0	0	0	0	0	0	0	0	Bacteria	Actinobacter	MB-A2-108	NA	NA	NA
ASV_2283	0	0	0	0.33	0	0	1	0	0	0	0	0	Bacteria	Proteobacter	Gammaprote	Legionellales	Legionellace	Legionella
ASV_2284	0	0	0.33	0	0	0	1	0	0	0	0	0	Bacteria	Actinobacter	Thermoleoph	Solirubrobact	Solirubrobact	Solirubrobacter
ASV_2285	2.33	0	0	0	0	0	0	0	0	0	0	0	Archaea	Crenarchaeo	Thermoprote	Sulfolobales	Sulfolobacea	Stygiolobus
ASV_2286	0.33	0	0	0	0.33	0	0	0.33	0	0	0	0	Bacteria	Actinobacter	Thermoleoph	NA	NA	NA
ASV_2287	0	0	0	0	0	1	1.5	0	0	0	0	0	Bacteria	Bacteroidota	Bacteroidia	Chitinophaga	Chitinophaga	Flavisolibacter
ASV_2288	0	0	0	0	0.33	0	0.5	0	0	0	0	0	Bacteria	Chloroflexi	TK10	NA	NA	NA
ASV_2289	0	0	0	0.67	0	0	0	0	0	0	0	0	Bacteria	Proteobacter	Gammaprote	Pseudomona	Moraxellacea	Acinetobacter

ASV_2290	0	0	0	0	1.33	0	0	0	0	0	0	0	Archaea	Thermoplasn	Thermoplasn	Thermoplasn	Thermoplasn	A-plasma
ASV_2291	0	0	0	0	0	0	0	0	0	0	2	0	Bacteria	Actinobacter	Actinobacter	Corynebacter	Corynebacter	Turicella
ASV_2292	0	0	0	0	0	1.67	0	0	0	0	0	0	Bacteria	Chloroflexi	AD3	NA	NA	NA
ASV_2293	0	0	0	0	0	0	0	3	0	0	0	0	Bacteria	Proteobacter	Alphaproteol	Sphingomona	Sphingomona	NA
ASV_2294	0	0	0	0	0	0	0	0	0	0	0	0	Bacteria	Chloroflexi	Chloroflexia	Kallotenuales	AKIW781	NA
ASV_2295	0	0	0	0	0	0	0	0	0	0	0	0	Bacteria	Firmicutes	Sulfobacillia	Sulfobacillale	Sulfobacillace	Sulfobacillus
ASV_2296	0.67	0	0	0.33	0	0	0	0	0	0	0	0	Bacteria	Patescibacter	ABY1	Candidatus K	NA	NA
ASV_2297	0	0	0	0	0	0	0	0	0	0	0	0.33	Bacteria	Proteobacter	Gammaprote	Burkholderia	Hydrogenoph	Thiobacillus
ASV_2298	0.33	0	0	0	0	0	0	0	0	0	0	0.33	Bacteria	Proteobacter	Alphaproteol	NA	NA	NA
ASV_2299	0.33	0	0	0.33	0	0.33	0	0	0	0	0	0	Bacteria	Bacteroidota	Bacteroidia	Chitinophaga	Chitinophaga	Terrimonas
ASV_2300	0	0	0	0	0	0	0	0	0	0	0	2	Bacteria	Actinobacter	Actinobacter	Corynebacter	Corynebacter	NA
ASV_2301	0	0	0	0.33	0	0	0	0	0	0	0	0	Bacteria	Bdellovibrion	Bdellovibrion	Bacteriovora	Bacteriovora	Peredibacter
ASV_2302	0.33	0	0	0.67	0	0	0	0	0	0	0	0	Bacteria	Cyanobacteri	Cyanobacteri	Cyanobacteri	Chroococcidi	NA
ASV_2303	0	0	0	0	0	0	0	0	0	0	0	1	Bacteria	Firmicutes	Bacilli	Staphylococc	Staphylococc	NA
ASV_2304	0.67	0	0	0	0	0	0	0	0	0	0	0	Bacteria	Proteobacter	Alphaproteol	Sphingomona	Sphingomona	Hephaestia
ASV_2305	0	0	0	0	0	0.33	1	0.67	0	0	0	0	Bacteria	Armatimonac	Armatimonac	Armatimonac	NA	NA
ASV_2306	0	0	0	0	0	0	0	0	0	0	2.33	0	Bacteria	NA	NA	NA	NA	NA
ASV_2307	0	0	0.33	0	0	0	0	0	0	0	0	0	Bacteria	Proteobacter	NA	NA	NA	NA
ASV_2308	0	0	0	0	0	0	3	0	0	0	0	0	Bacteria	Actinobacter	Actinobacter	Corynebacter	Dietziaceae	Dietzia
ASV_2309	0	0	0	0	0	0	0	0	0	0	0	0	Bacteria	Planctomycet	Planctomycet	Isosphaerales	Isosphaerace	NA
ASV_2310	0	0	0	0	0	0	0.5	0	0	0	0	0	Bacteria	Firmicutes	Bacilli	Staphylococc	Staphylococc	NA
ASV_2311	0	0	0	0	0	0.67	0	0	0	0	0	0	Bacteria	Actinobacter	Acidimicrobii	NA	NA	NA
ASV_2312	0	0	0	0.33	0	0	0	0.33	0	0	0	0	Bacteria	Actinobacter	Acidimicrobii	IMCC26256	NA	NA
ASV_2313	0.33	0	0	0	0	0	0	0	0	0	0	0	Bacteria	Actinobacter	Acidimicrobii	NA	NA	NA
ASV_2314	0	0	0	0.67	0	0	0	0	0	0	0	0	Bacteria	Proteobacter	Alphaproteol	Caulobactera	Caulobactera	Brevundimonas
ASV_2315	0.67	0	0	0	0	0	0	0	0	0	0	0	Archaea	Crenarchaeo	Thermoprote	Sulfolobales	Sulfolobacea	Stygiolobus
ASV_2317	0	1.33	0	0	0	0	0	0	0	0	0	0	Bacteria	Proteobacter	Alphaproteol	Rhizobiales	Rhizobiaceae	Pseudaminobacter
ASV_2318	0	0.33	0	0.33	0	0	0	0	0	0	0.33	0	Bacteria	Proteobacter	Gammaprote	Burkholderia	Comamonada	NA
ASV_2319	0	0	0	0	1	0	0.5	0	0	0	0	0	Bacteria	Actinobacter	Thermoleoph	Gaiellales	NA	NA
ASV_2320	0	0	0	0	0	0.33	1.5	0	0	0	0	0	Bacteria	Actinobacter	Actinobacter	Bifidobacteri	Bifidobacteri	Bifidobacterium
ASV_2321	0	0	2.67	0	0	0	0	0	0	0	0	0	Bacteria	Acidobacteri	Acidobacteria	Subgroup 13	NA	NA
ASV_2322	0	0	0	0	0.33	0	0	0	0	0	0	0	Bacteria	Sumerlaeota	Sumerlaeia	Sumerlaeales	Sumerlaeace	Sumerlaea
ASV_2323	0	0	0	0	0	0	0	0	0	0	2.67	0	Bacteria	Gemmatimor	Longimicrobi	Longimicrobi	Longimicrobi	NA
ASV_2325	0	0	0	0	2.33	0	0	0	3	0	0	0	Bacteria	Gemmatimor	S0134 terrest	NA	NA	NA
ASV_2326	0	0	0	0	1.33	0	0	0	0	0	0	0	Bacteria	Desulfobacte	Desulfuromo	Desulfuromo	NA	NA
ASV_2327	0	0	0	0	0	0	0	2.67	0	0	0	0	Bacteria	Proteobacter	Gammaprote	Nitrosococca	Nitrosococca	SZB85
ASV_2328	0	0	0	0	0	0	0	0	0	0	0	0.33	Bacteria	Proteobacter	Gammaprote	Pseudomona	Halomonada	Halomonas
ASV_2329	0.33	0	0	0	0	0	0	0	0	0	0	0	Bacteria	Proteobacter	Gammaprote	Burkholderia	Comamonada	Acidovorax
ASV_2330	0	0	0.67	0	0	0	0	0	0	0	0	0	Bacteria	Actinobacter	Acidimicrobii	NA	NA	NA
ASV_2331	0.33	0	0	0	0	0	0.5	0	0	0	0	0	Bacteria	Patescibacter	Saccharimon	Saccharimon	LWQ8	NA
ASV_2332	0	0	0	0	0	0	0.5	0	0	0	0	0	Bacteria	Proteobacter	Alphaproteol	Sphingomona	Sphingomona	Sandaracinobacter
ASV_2333	0	0	0	0	0	1	0	0	0	0	0	0	Bacteria	Patescibacter	Saccharimon	Saccharimon	NA	NA
ASV_2334	0	0	0	0	0.33	0	0	0	0	0	0	0	Bacteria	Actinobacter	Acidimicrobii	NA	NA	NA
ASV_2335	0.33	0	0	0	0	0	0	0	0	0	0	0	Bacteria	Actinobacter	Thermoleoph	Gaiellales	NA	NA
ASV_2336	0	0	0	0	0	0	0	0	0	0	0	0	Bacteria	Chloroflexi	NA	NA	NA	NA
ASV_2337	0	0	0	0	0	0	0	0	0	0	0	0	Bacteria	NA	NA	NA	NA	NA
ASV_2338	0	0	0	0	0	0	1	0	0	0	0	0	Bacteria	Armatimonac	Armatimonac	Armatimonac	NA	NA
ASV_2339	0	0	0	0	0	0	0	0	0	0	0	0	Bacteria	Bdellovibrion	Bdellovibrion	Bacteriovora	Bacteriovora	Peredibacter
ASV_2340	0	0	0	0	0	0	1	0	0	0	0	0	Bacteria	Bdellovibrion	Bdellovibrion	Bacteriovora	Bacteriovora	Peredibacter
ASV_2341	0	0	0.67	0	0	0	0	0	0	0	0	0	Bacteria	Chloroflexi	TK10	NA	NA	NA
ASV_2342	0	0	0	0	0	0	0	0	0	0	0	0	Bacteria	Firmicutes	Bacilli	Thermicanale	Thermicanac	Thermicanus
ASV_2343	0	0	0	0	0	0.67	0	0.33	0	0	0	0	Bacteria	Patescibacter	NA	NA	NA	NA
ASV_2344	0	0	0	0	0	0	1	0	0	0	0	0	Bacteria	Bacteroidota	Bacteroidia	Bacteroidales	Bacteroidete	NA
ASV_2345	0.33	0	0	0	0	0.33	1	0	0	0	0	0	Bacteria	Deinococcota	Deinococci	Deinococcale	Trueperacea	Truepera
ASV_2346	0	0	0	0	0	0.67	0.5	0	0	0	0	0	Bacteria	Proteobacter	Alphaproteol	Rhizobiales	Xanthobacter	NA
ASV_2347	0	0	0	0.33	0	0	0	0	0	0	0	0	Bacteria	Actinobacter	Actinobacter	Pseudonocar	Pseudonocar	NA
ASV_2348	0	0	0	0	0.33	0	0	0.33	0	0	0	0	Bacteria	Planctomycet	Phycisphaera	Tepidisphaer	WD2101 soil	NA
ASV_2349	0	0	0	0	0	0.33	0.5	0	0	0	0	0.67	Bacteria	Actinobacter	Actinobacter	0319-7L14	NA	NA
ASV_2350	0	0	0	0	0	0	0	0	0	0	0	0	Bacteria	Proteobacter	Alphaproteol	Caulobactera	Parvularculac	Amphiplicatus
ASV_2351	0	0	0	0	0.67	0	1	0	0	0	0	0	Bacteria	Abditibacteri	Abditibacteri	Abditibacteri	Abditibacteri	Abditibacterium
ASV_2352	0	0	0	0	1.33	0	0	0	0	0	0	0	Bacteria	Actinobacter	Actinobacter	Nitriliruptora	Nitriliruptora	NA
ASV_2353	0	0	0	0.67	0	0	0	0	0	0	0	0	Bacteria	Proteobacter	Alphaproteol	Rhizobiales	Rhizobiales Ir	NA
ASV_2354	0.33	0	0	0.33	0	0	0	0	0	0	0	0	Bacteria	Myxococcota	Polyangia	Blfdi19	NA	NA
ASV_2355	0.33	0	0	0	0	0	0.5	0	0	2	0	0	Bacteria	Actinobacter	Acidimicrobii	Microtrichale	Ilumatobacte	NA
ASV_2356	0	0	0	0	0	0.33	0.5	0	0	0	0	0	Bacteria	Actinobacter	Actinobacter	Euzebyales	Euzebyaceae	Euzebya
ASV_2357	0	0	0	0.67	0	0	0.5	0.67	0	0	0	0	Bacteria	Myxococcota	Polyangia	Polyangiales	Polyangiacea	Jahnella
ASV_2358	0	0	0	0.33	0	0	0	0.33	0	0	0	0	Bacteria	Patescibacter	Saccharimon	Saccharimon	NA	NA
ASV_2359	0.33	0	0.33	0	0	0	0	0	0	0	0	0	Bacteria	Bdellovibrion	Oligoflexia	0319-6G20	NA	NA
ASV_2360	0.33	0	0	0.33	0	0	0	0	0	0	0	0	Bacteria	Verrucomicro	Verrucomicro	Chthoniobact	Chthoniobact	Candidatus Udaeobacter
ASV_2361	0	0	0	1.67	0	0	0	0	0	0	0	0	Bacteria	Actinobacter	Actinobacter	Corynebacter	Mycobacteria	Mycobacterium
ASV_2362	0.33	0	0	0.33	0	0	0	0	0	0	0	0	Bacteria	Chloroflexi	Chloroflexia	Thermomicro	JG30-KF-CM4	NA
ASV_2363	0	0	0	0	0	0	0	0	0	0	0	0	Bacteria	Actinobacter	Actinobacter	Propionibact	Nocardioidac	Nocardioides
ASV_2364	0	0	0.33	0	0	0	1	0	0	0	0	0	Bacteria	Chloroflexi	JG30-KF-CM6	NA	NA	NA
ASV_2365	0	0	0	0	0	0	0	0	0	0	0	0	Bacteria	Verrucomicro	Verrucomicro	Chthoniobact	Chthoniobact	Chthoniobacter
ASV_2366	0	0	0	0	0	0	0	0	0	0	0	0	Bacteria	Firmicutes	Bacilli	Lactobacillale	Lactobacillace	Latilactobacillus
ASV_2367	0	0	0	0.33	0	0	0	0	0	0	0.67	0	Bacteria	Actinobacter	Actinobacter	0319-7L14	NA	NA
ASV_2368	0	0	0	0	0	0	0.5	1	0	0	0	0	Bacteria	Planctomycet	Phycisphaera	Tepidisphaer	WD2101 soil	NA
ASV_2370	0	0	0	0	0	0	0	0	0	0	0	0	Bacteria	Proteobacter	Alphaproteol	Sphingomona	Sphingomona	Sphingomonas
ASV_2371	0	0	0	0	0	0.67	0	0	0	0	0	0.33	Bacteria	Bacteroidota	Bacteroidia	Chitinophaga	NA	NA
ASV_2372	0	0	0	0	0	0	0	0	0	0	0	0	Bacteria	Actinobacter	Actinobacter	Propionibact	Nocardioidac	Nocardioides
ASV_2373	0	0	0	0	0	0.33	1.5	0	0	0	0	0	Bacteria	Planctomycet	Planctomycet	Gemmatales	Gemmatacea	NA
ASV_2374	0	0	0	0	0	0	0	0	0	0	2.67	0	Bacteria	Chloroflexi	Chloroflexia	Kallotenuales	AKIW781	NA
ASV_2375	0	0	0	0	0	0	0	0	0	0	0	0	Bacteria	Firmicutes	Sulfobacillia	Sulfobacillale	Sulfobacillace	NA
ASV_2376	0	0	0	0	0	0	0	0	0	0	0	0	Bacteria	Actinobacter	Thermoleoph	Gaiellales	NA	NA
ASV_2377	0	0	0	0	0	0	0	0	0	0	0	0	Bacteria	Firmicutes	Sulfobacillia	Sulfobacillale	Sulfobacillace	Sulfobacillus
ASV_2378	0	0	0	0	0	0	0	0	0	0	0	0	Bacteria	Chloroflexi	Ktedonobact	Ktedonobact	Ktedonobact	Thermogemmatispora
ASV_2379	0	0	0	0	0	0	0	0	0	0	0	0	Archaea	Crenarchaeo	Thermoprote	Sulfolobales	Sulfolobacea	Stygiolobus
ASV_2380	0	0	0.33	0	0	0	0	0	0	0	0	0	Bacteria	Actinobacter	Thermoleoph	Solirubrobact	67-14	NA
ASV_2381	0	0	0	0.67	0	0	0	0.33	0	0	0	0	Bacteria	Actinobacter	Acidimicrobii	NA	NA	NA
ASV_2382	0	0	0	0	0	0	0	0	0	0	0	0	Bacteria	Actinobacter	Thermoleoph	Solirubrobact	67-14	NA
ASV_2383	0	0	0	0	0.33	0	0	0	0	0	0	0	Bacteria	Chloroflexi	KD4-96	NA	NA	NA
ASV_2384	0	0	0	0	0	0	0	0	0	0	0	0.67	Bacteria	Actinobacter	Actinobacter	Frankiales	NA	NA
ASV_2385	0	0	0.33	0	0	0	0	0	0	0	0	0	Bacteria	NA	NA	NA	NA	NA
ASV_2386	0	0	0	0	0	0	0	0	0	0	0	0	Bacteria	Chloroflexi	JG30-KF-CM6	NA	NA	NA
ASV_2387	0	0	0	0	0	0	0.5	0	0	0	0	0	Bacteria	Chloroflexi	OLB14	NA	NA	NA
ASV_2388	0	0	0.33	0	0	0	0	0	0	0	0	0	Bacteria	Abditibacteri	Abditibacteri	Abditibacteri	Abditibacteri	Abditibacterium
ASV_2389	0.67	0	0	0	0	0	0	0	0	0	0	0	Bacteria	Proteobacter	Alphaproteol	Sphingomona	Sphingomona	NA
ASV_2390	0	0.67	0	0	0	0	0	0.33	0	0	0.33	0	Bacteria	Chloroflexi	Chloroflexia	Kallotenuales	AKIW781	NA
ASV_2391	0	0	0	0	0	0	0	0	0	0	0	0	Bacteria	Proteobacter	Alphaproteol	Rhizobiales	Beijerinckiace	NA
ASV_2392	0	0	0	0	0	0	0	0	0	0	0	0	Bacteria	Actinobacter	Thermoleoph	Gaiellales	NA	NA
ASV_2393	0	0	0	0	0	0.67	0.5	0	0	0	0	0.67	Bacteria	Actinobacter	Actinobacter	Corynebacter	NA	NA
ASV_2395	0	0	0.67	0	0	0	0	0	0	0	0	0	Archaea	Crenarchaeo	Thermoprote	Sulfolobales	Sulfolobacea	NA
ASV_2396	0	0	0	0	0.67	0	0	0	0	0	0	0	Bacteria	NA	NA	NA	NA	NA
ASV_2397	0	0	0	0	0.67	0	0.5	0.33	0	0	0	0	Bacteria	Actinobacter	Actinobacter	Frankiales	NA	NA
ASV_2398	0	0	0	0	0	0	4.5	0	0	0	0	0	Bacteria	Acidobacteri	Blastocatellia	Blastocatellal	Blastocatellac	NA
ASV_2399	0	0	0	0	0	0.33	1.5	0	0	0	0	0	Bacteria	NA	NA	NA	NA	NA
ASV_2400	0	0	0	0	0	0	3.5	0	0	0	0	0	Bacteria	Proteobacter	Gammaprote	Burkholderia	NA	NA
ASV_2401	0	0	0	0	0	0	0	0	0	0	0	0	Bacteria	Chloroflexi	Chloroflexia	Thermomicro	JG30-KF-CM4	NA
ASV_2402	0	0	0	0	0	0	0	0	0	0	0	1.67	Bacteria	Actinobacter	Actinobacter	Propionibact	Propionibact	NA
ASV_2403	0	0	0	0	0	0	0	0	0	0	0	0	Bacteria	Firmicutes	Negativicutes	Veillonellales	Veillonellacea	Dialister
ASV_2404	0.33	0	0	0.33	0	0	0	0	0	0	0	0	Bacteria	NA	NA	NA	NA	NA
ASV_2405	0	0	0	0	0	0	0	0	0	0	0	0	Bacteria	Bdellovibrion	Bdellovibrion	Bacteriovora	Bacteriovora	Peredibacter
ASV_2406	0	0	0	0	0	0	0	0	0	0	0	0	Bacteria	Verrucomicro	Verrucomicro	Chthoniobact	Xiphinematol	Candidatus Xiphinematobacter
ASV_2407	0	0.33	0	0	0	0	0	0	0	0	0	0	Bacteria	Chloroflexi	TK10	NA	NA	NA
ASV_2408	1	0	0	0	0	0	0	0	0	0	0	0	Archaea	Crenarchaeo	Thermoprote	Sulfolobales	Sulfolobacea	Stygiolobus
ASV_2409	0	0	0.33	0	0	0	1	0	0	0	0	0	Bacteria	Patescibacter	Saccharimon	Saccharimon	NA	NA
ASV_2410	0	0	0	0	0	0	0	0.33	0	0	0	0	Bacteria	Actinobacter	Thermoleoph	Gaiellales	NA	NA
ASV_2411	0	0	0	0	0.33	0	0	0	0	0	0	0	Bacteria	Proteobacter	Gammaprote	Diplorickettsi	Diplorickettsi	NA
ASV_2412	0	0	0	0	0	0.33	0	0.33	0	0	0	0	Bacteria	Acidobacteri	Blastocatellia	Pyrinomonad	Pyrinomonad	RB41
ASV_2413	0	0	0	0	0	0	0.5	0	0	0	0	0	Bacteria	Actinobacter	Actinobacter	Propionibact	Nocardioidac	NA
ASV_2414	1	0	0	0	0	0	0	0	0	0	0	0	Bacteria	Proteobacter	Gammaprote	Pseudomona	Cellvibrionac	Cellvibrio
ASV_2415	0	0	0	0	0	0	0	0	0	0	0	0	Archaea	Crenarchaeo	Thermoprote	Sulfolobales	Sulfolobacea	Stygiolobus
ASV_2416	0	0.67	0	0	0	0	0	0	0	0	0	0	Bacteria	Latescibacter	Latescibacter	Latescibacter	Latescibacter	NA
ASV_2417	0	0	0	0	0	0.33	0.5	0.33	0	0	0.33	0	Bacteria	Patescibacter	NA	NA	NA	NA
ASV_2418	0	0	0	0	0	0	0	0	0	0	0	0	Bacteria	Actinobacter	Coriobacterii	OPB41	NA	NA
ASV_2419	0	0	0	0	0	0	1.5	0	0	0	0	0.33	Bacteria	Cyanobacteri	Sericytochro	NA	NA	NA
ASV_2420	0	0	0	0	0	0	0	0	0	0	0	0	Bacteria	Firmicutes	YNPFFP2	NA	NA	NA
ASV_2421	0	0	0.67	0	0	0	0	0	0	0	0	0	Bacteria	Planctomycet	Phycisphaera	mle1-8	NA	NA
ASV_2422	0	0	0	0	0.33	0.67	0	0	0	0	0	0	Bacteria	Firmicutes	Sulfobacillia	Sulfobacillale	Sulfobacillace	Sulfobacillus
ASV_2423	0	0	0	0	0	3	0	0	0	0	0	0	Bacteria	Firmicutes	Sulfobacillia	Sulfobacillale	Sulfobacillace	NA
ASV_2424	0	0	0	0	0	0	3	0	0	0	0	0	Bacteria	Firmicutes	Bacilli	Paenibacillale	Paenibacillac	Paenibacillus
ASV_2425	0.67	0	0.33	0	0	0	1	0	0	0	0	0.67	Bacteria	Patescibacter	Gracilibacteri	NA	NA	NA
ASV_2426	0	0	0	0	0	0	0	0	0	0	0	0	Bacteria	Actinobacter	Acidimicrobii	Microtrichale	Ilumatobacte	NA
ASV_2427	0	0	0	0.33	0	0	0	0	0	0	0	0	Bacteria	Verrucomicro	Verrucomicro	Opitutales	Opitutaceae	Opitutus
ASV_2428	0	0	0	0	0	0.33	0	0	0	0	0	0	Bacteria	Gemmatimor	Gemmatimor	Gemmatimor	Gemmatimor	NA
ASV_2429	0	0	0.33	0	0	0	0	0	0	0	0	0	Bacteria	Cyanobacteri	Cyanobacteri	Leptolyngbya	Leptolyngbya	NA
ASV_2430	0	0	0.33	0	0	0	0	0.33	0	0	0	0	Bacteria	Actinobacter	Thermoleoph	Gaiellales	NA	NA
ASV_2431	0	0	0	0	0	0	0.5	0	0	0	0	0	Bacteria	Proteobacter	Alphaproteol	Holosporales	Holosporacea	NA
ASV_2432	0	0	0	0.33	0	0	0	0	0	0	0	0	Bacteria	Desulfobacte	NA	NA	NA	NA
ASV_2433	0	0	0	0	0	0	0.5	0.33	0	0	0	0	Bacteria	Patescibacter	Saccharimon	Saccharimon	LWQ8	NA
ASV_2434	0	0	0	0	0	0.33	0.5	0	0	0	0	0	Bacteria	Planctomycet	Phycisphaera	Tepidisphaer	WD2101 soil	NA
ASV_2435	0	0.33	0	0	0	0	1	0	0	0	0	0	Bacteria	Actinobacter	Actinobacter	Frankiales	Sporichthyac	hgcI clade
ASV_2436	0	0	1.33	0	0	0	0	0	0	0	0	0	Bacteria	Patescibacter	Gracilibacteri	JGI 0000069-	NA	NA
ASV_2437	0	0.33	0	0	0	0	0	0	0	0	0	0	Bacteria	Actinobacter	Actinobacter	NA	NA	NA
ASV_2438	0	0	0	0	0	0	0	0	0	0	0	0	Bacteria	Acidobacteri	Blastocatellia	Pyrinomonad	Pyrinomonad	RB41
ASV_2439	0	0	0	0	0	0	0	0.33	0	0	0	0	Bacteria	Actinobacter	Acidimicrobii	Microtrichale	Iamiaceae	Iamia
ASV_2440	0	0	0.33	0	0	0	0	0	0	0	0	0	Bacteria	Planctomycet	Planctomycet	Isosphaerales	Isosphaerace	NA

ASV_2441	0	0	0	0	0.67	0	0	0.33	0	0	0	0	Bacteria	Actinobacter	Acidimicrobii	Microtrichale	Ilumatobacte	NA
ASV_2442	0	0	0.33	0	0	0	1	0	0	0	0	0	Bacteria	Armatimonac	Armatimonac	Armatimonac	NA	NA
ASV_2443	0	0	0	0	0	0	0	0	0	0	0	0	Bacteria	Planctomycet	Phycisphaera	Tepidisphaer	WD2101 soil	NA
ASV_2444	0	0	0	0	1	0	0.5	0	0	0	0	0.33	Bacteria	Chloroflexi	TK10	NA	NA	NA
ASV_2445	0	0	0	0	0	0	0.5	0.67	0	0	0	0	Bacteria	Bacteroidota	Bacteroidia	Chitinophaga	Chitinophaga	Flavisolibacter
ASV_2446	0	0	0	0	0.67	0	0	0	0	0	0	0	Bacteria	Firmicutes	Clostridia	Clostridiales	Clostridiacea	Clostridium sensu stricto 10
ASV_2447	0	0	0	0	1	0	0	0	0	0	0	0	Bacteria	Actinobacter	Actinobacter	NA	NA	NA
ASV_2448	0	0	0	0	0	0	0	0	0	0	0	0	Bacteria	Actinobacter	Actinobacter	Micromonos	Micromonos	Actinoplanes
ASV_2449	0	0	0	0	0	0	0	0	0	0	0	0	Bacteria	Actinobacter	Actinobacter	0319-7L14	NA	NA
ASV_2450	0	0	0	0	0	0	0	0	0	0	0	0	Bacteria	Chloroflexi	AD3	NA	NA	NA
ASV_2451	0.33	0	0	0	0	0	0	0	0	0	0	0	Bacteria	Chloroflexi	JG30-KF-CM6	NA	NA	NA
ASV_2452	0	0	0	0	0	0	1	0	0	0	0	0	Bacteria	Actinobacter	Thermoleoph	Gaiellales	NA	NA
ASV_2453	0	0	0	0	0	0	0	0	0	0	0	0	Bacteria	Bdellovibrion	Bdellovibrion	Bacteriovora	Bacteriovora	Peredibacter
ASV_2454	0	0.67	0	0	0	0	0	0.67	0	0	0	0	Bacteria	NA	NA	NA	NA	NA
ASV_2455	0.33	0	0	0	0	0	0	0	0	0	0	0	Bacteria	Myxococcota	Polyangia	Polyangiales	BIrii41	NA
ASV_2456	0.33	0	0	0	0	0	0	0	0	0	0	0	Bacteria	Chloroflexi	Anaerolineae	SBR1031	NA	NA
ASV_2457	0	0	0	0	0	0	0	0	0	0	0	1.67	Bacteria	Firmicutes	Bacilli	Lactobacillale	Aerococcacea	NA
ASV_2458	0	0	0	0	0	0	0	0.33	0	0	0.33	0	Bacteria	Actinobacter	Rubrobacteri	Rubrobactera	Rubrobacteri	Rubrobacter
ASV_2459	0	0	0	0.33	0	0	0	0	0	0	0	0	Bacteria	Firmicutes	Sulfobacillia	Sulfobacillale	Sulfobacillace	Sulfobacillus
ASV_2460	0	0	0	0	0	0	0	0	0	0	0	0	Bacteria	Acidobacteri	Blastocatellia	Pyrinomonac	Pyrinomonad	RB41
ASV_2461	1	0	0	0	0	0	1	0	0	0	0	0	Bacteria	Proteobacter	Alphaproteol	Reyranellales	Reyranellacea	Reyranella
ASV_2462	0	0	0	0	0.33	0	0.5	0.33	0	0	0	0	Archaea	Thermoplasm	Thermoplasm	Thermoplasm	Thermoplasm	A-plasma
ASV_2463	0	0	0	0	0.33	0	0	0	0	0	0	0.67	Bacteria	Proteobacter	Gammaprote	Pseudomona	Moraxellacea	Acinetobacter
ASV_2464	0	0	0	0	0	0	0	0	0	0	0	0	Bacteria	Planctomycet	Planctomycet	Gemmatales	Gemmatacea	Fimbriiglobus
ASV_2465	0	0	0	0	0	0.67	0.5	0.33	0	0	0	0	Bacteria	Bacteroidota	Bacteroidia	Sphingobacte	NA	NA
ASV_2466	0	0	1.67	0	0	0	0	0	0	0	0	0	Bacteria	Chloroflexi	Anaerolineae	Anaerolineale	Anaerolineac	NA
ASV_2467	0	0	0	0	0	0	1	0	0	0	0	0	Bacteria	Desulfobacte	Desulfobulbi	Desulfobulba	Desulfocapsa	[Desulfobacterium] catecholicum group
ASV_2468	0	0	0	0	2.33	0	0	0	0	0	0	0	Bacteria	Proteobacter	Alphaproteol	Rhizobiales	Beijerinckiace	Microvirga
ASV_2469	0	0	0	0	0	0.33	0	0	0	0	0	0	Bacteria	Cyanobacteri	Cyanobacteri	NA	NA	NA
ASV_2470	0	0	0	0	0	0	1	0	0	0	0	0	Bacteria	Bacteroidota	Bacteroidia	Sphingobacte	env.OPS 17	NA
ASV_2471	0	0	0	0	0	0	2.5	0	0	0	0	0	Bacteria	Proteobacter	Alphaproteol	Rhizobiales	Rhizobiaceae	Neorhizobium
ASV_2472	0	0	0	0	0	0	0	0	0	0	0	0	Archaea	Crenarchaeo	Nitrososphae	Nitrosotaleal	Nitrosotaleac	NA
ASV_2473	0	0	0.67	0	0	0	0	0	3	0	0	0	Bacteria	Actinobacter	Thermoleoph	Solirubrobact	Solirubrobact	Solirubrobacter
ASV_2474	0	0	0	0	0	0	0	0	0	0	0	0	Bacteria	Actinobacter	Acidimicrobii	Microtrichale	Ilumatobacte	NA
ASV_2475	0	0	0	0	0	0	0.5	0	0	0	0	0	Bacteria	Proteobacter	Alphaproteol	Rhizobiales	Xanthobacte	NA
ASV_2476	0	0	0	0	0	0	0	0	0	0	0	0	Bacteria	Myxococcota	Polyangia	Polyangiales	Phaselicystid	Phaselicystis
ASV_2477	0.33	0	0	0	0	0	0	0	0	0	0	0	Bacteria	NA	NA	NA	NA	NA
ASV_2478	0	0	0	0.33	0	0	0	0	0	0	0	0	Bacteria	Actinobacter	Actinobacter	0319-7L14	NA	NA
ASV_2479	0	0	0	0	0	0	0	0	0	0	0	0	Bacteria	Proteobacter	Alphaproteol	Sphingomona	Sphingomona	Sphingomonas
ASV_2481	0	0	0	0	0	0	0	0	0	0	0	0	Bacteria	Actinobacter	Thermoleoph	Gaiellales	NA	NA
ASV_2482	0	0	0	0	0	0	0	0	0	0	0	0	Bacteria	Firmicutes	Clostridia	Lachnospirale	Lachnospirac	NA
ASV_2483	0	0	0	0	0	0	0	0	0	0	0	0	Bacteria	Actinobacter	Actinobacter	Frankiales	NA	NA
ASV_2484	0	0	0.33	0	0	0	2	0	0	0	0	0	Bacteria	Proteobacter	Alphaproteol	Tistrellales	Geminicocca	Candidatus Alysiosphaera
ASV_2485	0	0	0.33	0	0	0	0	0	0	7	0	0	Bacteria	Proteobacter	Gammaprote	Burkholderia	Nitrosomona	NA
ASV_2486	0	0	0	0	0	0.67	0.5	0	0	0	0	0	Bacteria	Actinobacter	Actinobacter	Micromonos	Micromonos	Longispora
ASV_2487	0	0	0	0	0	0	2	0	0	0	0	0	Bacteria	Actinobacter	Acidimicrobii	NA	NA	NA
ASV_2488	0	0	0	0	0	0.67	0.5	0	4	0	0	0	Bacteria	Proteobacter	Gammaprote	Burkholderia	Neisseriaceae	Eikenella
ASV_2489	0	0	0	0	0	0.67	0	0	0	0	0	0	Bacteria	Actinobacter	Acidimicrobii	NA	NA	NA
ASV_2490	0	0	0	0	0	0	0	0	0	0	0	0	Bacteria	Actinobacter	Thermoleoph	Solirubrobact	67-14	NA
ASV_2491	0	0	0	0	0	0	0	0	0	0	0	0	Bacteria	Bdellovibrion	Bdellovibrion	Bacteriovora	Bacteriovora	Peredibacter
ASV_2492	0.33	0	0.33	0	0	0	0	0	0	0	0	0	Bacteria	Bacteroidota	Bacteroidia	Flavobacteria	Flavobacteria	Antarcticibacterium
ASV_2493	0	0	0	0	0	0	0	0	0	0	0	0	Archaea	Crenarchaeo	Thermoprote	Sulfolobales	Sulfolobacea	Stygiolobus
ASV_2494	0	0	0	0	0	0	0	0	0	0	0	0	Bacteria	Firmicutes	Bacilli	Paenibacillale	Paenibacillac	NA
ASV_2496	0	0	0	0	0	0	0	0	0	0	0	0	Bacteria	Bdellovibrion	Bdellovibrion	Bdellovibrion	Bdellovibrion	Bdellovibrio
ASV_2497	0	0	0.67	0.33	0	0	0	0	0	0	0	0	Bacteria	Bacteroidota	Bacteroidia	Sphingobacte	FFCH9454	NA
ASV_2498	0	0	0	0	0	0	0	0	0	0	0	0	Bacteria	Chloroflexi	TK10	NA	NA	NA
ASV_2499	0	0	0	0	0	0	0	0.33	0	0	0	0	Bacteria	Patescibacter	Saccharimon	Saccharimon	LWQ8	NA
ASV_2500	0.33	0	0	0.33	0	0	0	0	0	0	0	0	Bacteria	Proteobacter	Alphaproteol	Acetobactera	Acetobactera	Paracraurococcus
ASV_2501	0	0	0	0	0	0.33	0	0	0	0	0	0	Bacteria	Actinobacter	Actinobacter	0319-7L14	NA	NA
ASV_2502	0	0	0	0	0	0	0	0	0	0	0	0	Bacteria	Bacteroidota	Bacteroidia	Cytophagales	Cytophagace	Sporocytophaga
ASV_2503	0	0	0	0	0	0	0	0	0	0	0	0	Bacteria	Cyanobacteri	Cyanobacteri	NA	NA	NA
ASV_2504	0	0.67	0	0	0	0	0	0	0	0	0	0	Archaea	Crenarchaeo	Thermoprote	Sulfolobales	Sulfolobacea	Stygiolobus
ASV_2505	0	0	0	0	0	0	0	0	0	0	0	0	Bacteria	Proteobacter	Alphaproteol	Rhizobiales	Beijerinckiace	NA
ASV_2506	0	0	0	0	0	0	0	0	0	0	0	0	Bacteria	Firmicutes	Bacilli	Lactobacillale	Streptococca	Streptococcus
ASV_2507	0	0	0	0	0	0	0	0	0	0	0	0	Bacteria	Bacteroidota	Bacteroidia	Flavobacteria	Weeksellacea	Cloacibacterium
ASV_2508	0	0	0.67	0	0	0	0	0	0	0	0	0	Bacteria	Firmicutes	Bacilli	Staphylococc	Gemellaceae	Gemella
ASV_2509	0	0	0	0	0	0	0	0	0	0	0	0	Bacteria	Verrucomicro	Verrucomicro	Verrucomicro	Rubritaleacea	Luteolibacter
ASV_2510	0	0	0	0	0	0	0	0	0	0	0	0	Bacteria	Verrucomicro	Verrucomicro	Pedosphaera	Pedosphaera	NA
ASV_2511	0	0	0	0	0	0	0	0	0	0	0	0	Bacteria	NA	NA	NA	NA	NA
ASV_2512	0	0	0	0	0.33	0	0.5	0	0	0	0	0	Bacteria	Fibrobactero	Fibrobacteria	Fibrobacteral	Fibrobactera	possible genus 04
ASV_2513	0	0	0	0	0	0	0	0	0	0	0	0	Bacteria	Patescibacter	Saccharimon	Saccharimon	NA	NA
ASV_2514	0	0	0	0	0	0	0	0	0	0	0	0	Bacteria	Gemmatimor	Gemmatimor	Gemmatimor	Gemmatimor	NA
ASV_2515	0	0	0	0	0	0	0	0.67	0	0	0	0	Bacteria	Acidobacteri	Blastocatellia	Pyrinomonac	Pyrinomonad	RB41
ASV_2516	0	0	0	0	0	0	0	0	0	0	0	0	Bacteria	Firmicutes	Sulfobacillia	Sulfobacillale	Sulfobacillace	Sulfobacillus
ASV_2517	0.33	0	0	0.33	0	0	0	0	0	0	0	0	Bacteria	Abditibacteri	Abditibacteri	Abditibacteri	Abditibacteri	Abditibacterium
ASV_2518	0.33	0	0	0	0	0	0	0	0	0	0	0	Bacteria	Actinobacter	Acidimicrobii	IMCC26256	NA	NA
ASV_2519	0.33	0	0	0	0	0	0	0	0	0	0	0	Bacteria	Verrucomicro	Verrucomicro	Opitutales	Opitutaceae	Opitutus
ASV_2520	0	0	0.33	0	0	0	0	0	0	0	0	0	Bacteria	Proteobacter	Gammaprote	Pseudomona	Pseudomona	Pseudomonas
ASV_2521	0	0	0.67	0	0	0	0	0	0	0	0	0	Bacteria	Bacteroidota	Bacteroidia	Cytophagales	Cytophagace	Sporocytophaga
ASV_2522	0	0	0.33	0	0	0	0.5	0	0	0	0	0	Bacteria	Planctomycet	vadinHA49	NA	NA	NA
ASV_2523	0.33	0	0	0	0	0	0	0	0	0	0	0	Bacteria	Actinobacter	Rubrobacteri	Rubrobactera	Rubrobacteri	Rubrobacter
ASV_2524	0	0	0	0	0	0	1	0	0	0	0	0	Bacteria	Bacteroidota	Bacteroidia	Bacteroidales	Bacteroidete	NA
ASV_2525	0	0	0	0	0	0.33	0	0	0	0	0	0	Bacteria	Patescibacter	Parcubacteria	NA	NA	NA
ASV_2526	0	0	0.33	0	0	0	0	0	0	0	0	0	Bacteria	Planctomycet	Phycisphaera	Tepidisphaer	WD2101 soil	NA
ASV_2527	1.33	0	0	0	0	0	0	0	0	0	0	0	Archaea	Crenarchaeo	Thermoprote	Sulfolobales	Sulfolobacea	Stygiolobus
ASV_2528	0	0	0	0	0	0	0	0	0	0	0	0	Bacteria	Firmicutes	Bacilli	Caldalkalibaci	Caldalkalibaci	Caldalkalibacillus
ASV_2529	0	0.33	0	0	0	0	0	0.33	0	0	0.33	0	Bacteria	Proteobacter	Gammaprote	Pseudomona	Halomonada	Halomonas
ASV_2530	0	0	0	0	0	0	0	0	0	0	0	0	Bacteria	Firmicutes	Bacilli	Paenibacillale	Paenibacillac	Paenibacillus
ASV_2531	0	0	0	0	0	0	0.5	0	0	0	0	0	Bacteria	Bacteroidota	Bacteroidia	Cytophagales	Spirosomace	NA
ASV_2532	0	0	0	0	0	0	0	0	0	0	0	0	Bacteria	Actinobacter	Actinobacter	Propionibact	Nocardioidac	Nocardioides
ASV_2533	0	0	0	0	0	0	0.5	0	0	0	0	0	Bacteria	Chloroflexi	KD4-96	NA	NA	NA
ASV_2534	0	0	0	0	0	0	0	0	0	0	0	0	Bacteria	Planctomycet	Planctomycet	Gemmatales	Gemmatacea	Gemmata
ASV_2535	0	0	0	0	0	0	0	0	0	0	0	0	Bacteria	Patescibacter	NA	NA	NA	NA
ASV_2536	0	0	0	0	0	0	0	0	0	0	0	0	Bacteria	Chloroflexi	Chloroflexia	Thermobacul	Thermobacul	Thermobaculum
ASV_2537	0	0	0	0	0	0.33	0	0	0	0	0	0	Bacteria	Actinobacter	MB-A2-108	NA	NA	NA
ASV_2538	0	0	0	0	0	0.33	0	0	0	0	0	0.33	Bacteria	Myxococcota	Polyangia	Haliangiales	Haliangiaceae	Haliangium
ASV_2539	0	0	0	0	0	0	0	0	0	0	0	0	Bacteria	Actinobacter	MB-A2-108	NA	NA	NA
ASV_2540	0	0	0	0	0	0	0	0	0	0	0	0	Bacteria	Bacteroidota	Bacteroidia	Sphingobacte	Sphingobacte	NA
ASV_2541	0	0	0	0	0	0	0	0	0	0	0	0	Bacteria	Bacteroidota	Kapabacteria	Kapabacteria	NA	NA
ASV_2542	0	0	0.33	0	0	0	1	0	0	0	0	0	Bacteria	Chloroflexi	Chloroflexia	Thermomicro	JG30-KF-CM4	NA
ASV_2543	0	0	0	0	0	0	0	0	0	0	0	0	Bacteria	Myxococcota	Myxococcia	Myxococcale	Myxococcace	NA
ASV_2544	0	0	0	0	0	0	0	0	0	0	0	0.33	Bacteria	Planctomycet	Planctomycet	Pirellulales	Pirellulaceae	Pirellula
ASV_2545	0	0	0	0.33	0	0	0	0	0	0	0	0	Bacteria	Chloroflexi	NA	NA	NA	NA
ASV_2546	0	0	0	0	0	0	0	0	0	0	0	0	Bacteria	Patescibacter	Saccharimon	Saccharimon	LWQ8	NA
ASV_2547	0.67	0	0	0	0	0	0	0	0	0	0	0	Bacteria	Proteobacter	Gammaprote	Burkholderia	TRA3-20	NA
ASV_2548	0	0	0.33	0	0	0	0	0	0	0	0	0.67	Bacteria	Bdellovibrion	Oligoflexia	Oligoflexales	Oligoflexacea	NA
ASV_2550	0	0	0	0	0	0	0	0.67	0	0	0	0	Bacteria	Proteobacter	Gammaprote	Pseudomona	Halomonada	Halomonas
ASV_2551	0	0	0	0	0	0.33	0.5	0	0	0	0	0	Bacteria	Bacteroidota	Bacteroidia	Chitinophaga	Saprospirace	NA
ASV_2552	0	0	0	0	0	0	0	0	0	0	0	0	Bacteria	Myxococcota	Polyangia	Polyangiales	NA	NA
ASV_2553	0	0	0	0	0	0	0	0	0	0	0	0	Bacteria	Chloroflexi	TK10	NA	NA	NA
ASV_2554	0	0	0	0	0.67	0	0	0	0	0	0	0	Bacteria	Chloroflexi	Ktedonobact	Ktedonobact	Ktedonobact	NA
ASV_2555	0	0	0	0	0	0	0	0	0	0	0	0	Bacteria	Bdellovibrion	Oligoflexia	Silvanigrellale	Silvanigrellac	NA
ASV_2556	0	0	0	0	0	0.33	0	0	0	0	0	0	Bacteria	Chloroflexi	Ktedonobact	C0119	NA	NA
ASV_2557	0	0	0	0	0	0.67	0	0	0	0	0	0	Bacteria	Firmicutes	Sulfobacillia	Sulfobacillale	Sulfobacillace	Sulfobacillus
ASV_2558	0	0	0	0	0	0	0	0	0	0	0	0	Bacteria	Proteobacter	Alphaproteol	Acetobactera	Acetobactera	Acidiphilium
ASV_2559	0	0	0	0	0	0	1.5	0	0	0	0	0	Archaea	Crenarchaeo	Bathyarchaei	NA	NA	NA
ASV_2560	0	0	0	0	0	0	0	0	0	0	0	0	Bacteria	Actinobacter	Actinobacter	NA	NA	NA
ASV_2561	0	0	0	0	0	0	0	0	0	0	0	0	Bacteria	Armatimonac	NA	NA	NA	NA
ASV_2562	0	0	0	0	0	0	0	0	0	0	0	0	Bacteria	Bacteroidota	Bacteroidia	Chitinophaga	Chitinophaga	Flaviaesturariibacter
ASV_2563	0	0	0	0	0	0	0	0	0	0	0	0	Bacteria	Patescibacter	NA	NA	NA	NA
ASV_2564	0	0	0	0	0	0.33	0	0	0	0	0	0	Bacteria	Gemmatimor	BD2-11 terre	NA	NA	NA
ASV_2565	0	0	0.67	0	0	0	0	0	0	0	0	0	Bacteria	Planctomycet	Planctomycet	Gemmatales	Gemmatacea	NA
ASV_2566	0	0	0	0	0	0	0	0.33	0	0	0	0	Bacteria	Planctomycet	Planctomycet	Isosphaerales	Isosphaerace	NA
ASV_2567	0.33	0	0	0	0	0	0	0	0	0	0	0	Bacteria	Proteobacter	Gammaprote	Pseudomona	Halomonada	Halomonas
ASV_2568	0	0	0	0	0	0	0	0	0	0	0	0	Bacteria	Chloroflexi	Chloroflexia	Kallotenuales	AKIW781	NA
ASV_2569	0	0	0	0	0	0	0	0	0	0	0	0	Archaea	Crenarchaeo	Thermoprote	Sulfolobales	Sulfolobacea	Stygiolobus
ASV_2570	0	0	0	0	0	0	0	0	0	0	0	0	Bacteria	Actinobacter	Actinobacter	Propionibact	Propionibact	Cutibacterium
ASV_2571	0.33	0	0	0	0.67	0	0	0	0	0	0	0	Bacteria	Verrucomicro	Verrucomicro	Verrucomicro	Verrucomicro	NA
ASV_2572	0	0	0	0	0	0	0	0	0	0	0	0	Bacteria	Actinobacter	Actinobacter	0319-7L14	NA	NA
ASV_2573	0	0	0	0	0	0	0.5	0	0	0	0	0	Bacteria	Actinobacter	Acidimicrobii	Microtrichale	Ilumatobacte	NA
ASV_2574	0	0	0	0	0	0	0	0	0	0	0	0	Bacteria	Bdellovibrion	Bdellovibrion	Bdellovibrion	Bdellovibrion	Bdellovibrio
ASV_2575	0	0	0	0	0	0	0	0	0	0	0	0	Bacteria	Actinobacter	Thermoleoph	Gaiellales	NA	NA
ASV_2576	0	0	0	0	0	0.67	0	0	0	0	0	0	Bacteria	Chloroflexi	TK10	NA	NA	NA
ASV_2577	0	0	0	0	0.33	0	0	0	0	0	0	0	Bacteria	Bacteroidota	Bacteroidia	Flavobacteria	Flavobacteria	Flavobacterium
ASV_2578	0	0	0	0	0	0.33	0	0	0	0	0	0	Bacteria	Chloroflexi	Chloroflexia	Thermomicro	NA	NA
ASV_2579	0	0	0	0	0	0	0	0	0	0	0	0	Bacteria	Chloroflexi	Chloroflexia	Thermomicro	JG30-KF-CM4	NA
ASV_2580	0	0	0	0	0	0.33	0	0	0	0	0	0	Bacteria	Proteobacter	Alphaproteol	Acetobactera	Acetobactera	NA
ASV_2581	0	0	0	0	0	0	0	0	0	0	0	0	Bacteria	Bdellovibrion	Oligoflexia	Oligoflexales	Oligoflexacea	NA
ASV_2582	0	0	0	0	1	0	0	0	0	0	0.67	0	Bacteria	Acidobacteri	Blastocatellia	Blastocatellal	Blastocatellac	Blastocatella
ASV_2583	0	0	0	0	0.67	0	0	0	0	0	0	0	NA	NA	NA	NA	NA	NA
ASV_2584	0	0	0	0	0	0	0	0	0	6	0	0	Bacteria	Actinobacter	Thermoleoph	NA	NA	NA
ASV_2585	0	0	0	0	0	0	0	0	0	0	0	0	Bacteria	Actinobacter	Thermoleoph	Gaiellales	NA	NA
ASV_2586	0	0	0	0	0	0	0	0	0	0	0	0	Bacteria	Proteobacter	Gammaprote	Burkholderia	Nitrosomona	MND1
ASV_2587	0.33	0	0.33	0	0	0	0	0	0	0	0	0	Bacteria	Bdellovibrion	Bdellovibrion	Bacteriovora	Bacteriovora	Bacteriovorax
ASV_2588	0	0	0	0	0	0	0	0	0	0	0	0	Bacteria	Bdellovibrion	Bdellovibrion	Bacteriovora	Bacteriovora	Peredibacter
ASV_2589	0	0	0	0	0	0	0	0	0	0	0	0	Bacteria	Proteobacter	Gammaprote	Pseudomona	Pseudomona	Pseudomonas
ASV_2592	0	0	0	0	0	0	0	0	0	0	0	0	Bacteria	Verrucomicro	Chlamydiae	Chlamydiales	Parachlamydi	Neochlamydia

ASV_2593	0	0	0	0	0	0	1	0	0	0	0	0	Bacteria	Bdellovibrion	Oligoflexia	Oligoflexales	Oligoflexacea	NA
ASV_2594	0	0	0	0.33	0	0	0	0	0	0	0	0	Bacteria	Gemmatimor	Gemmatimor	Gemmatimor	Gemmatimor	Gemmatimonas
ASV_2595	0	0	0	1	0	0	0	0	0	0	0	0	Archaea	Crenarchaeo	Thermoprote	Sulfolobales	Sulfolobacea	Stygiolobus
ASV_2596	0	0	0	0	0	0.33	0	0	0	0	0	0	Bacteria	Firmicutes	Sulfobacillia	Sulfobacillale	Sulfobacillace	NA
ASV_2597	0	0	0	0	0	0	0	0	0	0	0	0	Bacteria	Chloroflexi	Chloroflexia	Thermomicro	JG30-KF-CM4	NA
ASV_2598	0	0	0.33	0	0	0	0	0	0	0	0	0	Bacteria	NA	NA	NA	NA	NA
ASV_2599	0	0	0.33	0	0	0	0	0	0	0	0	0	Bacteria	Abditibacteri	Abditibacteri	Abditibacteri	Abditibacteri	Abditibacterium
ASV_2600	0	0	0	0	0	0	0	0	0	0	0	0	Bacteria	Proteobacter	Gammaprote	Diplorickettsi	Diplorickettsi	NA
ASV_2601	0	0	0	0	0	0	0	0	0	0	0	0	Bacteria	Chloroflexi	TK10	NA	NA	NA
ASV_2602	0	0	0	0	0	0	0	0	0	0	0	0	Bacteria	Patescibacter	Saccharimon	Saccharimon	Saccharimon	TM7a
ASV_2604	0	0	0	0	0	0	0	0.33	0	0	0	0	Bacteria	Verrucomicro	Verrucomicro	Verrucomicro	Verrucomicro	NA
ASV_2605	0	0	0	0	0.67	0	0	0	0	0	0	0	Bacteria	Chloroflexi	Chloroflexia	Thermomicro	AKYG1722	NA
ASV_2606	0	0	0	0	0	0	1	0	0	0	0	0	Bacteria	Gemmatimor	Gemmatimor	Gemmatimor	Gemmatimor	NA
ASV_2607	0	0	0	0	0	0	2	0	0	0	0	0	Bacteria	Actinobacter	Actinobacter	Corynebacter	Corynebacter	Corynebacterium
ASV_2608	0	0	0	0	0	0	0	1.33	0	0	0	0	Bacteria	Actinobacter	Actinobacter	Frankiales	NA	NA
ASV_2609	0	0	0	0	0	0	0	0	0	0	0	0	Bacteria	Firmicutes	NA	NA	NA	NA
ASV_2610	0.33	0	0	0	0	0	0	0	0	0	0	0	Bacteria	Proteobacter	Alphaproteol	Rhizobiales	Beijerinckiace	Rhodoblastus
ASV_2611	0	0	0	0	0	0	0	0	0	0	0	0	Bacteria	Patescibacter	Saccharimon	Saccharimon	NA	NA
ASV_2612	0	0	0	0	0	0	0	0.33	0	0	0	0	Bacteria	Patescibacter	Saccharimon	Saccharimon	NA	NA
ASV_2613	0	0	0.67	0	0	0	0	0	0	0	0	0	Bacteria	Planctomycet	Planctomycet	Pirellulales	Pirellulaceae	Roseimaritima
ASV_2614	0	0	0	0	0	0	1.5	0	0	0	0	0	Bacteria	Armatimonac	NA	NA	NA	NA
ASV_2615	0.33	0	0	0	0	0	0	0	0	0	0	0	Bacteria	Proteobacter	Gammaprote	NA	NA	NA
ASV_2616	0	0	1	0	0	0	0	0	0	0	0	0	Bacteria	Bacteroidota	Bacteroidia	Bacteroidete	NA	NA
ASV_2617	0	0	0	0	0	0	0	0	0	0	0	0	Bacteria	Abditibacteri	Abditibacteri	Abditibacteri	Abditibacteri	Abditibacterium
ASV_2618	0	0.67	0	0	0	0	0	0	0	0	0	0	Bacteria	Proteobacter	Gammaprote	EV818SWSAF	NA	NA
ASV_2619	0	0	0	0	0	0	0	0	0	0	0	0	Bacteria	Gemmatimor	Gemmatimor	Gemmatimor	Gemmatimor	NA
ASV_2620	0	0	0	0	0	0	0	0	0	0	0	0	Bacteria	Chloroflexi	Chloroflexia	Thermomicro	JG30-KF-CM4	NA
ASV_2621	0	0	0	0	0	0	0	0	0	0	0	0	Bacteria	Sumerlaeota	Sumerlaeia	Sumerlaeales	Sumerlaeace	Sumerlaea
ASV_2622	0	0	0	0	0	0	0	0	0	0	0	0	Bacteria	Proteobacter	Gammaprote	Acidiferrobac	Acidiferrobac	NA
ASV_2623	0	0	0	0	0	0	0	0	0	0	0	0	Bacteria	Chloroflexi	Chloroflexia	Thermomicro	JG30-KF-CM4	NA
ASV_2624	0	0	0	0	0	0	0	0	0	0	0	0	Bacteria	NA	NA	NA	NA	NA
ASV_2625	0	0	0	0.33	0	0	0	0	0	0	0	0	Bacteria	Firmicutes	Bacilli	Alicyclobacill	Alicyclobacill	Tumebacillus
ASV_2626	0	0	0	0	0	0	0	0	0	0	0	0	Bacteria	Actinobacter	Thermoleoph	Gaiellales	NA	NA
ASV_2627	0	0	0	0	0	0	0	0	0	0	0	0	Bacteria	Planctomycet	Planctomycet	Planctomycet	NA	NA
ASV_2628	0	0	0	0	0	0	0.5	0	0	0	0	0	Bacteria	Actinobacter	Thermoleoph	Solirubrobact	Solirubrobact	NA
ASV_2629	0	0	0	0	0	0	0	0.33	1	0	0	0	Bacteria	Patescibacter	Saccharimon	Saccharimon	LWQ8	NA
ASV_2630	0	0	0	0	0	0	1.5	0	0	0	0	0	Bacteria	Acidobacteri	Acidobacteri	Acidobacteri	Acidobacteri	NA
ASV_2632	0	0	0	0	0	0	0	0	0	0	0	0.33	Bacteria	Firmicutes	Bacilli	Lactobacillale	Streptococca	Streptococcus
ASV_2633	0	0	0	0	0	0	0	0	0	0	0	0	Bacteria	Firmicutes	Bacilli	Bacillales	Bacillaceae	Anaerobacillus
ASV_2634	0	0	0	0	0	0	0	0	0	0	0	0	Bacteria	Planctomycet	Planctomycet	Gemmatales	Gemmatacea	Gemmata
ASV_2635	0	0	0	0	0	0	0	0	0	0	0	0	Bacteria	NA	NA	NA	NA	NA
ASV_2636	0	0	0.33	0	0	0	0	0	0	0	0	0	Bacteria	Proteobacter	Gammaprote	Pseudomona	Pseudomona	Pseudomonas
ASV_2637	0	0	0	0	0	0	0	0	0	0	0	0	Bacteria	Proteobacter	Alphaproteol	Paracaedibac	Paracaedibac	Candidatus Paracaedibacter
ASV_2638	0	0	0	0	0	0	0	0	0	0	0	0	Archaea	Crenarchaeo	Thermoprote	Sulfolobales	Sulfolobacea	NA
ASV_2639	0	0	0	0	0	0	0	0	0	0	0	0	Bacteria	Proteobacter	Gammaprote	Enterobacter	Enterobacter	NA
ASV_2640	0	0	0	0	0	0	0	0	0	0	0	0	Bacteria	Patescibacter	Saccharimon	Saccharimon	NA	NA
ASV_2641	0	0	0	0	0	0	0	0	0	0	0	0	Bacteria	Actinobacter	Actinobacter	Corynebacter	Corynebacter	Corynebacterium
ASV_2643	0	0	0	0	0	0	0	0	0	0	0	0	Bacteria	Bacteroidota	Bacteroidia	Flavobacteria	Weeksellacea	NA
ASV_2644	0	0	0	0	0	0	0	0	0	0	1.33	0	Bacteria	Proteobacter	Gammaprote	Enterobacter	Aeromonada	Oceanisphaera
ASV_2645	0	0	0.67	0	0	0	0	0	0	0	0	0	Bacteria	Planctomycet	Planctomycet	Planctomycet	NA	NA
ASV_2647	0	0	0	0	0	0	0	0	0	2	0	0	Bacteria	Proteobacter	Gammaprote	Burkholderia	Burkholderia	Ralstonia
ASV_2648	0	0	0	0	0	0	0	0	0	0	0	0	Bacteria	Firmicutes	Thermoanae	Caldicellulosi	Caldicellulosi	Caldicellulosiruptor
ASV_2649	0	0	0	0	0	0	0	0	0	0	0	0	Bacteria	Bacteroidota	Bacteroidia	Sphingobacte	env.OPS 17	NA
ASV_2651	0	0	0	0	0	0	0	0	0	0	0	0	Bacteria	Proteobacter	Gammaprote	Pseudomona	Halomonada	Halomonas
ASV_2652	0	0	0	0	0	0	0	0	0	0	0	0	Bacteria	Chloroflexi	Ktedonobact	Ktedonobact	Ktedonobact	FCPS473
ASV_2653	0	0	0	0	0	0	0	0	0	0	0	0	Bacteria	Verrucomicro	Verrucomicro	Verrucomicro	DEV007	NA
ASV_2654	0	0	0	0	0	0	0	0	0	0	0	0	Bacteria	Planctomycet	Planctomycet	Planctomycet	Schlesneriace	Schlesneria
ASV_2658	0	0	0	0	0	0	0	0	0	2	0	0	Bacteria	Bacteroidota	Bacteroidia	Chitinophaga	Chitinophaga	NA
ASV_2659	0	0	0	0	0	0	0	0	0	0	0	0	Bacteria	Acidobacteri	Blastocatellia	Blastocatellal	Blastocatellac	Blastocatella
ASV_2660	0	0	0	0	0	0	0	0	0	0	0	0	Bacteria	Myxococcota	Polyangia	mle1-27	NA	NA
ASV_2661	0	0	0.33	0	0	0	0.5	0	0	0	0	0	Bacteria	Armatimonac	Chthonomon	Chthonomon	NA	NA
ASV_2663	0	0	0	0	0	0	0	0	0	0	0	0	Bacteria	Proteobacter	Alphaproteol	NA	NA	NA
ASV_2664	0	0	0	0	0.33	0	0	0	0	0	0	0	Archaea	Thermoplasn	Thermoplasn	Thermoplasn	Thermoplasn	A-plasma
ASV_2665	0	0	0	0	0	0.33	0	0	0	0	0	0	Bacteria	Chloroflexi	NA	NA	NA	NA
ASV_2666	0	0	0	0	0	0	0	0	0	0	0	0	Bacteria	Bacteroidota	Bacteroidia	Bacteroidales	Porphyromor	Porphyromonas
ASV_2667	0	0	0	0	0	0	0	0	0	0	0	0	Bacteria	Chloroflexi	Ktedonobact	Ktedonobact	Ktedonobact	Thermosporothrix
ASV_2668	0	0	0	0	0	0	0	0	0	0	0	0	Bacteria	Actinobacter	Actinobacter	Corynebacter	Nocardiaceae	Millisia
ASV_2670	0	0	0	0	0	0	0	0	0	0	0	0	Bacteria	Chloroflexi	Chloroflexia	Thermomicro	Thermomicro	Thermorudis
ASV_2671	0	0	0	0	0	0	0	0	0	0	0	0	Bacteria	Firmicutes	NA	NA	NA	NA
ASV_2672	0	0	0	0	0	0	0	0	0	0	0	0.33	Archaea	Halobacterot	Methanosarc	Methanosarc	Methanosaet	Methanosaeta
ASV_2674	0	0	0	0	0	0	0	0	0	0	0	0	Bacteria	Bacteroidota	Bacteroidia	Bacteroidales	Prevotellacea	Alloprevotella
ASV_2676	0	0	0	0	0	0	0	0	0	0	0	0	Bacteria	Proteobacter	Gammaprote	Pseudomona	Pseudomona	Pseudomonas
ASV_2680	0	0	0.67	0	0	0	0	0	0	0	0	0	Bacteria	Proteobacter	Gammaprote	Pseudomona	Pseudomona	Pseudomonas
ASV_2681	0	0	0	0	0	0	0	0	0	0	0	0	Bacteria	Cyanobacteri	Sericytochroi	NA	NA	NA
ASV_2682	0	0	0	0	0	0	0	0	0	0	0	0	Bacteria	Bacteroidota	Bacteroidia	Sphingobacte	KD3-93	NA
ASV_2686	0	0	0	0	0	0	0	0.33	0	0	0	0	Bacteria	Proteobacter	Gammaprote	Pseudomona	Halomonada	Halomonas
ASV_2687	0	0	0	0	0	0	0	0	0	0	0	0	Bacteria	Firmicutes	Bacilli	Thermicanale	Thermicanac	Thermicanus
ASV_2692	0	0	0	0	0	0	0	0	0	0	0	0	Bacteria	Proteobacter	Gammaprote	Pseudomona	Pseudomona	Pseudomonas
ASV_2694	0	0	0	0	0	0	0	0	0	0	0	0	Archaea	Crenarchaeo	Thermoprote	Sulfolobales	Sulfolobacea	Stygiolobus
ASV_2695	0.33	0	0	0	0	0	0	0	0	0	0	0	Bacteria	Firmicutes	Bacilli	Staphylococc	Staphylococc	Staphylococcus
ASV_2696	0.33	0	0	0	0	0	0	0	0	0	0	0	Archaea	Crenarchaeo	Thermoprote	Sulfolobales	Sulfolobacea	Stygiolobus
ASV_2697	0	0.33	0	0	0	0	0	0	0	0	0	0	Bacteria	Proteobacter	Gammaprote	Pseudomona	Halomonada	Halomonas
ASV_2699	0	0	0	0	0	0	0	0	0	0	0	0	Bacteria	Proteobacter	Gammaprote	Pseudomona	Halomonada	Halomonas
ASV_2701	0	0	0	0	0	0	0	0	0	0	0	0	Archaea	Crenarchaeo	Nitrososphae	Group 1.1c	NA	NA
ASV_2702	0	0	0	0	0	0	0	0	0	0	0	0	Bacteria	Myxococcota	Polyangia	Polyangiales	Sandaracinac	Sandaracinus
ASV_2703	0	0	0	0	0	0	0	0	0	0	0	0	Archaea	Crenarchaeo	Thermoprote	Sulfolobales	Sulfolobacea	Stygiolobus
ASV_2704	0	0	0	0	0	0	0	0	0	0	0	0	Bacteria	NA	NA	NA	NA	NA
ASV_2708	0	0	0	0	0	0	0	0	0	0	0	0	Archaea	Crenarchaeo	Nitrososphae	NA	NA	NA
ASV_2712	0	0	0	0	0	0	0	0	0	0	0	0	Bacteria	Proteobacter	Gammaprote	Pseudomona	Moraxellacea	Acinetobacter
