# Supplementary material for: Small-Scale Mineral and Microbial Heterogeneities near a Fumarole at the Furnas Hydrothermal Zone on the Azores
Source: Life (Basel). 2026 Jun 28;16(7):1086. doi: 10.3390/life16071086 (PMC13412118; doi:10.3390/life16071086)
Supplement: Supplementary file 1 [file life-16-01086-s001.zip › Supplement 3 - Table S3.pdf]

ASV	Taxonomy by BLAST against NCBI nt<br>Best hit taxa	Identity
ASV_1	uncultured archaeon	99.20%
ASV_3	uncultured archaeon	98.40%
ASV_4	Mycobacterium botniense	100%
ASV_5	Stygiolobus caldivivus/uncultured archaeon	100%
ASV_7	uncultured delta proteobacterium	97.20%
ASV_8	uncultured archaeon	97.60%
ASV_9	uncultured Thermoplasmatales archaeon	100%
ASV_10	uncultured Thermogymnomonas sp.	100%
ASV_11	Pseudomonas gessardii	100%
ASV_12	Ralstonia pickettii	100%
ASV_13	uncultured Thermogymnomonas sp.	100%
ASV_15	uncultured Thermogymnomonas sp.	100%
ASV_16	Mycobacterium - several species best hits	100%
ASV_17	Staphylococcus - several species best hits	100%
ASV_18	uncultured Thermogymnomonas sp.	100%
ASV_19	Mycobacterium xenopi/Mycobacterium heckeshornense	100%
ASV_20	uncultured archaeon	98.00%
ASV_21	uncultured archaeon	98%
ASV_22	uncultured archaeon	100%
ASV_23	Enterobacter/Klebsiella - several species best hits	100%

**Taxonomy assignment using Naive Bayes Classifier and SILVA v138.1 Kingdom;Phylum;Class;Order;Family;Genus**
Archaea;Crenarchaeota;Thermoprotei;Sulfolobales;Sulfolobaceae;Stygiolobus
Archaea;Crenarchaeota;Thermoprotei;Sulfolobales;Sulfolobaceae;
Bacteria;Actinobacteriota;Actinobacteria;Corynebacteriales;Mycobacteriaceae;Mycobacterium
Archaea;Crenarchaeota;Thermoprotei;Sulfolobales;Sulfolobaceae;Stygiolobus
Bacteria;RCP2-54;;;;
Archaea;Crenarchaeota;Nitrososphaeria;Group 1.1c;;
Archaea;Thermoplasmatota;Thermoplasmata;Thermoplasmatales;Thermoplasmataceae;A-plasma
Archaea;Thermoplasmatota;Thermoplasmata;Thermoplasmatales;Thermoplasmataceae;A-plasma
Bacteria;Proteobacteria;Gammaproteobacteria;Pseudomonadales;Pseudomonadaceae;Pseudomonas
Bacteria;Proteobacteria;Gammaproteobacteria;Burkholderiales;Burkholderiaceae;Ralstonia
Archaea;Thermoplasmatota;Thermoplasmata;Thermoplasmatales;Thermoplasmataceae;A-plasma
Archaea;Thermoplasmatota;Thermoplasmata;Thermoplasmatales;Thermogymnomonas;
Bacteria;Actinobacteriota;Actinobacteria;Corynebacteriales;Mycobacteriaceae;Mycobacterium
Bacteria;Firmicutes;Bacilli;Staphylococcales;Staphylococcaceae;Staphylococcus
Archaea;Thermoplasmatota;Thermoplasmata;BSLdp215;;
Bacteria;Actinobacteriota;Actinobacteria;Corynebacteriales;Mycobacteriaceae;Mycobacterium
Archaea;Thermoplasmatota;Thermoplasmata;Thermoplasmatales;Thermoplasmataceae;A-plasma
Archaea;Crenarchaeota;Nitrososphaeria;Group 1.1c;;
Bacteria;;;;;
Bacteria;Proteobacteria;Gammaproteobacteria;Enterobacterales;Enterobacteriaceae;Klebsiella
