# Supplementary figures and images for: Small-Scale Mineral and Microbial Heterogeneities near a Fumarole at the Furnas Hydrothermal Zone on the Azores
Source: Life (Basel). 2026 Jun 28;16(7):1086. doi: 10.3390/life16071086 (PMC13412118; doi:10.3390/life16071086)

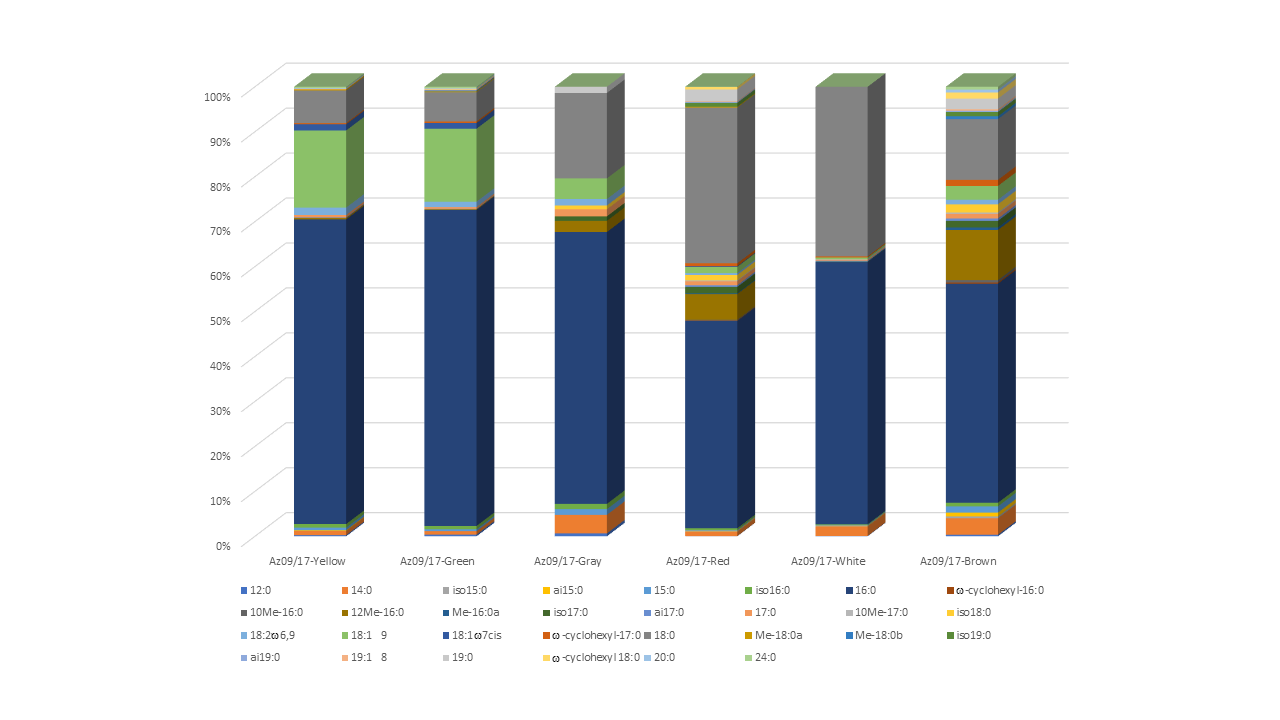

Supplement: Supplementary file 1 [file life-16-01086-s001.zip › Supplementary Figure S1.PNG]
